# Triple‐Helix‐Stabilizing Effects in Collagen Model Peptides Containing PPII‐Helix‐Preorganized Diproline Modules

**DOI:** 10.1002/anie.201914101

**Published:** 2020-02-03

**Authors:** Andreas Maaßen, Jan M. Gebauer, Elena Theres Abraham, Isabelle Grimm, Jörg‐Martin Neudörfl, Ronald Kühne, Ines Neundorf, Ulrich Baumann, Hans‐Günther Schmalz

**Affiliations:** ^1^ University of Cologne Department of Chemistry Greinstraße 4 50939 Cologne Germany; ^2^ University of Cologne Department of Chemistry Zülpicher Straße 47a 50674 Cologne Germany; ^3^ Leibniz-Institut für Molekulare Pharmakologie (FMP) Campus Berlin-Buch Robert-Rössle-Straße 10 13125 Berlin Germany

**Keywords:** collagen, HSP47, peptidomimetics, protein folding, triple helix stability

## Abstract

Collagen model peptides (CMPs) serve as tools for understanding stability and function of the collagen triple helix and have a potential for biomedical applications. In the past, interstrand cross‐linking or conformational preconditioning of proline units through stereoelectronic effects have been utilized in the design of stabilized CMPs. To further study the effects determining collagen triple helix stability we investigated a series of CMPs containing synthetic diproline‐mimicking modules (ProMs), which were preorganized in a PPII‐helix‐type conformation by a functionalizable intrastrand C_2_ bridge. Results of CD‐based denaturation studies were correlated with calculated (DFT) conformational preferences of the ProM units, revealing that the relative helix stability is mainly governed by an interplay of main‐chain preorganization, ring‐flip preference, adaptability, and steric effects. Triple helix integrity was proven by crystal structure analysis and binding to HSP47.

## Graphical abstract

**Collagen model peptides** represent attractive materials for biomedical applications. To rationally design peptide properties, knowledge about the impact of structural modifications is fundamental. Therefore, the impact of covalent diproline rigidification was studied and correlated with theoretical findings to understand the symphony of (de)stabilizing effects in the triple helix.

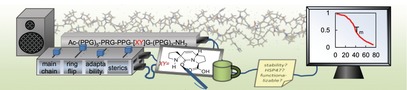

## Introduction

Collagen is the most abundant structural protein in animals, comprising a family of 28 known members differing in their composition and supramolecular assembly. By forming fibrils and networks, this main component of the extracellular matrix resides in skin, bone, and other tissues. Because of its unique biomechanical properties, collagen guarantees the structural integrity of vertebrates.^1^, ^2^, ^3^ In addition, collagen interacts with numerous proteins such as cell‐surface receptors or matrix metalloproteinases and is involved in processes such as cell adhesion, proliferation, and extracellular matrix regulation.^4^, ^5^ The biocompatibility and bioactivity of collagen together with advances in synthetic substitutes^6^, ^7^ open the path for biomedical applications such as bone grafts,^8^, ^9^ wound dressings,^10^, ^11^ engineering of functional tissues,^12^, ^13^ tendon repair,^14^ or inhibition of disease‐related target proteins.^4^, ^15^ In the context of diseases, pathological conditions are coined by structural defects and impaired collagen stability (e.g., osteogenesis imperfecta).^16^, ^17^ A stable structure and correct folding are therefore of fundamental importance for molecular recognition and function of collagen. At the molecular level, collagen forms a right‐handed triple helix consisting of three peptide strands, which adopt a left‐handed polyproline II (PPII) type helix conformation and are held together by interstrand hydrogen bonds.^18^ A repetitive unit of three amino acids [Xxx‐Yyy‐Gly]_*n*_ with a conserved glycine (Gly, G) and all‐*trans* amide bonds is observed. In the first two positions (Xxx and Yyy), proline (Pro, P) or (*4R*)‐hydroxyproline (Hyp, O) are predominantly found.^1^ Hyp can be introduced by prolyl‐4‐hydroxylase‐mediated functionalization of proline residues.^3^ Another member of the machinery involved in the complex biosynthesis of collagen is the chaperone HSP47. This essential heat shock protein transiently and exclusively binds to triple helical collagen to stabilize its conformation.^3^, ^19^ Upon binding, an arginine–aspartate salt bridge was observed in a model system.^20^

**Figure 1:**
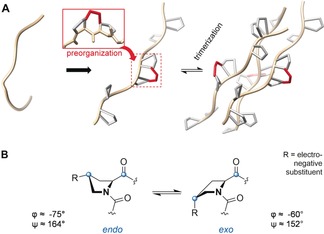
A) Chain preorganization of a CMP by introducing an intrastrand C_2_ bridge (red) between pairs of prolines and triple helix folding of the chains. B) Cγ *endo*/*exo* ring‐flip equilibrium of proline units. (PPG)_10_ served as a structural basis.^21^

Collagen model peptides (CMPs) adhering to the [XxxYyyGly]_*n*_ motif have been used to study the interactome, the structure, and structure–stability relationships of collagen.^1^, ^5^ Various CMPs with different amino acid substitutions have been prepared using preorganization as a guiding design principle.^2^ Generally, the folding of three peptide chains into a triple helix is entropically unfavorable. However, if the tendency of the free peptide strands to adopt a PPII‐helix‐related conformation is increased, the entropic cost for folding is decreased (Figure 1 A). Accordingly, the triple‐helical state can be promoted by proper preorganization. In this context, the structural preferences (puckering) of the proline pyrrolidine rings deserve special attention (Figure 1 B). Crystal structures of proline‐rich CMPs like (PPG)_10_ underlined that a Cγ‐*endo* conformation is found in the Xxx position and Cγ‐*exo* in the Yyy position.^2^, ^21^ As substituents influence the ring‐flip preference and, accordingly, also the main‐chain conformation, substitution at Cγ of the proline ring has become a strategy to improve preorganization in CMPs.^22^

As an example, 4*R*‐hydroxylation in Yyy position resulted in increased triple‐helix stability by promoting both an *exo*‐ring pucker and a high *trans*/*cis* ratio of the preceding amide bond. The underlying stereoelectronic effect^2^, ^23^ was also exploited using (4*R*)‐fluoroproline.^24^, ^25^ Steric effects contribute to triple‐helix stability as well, for instance, in the case of 4*S*‐methylproline.^2^, ^25^ A particularly strong helix stabilization was recently achieved by covalent interstrand crosslinking.^26^ However, collagen triple‐helix stability still remains unpredictable in many cases and deserves further investigation.^2^

**Figure 2:**
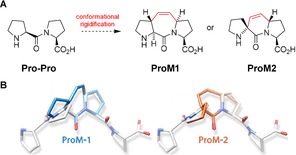
Design of **ProM1** and **ProM2**. A) Conformational rigidification of a Pro‐Pro unit by stereo‐controlled introduction of an ethylidene bridge (intrastrand bridging). B) Section of an idealized PPII helix (white) to show the structural fit upon replacement of two prolines by either **ProM1** or **ProM2**.

In the course of our previous studies aiming at the development of small‐molecule inhibitors of the PPII helix recognizing Ena/VASP homology 1 (EVH1) domain, we developed proline‐derived modules (ProMs) **ProM1** and **ProM2** (Figure 2).^27^, ^28^, ^29^, ^30^ The design is based on the stereo‐defined covalent connection of two adjacent proline rings by a C_2_ bridge to freeze the system in a PPII‐helix‐type conformation.

We now reasoned that scaffolds derived from **ProM1** and **ProM2**, respectively, would represent interesting building blocks (as Pro‐Pro substitutes) for the construction of CMPs. Initially, we only expected an entropic gain due to the (partial) preorganization of the PPII helix by intrastrand bridging. However, functionalization of the alkene unit additionally allowed the synthesis of structurally related ProMs differing in their conformational preferences, flexibility, bulkiness, and hydrophilicity. Herein, we report the results of a systematic study that indeed led to an improved understanding of the factors contributing to CMP triple‐helix stability—as a subtle interplay of different effects.

## Results and Discussion

### Synthesis of ProM‐Derived Scaffolds

We started our investigation with the gram‐scale synthesis of Boc‐**ProM1**‐O*t*Bu following previously published procedures.^27^, ^29^ To facilitate solid phase peptide synthesis (SPPS) of CMPs (see below), a sample of Boc‐**ProM1**‐O*t*Bu was converted into the corresponding Fmoc‐protected free acid (Fmoc‐**ProM1**‐OH) in a one‐pot procedure by TFA‐induced cleavage of the acid‐labile Boc and *tert*‐butyl ester groups and subsequent reprotection of the N‐terminus with FmocCl in the presence of NaHCO_3_.

**Figure 3:**
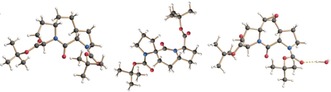
Structures of Boc‐**H_2_‐ProM1**‐O*t*Bu, Boc‐**H_2_‐ProM2**‐O*t*Bu, and Boc‐**ep‐ProM1**‐O*t*Bu in the crystalline state.

**Scheme 1:**
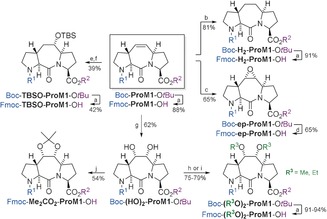
Synthesis of **ProM1**‐derived building blocks. Reagents and conditions: a) TFA; then FmocCl, NaHCO_3_, H_2_O/THF, 25 °C, 22 h; b) H_2_, Pd/C, MeOH, 25 °C, 10 min; c) oxone®, 1,1,1‐trifluoroacetone, NaHCO_3_, MeCN, 15 °C, 4.5 h; d) TFA, H_2_O, TIPS; then FmocCl, NaHCO_3_, H_2_O/THF, 18 °C, 2 h; e) BH_3_⋅Me_2_S; then H_2_O_2_, aq. NaOH, THF, 0 °C to 25 °C, 2 h, 70 %; f) TBSOTf, NEt_3_, CH_2_Cl_2_, −78 °C, 40 min, 55 %; g) RuCl_3_⋅3 H_2_O, CeCl_3_, NaIO_4_, EtOAc/MeCN/H_2_O, 0 °C, 10 min; h) NaH, MeI, DMF, 0 °C to 25 °C, 2 h; i) NaH, EtI, DMF, 0 °C to 25 °C, 15 h; j) TFA, H_2_O, TIPS; then FmocCl, NaHCO_3_, H_2_O/THF, 25 °C, 18 h; then 2,2‐dimethoxypropane, *p*‐TsOH, acetone/CH_2_Cl_2_, 25 °C, 6 h. FmocCl=fluorenylmethyloxycarbonyl chloride, TBSOTf=*tert*‐butyldimethylsilyl trifluoromethanesulfonate, TIPS=triisopropylsilane.

As a first C_2_ linker variation (Scheme 1), Boc‐**ProM1**‐O*t*Bu was subjected to Pd‐catalyzed catalytic hydrogenation to afford Boc‐**H_2_‐ProM1**‐O*t*Bu as a crystalline compound, which was characterized by X‐ray crystallography (Figure 3). Its conversion into Fmoc‐**H_2_‐ProM1**‐OH then proceeded smoothly under the standard conditions. While other reagents, such as MCPBA, gave unsatisfactory results, epoxidation of the C=C double bond of Boc‐**ProM1**‐O*t*Bu was achieved by dioxirane generated in situ from oxone® and trifluoroacetone at 15 °C.^31^ Noteworthy, the α‐epoxide (Boc‐**ep‐ProM1**‐O*t*Bu) was obtained as a single diastereomer. The configuration was assigned through NOE NMR experiments and confirmed by crystal‐structure analysis (Figure 3). Because of the sensitivity of the epoxide function, triisoproylsilane was added during TFA treatment in the preparation of Fmoc‐**ep‐ProM1**‐OH. Hydroboration of Boc‐**ProM1**‐O*t*Bu followed by oxidation led to an inseparable mixture of regioisomeric alcohols. However, after reacting this mixture with TBSOTf/NEt_3_ at low temperature, the silyl ether Boc‐**TBSO‐ProM1**‐O*t*Bu was isolated as the sole isomer, and its structure was elucidated by NMR analysis (see the Supporting Information, Figure S2). It is noteworthy that the OTBS group remained untouched under the standard trans‐protection conditions to give access to Fmoc‐**TBSO‐ProM1**‐OH. First attempts to functionalize the C_2_ bridge of Boc‐**ProM1**‐O*t*Bu through 1,2‐dihydroxylation employing Sharpless AD mix (α or β)^32^ only resulted in slow conversion, even when using increased amounts of K_2_OsO_2_(OH)_4_. In contrast, the CeCl_3_‐improved method to in situ generate RuO_4_ from RuCl_3_/NaIO_4_ proved to be successful.^33^ Upon increasing the RuCl_3_ loading from 0.25 mol %^33^ to 5 mol %, the dihydroxylation proceeded rapidly to diastereoselectively afford the α‐diol Boc‐**(HO)_2_‐ProM1**‐O*t*Bu in 62 % yield (besides 11 % of the β‐isomer; see the Supporting Information). This compound was then smoothly converted into either Fmoc‐**(MeO)_2_‐ProM1**‐OH or Fmoc‐**(EtO)_2_‐ProM1**‐OH by double O‐alkylation (NaH, alkyl iodide, DMF) and subsequent protecting group adjustment under the standard conditions. Finally, to avoid free hydroxy groups during SPPS, the diol unit of Boc‐**(HO)_2_‐ProM1**‐O*t*Bu was protected as an acetonide to afford Fmoc‐**Me_2_CO_2_‐ProM1**‐OH after protecting‐group exchange.

**Scheme 2:**
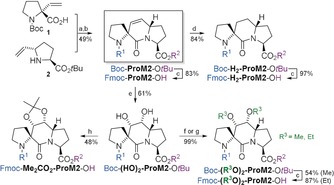
Synthesis of **ProM2**‐derived building blocks. Reagents and conditions: a) HATU, (*i*Pr)_2_NEt, MeCN, reflux, 22 h; b) Grubbs II catalyst, CuI, Et_2_O, reflux, 6 h; c) TFA; then FmocCl, NaHCO_3_, H_2_O/THF, 25 °C, 22 h; d) H_2_, Pd/C, MeOH, 25 °C, 10 min; e) RuCl_3_⋅3 H_2_O, CeCl_3_, NaIO_4_, EtOAc/MeCN/H_2_O, 0 °C, 10 min; f) NaH, MeI, DMF, 0 °C to 25 °C, 2 h; g) NaH, EtI, DMF, 0 °C to 25 °C, 15 h; h) TFA, H_2_O, TIPS; then FmocCl, NaHCO_3_, H_2_O/THF, 25 °C, 2 h; then 2,2‐dimethoxypropane, *p*‐TsOH, acetone/CH_2_Cl_2_, 25 °C, 6 h. HATU=1‐[bis(dimethylamino)methylene]‐1*H*‐1,2,3‐triazolo[4,5‐*b*]pyridinium‐3‐oxide hexafluorophosphate.

The synthesis of **ProM2**‐derived building blocks is shown in Scheme 2. Boc‐**ProM2**‐O*t*Bu was prepared from the known building blocks **1** and **2** in an improved two‐step protocol through HATU‐mediated peptide coupling (in MeCN as a superior solvent) and subsequent Grubbs II‐catalyzed ring‐closing metathesis. The syntheses of the (crystalline) dihydro derivative Boc‐**H_2_‐ProM2**‐O*t*Bu (see Figure 3), the dihydroxylated product Boc‐**(HO)_2_‐ProM2**‐O*t*Bu, and protected derivatives thereof were accomplished under the conditions elaborated before. Stereochemical assignments were again supported by NMR NOE experiments.

It is noteworthy that epoxidation, hydroboration, and dihydroxylation of both Boc‐**ProM1**‐O*t*Bu and Boc‐**ProM2**‐O*t*Bu occurred predominantly from the bottom (α) face of the C=C double bond, probably because of steric effects.

### Synthesis of Collagen Model Peptides

Using common SPPS protocols (for details see the Supporting Information), the Fmoc‐protected **ProM1** and **ProM2** derivatives were successfully incorporated into the [XY] position of the CMP sequence Ac‐(PPG)_5_‐PRG‐PPG‐**[XY]**G‐(PPG)_3_‐NH_2_ (Table 1). All peptides were purified by HPLC and characterized by ESI‐MS (see the Supporting Information for details). The acetal and TBS protecting groups were lost under the acidic conditions (TFA) used to cleave off the peptide from the resin. The MS data of the **ep‐ProM1**‐derived model peptide indicated that the epoxide function had not survived the cleavage conditions. Thus, the obtained undefined mixture of peptides (containing a *trans*‐diol unit) was excluded from further studies. In contrast, the ProM units of all other CMPs remained unaffected according to the analytical data.

**Table 1:** Synthesized CMPs and their experimental transition temperatures.

Entry	Collagen model peptide sequence^[a]^	*T*_m_ [°C]
1	Ac‐(PPG)_5_‐PRG‐PPG‐**ProPro**G‐(PPG)_3_‐NH_2_	46.5
2	Ac‐(PPG)_5_‐PRG‐PPG‐**ProHyp**G‐(PPG)_3_‐NH_2_^[b]^	49.8
3	Ac‐(PPG)_5_‐PRG‐PPG‐**ProM1**G‐(PPG)_3_‐NH_2_	39.3
4	Ac‐(PPG)_5_‐PRG‐PPG‐**H_2_‐ProM1**G‐(PPG)_3_‐NH_2_	39.9
5	Ac‐(PPG)_5_‐PRG‐PPG‐**HO‐ProM1**G‐(PPG)_3_‐NH_2_	43.0
6	Ac‐(PPG)_5_‐PRG‐PPG‐(**HO)_2_‐ProM1**G‐(PPG)_3_‐NH_2_	44.1
7	Ac‐(PPG)_5_‐PRG‐PPG‐(**MeO)_2_‐ProM1**G‐(PPG)_3_‐NH_2_	43.6
8	Ac‐(PPG)_5_‐PRG‐PPG‐(**EtO)_2_‐ProM1**G‐(PPG)_3_‐NH_2_	44.5
9	Ac‐(PPG)_5_‐PRG‐PPG‐**ProM2**G‐(PPG)_3_‐NH_2_	43.8
10	Ac‐(PPG)_5_‐PRG‐PPG‐**H_2_‐ProM2**G‐(PPG)_3_‐NH_2_	45.1
11	Ac‐(PPG)_5_‐PRG‐PPG‐(**HO)_2_‐ProM2**G‐(PPG)_3_‐NH_2_	37.9
12	Ac‐(PPG)_5_‐PRG‐PPG‐(**MeO)_2_‐ProM2**G‐(PPG)_3_‐NH_2_	40.2
13	Ac‐(PPG)_5_‐PRG‐PPG‐(**EtO)_2_‐ProM2**G‐(PPG)_3_‐NH_2_	38.9
14	Ac‐(PPG)_3_‐**ProM1**G‐PPG‐PRG‐(PPG)_5_‐NH_2_	40.5
15	Ac‐(PPG)_3_‐**H_2_‐ProM2**G‐PPG‐PRG‐(PPG)_5_‐NH_2_	44.6

[a] See the Supporting Information for biotinylated or R‐free peptides. [b] Hyp=(2*S*,4*R*)‐hydroxyproline.

### Determination of Triple‐Helix Stability

**Figure 4:**
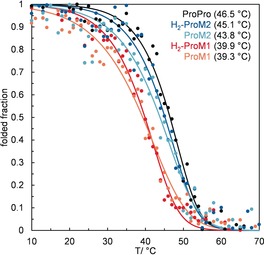
Thermal triple‐helix denaturation monitored by the decrease in CD ellipticity at 225 nm for ProM‐modified peptides Ac‐(PPG)_5_‐PRG‐PPG‐[XY]G‐(PPG)_3_‐NH_2_ and corresponding *T*_m_ values (heating rate: 12 °C h^−1^; see the color code for incorporated ProM at [XY] and see the Supporting Information for experimental conditions).

For all prepared model peptides, CD spectra were recorded in phosphate‐buffered saline (after incubation for 24 h at 4 °C). Characteristic ellipticity curves and a maximum at *λ*=225 nm (see Figure S7) indicated a PPII‐type helix conformation within a collagen triple‐helical architecture in all cases.^34^ The peptides were then subjected to thermal denaturation studies (see the Supporting Information for details) monitoring the decrease of ellipticity. The curves were fitted to assess triple‐helical stability by the transition temperature (*T*_m_, ±1 °C error) in a standardized fashion (see Table 1 and the Supporting Information). Selected denaturation curves are depicted in Figure 4.

### Computer‐Assisted Conformational Analysis

**Figure 5:**
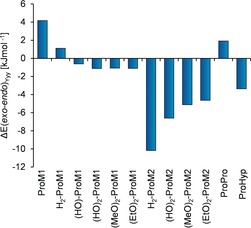
Relative DFT energies for the ring flip at the Yyy position (see the Supporting Information for details). Negative values indicate *exo* preference, positive ones *endo* preference.

As a precondition for an in‐depth interpretation of the relative triple‐helical stabilities of the CMPs, we computationally assessed the relevant structural space of all ProM units using conformational searches and subsequent DFT geometry optimizations (B3LYP‐D3/6‐31G*, H_2_O polarized continuum model; for details see the Supporting Information). In a minimalistic approach, only the N‐acetylated dipeptide methyl esters (Ac‐[ProM]‐OMe) were evaluated to save computational costs. In addition to this, only collagen‐relevant conformers were selected and then further optimized. For each ProM a set of up to four collagen‐relevant conformers was obtained (all‐*trans* amides, *ψ*_Yyy_ close to 180°, *endo*/*endo*, *endo*/*exo*, *exo*/*endo*, or *exo*/*exo* puckered rings). Generally, increased thermal stability was anticipated if this set of DFT‐optimized structures contained a low‐energy conformer that fulfills the structural requirements of the collagen triple helix (preorganization, see above). In (PPG)_10_, ϕ_Xxx_=−75°, ψ_Xxx_=164°, ϕ_Yyy_=−60° and ψ_Yyy_=152° were found as mean values around which the main‐chain torsional angles fluctuate.^21^ Consideration of the main‐chain torsional angles of the DFT‐optimized, lowest‐energy structure of Ac‐**ProM1**‐OMe (ϕ_Xxx_=−67°, ψ_Xxx_=170°, ϕ_Yyy_=−74°, ψ_Yyy_=160°) underlined the preorganization of the main chain. In Table S3, structural parameters of all calculated structures are given, and the deviation from (PPG)_10_ average values is depicted in Figure S10. Because of the overall small deviation (<15°) for each torsion angle, the concept of preorganization in ProMs could be confirmed. As an important aspect, the intrinsic *endo* or *exo* ring‐flip preferences were analyzed for all ProMs using the set of DFT‐calculated collagen‐relevant conformers. As the ring pucker at the Yyy position was found to be crucial for triple‐helix stability,^1^ energy differences only for the Yyy position were determined in the following (see Figure 5).

### Analysis of Structure–Stability Relationships

**Figure 6:**
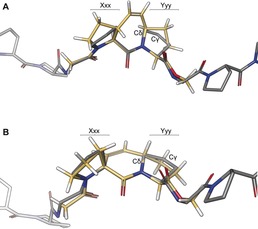
Main‐chain atom alignment of *exo*/*endo*‐Ac‐**ProM1**‐OMe (A, yellow) or *endo*/*exo*‐Ac‐**ProM2**‐OMe (B, yellow) with (PPG)_10_ (gray, PDB: 1k6f).^21^

In Figure 4, thermal denaturation curves of ProM‐modified peptides are presented in comparison to Ac‐(PPG)_5_‐PRG‐(PPG)_5_‐NH_2_ as a reference peptide. Unexpectedly, the incorporation of **H_2_‐ProM1** or **ProM1** led to significant destabilization of the triple helix (Δ*T*_m_=−6 °C). Regarding the reference peptide, the stability was slightly decreased for the **ProM2**‐CMP or maintained in the case of the **H_2_‐ProM2**‐CMP. To address the question why the **ProM1** derivatives significantly impaired triple‐helical stability while the **ProM2** derivatives did not, we analyzed the conformational preferences of the two systems with regard to five‐ring puckering (compare Figures 1 and 5). Remarkably, Ac‐**ProM1**‐OMe and Ac‐**H_2_‐ProM1**‐OMe showed an *endo* preference whereas Ac‐**H_2_‐ProM2**‐OMe exhibited a strong preference for the *exo* state. In the case of Ac‐**ProM2**‐OMe, no conformer with an *endo* ring flip at Yyy was found in the initial conformational search (30 kJ mol^−1^ cutoff), demonstrating the general *exo* propensity of **ProM2** derivatives. Thus, considering the DFT‐optimized structures, the different ring‐flip preferences can be explained by the configuration at Cδ in the proline ring at the Yyy position. Cδ connects the Cγ atom with the rigid C_2_ bridge. A pseudo‐equatorial orientation of the rigid ethylene linker relative to the Yyy ring is sterically favored and thus controls the Cγ ring flip depending on the configuration at Cδ (*endo* for Ac‐**ProM1**‐OMe, *exo* for Ac‐**ProM2**‐OMe; see Figure 6). Comparable steric effects have already been reported for 4‐methylproline.^25^, ^35^

Alignment of the calculated lowest‐energy structures of Ac‐**ProM1**‐OMe and Ac‐**ProM2**‐OMe with the collagen model peptide (PPG)_10_ again confirmed the preorganization of the main chain,^21^ but did not explain the different stabilities of the **ProM1**‐ and the **ProM2**‐CMP. Because of their desired *endo*/*exo* flip the proline rings of Ac‐**ProM2**‐OMe were positioned in space that is also occupied by proline rings in a collagen environment (see Figure 6). In fact, this *endo*/*exo* flip could be found in a crystal structure of the **ProM2**‐CMP facilitating a close packing of proline residues (see below). However, the *exo*/*endo* preference observed for Ac‐**ProM1**‐OMe probably led to significant distortion in the corresponding CMP. This distortion could perturb the interstrand packing (and the van der Waals interactions) of unpolar proline residues at the triple helix periphery, resulting in reduced triple‐helix stability.^36^

Hydroxy functionalization of proline in general can change conformational properties and can lead to triple‐helix stabilization (see above). We therefore used the opportunity to test the influence of linker hydroxylation instead of ring hydroxylation. CD experiments revealed that α‐monohydroxylation or α‐dihydroxylation of the **ProM1**‐CMP caused significant triple‐helix stabilization with regard to the non‐hydroxylated **ProM1**‐CMP (see Figure S8). The *T*_m_ value of the **(HO)_2_‐ProM1**‐modified peptide is close to the one of the reference peptide (XY=PP). To probe whether hydrophilic interactions^18^, ^37^ between the hydroxylated linker and water were responsible for this stabilizing effect, the **(MeO)_2_‐** and **(EtO)_2_‐ProM1** peptides were compared with the **(HO)_2_‐ProM1**‐CMP. The transition temperature remained unchanged at 44 °C. Therefore, the water interaction hypothesis^18^, ^37^ had to be discarded as alkylation of the hydroxy groups would have been expected to weaken a possible hydrogen‐bond interaction. In the literature, an analogous observation was made for (4*R*)‐hydroxyproline and (4*R*)‐methoxyproline.^38^ It was concluded that the stabilization in such substitutions rather originates from a stereoelectronic *gauche* effect, which preorganizes the proline ring and requires stereochemically defined substitution with electronegative groups (‐OH, ‐OMe, ‐F).^1^, ^38^ In the case of Ac‐**ProM1**‐OMe, calculations underlined that the relative position of proline rings to each other was altered upon oxy functionalization of the C_2_ bridge (see the torsion angles in Table S3 and Figure S10). Furthermore, the ring pucker preference was inverted from *endo* to *exo* at the Yyy position (see Figure 5). Possibly, oxy functionalization of the C_2_ bridge affected the dipeptide conformation and overall triple‐helical stability via a *gauche* effect on the ring‐linker‐ring torsion.

**Figure 7:**
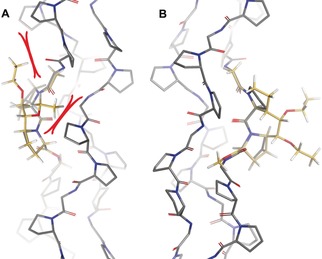
Main‐chain alignment of A) Ac‐**(EtO)_2_‐ProM2**‐OMe (steric clashes in red) and B) Ac‐**(EtO)_2_‐ProM1**‐OMe with reference peptide (PPG)_10_.^21^

For the **ProM2**‐based CMPs, different results were obtained when the interconnecting C_2_ linker was HO‐, MeO‐, or EtO‐functionalized. The **(HO)_2_‐ProM2**‐modified peptide showed significant (Δ*T*_m_=−7 °C) destabilization with regard to the non‐functionalized system. This was also observed to a slightly lower extent (Δ*T*_m_=−5 or −6 °C) for the **(MeO)_2_‐ProM2**‐ and the **(EtO)_2_‐ProM2**‐CMP. In a previous work, an intrastrand H‐bond to the main chain was reported to destabilize the triple helix.^39^, ^40^ DFT calculations in this work demonstrated that α‐dihydroxylation of Ac‐**ProM2**‐OMe introduced at least one intramolecular H‐bond (see Figure S11). Hence, the diproline structure was distorted and notably deviated from the required values for the main chain of a collagen triple helix (see Figures S10), resulting in reduced stability. Although alkylation of the HO groups in the **(HO)_2_‐ProM2**‐CMP was expected to switch off intramolecular H‐bonding, the corresponding peptides were still destabilized. As steric repulsion within the triple helix could explain this result,^39^ the DFT‐optimized structures of Ac‐**(EtO)_2_‐ProM1**‐OMe and Ac‐**(EtO)_2_‐ProM2**‐OMe were aligned with (PPG)_10_ to describe the steric situation in the corresponding collagen model peptides. The alignments (Figure 7) showed that the ethoxy groups in Ac‐**(EtO)_2_‐ProM2**‐OMe indeed clash with a preceding carbonyl group of the same strand and a proline ring of a neighboring strand. However, the ethoxy groups in Ac‐**(EtO)_2_‐ProM1**‐OMe were found to be radially oriented, pointing away from the triple helix axis. In addition to existing methods for triple‐helix functionalization,^41^, ^42^, ^43^ the **(HO)_2_‐ProM1** scaffold might be used for double functionalization by O‐alkylation. In conclusion, the steric demand of the linker group was determined to be crucial for the thermal stability of ProM‐modified CMPs.

A still remaining question was why most of the ProM‐modified collagen peptides (except for **H_2_‐ProM2**‐CMP) showed a significantly lower *T*_m_ value than the reference peptide (XY=PP) despite their entropic advantage resulting from preorganization in a PPII‐helix‐type conformation (as confirmed by DFT calculations and structural alignments). The observed trend remained valid upon substituting a different pair of prolines in the model peptide sequence by a ProM unit or upon replacing an arginine by a proline (see Figure S9). Hence, a surprising destabilizing effect seemed to affect all ProM‐modified peptides. In diproline, ring movement can rather occur individually when compared to ProMs in which the two pyrrolidine rings are fused to a central ring. Possibly, the two proline rings in ProMs might fail to individually adapt to thermal fluctuations^44^, ^45^ of the main chain without producing strain in the tricyclic system. Adaptability was therefore proposed to be a relevant factor for triple‐helix stability and was assumed to be improvable for the thus far tested ProMs. Generally, the importance of adaptability beside preorganization was highlighted in previous work on linker optimization in fragment‐based drug design.^46^

In the previous sections, four key aspects (main‐chain and ring‐flip preorganization, adaptability, steric effects) were identified to be crucial for the triple‐helix stability of ProM‐modified CMPs. DFT calculations on dipeptide systems and crystal structure alignments were performed to investigate ProMs regarding these key aspects. Guided by literature and DFT‐results, the ProMs were evaluated in terms of triple‐helix suitability. Significant differences in triple‐helix suitability were observed and now allowed us to rank the ProMs for each above‐mentioned key aspect (for details, see the Supporting Information). To consider all rankings for all key aspects simultaneously, a scoring system was introduced and optimized. As a result, the theoretical findings could be correlated with experimental data (see Table 2 and the Supporting Information). More investigations will be necessary to enhance the predictive applicability of this simplified, semi‐empirical model. However, the model sums up and explains the structure–stability relationships of ProM‐modified CMPs described in this work.

**Table 2:** Ranking results based on DFT calculations and correlation with *T*_m_ values of the corresponding CMPs.

Ac‐[ProM]‐OMe	Main chain^[a]^	Ring flip^[b]^	Adaptability^[c]^	Sterics^[d]^	S^[e]^	*T*_m_ [°C]
**ProHyp**	o	+	+	o	++	49.8
**ProPro**	+	–	+	o	+	46.5
**H_2_‐ProM2**	o	+	o	o	+	45.1
**(EtO)_2_‐ProM1**	+	o	o	o	+	44.5
**(HO)_2_‐ProM1**	+	o	o	o	+	44.1
**ProM2**	o	+	o	o	+	43.8
**(MeO)_2_‐ProM1**	+	o	o	o	+	43.6
**(HO)‐ProM1**	+	o	o	o	+	43.0
**(MeO)_2_‐ProM2**	o	+	o	–	–	40.2
**H_2_‐ProM1**	o	–	o	o	–	39.9
**ProM1**	o	–	o	o	–	39.3
**(EtO)_2_‐ProM2**	o	+	o	–	–	38.9
**(HO)_2_‐ProM2**	–	+	o	o	–	37.9

[a] Preorganization of the torsional angles ϕ_Xxx_, ψ_Xxx_, and ϕ_Yyy_. [b] The *exo* ring flip preference in Yyy position. [c] Adaptability to small torsion‐angle fluctuations. [d] Steric toleration in the collagen triple helix. [e] Sum of the results from the four ranking categories. “+” above average; “o” average; “–” below average in terms of triple helix suitability. See the main text and the Supporting Information for more details.

### Crystal‐Structural Analysis of the ProM2‐CMP

**Figure 8:**
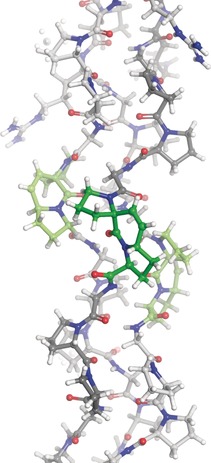
Section of the X‐ray crystal structure of Ac‐(PPG)_5_‐PRG‐PPG‐[**ProM2**]G‐(PPG)_3_‐NH_2_ centered on the **ProM2** substitution sites (PDB: 6SYJ).

In addition to this model, more structural insights could be obtained. To our delight, the Ac‐(PPG)_5_‐PRG‐PPG‐[**ProM2**]G‐(PPG)_3_‐NH_2_ peptide (**ProM2**‐CMP) crystallized, giving access to an X‐ray structure of high resolution (PDB: 6SYJ; see Figure 8 and Tables 3 and S6 for parameters). This is the first crystal structure of an intrastrand‐linked collagen model peptide in which two subsequent proline residues are connected.

**Table 3:** Comparison of triple helix parameters from crystal structures.

Torsion angle [°]	**ProM2**‐CMP^[a]^	(PPG)_10_^[b]^
ϕ_Xxx_	−73.8±5.0	−74.5±2.9
ψ_Xxx_	163.6±4.7	164.3±4.1
ω_Xxx_	174.2±3.9	176.0±2.5
ϕ_Yyy_	−59.9±3.5	−60.1±3.6
ψ_Yyy_	151.2±3.8	152.4±2.6
ω_Yyy_	172.8±4.1	175.4±3.4
ϕ_Gly_	−68.4±3.9	−71.7±3.7
ψ_Gly_	173.8±4.2	175.9±3.1
ω_Gly_	178.8±2.9	179.7±2.0

[a] Peptide sequence: Ac‐(PPG)_5_‐PRG‐PPG‐[**ProM2**]G‐(PPG)_3_‐NH_2_; torsion angles were averaged for the central nine amino acid triplets. [b] See Ref. 21.

Regarding the overall structure, the three peptide strands adopted the typical triple‐helical conformation, and no distortion could be detected, which was underlined by comparison of key parameters (see Table 3) and alignment with (PPG)_10_ (see Figure S15).^21^ Considering the modified positions, **ProM2** units were assembled in a PPII‐type helical shape (like all the other Pro rings) and were well‐embedded into the overall structure. In combination with the fact that all **ProM2** units adopted an *endo*,*exo* ring puckering (as predicted by DFT calculations), a close packing of Pro rings within the triple helix was observed. The crystal structure not only confirmed the synthetic success of introducing covalent linkages in collagen models, but also demonstrated that the triple helical structure was maintained upon this manipulation.

### Binding of ProM‐Modified CMPs to HSP47

To evaluate structural and functional consequences of ProM modifications in collagen models, the interaction with HSP47 was investigated using biolayer interferometry (BLI). Biotinylated collagen model peptides containing the PRG‐binding motif were prepared (see the Supporting Information) and immobilized on streptavidin biosensors. HSP47 binding kinetics were then analyzed to give *K*_D_ values for these peptides (see Tables 4 and S7). First of all, HSP47 was detected to bind to all triple‐helical peptides, which is consistent with previous observations.^47^ Although the **(HO)_2_‐ProM1** peptide showed a weaker interaction, the *K*_D_ values for all other ProM‐modified peptides were very similar to that for the reference peptide; they even hinted at a tighter binding. As a result, an HSP47‐compatible position for an intrastrand linkage could be identified in this work. These modified peptides were recognized by HSP47 and therefore all formed a regular triple helix.

**Table 4:** HSP47 dissociation constants of model peptides.

	Peptide,^[a]^ XY=	*K*_D_ [nm]^[b]^	
	**PP**	370.3	
	**ProM2**	361.7	
	**H_2_‐ProM2**	353.0	
	**H_2_‐ProM1**	268.9	
	**(HO)_2_‐ProM1**	502.3

[a] Biotin‐Ebes‐(PPG)_5_‐PRG‐PPG‐[XY]G‐(PPG)_3_‐NH_2_ with XY indicating the (modified) diproline unit. [b] Determined by fit of biolayer interferometry parameters *k*_on_, *k*_off_ and using *K*_D_=*k*_off_/*k*_on_ (see the Supporting Information). Ebes‐OH=*N*‐(8‐amino‐3,6‐dioxaoctyl)succinamic acid.

## Conclusion

In summary, novel ProM derivatives have been synthesized that differ in the linker motif connecting two consecutive proline rings. The stereochemistry of the linker oxy functionalization was dominated by substrate control. By using Fmoc/*t*Bu‐based SPPS, the ProMs could be incorporated into collagen model peptides, which folded into triple helices as confirmed by CD. As a result, a method for intrastrand diproline linkage could be developed, which complements literature‐known options for structural modifications in collagen. Previous studies were mainly focused on 4‐substituted proline derivatives. Relations between structural modification and effects on collagen triple‐helix stability have been elucidated. Theoretical and experimental results were successfully correlated to explain such relations in the context of ProM‐modified CMPs by considering the interplay of four key aspects (main‐chain and ring‐flip preorganization, adaptability, and steric effects). Although the adaptability of ProM‐modified CMPs remains improvable, the **H_2_‐ProM2**‐modified peptide was as stable as the reference system. An X‐ray crystal structure and an HSP47 binding assay confirmed the structural and functional integrity of ProM‐modified CMPs. Our strategy of using intrastrand diproline linkages might be attractive for future studies on proteolytic stability, folding kinetics, or CMP functionalization. Generally, our structure–stability model can support the design of proline‐rich collagen‐based biomaterials.^48^

## Conflict of interest

The authors declare no conflict of interest.

## Supporting information

As a service to our authors and readers, this journal provides supporting information supplied by the authors. Such materials are peer reviewed and may be re‐organized for online delivery, but are not copy‐edited or typeset. Technical support issues arising from supporting information (other than missing files) should be addressed to the authors.

Supplementary
