# Supplementary material for: Triple‐Helix‐Stabilizing Effects in Collagen Model Peptides Containing PPII‐Helix‐Preorganized Diproline Modules
Source: Angew Chem Int Ed Engl. 2020 Feb 3;59(14):5747–55. doi: 10.1002/anie.201914101 (PMC7154665; doi:10.1002/anie.201914101)
Supplement: Supplementary file 1 — Supplementary [file ANIE-59-5747-s001.pdf]

Angewandte Chemie
Eine Zeitschrift der Gesellschaft Deutscher Chemiker

Supporting Information

Triple-Helix-Stabilizing Effects in Collagen Model Peptides Containing PPII-Helix-Preorganized Diproline Modules

Andreas Maaßen, Jan M. Gebauer, Elena Theres Abraham, Isabelle Grimm, Jörg-Martin Neudörfl, Ronald Kühne, Ines Neundorf, Ulrich Baumann, and Hans-Günther Schmalz**

anie_201914101_sm_miscellaneous_information.pdf

Author Contributions

A.M. Data curation: Lead; Formal analysis: Lead; Investigation: Lead; Methodology: Lead; Project administration: Lead; Writing—Original Draft: Lead.

Table of Contents

General Conditions 3
Organic Syntheses 4
Stereochemical Assignments 32
Solid Phase Peptide Syntheses 34
CD spectra and Thermal Denaturation Experiments 35
Computational Methods and Primary Results 39
Ranking Procedure 42
X-ray Crystal Structure Parameters of ProM-derivatives 43
X-ray Crystal Structure Parameters of ProM2-CMP and Structural Alignment 47
HSP47 binding assay and K_D-determination 49
NMR spectra 51
ESI-MS spectra of peptides 84
Optimized Structures and Cartesian Coordinates 95
References 119
Author Contributions 120

General Conditions

Conditions and Reagents - All organic syntheses with air- or water-sensitive reagents were performed under an atmosphere of argon (*Linde*, Argon 4.6). Glassware was flame-dried under oil pump vacuum prior to argon purging. The addition of solids was performed under Argon counter flow whereas liquids were added *via* oven-dried and argon-flushed syringes, needles or cannula. For inert reaction conditions, dry THF was obtained by reflux-distillation over sodium/benzophenone and dry dichloromethane by distillation over calcium hydride. Dry acetonitrile, acetone and DMF were purchased in extra dry quality (*Acros*) or HPLC grade was employed in the case of methanol. All reagents were used without further purification.
Chromatography - Analytical thin layer chromatography was carried out on silica gel plates (*Merck*, TLC Silica Gel 60 F254) using UV light, $KMnO_4$ or $Ce(SO_4)_2$ stain solution for detection of spots. Column chromatography or chromatographic filtrations were performed with standard silica gel (*Acros*, 60 A, 0.035 – 0.070 mm) or ultrapure silica gel (*Acros*, 60 A, 0.040 – 0.060 mm) using distilled solvents (EtOAc, cHex, CH_2Cl_2, EtOH, MeOH).
GC-MS - Gas chromatography was run on an *Agilent* HP6890N system equipped with 5937N mass detector and Optima 1 MS (30 m x 0.25 mm) column (*Macherey-Nagel*). The heating process was the following: 2 min 50 °C, rate: 25 °C/min, 5 min 300 °C, 5 min 320 °C.
NMR - All ^1H and ^{13}C NMR spectra were recorded at RT on *Bruker* instruments (Avance II operating at 300 MHz, Avance operating at 400 MHz, Avance III operating at 500 MHz, Avance II+ operating at 600 MHz). In proton spectra the chemical shifts were referenced to tetramethylsilane (0.00 ppm) or to the solvent residual signal (77.1 ppm, $CDCl_3$) in carbon spectra, respectively. Coupling constants are given in Hertz (Hz) and multiplicities as follows: s = singlet, d = doublet, t = triplet, q = quartet, m = multiplet, br. = broad. In ^{13}C spectra multiplicities were identified by attached proton tests (s = quartenary, d = tertiary, t = secondary, q = primary carbon atom). The assignment of signals was either confirmed by comparison to data from the literature or by analysis of 2D experiments (COSY, HMQC, HMBC, NOESY).
IR - Infrared spectra were recorded on a Spectrum Two UATR FT-IR spectrometer (*Perkin Elmer*) at RT and relative intensities were reported as strong (s), medium (m) or weak (w).
MP - Melting points were determined on an apotec (*Wepa*) instrument.
HR-MS - For high-resolution mass spectrometry of small molecules, a LTQ Orbitrap XL mass spectrometer (*Thermo Scientific*) was used in positive ion mode. Ionization of samples was achieved by electrospray ionization (ESI).
$[\alpha]^{20}_D$ - Optical rotations were determined on a MCP 200 polarimeter (*Anton Paar*) at 20 °C using a cuvette with a path length of 10 cm. Sample concentrations are given in g/100 ml solvent.
.

Organic Syntheses

Construction of the ProM1- and ProM2-scaffold

Synthesis of ProM1-precursor peptide 5

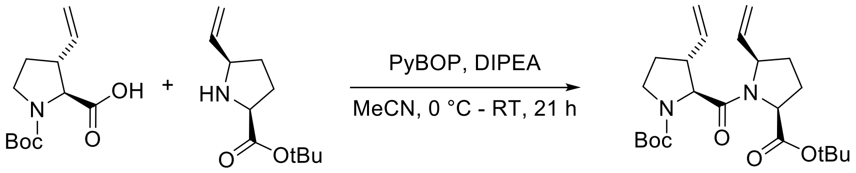

3 **4** **5**

The enantiomerically pure acid **3** (1.11 g, 4.61 mmol, 1.0 eq) was dissolved together with the *cis*-amine **4** (1.00 g, 5.07 mmol, 1.1 eq) in 23 ml dry acetonitrile at RT under argon. Diisopropylethylamine (2.55 ml, 14.99 mmol, 3.3 eq) was slowly added at 0 °C. Within 10 min, a solution of PyBOP (3.11 g, 5.97 mmol, 1.3 eq) in 23 ml dry acetonitrile cooled to 0 °C was added and the reaction mixture was stirred for 1 h at 0 °C and overnight at RT. After concentration of the reddish-brown solution, 60 ml MTBE and 60 ml water were added and the phases separated, the aqueous phase being extracted with MTBE (3 x 60 ml). The combined organic phases were washed with 30 ml sat. NaCl solution, dried over $MgSO_4$ and concentrated at the rotary evaporator. For preliminary purification, the raw product was filtered twice over a thin silica gel layer (5 cm, cHex/EtOAc = 1:1) and stirred for 2 h over activated carbon (in 30 ml EtOAc). Column chromatography on silica gel (cHex/EtOAc = 3:1 to 1:1) resulted in a colorless oil.

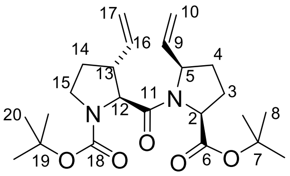

Yield 1.74 g, 4.15 mmol, 90% (Lit[1]: 81%).

MW 420.55 g mol^{-1} ($C_{23}H_{36}N_2O_5$).

R_f 0.58 (cHex/EtOAc = 1:1).

$[\alpha]^{20}_{\lambda}$ (c = 0.24, $CHCl_3$): -23.5° (365 nm), -12.8° (436 nm), -6.8° (546 nm), -7.0° (579 nm), -8.7° (589 nm).

^{1}H-NMR (300 MHz, $CDCl_3$, mixture of rotamers) δ [ppm] = 6.02 – 5.89 (m, 1.0H, H-9), 5.82 – 5.68 (m, 1.0H, H-16), 5.43 (d, *J* = 17.0 Hz, 1.0H, H-10), 5.15 (t, *J* = 9.4 Hz, 1.0H, H-10), 5.05 – 5.00 (m, 2.0H, H-17), 4.91 (t, *J* = 6.4 Hz, 0.6H, H-2), 4.58 (t, *J* = 6.4 Hz, 0.4H, H-2), 4.39 (t, *J* = 7.6 Hz, 1.0H, H-5), 4.28 (s, 0.6H, H-12), 4.19 (s, 0.4H, H-12), 3.70 (t, *J* = 9.9 Hz, 0.4H, H-15), 3.60 – 3.53 (m, 0.6H, H-15), 3.46 – 3.36 (m, 1.0H, H-15), 2.92 – 2.80 (m, 1.0H, H-13), 2.47 – 2.33 (m, 1.0H, H-14), 2.25 – 2.08 (m, 2.0H, H-3, H-4), 2.00 – 1.77 (m, 2.0H, H-3, H-4), 1.75 – 1.67 (m, 1.0H, H-14), 1.46, 1.42, 1.41 (3 x s, 18.0H, H-8, H-20).

^{13}C-NMR (75 MHz, $CDCl_3$, mixture of rotamers) δ [ppm] = 172.3 (s, C11), 172.0 (s, C11), 171.4 (s, C6), 171.2 (s, C6), 154.6 (s, C18), 139.2 (d, C9/C16), 138.8 (d, C9/16), 116.9 (t, C10), 116.5 (t, C10), 114.8 (t, C17), 114.8 (t, C17), 81.3 (s, C7/C19), 81.0 (s, C7/C19), 79.7 (s, C7/C19), 79.5 (s, C7/C19), 61.9 (d, C12), 61.7 (d, C12), 61.2 (d, C2), 61.1 (d, C2), 60.9 (d, C5), 47.1 (d, C13), 46.3 (d, C13), 46.1 (t, C15), 45.7 (t, C15), 33.0 (t, C3/C4), 32.7 (t, C3/C4), 29.8 (t, C14), 28.7 (q, C8, C20), 28.6 (q, C8, C20), 28.5 (t, C14), 28.1 (q, C8, C20), 27.1 (t, C3/C4).

IR $\tilde{\nu}$ [cm^{-1}] = 3081 (w), 2976 (w), 2931 (w), 2881 (w), 1737 (m), 1691 (m), 1656 (m), 1478 (w), 1391 (s), 1365 (s), 1321 (m), 1301 (m), 1255 (m), 1211 (m), 1154 (s), 1116 (m), 1068 (w), 1032 (w), 988 (w), 912 (m), 865 (w), 842 (w), 771 (w), 689 (w).

GC-MS (τ = 10.57 min, EI, 70 eV): m/z (%) = 420 (1) [M^+], 364 (1), 347 (4), 308 (2), 291 (2), 263 (7), 222 (6), 196 (10), 168 (5), 140 (100), 124 (4), 96 (85), 79 (5), 57 (41), 41 (49).

Analytical data was in agreement with the literature.[1]

Synthesis of ProM2-precursor peptide 6

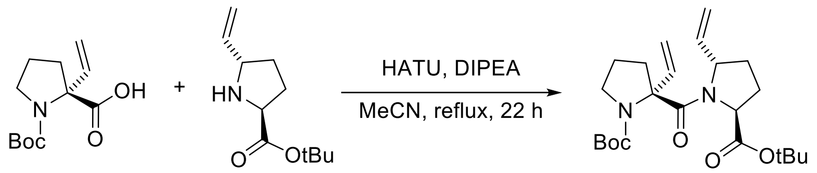

1 **2** **6**

Under argon, the enantiomerically pure acid **1** (1.33 g, 5.52 mmol, 1.00 eq) and HATU (2.26 g, 5.94 mmol, 1.08 eq) were dissolved in 55 ml dry acetonitrile. At RT DIPEA (1.79 ml, 10.53 mmol, 1.91 eq) was added and the reaction mixture stirred for 10 min at RT. The *trans*-amine **2** (1.24 g, 6.29 mmol, 1.14 eq) was added and the mixture was stirred for 22 h with moderate reflux (oil bath temp. = 85 °C). After being cooled to RT the reaction mixture was added to 100 ml of 10 wt% citric acid solution and extracted with MTBE (3 x 150 ml). The combined organic phases were washed with 50 ml saturated $NaHCO_3$ solution and 50 ml saturated NaCl solution. After drying over $MgSO_4$ the solvent was removed at the rotary evaporator. The reddish-brown raw product was pre-cleaned by twofold filtration over SiO_2 (400 ml cHex/EtOAc = 1:1, then solvent removal) and stirred for 1 h over activated carbon in 1:1 cHex/EtOAc, resulting in a yellowish oil. For final purification, column chromatographic purification was performed on SiO_2 (cHex/EtOAc = 3:1 to 3:2) to yield a colorless, viscous oil after vacuum drying.

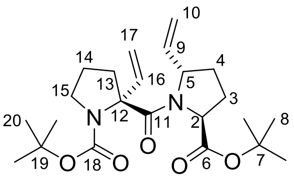

Yield 1.54 g, 3.65 mmol, 66% (LIt[2]: 72%).

MW 420.55 g mol^{-1} ($C_{23}H_{36}N_2O_5$).

R_f 0.59 (cHex/EtOAc = 1:1).

$[\alpha]^{20}_{\lambda}$ (c = 0.25, $CHCl_3$): -178.6° (365 nm), -113.7° (436 nm), -67.1° (546 nm), 59.5° (579 nm), -57.7° (589 nm).

^{1}H-NMR (500 MHz, $CDCl_3$, mixture of rotamers) δ [ppm] = 6.71 – 6.61 (m, 1.0H, H-16), 5.87 – 5.69 (m, 1.0H, H-9), 5.33 – 5.00 (m, 4.0H, H-17, H-10), 4.88 – 4.73 (m, 1.3H, H-2, H-5), 4.64 – 4.57 (m, 0.2H, H-5), 4.39 – 4.32 (m, 0.6H, H-2), 3.76 – 3.47 (m, 2.0H, H-15), 2.37 – 2.31 (m, 0.8H, H-13), 2.27 – 2.18 (m, 1.7H, H-13, H-4), 2.14 – 2.05 (m, 1.1H, H-3), 2.02 – 1.92 (m, 0.6H, H-4), 1.88 – 1.78 (m, 2.0H, H-14), 1.77 – 1.69 (m, 1.0H, H-3), 1.68 – 1.63 (m, 0.8H, H-4), 1.48 – 1.43 (3 x s, 18.0H, H-8, H-20).

^{13}C-NMR (75 MHz, $CDCl_3$, mixture of rotamers) δ [ppm] = 171.3 (s, C11), 170.7 (s, C6), 140.1 (d, C16), 138.6 (d, C9), 114.6 (t, C10), 112.2 (t, C17), 80.8 (s, C7/19), 70.7 (s, C12), 70.2 (s, C12), 62.3 (d, C2), 61.9 (d, C2), 60.2 (d, C5), 59.8 (d, C5), 48.5 (t, C15), 48.2 (t, C15), 36.9 (t, C13), 35.6 (t, C13), 31.4 (t, C4), 28.5 (q, C8, C20), 28.0 (q, C8, C20), 25.0 (t, C3), 21.8 (t, C14).

IR $\tilde{\nu}$ [cm^{-1}] = 3086 (w), 2976 (m), 2934 (w), 2877 (w), 1736 (m), 1694 (s), 1648 (m), 1630 (m), 1477 (w), 1454 (w), 1381 (s), 1366 (s), 1304 (w), 1252 (m), 1219 (m), 1152 (s), 1074 (w), 991 (w), 919 (w), 849 (w), 771 (w), 662 (w).

GC-MS (τ = 10.67 min, EI, 70 eV): m/z (%) = 420 (1) [M$^+$], 364 (1), 347 (2), 320 (1), 291 (6), 263 (2), 246 (2), 196 (6), 140 (100), 124 (6), 96 (70), 57 (42), 41 (33).

Analytical data was in agreement with the literature.[2]

Synthesis of Boc-ProM1-O*t*Bu

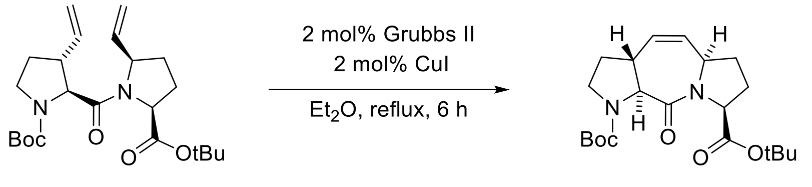

Under argon the dipeptide **5** (1.70 g, 4.04 mmol, 1.00 eq) was dissolved in 69 ml absolute diethyl ether. Grubbs II catalyst (34.3 mg, 40.4 µmol, 0.01 eq) and copper(I) iodide (7.7 mg, 40.4 µmol, 0.01 eq) were added to the solution, whereupon the reaction mixture was heated to reflux. After 1 h, the mixture was cooled to RT for 10 min and the addition of Grubbs II and copper(I) iodide was repeated with the quantities mentioned above. Then, the reaction mixture was heated for 5 h to reflux. The mixture was cooled to RT and stirred for 3 h over aminopropylated silica gel, which was washed with EtOAc after filtration. The filtrate was stirred overnight over activated carbon, which was then filtered off over a small amount of silica gel (washing with EtOAc). A colorless solid was obtained after column chromatography on silica gel (cHex/EtOAc = 1:1 to 1:2) and (optional) recrystallization from EtOAc/n-heptane (1:1).

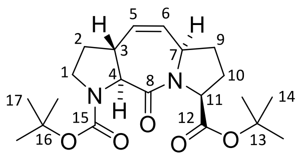

Yield 1.42 g, 3.61 mmol, 89% (Lit[1]: 91%).

MW 392.50 g mol^{-1} ($C_{21}H_{32}N_2O_5$).

R$_f$ 0.26 (cHex/EtOAc = 1:1).

Smp 134 - 136 °C.

[α]$^{20}_{\lambda}$ (c = 0.25, $CHCl_3$): -269.0° (546 nm), -236.7° (579 nm), -228.6° (589 nm).

^{1}H-NMR (300 MHz, $CDCl_3$, mixture of rotamers) δ [ppm] = 5.82 (d, *J* = 10.8 Hz, 1.0H, H-5), 5.57 (d, *J* = 10.8 Hz, 1.0H, H-6), 4.72 – 4.63 (m, 2.0H, H-7, H-11), 4.36 (d, *J* = 11.1 Hz, 0.7H, H-4), 4.30 (d, *J* = 11.1 Hz, 0.3H, H-4), 3.79 – 3.64 (m, 1.0H, H-1), 3.46 – 3.35 (m, 1.0H, H-1), 3.01 – 2.91 (m, 1.0H, H-3), 2.31 – 2.24 (m, 1.0H, H-9), 2.10 – 1.97 (m, 3.0H, H-2, H-10), 1.92 – 1.82 (m, 1.0H, H-9), 1.69 – 1.57 (m, 1.0H, H-2), 1.46, 1.42, 1.40 (3 x s, 18.0H, H-14, H-17).

^{13}C-NMR (75 MHz, $CDCl_3$, mixture of rotamers) δ [ppm] = 171.2 (s, C12), 169.4 (s, C8), 129.7 (d, C6), 128.8 (d, C5), 81.3 (s, C13/16), 79.8 (s, C13/16), 62.4 (d, C4), 62.2 (d, C4), 60.3 (d, C11), 57.4 (d, C7), 57.3 (d, C7), 47.0 (t, C1), 46.4 (t, C1), 42.1 (d, C3), 41.5 (d, C3), 33.2 (t, C9), 31.5 (t, C2), 31.0 (t, C2), 28.6 (q, C14, C17), 28.3 (q, C14, C17), 28.1 (q, C14, C17), 27.6 (t, C10), 27.4 (t, C10).

IR $\tilde{\nu}$[cm^{-1}] = 2974 (m), 2933 (w), 2902 (w), 2883 (w), 1736 (m), 1700 (s), 1681 (s), 1478 (w), 1405 (s), 1392 (s), 1366 (m), 1347 (w), 1326 (w), 1256 (w), 1222 (w), 1207 (w), 1161 (s), 1122 (m), 1074 (w), 1056 (w), 971 (w), 934 (w), 903 (w), 864 (w), 808 (w), 655 (w).

Metathesis conditions were improved in previous work.[3] Analytical data was in agreement with the literature.[1]

Synthesis of Boc-ProM2-O*t*Bu

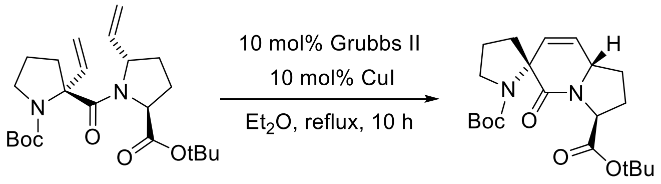

Under inert conditions, the dipeptide **6** (1.50 g, 3.57 mmol, 1.00 eq) was dissolved in 61 ml absolute diethyl ether and mixed with Grubbs II catalyst (30.3 mg, 35.7 µmol, 0.01 eq) and copper(I) iodide (6.8 mg, 35.7 µmol, 0.01 eq). The reaction mixture was then heated to reflux, with further Grubbs II catalyst and copper(I) iodide being added every one to two hours (oil bath removed for 10 min for each addition). In total, the reaction mixture was heated under reflux for 10 h at a gradual loading of 0.01 to 0.10 eq Grubbs II and 0.01 to 0.10 eq CuI. The reaction mixture was cooled to RT and stirred overnight over aminopropylated silica gel. After filtration (washing with EtOAc), the solution was stirred over activated carbon for 3 h, filtered over a small amount of silica gel and concentrated. A colorless solid was obtained after column chromatography on silica gel (cHex/EtOAc = 1:1) and (optional) recrystallization from EtOAc/n-heptane (1:1).

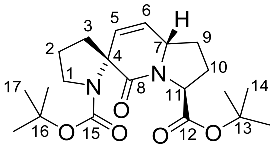

Yield 1.04 g, 2.65 mmol, 74% (Lit[2]: 68%).

MW 392.50 g mol^{-1} ($C_{21}H_{32}N_2O_5$).

R_f 0.29 (cHex/EtOAc = 1:1).

Smp 114 - 117 °C.

$[\alpha]^{20}_{\lambda}$ (c = 0.25, $CHCl_3$): -343.3° (436 nm), -193.3° (546 nm), -168.6° (579 nm), -163.3° (589 nm).

^{1}H-NMR (500 MHz, $CDCl_3$, mixture of rotamers) δ [ppm] = 5.94 - 5.89 (dd, *J* = 9.7, 2.0 Hz, 0.4H, H-5/6), 5.85 - 5.79 (m, 1.0H, H-5, H-6), 5.77 - 5.73 (dd, *J* = 9.7, 2.0 Hz, 0.6H, H-5/6), 4.59 (t, *J* = 8.5 Hz, 0.4H, H-11), 4.49 (t, *J* = 8.5 Hz, 0.6H, H-11), 4.25 - 4.18 (m, 1.0H, H-7), 3.78 (ddd, *J* = 10.5, 7.9, 4.3 Hz, 0.6H, H-1), 3.73 - 3.68 (m, 0.4H, H-1), 3.53 - 3.43 (m, 1.0H, H-1), 2.44 (dq, *J* = 12.9, 8.5 Hz, 1.0H, H-10), 2.29 - 2.22 (m, 1.0H, H-3), 2.18 - 2.08 (m, 1.0H, H-9), 2.08 - 1.98 (m, 1.0H, H-2), 1.95 - 1.79 (m, 3.0H, H-2, H-3, H-10), 1.78 - 1.70 (m, 0.4H, H-9), 1.60 - 1.52 (m, 0.7H, H-9), 1.47 - 1.43 (3 x s, 12.6H, H-14, H-17), 1.33 (s, 5.4H, H-14, H-17).

^{13}C-NMR (75 MHz, $CDCl_3$, mixture of rotamers) δ [ppm] = 171.6 (s, C12), 171.3 (s, C12), 168.1 (s, C8), 168.0 (s, C8), 154.3 (s, C15), 154.2 (s, C15), 134.1 (d, C6), 133.5 (d, C6), 122.6 (d, C5), 121.5 (d, C5), 81.4 (s, C13/16), 81.1 (s, C13/16), 79.7 (s, C13/16), 79.4 (s, C13/16), 64.4 (s, C4), 64.1 (s, C4), 58.7 (d, C7), 58.6 (d, C7), 57.9 (d, C11), 57.7 (d, C11), 48.3 (t, C1), 48.1 (t, C1), 39.5 (t, C3), 38.2 (t, C3), 31.4 (t, C9), 31.2 (t, C9), 28.6 (q, C14/17), 28.4 (q, C14/17), 28.3 (t, C10), 28.2 (t, C10), 28.1 (q, C14/17), 22.9 (t, C2), 22.6 (t, C2).

IR $\tilde{\nu}$ [cm^{-1}] = 2974 (m), 2928 (m), 2877 (w), 1736 (m), 1697 (s), 1661 (s), 1480 (w), 1434 (m), 1386 (s), 1365 (s), 1293 (m), 1257 (m), 1212 (m), 1151 (s), 1005 (w), 961 (w), 930 (w), 844 (w), 770 (w), 704 (w).

Metathesis conditions were improved in previous work.[3] Analytical data was in agreement with the literature.[2]

Derivatives of ProM1 and ProM2

Synthesis of Boc-H_2-ProM1-O*t*Bu

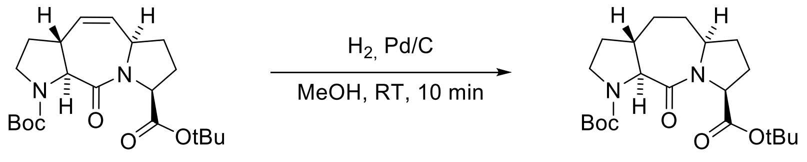

Boc-**ProM1**-O*t*Bu Boc-$\mathbf{H_2}$-**ProM1**-O*t*Bu

Boc-**ProM1**-O*t*Bu (55.0 mg, 140 µmol) was dissolved in 4.0 ml distilled MeOH in a Schlenk tube. After adding 2.8 mg palladium/carbon (10 wt% loading), hydrogen was passed through the reaction mixture for 10 min while stirring vigorously. The catalyst was separated by filtration and washed with 10 ml MeOH. The filtrate was concentrated at the rotary evaporator and the residue was purified by column chromatography on SiO_2 (cHex/EtOAc = 2:3) resulting in a colorless solid after removal of the solvent and drying *in vacuo*.

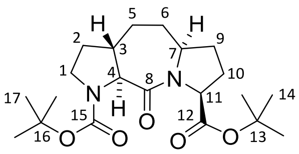

Yield 45 mg, 114 µmol, 81%.

MW 394.51 g mol^{-1} ($C_{21}H_{34}N_2O_5$).

R_f 0.34 (cHex/EtOAc = 1:2).

Smp 161 - 163 °C.

$[\alpha]^{20}_{\lambda}$ (c = 0.13, $CHCl_3$): -220.5° (546 nm), -194.6° (579 nm), -190.3° (589 nm).

^{1}H-NMR (500 MHz, $CDCl_3$, mixture of rotamers) δ [ppm] = 4.65 (dd, *J* = 8.4, 3.0 Hz, 0.7H, H-11), 4.56 (dd, *J* = 7.6, 3.0 Hz, 0.3H, H-11), 4.12 (d, *J* = 10.0 Hz, 0.7H, H-4), 4.03 (d, *J* = 10.0 Hz, 0.3H, H-4), 3.99 - 3.89 (m, 1.0H, H-7), 3.76 (dd, *J* = 10.8, 8.1 Hz, 0.5H, H-1), 3.69 (dd, *J* = 10.8, 8.1 Hz, 0.5H, H-1), 3.35 - 3.27 (m, 1.0H, H-1), 2.29 - 2.19 (m, 1.0H, H-3, H-5, H-9), 2.18 - 2.08 (m, 2.0H, H-3, H-5), 2.03 - 1.89 (m, 3.1H, H-2, H-10), 1.87 - 1.81 (m, 2.0H, H-6), 1.76 - 1.65 (m, 1.0H, H-9), 1.59 - 1.48 (m, 2.0H, H-2/5), 1.46, 1.45, 1.39 (3 x s, 18.0H, H-14, H-17).

^{13}C-NMR (125 MHz, $CDCl_3$, mixture of rotamers) δ [ppm] = 171.7 (s, C12), 171.5 (s, C12), 170.0 (s, C8), 169.7 (s, C8), 154.5 (s, C15), 154.1 (s, C15), 81.3 (s, C13/16), 81.1 (s, C13/16), 79.4 (s, C13/16), 79.3 (s, C13/16), 64.5 (d, C4), 64.2 (d, C4), 60.6 (d, C11), 58.1 (d, C7), 58.0 (d, C7), 46.9 (t, C1), 46.2 (t, C1), 42.3 (d, C3), 41.8 (d, C3), 34.8 (t, C6), 34.7 (t, C6), 33.5 (t, C5), 33.2 (t, C9), 33.1 (t, C9), 33.0 (t, C2), 32.5 (t, C2), 28.5 (q, C14/17), 28.2 (q, C14/17), 28.1 (q, C14/17), 27.9 (t, C10), 27.7 (t, C10).

IR $\tilde{\nu}$[cm^{-1}] = 2974 (m), 2930 (m), 2876 (w), 1737 (m), 1699 (s), 1672 (s), 1479 (w), 1450 (w), 1401 (s), 1365 (s), 1326 (m), 1283 (w), 1256 (m), 1208 (w), 1154 (s), 1123 (m), 1066 (w), 974 (w), 927 (w), 858 (w), 817 (w), 781 (w), 741 (w), 686 (w).

HRMS (ESI) calcd: m/z = 395.25405 [M+H]$^+$, 417.23599 [M+Na]$^+$, found: 395.25444 [M+H]$^+$, 417.23577 [M+Na]$^+$.

Synthesis of Boc-H$_2$-ProM2-O*t*Bu

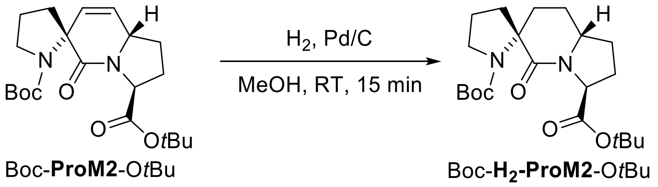

Boc-**ProM2**-O*t*Bu (100 mg, 255 µmol) was dissolved in 4.0 ml methanol and 6.0 mg palladium on carbon (10 wt% loading) were added. Hydrogen was passed into the vigorously stirred solution for 15 min. After filtering off the catalyst (washing with MeOH), the solvent was removed in the rotary evaporator and the raw product was purified by column chromatography on silica gel (cHex/EtOAc = 3:2 to 1:1), which produced a colorless oil that solidified after prolonged storage.

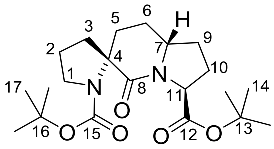

Yield 84 mg, 213 µmol, 84%.

MW 394.51 g mol^{-1} ($C_{21}H_{34}N_2O_5$).

R$_f$ 0.30 (cHex/EtOAc = 1:1).

SMP 78 – 83 °C.

$[\alpha]^{20}_{\lambda}$ (c = 0.19, $CHCl_3$): -324.4° (365 nm), -203.7° (436 nm), -114.7° (546 nm), -101.1° (579 nm), -96.0° (589 nm).

^{1}H-NMR (500 MHz, $CDCl_3$, mixture of rotamers) δ [ppm] = 4.51 (t, *J* = 8.5 Hz, 0.5H, H-11), 4.45 (t, *J* = 8.5 Hz, 0.5H, H-11), 3.66 – 3.57 (m, 2.0H, H-1, H-7), 3.54 – 3.42 (m, 1.0H, H-1), 2.48 – 2.43 (m, 0.5H, H-5), 2.38 – 2.28 (m, 2.5H, H-3, H-5, H-9/10), 2.18 – 2.06 (m, 1.0H, H-6), 2.00 – 1.86 (m, 4.5H, H-2, H-3, H-5, H-9/10), 1.83 – 1.72 (m, 2.5H, H-2, H-9/10), 1.68 – 1.61 (m, 0.5H, H-6), 1.58 – 1.52 (m, 0.5H, H-6), 1.45, 1.44, 1.43, 1.41 (4 x s, 18.0H, H-14, H-17).

^{13}C-NMR (100 MHz, $CDCl_3$, mixture of rotamers) δ [ppm] = 171.7 (s, C12), 171.4 (s, C12), 170.7 (s, C8), 170.5 (s, C8), 154.4 (s, C15), 154.1 (s, C15), 81.3 (s, C13/16), 81.0 (s, C13/16), 80.0 (s, C13/16), 79.1 (s, C13/16), 64.0 (s, C4), 63.6 (s, C4), 59.5 (d, C11), 59.4 (d, C11), 59.0 (d, C7), 58.5 (d, C7), 48.6 (t, C1), 48.3 (t, C1), 43.3 (t, C3), 42.1 (t, C3), 36.9 (t, C5), 36.1 (t, C5), 33.2 (t, C6), 33.1 (t, C6), 28.8 (q, C14/17), 28.7 (q, C14/17), 28.4 (t, C9/10), 28.3 (t, C9/10), 28.2 (t, C9/10), 28.1 (q, C14/17), 28.0 (t, C9/10), 23.0 (t, C2), 22.6 (t, C2).

IR $\tilde{\nu}$[cm^{-1}] = 2974 (m), 2929 (w), 2877 (w), 1736 (m), 1693 (s), 1655 (s), 1476 (w), 1449 (m), 1422 (m), 1386 (s), 1365 (s), 1315 (m), 1257 (m), 1213 (m), 1152 (s), 1098 (m), 1082 (m), 1003 (m), 924 (w), 845 (m), 770 (w), 633 (w), 556 (w).

GC-MS (τ = 11.02 min, EI, 70 eV): m/z (%) = 394 (8) [M$^+$], 338 (4), 321 (6), 282 (6), 265 (10), 252 (5), 237 (75) [M-CO$_2$*t*Bu,-*t*Bu]$^+$, 209 (7), 196 (100), 165 (43), 150 (14), 136 (4), 122 (5), 108 (4), 96 (17), 83 (7), 57 (28), 41 (40).

HRMS (ESI) calcd: m/z = 395.25405 [M+H]$^+$, 417.23599 [M+Na]$^+$, 433.20993 [M+K]$^{+,}$ found: 417.23576 [M+Na]$^+$, 433.20954 [M+K]$^+$.

Synthesis of Boc-ep-ProM1-O*t*Bu

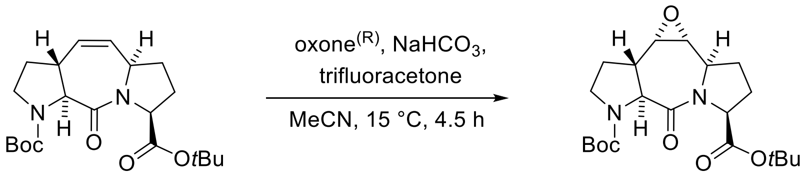

Boc-**ProM1**-O*t*Bu   Boc-**ep-ProM1**-O*t*Bu

Under argon, Boc-**ProM1**-O*t*Bu (160 mg, 408 µmol, 1.0 eq) was dissolved in 4.8 ml acetonitrile and mixed with a solution of Na_2EDTA dihydrate (7 mg, 19 µmol, 0.05 eq) in 183 µl water at 0 °C. After addition of 1,1,1-trifluoroacetone (0.37 ml, 4.1 mmol, 10.0 eq) at 0 °C, a solid mixture of $KHSO_5 \cdot ½KHSO_4 \cdot ½K_2SO_4$ (oxone(R), 706 mg, 2.3 mmol, 5.6 eq) and $NaHCO_3$ (241 mg, 2.9 mmol, 7.0 eq) were added while stirring vigorously. Within 3 h the temperature was increased to 15 °C, whereby 1,1,1-trifluoroacetone (2.59 ml, 28.9 mmol, 70.0 eq) was added in portions. After 1.5 h stirring at 15 °C, the reaction mixture was diluted with 10 ml CH_2Cl_2 and solid components were filtered off (washed with 30 ml CH_2Cl_2). The combined organic phases were then washed with 10 ml water and concentrated. A column chromatography on silica gel (EtOAc/cHex = 2:1) was performed and the solvent removed to give a colorless oil.

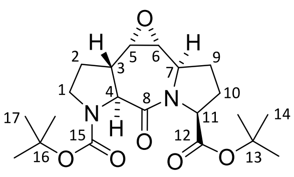

Yield 109 mg, 267 µmol, 65%.

MW 408.50 g mol^{-1} ($C_{21}H_{32}N_2O_6$).

R$_f$ 0.33 (EtOAc/cHex = 3:1).

[α]$^{20}_{\lambda}$ (c = 0.46, $CHCl_3$): -245.9° (546 nm), -216.2° (579 nm), -209.2° (589 nm).

^{1}H-NMR (600 MHz, $CDCl_3$, mixture of rotamers) δ [ppm] = 4.62 (d, *J* = 5.9 Hz, 0.6H, H-11), 4.58 (d, *J* = 5.9 Hz, 0.4H, H-11), 4.28 (t, *J* = 5.6 Hz, 0.6H, H-7), 4.20 (t, *J* = 5.6 Hz, 0.4H, H-7), 4.02 (d, *J* = 11.2 Hz, 0.6H, H-4), 3.95 (d, *J* = 11.2 Hz, 0.4H, H-4), 3.79 (dd, *J* = 10.6 Hz, 10.1 Hz, 0.4H, H-1), 3.71 (dd, *J* = 10.6 Hz, 8.2 Hz, 0.6H, H-1), 3.48 – 3.41 (m, 1.0H, H-1), 3.36 (dd, *J* = 3.8 Hz, 1.2 Hz, 1.0H, H-5), 3.12 – 3.10 (m, 1.0H, H-6), 2.70 – 2.63 (m, 1.0H, H-3), 2.55 – 2.47 (m, 1.0H, H-9), 2.11 – 2.00 (m, 4.0H, H-2, H-9, H-10), 1.96 – 1.89 (m, 1.0H, H-2), 1.45, 1.44, 1.37 (3 x s, 18.0H, H-14, H-17).

^{13}C-NMR (150 MHz, $CDCl_3$, mixture of rotamers) δ [ppm] = 171.5 (s, C12), 169.3 (s, C8), 154.8 (s, C15), 81.8 (s, C13/16), 81.6 (s, C13/16), 79.9 (s, C13/16), 79.8 (s, C13/16), 59.8 (d, C11), 59.6 (d, C11), 58.9 (d, C6), 57.8 (d, C7), 57.1 (d, C4), 56.9 (d, C4), 56.1 (d, C5), 56.0 (d, C5), 47.3 (t, C1), 46.7 (t, C1), 42.6 (d, C3), 42.1 (d, C3), 32.7 (t, C9), 29.0 (t, C2), 28.6 (q, C14, C17), 28.4 (t, C2), 28.2 (q, C14, C17), 28.2 (t, C10), 28.1 (q, C14, C17).

IR $\tilde{v}$[cm^{-1}] = 2974 (w), 2931 (w), 2887 (w), 1733 (w), 1678 (m), 1478 (w), 1435 (w), 1399 (m), 1365 (m), 1349 (m), 1281 (w), 1256 (m), 1231 (m), 1211 (w), 1154 (s), 1122 (m), 1094 (w), 1078 (w), 1064 (w), 1049 (w), 1006 (w), 989 (w), 890 (w), 864 (w), 834 (m), 803 (w), 744 (w).

HRMS (ESI) calcd: m/z = 409.23331 [M+H]$^+$, 431.21526 [M+Na]$^+$, found: 409.23351 [M+H]$^+$, 431.21521 [M+Na]$^+$.

Synthesis of Boc-TBSO-ProM1-O*t*Bu

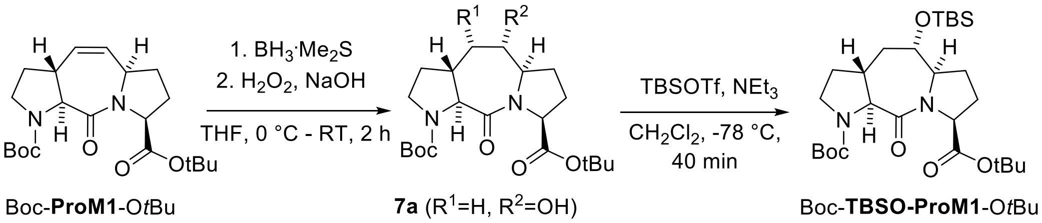

Boc-**ProM1**-O*t*Bu **7a** (R^1=H, R^2=OH) **7b** (R^1=OH, R^2=H) (2:1) Boc-**TBSO-ProM1**-O*t*Bu

Boc-**ProM1**-O*t*Bu (100 mg, 255 µmol, 1.0 eq) was dissolved under argon in 5.0 ml dry THF. To this solution BH_3 dimethyl sulfide solution (c = 10 M, 50 µl, 500 µmol, 2.0 eq) was added dropwisely at 0 °C. The reaction mixture was stirred for 10 min at 0 °C and 2 h at RT, whereupon sodium hydroxide solution (c = 3 M, 0.5 ml, 1.5 mmol, 5.9 eq) and hydrogen peroxide solution (30 wt%, 0.5 ml, 4.9 mmol, 19.2 eq) were added at 0 °C. After 1 h of vigorous stirring at RT, 10 ml sat. NaCl solution was added and the mixture was extracted with EtOAc (3 x 15 ml). The combined organic phases were washed with 10 ml semi-sat. NaCl solution, dried over Na_2SO_4 and concentrated. This was followed by column chromatography on silica gel (EtOAc/cHex = 9:1 to 19:1) producing an inseparable regioisomeric mixture (approx. 2:1 **7a**/**7b** by NMR, separation by derivatization see below) in the form of a colorless oil (73 mg, 178 µmol, 70%).

The mixture of regioisomers (approx. 2:1 **7a**/**7b** by NMR, 63 mg, 154 µmol, 1.0 eq) was dissolved under argon in 3.0 ml dry CH_2Cl_2 and treated at -78 °C with triethylamine (85 µl, 614 µmol, 4.0 eq) and TBS triflate (70 µl, 307 µmol, 2.0 eq). After stirring at this temperature for 40 min, the cooling bath was removed and the reaction was quenched by adding 5.0 ml semi-sat. $NaHCO_3$ solution. The phases were separated, the aqueous phase extracted with CH_2Cl_2 (3 x 15 ml) and the combined organic phases dried over Na_2SO_4. After column chromatographic purification on silica gel (cHex/EtOAc = 2:1 to 1:1) the desired product was obtained in diastereomeric pure form as colorless oil.

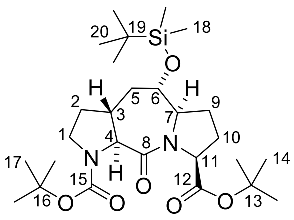

Yield 44 mg, 84 µmol, 55%.

MW 524.77 g mol^{-1} ($C_{27}H_{48}N_2O_6Si$).

R_f 0.24 (EtOAc/cHex = 1:1).

$[\alpha]^{20}_{\lambda}$ (c = 0.18, $CHCl_3$): -232.2° (365 nm), -141.9° (436 nm), -81.7° (546 nm), -72.8° (579 nm), -73.7° (589 nm).

^{1}H-NMR (500 MHz, $CDCl_3$, mixture of rotamers) δ [ppm] = 4.69 – 4.67 (m, 0.6H, H-11), 4.61 (t, *J* = 5.8 Hz, 0.4H, H-11), 4.18 (d, *J* = 10.1 Hz, 0.6H, H-4), 4.10 (d, *J* = 11.0 Hz, 0.4H, H-4), 3.90 – 3.85 (m, 1.0H, H-7), 3.79 – 3.67 (m, 2.0H, H-1, H-6), 3.32 – 3.25 (m, 1.0H, H-1), 2.30 – 2.12 (m, 3.0H, H-3, H-5, H-9), 2.01 – 1.89 (m, 4.0H, H-2, H-9, H-10), 1.63 – 1.51 (m, 2.0H, H-2, H-5), 1.46, 1.45, 1.39 (3 x s, 18.0H, H-14, H-17), 0.88 (s, 9.0H, H-20), 0.08, 0.06 (2 x s, 6.0H, H-18).

^{13}C-NMR (150 MHz, $CDCl_3$, mixture of rotamers) δ [ppm] = 171.8 (s, C12), 169.9 (s, C8), 169.7 (s, C8), 154.6 (s, C15), 81.5 (C13/C16), 81.3 (s, C13/C16), 79.7 (s, C13/C16), 79.6 (s, C13/C16), 72.9 (d, C6), 72.8 (d, C6), 64.1 (d, C4), 63.8 (d, C4), 63.1 (d, C7), 63.0 (d, C7), 60.8 (d, C11), 46.9 (t, C1), 46.2 (t, C1), 43.4 (t, C5), 40.8 (d, C3), 40.2 (d, C3), 33.2 (t, C2), 32.7 (t, C2), 30.2 (t, C9), 29.9 (t, C9), 28.6 (q, C14, C17), 28.3 (q, C14, C17), 28.2 (q, C14, C17), 27.6 (t, C10), 27.4 (t, C10), 25.9 (q, C20), 17.6 (s, C19), -4.4 (q, C18), -5.1 (q, C18).

IR $\tilde{v}$ [cm^{-1}] = 2957 (m), 2928 (m), 2857 (w), 1737 (m), 1704 (s), 1682 (w), 1453 (w), 1402 (s), 1365 (m), 1346 (w), 1325 (w), 1288 (w), 1255 (m), 1215 (w), 1154 (s), 1133 (m), 1088 (m), 1067 (m), 1006 (w), 909 (w), 856 (w), 837 (m), 774 (w), 688 (w), 559 (w), 408 (w).

HRMS (ESI) calcd: m/z = 547.31738 [M+Na]$^+$, 563.29132 [M+K]$^+$, found: 547.31725 [M+Na]$^+$, 563.29106 [M+K]$^+$.

Synthesis of Boc-(HO)$_2$-ProM1-O*t*Bu via Os(VIII)-catalysis

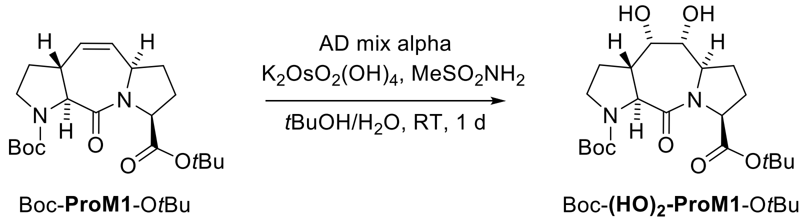

AD mix alpha[*] (840 mg) and $K_2OsO_2(OH)_4$ (6 mg, 16 µmol, 0.05 eq) were dissolved under argon in 6.0 ml *t*BuOH/H_2O (1:1). Methanesulfonic acid amide (30 mg, 315 µmol, 1.03 eq) and Boc-**ProM1**-O*t*Bu (120 mg, 306 µmol, 1.00 eq) were added to the yellow two-phase mixture at 0 °C. Within 3 h the reaction mixture was warmed to RT and stirred for 24 h at RT, whereupon 15 ml of an aqueous Na_2SO_3 solution (10 wt%) were added. Extraction was performed with EtOAc (3 x 30 ml). The combined organic phases were washed with sat. NaCl solution (10 ml), dried over Na_2SO_4 and the solvent was removed. The raw product was purified by column chromatography on ultrapure SiO_2 (EtOAc/EtOH = 40:1), which after drying in vacuum led to the α-Diol in the form of a colorless solid (90 mg, 211 µmol, 69%) (analytical data: see below).

[*] Usage of AD mix beta resulted in decreased yields (50%) of the α-diol.

Synthesis of α- und β-Boc-(HO)$_2$-ProM1-O*t*Bu via Ru(VIII)-catalysis

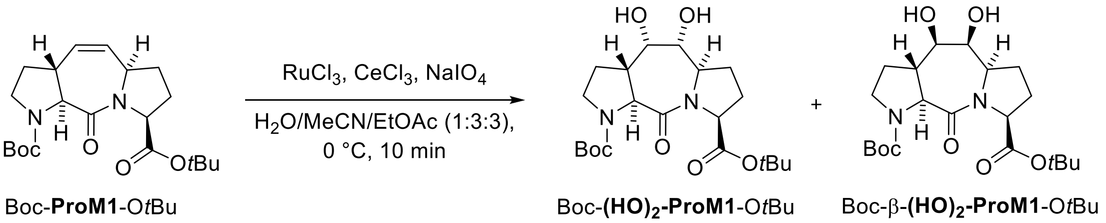

Under argon, sodium periodate (240 mg, 1.12 mmol, 1.50 eq) and cerium(III)-chloride heptahydrate (45 mg, 121 µmol, 0.15 eq) were suspended in 0.75 ml water and heated at 60 °C for 1 min. At 0 °C 3.0 ml ethyl acetate and 4.5 ml acetonitrile were added to the light yellow suspension. A solution of ruthenium(III)-chloride trihydrate (9.8 mg, 37 µmol, 0.05 eq) in 0.75 ml water and a solution of Boc-**ProM1**-O*t*Bu (300 mg, 764 µmol, 1.00 eq) in 1.5 ml ethyl acetate were then directly added. After vigorous stirring for 10 min at 0 °C the reaction was quenched by adding 9.0 ml sat. Na_2SO_3 solution. The reaction mixture was diluted with 20 ml water and extracted with ethyl acetate (3 x 50 ml). The combined organic phases were washed with 20 ml sat. NaCl solution, dried over Na_2SO_4 and the solvent was removed. Purification by column chromatography on ultrapure SiO_2 resulted in pure α-diol as a colorless oil that slowly solidified to a colorless solid. In addition, a mixture of α-/β-diol was obtained (approx. 1:1 ratio on TLC, separation see below).

α-diol:

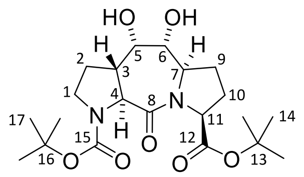

Yield 203 mg, 476 µmol, 62%.
MW 426.51 g mol^{-1} ($C_{21}H_{34}N_2O_7$).
R$_f$ 0.60 (EtOAc/EtOH = 9:1).
Smp 112 - 115 °C.

[α]$^{20}_{\lambda}$ (c = 0.29, $CHCl_3$): -270.1° (436 nm), -153.8° (546 nm), -135.2° (579 nm), -130.8° (589 nm).

^{1}H-NMR (500 MHz, $CDCl_3$, mixture of rotamers) δ [ppm] = 4.69 (d, *J* = 10.1 Hz, 0.6H, H-4), 4.63 - 4.54 (m, 1.4H, H-4, H-11), 4.24 - 4.12 (m, 1.0H, H-7), 4.11 (br. s., 1.0H, H-5), 3.96 (d, *J* = 3.4 Hz, 0.3H, OH), 3.81 (d, *J* = 3.4 Hz, 0.6H, OH), 3.78 - 3.69 (m, 1.0H, H-1), 3.68 - 3.59 (m, 1.0H, H-6), 3.44 (d, *J* = 8.5 Hz, 0.4H, OH), 3.37 (d, *J* = 8.5 Hz,

0.6H, OH), 3.29 (td, J = 11.3, 5.5 Hz, 1.0H, H-1), 2.49 - 2.41 (m, 1.0H, H-3), 2.20 - 1.94 (m, 5.0H, H-2, H-9, H-10), 1.80 - 1.74 (m, 1.0H, H-2), 1.45, 1.44, 1.44, 1.38 (4 x s, 18.0H, H-14, H-17).

^{13}C-NMR (150 MHz, $CDCl_3$, mixture of rotamers) δ [ppm] = 171.7 (s, C12), 171.6 (s, C12), 171.0 (s, C8), 170.9 (s, C8), 154.9 (s, C15), 154.6 (s, C15), 81.7 (s, C13/16), 81.6 (s, C13/16), 79.9 (s, C13/16), 79.8 (s, C13/16), 74.3 (d, C6), 74.1 (d, C6), 70.5 (d, C5), 70.4 (d, C5), 60.8 (d, C11), 60.7 (d, C11), 56.1 (d, C4, C7), 56.0 (d, C4, C7), 55.9 (d, C4, C7), 55.9 (d, C4, C7), 46.8 (t, C1), 46.1 (t, C1), 46.0 (d, C3), 45.5 (d, C3), 29.4 (t, C9), 29.3 (t, C9), 28.7 (q, C14, C17), 28.4 (q, C14, C17), 28.2 (q, C14, C17), 28.2 (t, C2), 27.7 (t, C10), 27.5 (t, C10).

IR $\tilde{\nu}$ [cm^{-1}] = 3405 (w), 2974 (w), 2928 (w), 2877 (w), 2854 (w), 1735 (m), 1675 (m), 1651 (m), 1405 (m), 1366 (m), 1255 (m), 1154 (s), 1125 (m), 1101 (m), 1067 (w), 1047 (w), 935 (w), 842 (w), 768 (w), 684 (w), 540 (w).

HRMS (ESI) calcd: m/z = 449.22582 [M+Na]$^+$, 465.19976 [M+K]$^+$, found: 449.22575 [M+Na]$^+$, 465.19967 [M+K]$^+$.

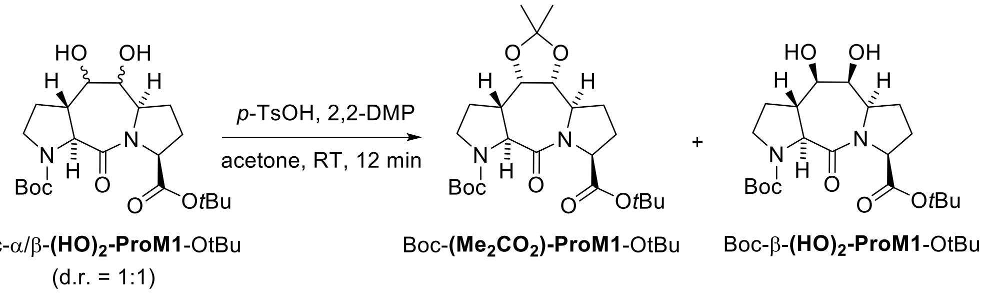

The α-/β-mixture (80 mg, 187 µmol, 1.0 eq) was dissolved in 4.0 ml acetone/2,2-dimethoxypropane (1:1), mixed with *para*-toluenesulfonic acid (7 mg, 38 µmol, 0.2 eq) and stirred for 12 min at RT. Semi-sat. $NaHCO_3$ solution (4.0 ml) were added, the volatile components removed at the rotary evaporator and the residual aqueous solution extracted with EtOAc (3 x 10 ml). After washing, drying and column chromatography on SiO_2 (EtOAc/EtOH = 40:1 to 30:1) diastereomerically pure β-diol was obtained as colorless oil.

β-diol:

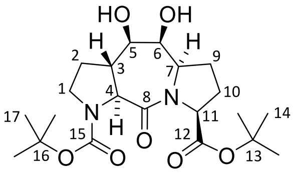

Yield 35 mg, 82 µmol, 11% (over two steps).

MW 426.51 g mol^{-1} ($C_{21}H_{34}N_2O_7$).

R$_f$ 0.49 (EtOAc/EtOH = 9:1).

$[\alpha]^{20}_{\lambda}$ (c = 0.20, $CHCl_3$): -332.2° (365 nm), -211.3° (436 nm), -125.2° (546 nm), -113.7° (579 nm), -113.3° (589 nm).

^{1}H-NMR (500 MHz, $CDCl_3$, mixture of rotamers) δ [ppm] = 5.05 (dd, J = 9.5 Hz, 3.4 Hz, 1.0H, HO-6), 4.64 (dd, J = 9.2 Hz, 3.4 Hz, 0.8H, H-11), 4.57 (dd, J = 8.1 Hz, 3.9 Hz, 0.2H, H-11), 4.16 – 4.10 (m, 1.0H, H-7), 4.04 (d, J = 9.9 Hz, 0.8H, H-4), 3.95 (d, J = 9.9 Hz, 0.2H, H-4), 3.81 (dd, J = 9.5 Hz, 2.9 Hz, 1.3H, H-1, H-6), 3.77 – 3.74 (m, 0.7H, H-1), 3.49 (dt, J = 9.1 Hz, 2.9 Hz, 1.0H, H-5), 3.33 (td, J = 11.5 Hz, 5.1 Hz, 1.0H, H-1), 2.86 (d, J = 9.1 Hz, 1.0H, HO-5), 2.33 – 2.20 (m, 4.0H, H-2, H-3, H-9), 2.16 – 1.99 (m, 2.0H, H-10), 1.76 – 1.66 (m, 1.0H, H-2), 1.49, 1.47, 1.47, 1.39 (4 x s, 18.0H, H-14, H-17).

^{13}C-NMR (125 MHz, $CDCl_3$, mixture of rotamers) δ [ppm] = 175.0 (s, C12), 174.7 (s, C12), 169.4 (s, C8), 169.2 (s, C8), 154.6 (s, C15), 153.8 (s, C15), 83.7 (s, C13/16), 83.5 (s, C13/16), 80.0 (s, C13/16), 79.8 (s, C13/16), 77.7 (d, C5), 77.4 (d, C5), 72.9 (d, C6), 72.7 (d, C6), 60.4 (d, C4), 60.2 (d, C4), 60.0 (d, C11), 59.9 (d, C11), 58.4 (d, C7), 58.4 (d, C7), 47.0 (t, C1), 46.2 (t, C1), 43.5 (d, C3), 42.8 (d, C3), 30.6 (t, C2), 30.1 (t, C2), 29.6 (t, C9), 29.5 (t, C9), 28.6 (q, C14, C17), 28.3 (q, C14, C17), 28.0 (q, C14, C17), 27.7 (t, C10), 27.5 (t, C10).

IR $\tilde{\nu}$ [cm^{-1}] = 3675 (w), 3405 (w), 2983 (s), 2973 (s), 2922 (s), 2901 (m), 1698 (m), 1687 (m), 1451 (m), 1405 (s), 1394 (s), 1369 (m), 1331 (m), 1250 (m), 1164 (m), 1077 (s), 1060 (s), 1057 (s), 891 (w), 828 (w), 419 (w), 407 (w).

HRMS (ESI) calcd: m/z = 427.24388 [M+H]$^+$, 449.22582 [M+Na]$^+$, found: 427.24417 [M+H]$^+$, 449.22592 [M+Na]$^+$.

Synthesis of Boc-(Me$_2$CO$_2$)-ProM1-OtBu

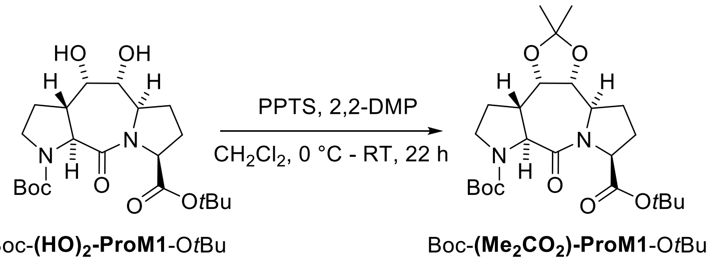

Under argon Boc-(HO)$_2$-ProM1-OtBu (20 mg, 46.9 µmol, 1.0 eq) was dissolved together with PPTS (1.5 mg, 6.0 µmol, 0.1 eq) in 2.0 ml dry CH_2Cl_2. Then, 2,2-dimethoxypropane (244 µl, 2.0 mmol, 42.6 eq) was added to the reaction mixture at 0 °C which was stirred for 2 h at 0 °C and 6 h at RT. More 2,2-dimethoxypropane (244 µl, 2.0 mmol, 42.6 eq) was added and the mixture was stirred overnight. The organic phase was diluted with 10 ml CH_2Cl_2, washed with 5 ml sat. $NaHCO_3$ solution and 5 ml sat. NaCl solution, dried over Na_2SO_4 and concentrated at the rotary evaporator. The raw product was purified by column chromatography on ultrapure silica gel (cHex/EtOAc = 1:1) resulting in a colorless oil after drying *in vacuo*.

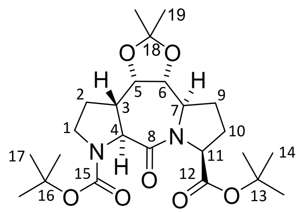

Yield 12 mg, 26 µmol, 55%.

MW 466.58 g mol^{-1} ($C_{24}H_{38}N_2O_7$).

R$_f$ 0.40 (EtOAc/cHex = 2:1).

[α]$^{20}_\lambda$ (c = 0.10, $CHCl_3$): -229.0° (436 nm), -131.7° (546 nm), -115.7° (579 nm), -114.3° (589 nm).

^{1}H-NMR (500 MHz, $CDCl_3$, mixture of rotamers) δ [ppm] = 4.67 (d, *J* = 10.5 Hz, 0.7H, H-4), 4.56 – 4.49 (m, 1.3H, H-4, H-11), 4.42 (dd, *J* = 4.4 Hz, 2.6 Hz, 1.0H, H-5), 4.12 – 4.07 (m, 0.7H, H-7), 4.00 – 3.91 (m, 1.3H, H-6, H-7), 3.79 (dd, *J* = 10.9 Hz, 8.1 Hz, 0.3H, H-1), 3.71 (dd, *J* = 10.9 Hz, 8.1 Hz, 0.7H, H-1), 3.35 (td, *J* = 10.9 Hz, 5.5 Hz, 1.0H, H-1), 2.67 – 2.59 (m, 1.0H, H-3), 2.19 – 2.00 (m, 4.0H, H-2/H-9/H-10), 1.91 – 1.84 (m, 2.0H, H-2/H-9/H-10), 1.51 (s, 1.0H, H-19), 1.47, 1.45, 1.42 (3 x s, 18.0H, H-14, H-17), 1.39 (s, 2.0H, H-19), 1.37, 1.36 (2 x s, 3.0H, H-19).

^{13}C-NMR (125 MHz, $CDCl_3$, mixture of rotamers) δ [ppm] = 171.7 (s, C12), 171.5 (s, C12), 169.9 (s, C8), 169.8 (s, C8), 154.6 (s, C15), 154.1 (s, C15), 108.5 (s, C18), 81.6 (s, C13/C16), 81.4 (s, C13/C16), 79.7 (s, C13/C16), 79.6 (s, C13/C16), 77.9 (d, C6), 77.5 (d, C6), 75.4 (d, C5), 59.9 (d, C11), 56.8 (d, C7), 56.8 (d, C4), 56.6 (d, C4), 46.5 (t, C1), 45.9 (t, C1), 42.9 (d, C3), 42.3 (d, C3), 29.1 (t, C9), 28.8 (t, C9), 28.8 (q, C19), 28.7 (q, C14, C17), 28.6 (t, C2), 28.5 (q, C14, C17), 28.2 (q, C19), 28.0 (q, C14, C17), 27.4 (t, C10), 27.2 (t, C10), 26.2 (q, C19), 26.1 (q, C19).

IR $\tilde{v}$[cm^{-1}] = 2975 (w), 2926 (m), 2877 (w), 2859 (w), 1735 (m), 1702 (s), 1687 (s), 1457 (w), 1405 (m), 1367 (m), 1343 (w), 1330 (w), 1241 (w), 1220 (m), 1154 (s), 1120 (w), 1069 (w), 1035 (w), 981 (w), 921 (w), 864 (w), 792 (w).

HRMS (ESI) calcd: m/z = 489.25712 [M+Na]$^+$, 505.23106 [M+K]$^+$, found: 489.25707 [M+Na]$^+$, 505.23115 [M+K]$^+$.

Synthesis of Boc-(HO)$_2$-ProM2-O*t*Bu

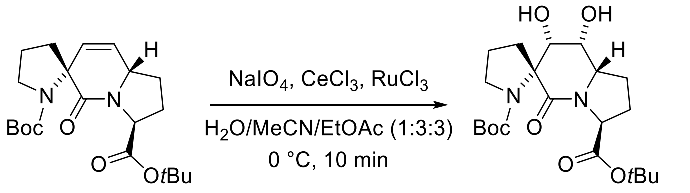

Boc-**ProM2**-O*t*Bu Boc-**(HO)$_2$-ProM2**-OtBu

Sodium periodate (320 mg, 1.50 mmol, 1.50 eq) and cerium(III)-chloride heptahydrate (60 mg, 161 µmol, 0.16 eq) were suspended in 1.0 ml of water and heated at 60 °C for 1 min, resulting in a light yellow color. After cooling to 0 °C, 4.0 ml ethyl acetate and 6.0 ml acetonitrile were added. A solution of ruthenium(III)-chloride trihydrate (13 mg, 50 µmol, 0.05 eq) in 1.0 ml water was added while stirring vigorously. After 2 min stirring at 0 °C a solution of Boc-**ProM2**-O*t*Bu (400 mg, 1.02 mmol, 1.00 eq) in 2.0 ml ethyl acetate was added to the reaction mixture. The greenish mixture was stirred vigorously for 10 min at 0 °C (brown color) and then treated with 12 ml sat. Na_2SO_3 solution. After dilution with 10 ml water, it was extracted with EtOAc (3 x 50 ml). The combined organic phases were washed with 30 ml sat. NaCl solution, dried over Na_2SO_4 and stirred for 30 min over activated carbon. Subsequently, the activated carbon was removed by filtration and the organic solution was concentrated and the residue subjected to column chromatography on silica gel (EtOAc/cHex = 3:1) to give diastereomerically pure product as a colorless foam.

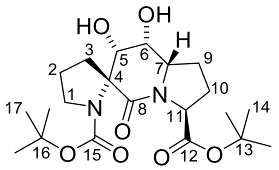

Yield 263 mg, 617 µmol, 61%.

MW 426.51 g mol^{-1} ($C_{21}H_{34}N_2O_7$).

R$_f$ 0.31 (EtOAc/cHex = 4:1).

$[\alpha]^{20}_{\lambda}$ (c = 0.25, $CHCl_3$): -238.0° (365 nm), -145.2° (436 nm), -81.4° (546 nm), -71.2° (579 nm), -69.9° (589 nm).

^{1}H-NMR (500 MHz, $CDCl_3$, mixture of rotamers) δ [ppm] = 4.46 (t, *J* = 8.3 Hz, 0.7H, H-11), 4.40 (t, *J* = 7.9 Hz, 0.3H, H-11), 4.22 – 4.18 (m, 1.1H, H-5), 4.11 – 4.06 (m, 0.7H, H-6), 3.96 – 3.83 (m, 0.8H, OH), 3.83 – 3.78 (m, 0.3H, H-6), 3.74 – 3.70 (m, 1.2H, H-7), 3.59 – 3.42 (m, 2.0H, H-1), 2.79 – 2.63 (m, 1.0H, H-3), 2.36 – 2.24 (m, 2.0H, H-9, H-10), 2.05 – 1.99 (m, 1.0H, H-3), 1.92 – 1.75 (m, 4.0H, H-2, H-9, H-10), 1.44, 1.41, 1.39 (3 x s, 18.0H, H-14, H-17).

^{13}C-NMR (125 MHz, $CDCl_3$, mixture of rotamers) δ [ppm] = 171.7 (s, C12), 170.8 (s, C12), 169.6 (s, C8), 169.3 (s, C8), 154.4 (s, C15), 154.2 (s, C15), 81.6 (s, C13), 81.5 (s, C13), 80.8 (s, C16), 79.7 (s, C16), 72.9 (d, C5), 72.7 (d, C5), 71.5 (d, C6), 71.2 (d, C6), 69.4 (s, C4), 68.9 (s, C4), 60.2 (d, C11), 60.1 (d, C11), 59.1 (d, C7), 58.4 (d, C7), 48.6 (t, C1), 48.4 (t, C1), 34.5 (t, C3), 32.9 (t, C3), 31.0 (t, C9), 30.8 (t, C9), 28.6 (q, C14, C17), 28.6 (q, C14, C17), 28.5 (q, C14, C17), 28.2 (t, C10), 28.1 (q, C14, C17), 28.0 (t, C10), 23.5 (t, C2), 23.4 (t, C2).

IR $\tilde{v}$ [cm^{-1}] = 3386 (w), 2978 (w), 2926 (m), 2879 (w), 2856 (w), 1738 (m), 1694 (s), 1666 (m), 1633 (m), 1452 (m), 1391 (s), 1367 (s), 1294 (w), 1256 (m), 1214 (w), 1149 (s), 1095 (w), 1038 (w), 995 (w), 970 (w), 932 (w), 911 (w), 846 (w), 766 (w).

HRMS (ESI) calcd: m/z = 449.22582 [M+Na]$^+$, 465.19976 [M+K]$^+$, found: 449.22577 [M+Na]$^+$, 465.19958 [M+K]$^+$.

Synthesis of Boc-(MeO)$_2$-ProM1-O*t*Bu

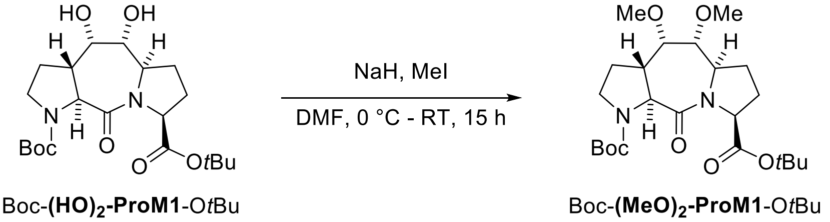

Boc-**(HO)$_2$-ProM1**-O*t*Bu (55.0 mg, 129 µmol, 1.0 eq) was dissolved in 1.7 ml DMF under argon, sodium hydride (22.7 mg, 60% suspension in mineral oil, 567 µmol, 4.4 eq) was added at RT and the mixture was stirred for 10 min at RT (weak gas evolution). At 0 °C methyl iodide (106 µL, 1.70 mmol, 13.2 eq) was added. The reaction mixture was allowed to warm to RT over 3 h and was stirred overnight. At RT the mixture was treated with 4.0 ml of NH_4Cl/NH_3 buffer solution (pH = 8) for 45 min. After diluting with 30 ml water it was extracted with EtOAc (3 x 50 ml). The combined organic phases were washed with water (2 x 50 ml), sat. NaCl solution (30 ml) and then dried over $MgSO_4$. After 90 min stirring over activated carbon, column chromatographic purification was performed on silica gel (EtOAc/cHex = 2:1) to obtain a colorless oil which solidified after prolonged storage.

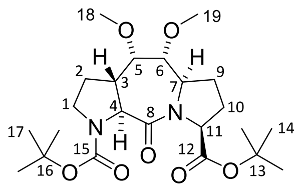

Yield 44 mg, 97 µmol, 75%.

MW 454.56 g mol^{-1} ($C_{23}H_{38}N_2O_7$).

R$_f$ 0.38 (EtOAc/cHex = 4:1).

Smp 169 - 172 °C.

[α]$^{20}_{\lambda}$ (c = 0.20, $CHCl_3$): -339.3° (365 nm), -206.0° (436 nm), -117.5° (546 nm), -102.8° (579 nm), -100.2° (589 nm).

^{1}H-NMR (500 MHz, $CDCl_3$, mixture of rotamers) δ [ppm] = 4.67 – 4.65 (m, 0.7H, H-11), 4.63 (d, *J* = 10.2 Hz, 0.7H, H-4), 4.59 – 4.56 (m, 0.3H, H-11), 4.50 (d, *J* = 9.9 Hz, 0.3H, H-4), 4.33 – 4.23 (m, 1.0H, H-7), 3.82 – 3.80 (m, 1.0H, H-5), 3.79 – 3.68 (m, 1.0H, H-1), 3.62, 3.61 (2 x s, 3.0H, H-18), 3.47 (s, 3.0H, H-19), 3.36 – 3.32 (m, 1.0H, H-6), 3.31 – 3.25 (m, 1.0H, H-1), 2.37 – 2.30 (m, 1.0H, H-3), 2.23 – 2.16 (m, 1.0H, H-9), 2.05 – 1.87 (m, 4.0H, H-2, H-9, H-10), 1.82 – 1.76 (m, 1.0H, H-2), 1.46, 1.44, 1.39 (3 x s, 18.0H, H-14, H-17).

^{13}C-NMR (125 MHz, $CDCl_3$, mixture of rotamers) δ [ppm] = 172.1 (s, C12), 171.9 (s, C12), 170.3 (s, C8), 170.2 (s, C8), 154.6 (s, C15), 154.4 (s, C15), 85.5 (d, C6), 85.4 (d, C6), 81.6 (s, C13/16), 81.3 (s, C13/16), 79.6 (s, C13/16), 79.5 (s, C13/16), 76.0 (d, C5), 75.9 (d, C5), 62.3 (q, C18), 62.3 (q, C18), 60.7 (d, C11), 60.7 (d, C11), 58.3 (q, C19), 58.3 (q, C19), 56.6 (d, C4), 56.5 (d, C4), 55.5 (d, C7), 55.4 (d, C7), 46.5 (t, C1), 46.1 (d, C3), 45.9 (t, C1), 45.5 (d, C3), 29.1 (t, C9), 29.0 (t, C9), 28.6 (q, C14, C17), 28.3 (t, C2), 28.3 (q, C14, C17), 28.2 (q, C14, C17), 27.8 (t, C2), 27.7 (t, C10), 27.6 (t, C10).

IR $\tilde{\nu}$ [cm^{-1}] = 2974 (m), 2929 (m), 1735 (m), 1699 (s), 1682 (s), 1454 (w), 1403 (s), 1365 (m), 1340 (w), 1325 (w), 1257 (w), 1229 (w), 1156 (s), 1120 (s), 1077 (m), 935 (w), 844 (w), 748 (w).

HRMS (ESI) calcd: m/z = 455.27518 [M+H]$^+$, 477.25712 [M+Na]$^+$, found: 455.27541 [M+H]$^+$, 477.25701 [M+Na]$^+$.

Synthesis of Boc-(EtO)$_2$-ProM1-O*t*Bu

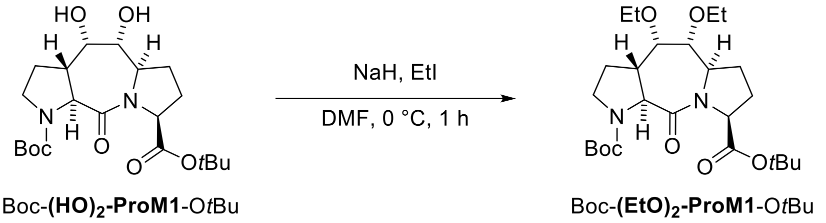

Under argon Boc-**(HO)$_2$-ProM1**-O*t*Bu (153 mg, 359 µmol, 1.0 eq) was dissolved in 1.5 ml dry DMF and sodium hydride (57 mg, 60%-suspension in mineral oil, 1.43 mmol, 4.0 eq) was added at 0 °C. After 5 min stirring at 0 °C, ethyl iodide (692 µl, 8.61 mmol, 24.0 eq) was added and the mixture was stirred for 1 h. The reaction mixture was treated with 10 ml semi-sat. NH_4Cl/NH_3 buffer solution (pH = 9) for 1 h, diluted with additional buffer solution (40 ml) and extracted with ethyl acetate (3 x 50 ml). The combined organic phases were washed with water (3 x 50 ml) and 30 ml sat. NaCl solution, dried over $MgSO_4$ and concentrated. A colorless solid was obtained after column chromatography on silica gel (EtOAc/cHex = 2:1) and solvent removal.

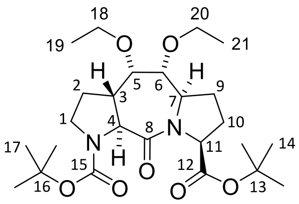

Yield 136 mg, 282 µmol, 79%.

MW 482.62 g mol^{-1} ($C_{25}H_{42}N_2O_7$).

R$_f$ 0.36 (EtOAc/cHex = 4:1).

Smp 167 - 169 °C.

[α]$^{20}_{\lambda}$ (c = 0.30, $CHCl_3$): -321.0° (365 nm), -196.3° (436 nm), -111.7° (546 nm), -98.7° (579 nm), -96.8° (589 nm).

^{1}H-NMR (500 MHz, $CDCl_3$, mixture of rotamers) δ [ppm] = 4.68 – 4.64 (m, 1.3H, H-4, H-11), 4.58 – 4.55 (m, 0.3H, H-11), 4.51 (d, *J* = 9.8 Hz, 0.3H, H-4), 4.37 – 4.25 (m, 1.0H, H-7), 3.99 – 3.93 (m, 1.0H, H-18), 3.84 (br. s., 1.0H, H-5), 3.80 – 3.68 (m, 2.0H, H-1, H-20), 3.60 – 3.40 (m, 3.0H, H-6, H-18, H-20), 3.32 – 3.25 (m, 1.0H, H-1), 2.35 – 2.28 (m, 1.0H, H-3), 2.23 – 2.13 (m, 1.0H, H-9), 2.05 – 1.87 (m, 4.0H, H-2, H-9, H-10), 1.78 – 1.72 (m, 1.0H, H-2), 1.47, 1.46, 1.44, 1.40 (4 x s, 18.0H, H-14, H-17), 1.24 – 1.19 (m, 6.0H, H-19, H-21).

^{13}C-NMR (125 MHz, $CDCl_3$, mixture of rotamers) δ [ppm] = 171.9 (s, C12), 171.7 (s, C12), 170.2 (s, C8), 170.1 (s, C8), 154.5 (s, C15), 154.3 (s, C15), 83.2 (d, C6), 81.3 (s, C13/C16), 81.1 (s, C13/C16), 79.3 (s, C13/C16), 74.9 (d, C5), 74.7 (d, C5), 69.5 (t, C18), 69.4 (t, C18), 65.7 (t, C20), 60.6 (d, C11), 56.6 (d, C4), 56.4 (d, C4), 55.4 (d, C7), 55.3 (d, C7), 46.4 (t, C1), 46.1 (d, C3), 45.7 (t, C1), 45.5 (d, C3), 28.9 (t, C9), 28.8 (t, C9), 28.5 (q, C14, C17), 28.2 (q, C14, C17), 28.1 (t, C2), 28.0 (q, C14, C17), 27.7 (t, C10), 27.5 (t, C10), 15.8 (q, C19/C21), 15.4 (q, C19/C21).

IR $\tilde{v}$[cm^{-1}] = 2973 (m), 2927 (m), 2872 (w), 1735 (m), 1699 (s), 1680 (m), 1479 (w), 1454 (w), 1402 (s), 1365 (m), 1327 (w), 1291 (w), 1257 (w), 1230 (w), 1208 (w), 1157 (s), 1120 (m), 1092 (m), 1074 (m), 981 (w), 931 (w), 846 (w), 745 (w), 686 (w), 470 (w).

HRMS (ESI) calcd: m/z = 483.30648 [M+H]$^+$, 505.28842 [M+Na]$^+$, found: 483.30641 [M+H]$^+$, 505.28773 [M+Na]$^+$.

Synthesis of Boc-(MeO)$_2$-ProM2-O*t*Bu

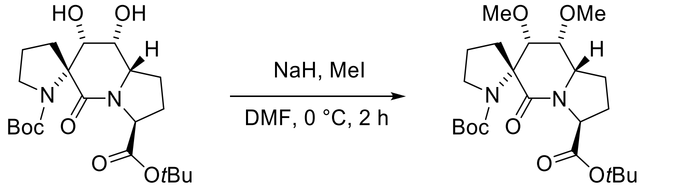

Boc-**(HO)$_2$-ProM2**-O*t*Bu   Boc-**(MeO)$_2$-ProM2**-O*t*Bu

Boc-(HO)$_2$-**ProM2**-O*t*Bu (88 mg, 206 µmol, 1.0 eq) was dissolved under argon in 1.5 ml dry DMF and sodium hydride (34 mg, 60% suspension in mineral oil, 844 µmol, 4.1 eq) was added at 0 °C. After stirring for 5 min, methyl iodide (316 µl, 5.1 mmol, 24.5 eq) was added and the mixture was stirred for 2 h at 0 °C. Subsequently, the reaction mixture was treated with 8.0 ml of semi-sat. NH_4Cl/NH_3 buffer solution (pH = 9) and warmed to RT within 1 h. Further 50 ml buffer solution were added and the mixture was extracted with ethyl acetate (3 x 50 ml). The combined organic phases were washed with water (3 x 50 ml), sat. NaCl solution (50 ml), dried over $MgSO_4$ and concentrated. A slightly yellowish oil was obtained after column chromatography on silica gel (cHex/EtOAc = 3:2 to 1:1).

Yield 93 mg, 205 µmol, 99%.

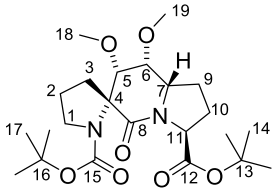

MW 454.56 g mol^{-1} ($C_{23}H_{38}N_2O_7$).

R$_f$ 0.40 (EtOAc/cHex = 1:1).

$[\alpha]^{20}_{\lambda}$ (c = 0.45, $CHCl_3$): -199.2° (365 nm), -124.2° (436 nm), -72.2° (546 nm), -64.1° (579 nm), -63.0° (589 nm).

^{1}H-NMR (500 MHz, $CDCl_3$, mixture of rotamers) δ [ppm] = 4.47 – 4.39 (m, 1.0H, H-11), 3.98 – 3.91 (m, 1.0H, H-5), 3.87 – 3.79 (m, 1.7H, H-6, H-7), 3.74 – 3.68 (m, 0.3H, H-1), 3.66 – 3.62 (m, 0.3H, H-6), 3.61 – 3.57 (m, 0.8H, H-1), 3.56 (s, 3.0H, H-18), 3.47 (s, 3.0H, H-19), 3.45 – 3.40 (m, 0.8H, H-1), 2.70 – 2.52 (m, 1.0H, H-3), 2.36 – 2.29 (m, 1.0H, H-10), 2.28 – 2.19 (m, 1.0H, H-9), 2.14 – 2.07 (m, 1.0H, H-3), 1.98 – 1.90 (m, 1.0H, H-2), 1.84 – 1.64 (m, 3.0H, H-2, H-9, H-10), 1.44, 1.43 (2 x s, 18.0H, H-14, H-17).

^{13}C-NMR (125 MHz, $CDCl_3$, mixture of rotamers) δ [ppm] = 171.3 (s, C12), 170.8 (s, C12), 168.9 (s, C8), 168.6 (s, C8), 154.2 (s, C15), 153.8 (s, C15), 81.1 (s, C13/C16), 81.0 (d, C6), 80.8 (d, C6), 80.1 (d, C5), 79.5 (s, C13/C16), 79.4 (d, C5), 69.1 (s, C4), 68.9 (s, C4), 61.4 (q, C18), 61.1 (q, C18), 59.5 (d, C11), 58.7 (d, C7), 58.3 (q, C19), 58.1 (q, C19), 48.7 (t, C1), 48.6 (t, C1), 34.6 (t, C3), 32.9 (t, C3), 31.4 (t, C9), 31.3 (t, C9), 28.8 (q, C14, C17), 28.6 (q, C14, C17), 28.1 (q, C14, C17), 27.9 (t, C10), 23.3 (t, C2), 22.9 (t, C2).

IR $\tilde{\nu}$ [cm^{-1}] = 2974 (s), 2928 (m), 2901 (m), 1739 (m), 1695 (s), 1661 (m), 1450 (m), 1392 (s), 1366 (s), 1286 (w), 1255 (m), 1228 (m), 1154 (s), 1116 (m), 1077 (s), 1066 (s), 1057 (s), 1027 (m), 937 (w), 892 (w), 860 (w), 770 (w), 551 (w), 407 (w).

HRMS (ESI) calcd: m/z = 455.27518 $[M+H]^+$, 477.25712 $[M+Na]^+$, found: 455.27562 $[M+H]^+$, 477.25721 $[M+Na]^+$.

Synthesis of Boc-(EtO)$_2$-ProM2-O*t*Bu

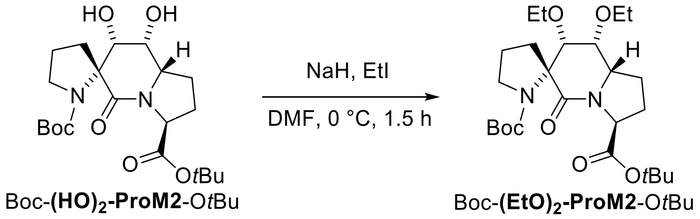

At 0 °C Boc-**(HO)$_2$-ProM2**-O*t*Bu (125 mg, 293 µmol, 1.0 eq) was dissolved under argon in 1.5 ml dry DMF and sodium hydride (47 mg, 60%-suspension in mineral oil, 1.18 mmol, 4.0 eq) was added. After addition of ethyl iodide (570 µl, 7.09 mmol, 24.2 eq), the reaction mixture was treated with NH_4Cl/NH_3 buffer solution (pH = 9) for 1.5 h at 0 °C and 1 h at RT. Further 40 ml of buffer solution were added and the mixture was extracted with ethyl acetate (3 x 50 ml). The combined organic phases were washed with water (3 x 50 ml), sat. NaCl solution (30 ml), dried over $MgSO_4$ and concentrated. The raw product was purified by column chromatography (silica gel, EtOAc/cHex = 1:2 to 1:1), which gave rise to a yellow, viscous oil after solvent removal.

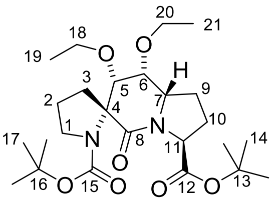

Yield 141 mg, 292 µmol, 99%.

MW 482.62 g mol^{-1} ($C_{25}H_{42}N_2O_7$).

R$_f$ 0.46 (EtOAc/cHex = 1:1).

$[\alpha]^{20}_{\lambda}$ (c = 0.30, $CHCl_3$): -215.3° (365 nm), -95.7° (436 nm), -55.0° (546 nm), -49.0° (579 nm), -49.7° (589 nm).

^{1}H-NMR (500 MHz, $CDCl_3$, mixture of rotamers) δ [ppm] = 4.46 – 4.39 (m, 1.0H, H-11), 3.95 – 3.91 (m, 1.5H, H-5, H-6), 3.88 – 3.80 (m, 2.0H, H-7, H-18), 3.72 – 3.65 (m, 1.5H, H-1, H-6, H-20), 3.62 – 3.52 (m, 3.0H, H-1, H-18, H-20), 3.49 – 3.39 (m, 1.0H, H-1), 2.76 – 2.58 (m, 1.0H, H-3), 2.34 – 2.29 (m, 1.0H, H-10), 2.25 – 2.19 (m, 1.0H, H-9), 2.15 – 2.06 (m, 1.0H, H-3), 1.95 – 1.89 (m, 1.0H, H-2), 1.80 – 1.63 (m, 3.0H, H-2, H-9, H-10), 1.43, 1.42 (2 x s, 18.0H, H-14, H-17), 1.23 – 1.18 (m, 6.0H, H-19, H-21).

^{13}C-NMR (125 MHz, $CDCl_3$, mixture of rotamers) δ [ppm] = 171.2 (s, C12), 170.7 (s, C12), 168.8 (s, C8), 168.6 (s, C8), 154.1 (s, C15), 80.8 (s, C13/C16), 79.5 (d, C6), 79.2 (s, C13/C16), 79.1 (d, C6), 78.5 (d, C5), 68.9 (t, C18), 68.8 (t, C18), 65.8 (t, C20), 65.6 (t, C20), 59.7 (d, C11), 58.5 (d, C7), 57.9 (d, C7), 48.6 (t, C1), 48.4 (t, C1), 34.9 (t, C3), 33.2 (t, C3), 31.1 (t, C9), 31.0 (t, C9), 28.7 (q, C14, C17), 28.5 (q, C14, C17), 28.0 (t, C10), 28.0 (q, C14, C17), 27.8 (t, C10), 23.2 (t, C2), 22.8 (t, C2), 15.7 (q, C19/C21), 15.5 (q, C19/C21).

IR $\tilde{\nu}$[cm^{-1}] = 2975 (m), 2929 (m), 2877 (w), 1738 (m), 1695 (s), 1661 (m), 1477 (w), 1447 (m), 1382 (s), 1366 (s), 1284 (w), 1256 (m), 1211 (), 1153 (s), 1117 (s), 1073 (m), 993 (w), 935 (w), 851 (w), 770 (w), 723 (w), 552 (w), 470 (w).

HRMS (ESI) calcd: m/z = 483.30648 [M+H]$^+$, 505.28842 [M+Na]$^+$, found: 483.30639 [M+H]$^+$, 505.28776 [M+Na]$^+$.

Fmoc-protected building blocks for SPPS

General procedure for the synthesis of Fmoc-ProM building blocks (Fmoc-procedure)

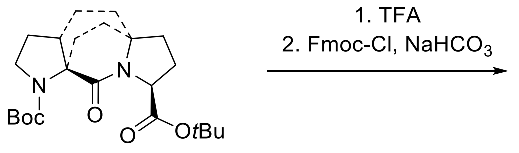

Under argon Boc-protected ProM-*tert*-butylester (100 to 500 µmol, 1.0 eq) was dissolved in 280 µl dry CH_2Cl_2 per 100 µmol starting material and the same volume of trifluoroacetic acid (280 µl per 100 µmol starting material, 36.0 eq) was added at 0 °C. The reaction mixture was stirred for 1 h at RT. After removing the volatile components in oil pump vacuum, the residue was dissolved in 0.6 ml sat. $NaHCO_3$ solution (per 100 µmol starting material) and 200 mg solid $NaHCO_3$ was added to achieve pH = 8. A solution of Fmoc-chloride (1.5 eq) in THF (concentration: 50 mg/ml) was then added and the mixture was stirred overnight. The reaction mixture was diluted with approx. 10 ml water and the THF was quickly removed at the rotary evaporator. After washing the aqueous phase with Et_2O (2 x 10 ml), it was saturated by adding Na_2SO_4 and adjusted to pH = 1-2 by adding drops of $KHSO_4$ solution so that CH_2Cl_2 (4 x 10 ml) could be used for extraction. The combined organic phases were washed with sat. NaCl solution, dried over Na_2SO_4 and the solvent was removed, whereby the Fmoc-ProM building blocks were obtained as solids (Et_2O addition promotes solidification).

Synthesis of Fmoc-ProM1-OH

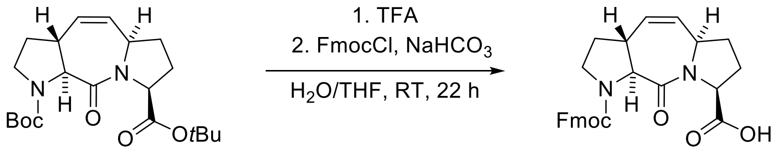

Boc-**ProM1**-O*t*Bu Fmoc-**ProM1**-OH

The reaction was carried out with Boc-**ProM1**-O*t*Bu (100 mg, 255 µmol, 1.0 eq) in accordance with the Fmoc procedure (see above), which resulted in a colorless foam after drying *in vacuo*.

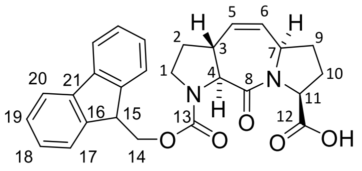

Yield 103 mg, 225 µmol, 88% (Lit[1]: 86%).

MW 458.51 g mol^{-1} ($C_{27}H_{26}N_2O_5$).

R$_f$ 0.43 (CH_2Cl_2/EtOH/HOAc = 9:1:0.05).

Smp 138 - 143 °C.

$[\alpha]^{20}_{\lambda}$ (c = 0.09, MeOH): -311.8° (436 nm), -188.2° (546 nm), -167.1° (579 nm), -163.5° (589 nm).

^{1}H-NMR (500 MHz, $CDCl_3$, mixture of rotamers) δ [ppm] = 8.60 (br. s., 1.0H, CO_2H), 7.79 - 7.68 (m, 2.0H, H-20), 7.64 - 7.52 (m, 2.0H, H-17), 7.38 (q, *J* = 7.6 Hz, 2.0H, H-19), 7.29 (q, *J* = 7.6 Hz, 2.0H, H-18), 5.77 (d, *J* = 11.0 Hz, 0.6H, H-5), 5.69 (d, *J* = 11.2 Hz, 0.4H, H-5), 5.53 (d, *J* = 11.0 Hz, 0.6H, H-6), 5.44 (d, *J* = 11.2 Hz, 0.4H, H-6), 4.77 (d, *J* = 8.2 Hz, 0.6H, H-11), 4.69 - 4.51 (m, 2.0H, H-7, H-14), 4.44 - 4.38 (m, 1.0H, H-4, H-11), 4.32 - 4.23 (m, 1.6H, H-7, H-14, H-15), 4.17 – 4.14 (m, 0.4H, H-15), 3.95 (d, *J* = 10.7 Hz, 0.4H, H-4), 3.79 - 3.71 (m, 1.0H, H-1), 3.50 (td, *J* = 11.1, 5.4 Hz, 0.6H, H-1), 3.32 (td, *J* = 11.1, 5.4 Hz, 0.4H, H-1), 2.94 (br. s., 0.6H, H-3), 2.85 (br. s., 0.4H, H-3), 2.33 - 2.21 (m, 2.0H, H-2, H-10), 2.13 (dt, *J* = 11.6, 5.8 Hz, 0.6H, H-9), 2.03 - 1.81 (m, 2.4H, H-2, H-9, H-10), 1.72 - 1.61 (m, 0.6H, H-9), 1.50 - 1.39 (m, 0.4H, H-9).

**^{13}C-NMR**	(75 MHz, $CDCl_3$, mixture of rotamers) δ [ppm] = 172.9 (s, C8/12), 172.2 (s, C8/12), 171.9 (s, C8/12), 155.3 (s, C13), 154.9 (s, C13), 144.7, 144.3, 143.8, 141.4, 141.3 (s, C16, C21), 128.9 (d, C5/6), 128.5 (d, C5/6), 127.8, 127.8, 127.7, 127.6, 127.2, 127.1, 127.0, 125.3, 125.1, 125.0, 124.9, 120.0, 119.8, 119.7 (d, C17, C18, C19, C20), 67.8 (t, C14), 66.2 (t, C14), 62.2 (d, C4), 61.9 (d, C4), 60.8 (d, C11), 60.4 (d, C11), 58.2 (d, C7), 58.0 (d, C7), 47.7 (d, C15), 47.3 (d, C15), 47.2 (t, C1), 46.9 (t, C1), 42.2 (d, C3), 41.4 (d, C3), 33.4 (t, C9), 33.3 (t, C9), 31.4 (t, C2), 30.7 (t, C2), 25.9 (t, C10), 25.8 (t, C10).
**IR**	$\tilde{\nu}$[cm^{-1}] = 3670 (w), 3423 (w), 3067 (w), 2971 (w), 2896 (w), 2886 (w), 2604 (w), 2516 (w), 1737 (m), 1702 (s), 1682 (s), 1450 (s), 1421 (s), 1351 (s), 1331 (m), 1242 (w), 1222 (w), 1190 (m), 1171 (m), 1122 (m), 1091 (w), 1073 (w), 1046 (w), 1022 (w), 985 (w), 942 (w), 897 (w), 863 (w), 838 (w), 760 (m), 740 (m), 631 (w), 621 (w).
	Analytical data was in agreement with the literature.[1]

Synthesis of Fmoc-ProM2-OH

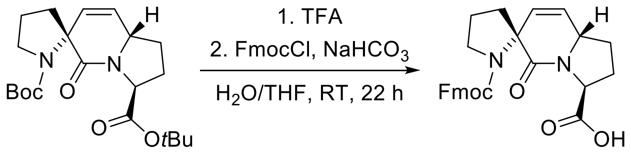

Following the Fmoc procedure (see above), Boc-**ProM2**-O*t*Bu (182 mg, 464 µmol, 1.0 eq) was re-protected, obtaining the product as a yellowish solid.

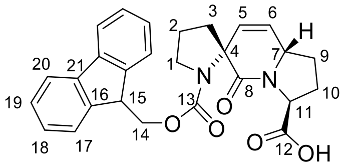

**Yield**	177 mg, 386 µmol, 83% (Lit[2]: 97%).
**MW**	458.51 g mol^{-1} ($C_{27}H_{26}N_2O_5$).
**R$_f$**	0.27 (CH_2Cl_2/EtOH/HOAc = 9:1:0.05).
**Smp**	97 - 99 °C.
**[α]$^{20}_{\lambda}$**	(c = 0.26, MeOH): -223.5° (436 nm), -128.8° (546 nm), -116.9° (579 nm), -116.9° (589 nm).
**^{1}H-NMR**	(400 MHz, $CDCl_3$, mixture of rotamers) δ [ppm] = 8.02 (br. s., 0.5H, CO_2H), 7.77 – 7.69, 7.63 – 7.52, 7.41 – 7.35, 7.32 – 7.27 (4 x m, 4 x 2.0H, H-17, H-18, H-19, H-20), 5.89 (d, *J* = 1.7 Hz, 0.3H, H-5), 5.86 (d, *J* = 1.7 Hz, 0.5H, H-5), 5.79 (d, *J* = 2.5 Hz, 0.4H, H-6), 5.76 (d, *J* = 2.5 Hz, 0.3H, H-6), 5.50 – 5.43 (m, 0.4H, H-5, H-6), 4.72 (t, *J* = 8.8 Hz, 0.7H, H-11), 4.61 (dd, *J* = 11.2 Hz, 5.5 Hz, 0.2H, H-14), 4.48 (dd, *J* = 11.2 Hz, 5.5 Hz, 0.2H, H-14), 4.42 – 4.35 (m, 0.8H, H-14), 4.30 – 4.22 (m, 1.8H, H-11, H-14, H-15), 4.20 – 4.14 (m, 0.8H, H-7), 4.08 (t, *J* = 5.5 Hz, 0.2H, H-15), 4.02 – 3.98 (m, 0.2H, H-7), 3.86 – 3.76 (m, 1.0H, H-1), 3.68 – 3.53 (m, 1.0H, H-1), 2.41 – 2.24 (m, 2.2H, H-3, H-10), 2.23 – 2.05 (m, 2.3H, H-2, H-3, H-9, H-10), 2.04 – 1.91 (m, 2.0H, H-2, H-3, H-9), 1.91 – 1.69 (m, 1.2H, H-2, H-3, H-9), 1.21 – 1.07 (m, 0.2H, H-9).
**^{13}C-NMR**	(100 MHz, $CDCl_3$, mixture of rotamers) δ [ppm] = 172.5 (s, C12), 172.3 (s, C12), 171.7 (s, C8), 171.0 (s, C8), 154.9 (s, C13), 154.8 (s, C13), 144.4, 144.2, 143.9, 141.4, 141.4, 141.3 (s, C16, C21), 131.9 (d, C6), 130.4 (d, C6), 127.8, 127.8, 127.6, 127.6, 127.1, 127.1, 127.1, 127.0, 125.3, 125.2, 124.9, 124.9 (d, C17, C18, C19, C20), 122.9 (d, C5), 122.3 (d, C5), 120.0, 119.9 (d, C17, C18, C19, C20), 67.5 (t, C14), 66.3 (t, C14), 64.1 (s, C4), 63.9 (s, C4), 59.7 (d, C7), 59.1 (d, C7), 59.0 (d, C11), 58.4 (d, C11), 49.0 (t, C1), 48.1 (t, C1), 47.6 (d, C15), 47.3 (d, C15), 40.1 (t, C3), 39.1 (t, C3), 31.0 (t, C9), 30.7 (t, C9), 26.9 (t, C10), 26.7 (t, C10), 23.6 (t, C2), 22.5 (t, C2).
**IR**	$\tilde{\nu}$[cm^{-1}] = 3425 (w), 3066 (w), 3040 (w), 2973 (m), 2957 (m), 2900 (m), 2879 (m), 1739 (m), 1697 (s), 1684 (s), 1449 (s), 1414 (s), 1353 (m), 1343 (m), 1307 (w), 1219 (m), 1195 (m), 1154 (m), 1139 (m), 1038 (w), 1009 (w), 942 (w), 877 (w), 760 (m), 741 (m), 668 (w), 621 (w).
	Analytical data was in agreement with the literature.[2]

Synthesis of Fmoc-H_2-ProM1-OH

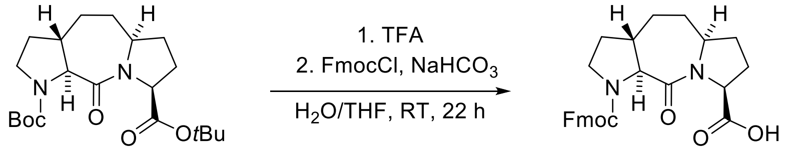

Boc-**H_2-ProM1**-O*t*Bu Fmoc-**H_2-ProM1**-OH

According to the Fmoc-procedure (see above), Boc-H_2-ProM1-O*t*Bu (97 mg, 246 µmol, 1.0 eq) was transformed to the corresponding Fmoc-derivative, which was isolated as a colorless solid.

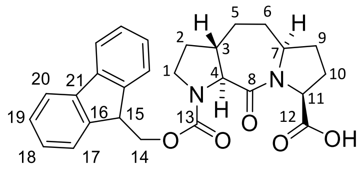

Yield 103 mg, 224 µmol, 91%.

MW 460.53 g mol^{-1} ($C_{27}H_{28}N_2O_5$).

R_f 0.37 (CH_2Cl_2/EtOH/HOAc = 9:1:0.05).

Smp 132 - 136 °C.

$[\alpha]^{20}_{\lambda}$ (c = 0.11, MeOH): -244.5° (436 nm), -145.5° (546 nm), -129.1° (579 nm), -127.3° (589 nm).

^{1}H-NMR (600 MHz, $CDCl_3$, mixture of rotamers) δ [ppm] = 9.80 (br. s., 0.5H, CO_2H), 7.76 – 7.72, 7.65 – 7.52, 7.40 – 7.36, 7.31 – 7.27 (4 x m, 4 x 2.0H, H-17, H-18, H-19, H-20), 4.73 (d, *J* = 7.6 Hz, 0.6H, H-11), 4.62 (dd, *J* = 11.0 Hz, 5.2 Hz, 0.4H, H-14), 4.55 – 4.50 (m, 1.0H, H-14), 4.38 (d, *J* = 6.9 Hz, 0.4H, H-11), 4.30 – 4.26 (m, 1.2H, H-14, H-15), 4.19 (d, *J* = 9.8 Hz, 0.6H, H-4), 4.15 (t, *J* = 5.2 Hz, 0.4H, H-15), 3.94 (q, *J* = 9.2 Hz, 0.6H, H-7), 3.78 (q, *J* = 8.2 Hz, 1.0H, H-1), 3.73 (d, *J* = 9.8 Hz, 0.4H, H-4), 3.67 – 3.63 (m, 0.4H, H-7), 3.43 (dt, *J* = 11.2 Hz, 5.5 Hz, 0.6H, H-1), 3.25 (dt, *J* = 11.2 Hz, 5.3 Hz, 0.4H, H-1), 2.28 – 2.17 (m, 2.0H, H-5, H-10), 2.14 – 2.08 (m, 1.2H, H-3, H-2/H-9), 2.02 – 1.97 (m, 1.1H, H-3, H-2/H-9), 1.96 – 1.92 (m, 0.6H, H-10), 1.91 – 1.73 (m, 2.8H, H-5, H-6, H-10, H-2/H-9), 1.63 – 1.50 (m, 2.3H, H-6, H-2/H-9), 1.42 – 1.36 (m, 0.5H, H-2/H-9), 1.34 – 1.28 (m, 0.5H, H-2/H-9).

^{13}C-NMR (150 MHz, $CDCl_3$, mixture of rotamers) δ [ppm] = 173.6 (s, C12), 173.5 (s, C12), 172.5 (s, C8), 172.4 (s, C8), 155.2 (s, C13), 154.8 (s, C13), 144.9, 144.4, 143.8, 143.6, 141.4, 141.3 (s, C16, C21), 127.8, 127.7, 127.7, 127.5, 127.2, 127.1, 127.0, 125.4, 125.2, 125.1, 125.0, 120.0, 120.0, 119.8, 119.7 (d, C17, C18, C19, C20), 67.7 (t, C14), 66.3 (t, C14), 64.5 (d, C4), 64.1 (d, C4), 61.1 (d, C11), 60.7 (d, C11), 58.9 (d, C7), 58.8 (d, C7), 47.8 (d, C15), 47.4 (d, C15), 47.1 (t, C1), 46.9 (t, C1), 42.7 (d, C3), 41.8 (d, C3), 34.8 (t, C6), 34.7 (t, C6), 33.5 (t, C5), 33.4 (t, C5), 33.0 (t, C2/9), 33.0 (t, C2/9), 32.9 (t, C2/9), 32.3 (t, C2/9), 26.4 (t, C10), 26.3 (t, C10).

IR $\tilde{\nu}$[cm^{-1}] = 3440 (w), 3063 (w), 3040 (w), 2965 (m), 2923 (m), 2883 (m), 2721 (w), 2594 (w), 1738 (w), 1703 (s), 1671 (s), 1450 (s), 1421 (s), 1353 (m), 1330 (m), 1283 (w), 1251 (w), 1209 (m), 1180 (m), 1123 (m), 1066 (w), 1048 (w), 986 (w), 926 (w), 891 (w), 816 (w), 760 (m), 741 (m), 686 (w), 621 (w).

HRMS (ESI) calcd: m/z = 461.20710 $[M+H]^+$, 483.18904 $[M+Na]^+$, found: 461.20727 $[M+H]^+$, 483.18899 $[M+Na]^+$.

Synthesis of Fmoc-H_2-ProM2-OH

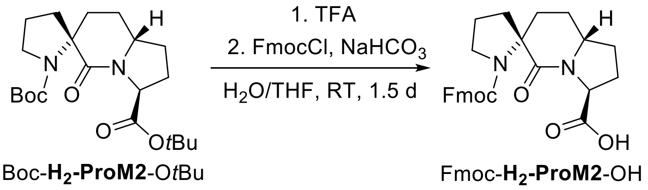

By following the Fmoc-protocol (see above), Boc-**H_2-ProM2**-O*t*Bu (106 mg, 269 µmol, 1.0 eq) was re-protected resulting in the Fmoc-derivative as a colorless solid.

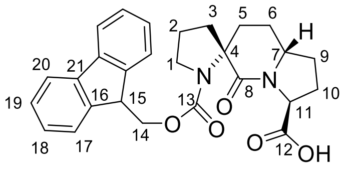

Yield 120 mg, 261 µmol, 97%.

MW 460.53 g mol^{-1} ($C_{27}H_{28}N_2O_5$).

R_f 0.29 (CH_2Cl_2/EtOH/HOAc = 9:1:0.05).

Smp 87 - 89 °C.

$[\alpha]^{20}_{\lambda}$ (c = 0.24, MeOH): -106.7° (365 nm), -87.1° (436 nm), -57.5° (546 nm), -55.0° (579 nm), -58.3° (589 nm).

^{1}H-NMR (400 MHz, $CDCl_3$, mixture of rotamers) δ [ppm] = 9.00 (br. s., 0.7H, OH), 7.76 – 7.71, 7.63 – 7.57, 7.40 – 7.35, 7.32 – 7.28 (4 x m, 4 x 2.0H, H-17, H-18, H-19, H-20), 4.79 – 4.71 (m, 0.2H, H-14), 4.67 (t, *J* = 8.5 Hz, 0.8H, H-11), 4.40 (dd, *J* = 13.2 Hz, 10.0 Hz, 0.8H, H-14), 4.26 – 4.21 (m, 1.7H, H-14, H-15), 4.17 – 4.14 (m, 0.2H, H-15), 3.99 (t, *J* = 8.2 Hz, 0.2H, H-11), 3.76 – 3.69 (m, 0.8H, H-1), 3.64 – 3.55 (m, 1.8H, H-1, H-7), 3.52 – 3.45 (m, 0.2H, H-1), 3.38 – 3.28 (m, 0.2H, H-7), 2.44 – 2.25 (m, 2.6H, H-3, H-5, H-9/10), 2.20 – 2.00 (m, 3.9H, H-2, H-6, H-9/10), 1.95 – 1.80 (m, 4.0H, H-2, H-3, H-5, H-9/10), 1.73 – 1.62 (m, 1.1H, H-6), 1.56 – 1.49 (m, 0.3H, H-5, H-9/10), 1.15 – 1.09 (m, 0.2H, H-6).

^{13}C-NMR (100 MHz, $CDCl_3$, mixture of rotamers) δ [ppm] = 173.4 (s, C8), 172.8 (s, C12), 155.1 (s, C13), 144.3, 143.8, 141.4, 141.4 (s, C16, C21), 127.2, 127.2, 127.1, 127.1, 125.4, 125.2, 124.7, 124.6, 120.0, 119.9, 119.8 (d, C17, C18, C19, C20), 67.4 (t, C14), 65.3 (t, C14), 64.3 (s, C4), 63.8 (s, C4), 60.1 (d, C11), 60.1 (d, C7), 59.2 (d, C11), 58.1 (d, C7), 49.0 (t, C1), 48.2 (t, C1), 47.6 (d, C15), 47.3 (d, C15), 42.4 (t, C3), 40.9 (t, C3), 35.5 (t, C5), 34.5 (t, C5), 32.4 (t, C6), 32.2 (t, C6), 26.9 (t, C9/10), 26.7 (t, C9/10), 26.6 (t, C9/10), 23.5 (t, C2), 22.3 (t, C2).

IR $\tilde{v}$ [cm^{-1}] = 3415 (w), 3066 (w), 3037 (w), 2970 (m), 2934 (w), 2877 (w), 1738 (m), 1698 (s), 1651 (m), 1449 (m), 1411 (s), 1351 (m), 1335 (m), 1192 (m), 1161 (m), 1125 (m), 1084 (m), 1047 (m), 1005 (m), 945 (w), 877 (w), 759 (m), 740 (s), 636 (w), 621 (w), 543 (w).

HRMS (ESI) calcd: m/z = 461.20710 $[M+H]^+$, 483.18904 $[M+Na]^+$, found: 461.20722 $[M+H]^+$, 483.18915 $[M+Na]^+$.

Synthesis of Fmoc-epoxy-ProM1-OH

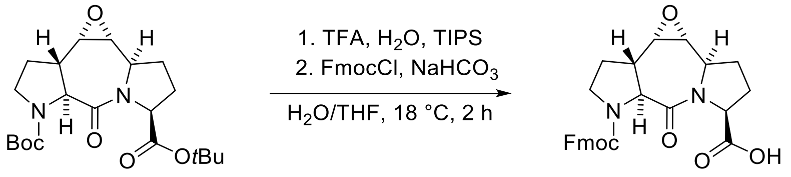

Boc-**ep-ProM1**-O*t*Bu Fmoc-**ep-ProM1**-OH

Under Argon Boc-**ep-ProM1**-O*t*Bu (109 mg, 267 µmol, 1.0 eq) was dissolved in 1.3 ml dry CH_2Cl_2 and successively mixed with water (32 µl, 1.8 mmol, 6.7 eq), triisopropylsilane (32 µl, 156 µmol, 0.6 eq) and trifluoroacetic acid (1.24 ml, 16.1 mmol, 60 eq) at 0 °C. The reaction mixture was stirred at this temperature for 5 min and then for 1 h at 15 °C. After removal of the volatile components *in vacuo*, 500 mg $NaHCO_3$ and 2.0 ml sat. $NaHCO_3$ solution were added. At 18 °C, Fmoc chloride (100 mg, 387 µmol, 1.4 eq) in 3.0 ml THF was added to the reaction mixture, which was stirred for 2 h at the same temperature. It was diluted with 8.0 ml water and the THF was rapidly removed at the rotary evaporator. The aqueous solution was washed with Et_2O (3 x 10 ml), saturated with Na_2SO_4 and treated with $KHSO_4$ solution to adjust the pH to 1-2. Extraction was performed with CH_2Cl_2 (3 x 20 ml). Washing the combined organic phases with semi-sat. NaCl solution, drying over Na_2SO_4 and solvent removal resulted in a colorless foamy solid.

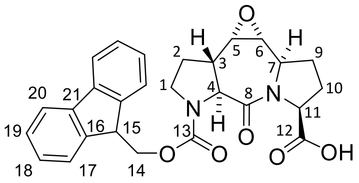

Yield 82 mg, 173 µmol, 65%.

MW 474.51 g mol^{-1} ($C_{27}H_{26}N_2O_6$).

R$_f$ 0.20 (CH_2Cl_2/EtOH/HOAc = 9:1:0.05).

Smp 143 - 146 °C.

$[\alpha]^{20}_{\lambda}$ (c = 0.14, MeOH): -268.6° (365 nm), -182.9° (436 nm), -112.9° (546 nm), -106.4° (579 nm), -111.4° (589 nm).

^{1}H-NMR (500 MHz, $CDCl_3$, mixture of rotamers) δ [ppm] = 9.93 (br. s., 0.9H, OH), 7.77 – 7.73, 7.63 – 7.49, 7.39 – 7.35, 7.32 – 7.27 (4 x m, 4 x 2.0H, H-17, H-18, H-19, H-20), 4.68 (d, *J* = 5.5 Hz, 0.5H, H-11), 4.59 – 4.47 (m, 1.5H, H-14), 4.39 – 4.37 (m, 0.4H, H-11), 4.29 – 4.24 (m, 1.6H, H-7, H-14, H-15), 4.13 – 4.07 (m, 1.0H, H-4, H-15), 3.82 – 3.73 (m, 1.5H, H-1, H-7), 3.65 (d, *J* = 11.7 Hz, 0.4H, H-4), 3.53 (dt, *J* = 11.0 Hz, 5.8 Hz, 0.5H, H-1), 3.34 (dt, *J* = 11.0 Hz, 5.8 Hz, 0.4H, H-1), 3.28 (d, *J* = 4.3 Hz, 0.5H, H-5), 3.20 (d, *J* = 4.3 Hz, 0.4H, H-5), 3.07 (dd, *J* = 4.3 Hz, 2.4 Hz, 0.5H, H-6), 2.98 (dd, *J* = 4.3 Hz, 1.8 Hz, 0.4 Hz, H-6), 2.67 (dt, *J* = 11.7 Hz, 5.8 Hz, 0.6H, H-3), 2.58 (dt, *J* = 11.7 Hz, 6.3 Hz, 0.4H, H-3), 2.53 – 2.41 (m, 1.0H, H-9), 2.23 – 1.93 (m, 4.5H, H-2, H-9, H-10), 1.76 – 1.67 (m, 0.4H, H-2), 1.43 (d, *J* = 6.3 Hz, 0.1H, H-2).

^{13}C-NMR (125 MHz, $CDCl_3$, mixture of rotamers) δ [ppm] = 173.0 (s, C12), 172.9 (s, C12), 171.5 (s, C8), 155.4 (s, C13), 154.9 (s, C13), 144.6, 144.3, 143.8, 143.5, 141.4, 141.4 (s, C16, C21), 127.9, 127.8, 127.8, 127.5, 127.2, 127.2, 127.1, 127.0, 125.3, 125.2, 125.1, 124.9, 120.2, 120.1, 120.0, 119.9 (d, C17, C18, C19, C20), 67.9 (t, C14), 66.3 (t, C14), 60.1 (d, C11), 59.7 (d, C11), 58.6 (d, C7), 58.3 (d, C7), 57.8 (d, C6), 57.6 (d, C6), 57.0 (d, C4), 56.5 (d, C4), 55.6 (d, C5), 55.6 (d, C5), 47.8 (d, C15), 47.5 (t, C1), 47.3 (d, C15), 47.2 (t, C1), 42.9 (d, C3), 42.1 (d, C3), 33.0 (t, C9), 32.8 (t, C9), 29.1 (t, C2), 28.5 (t, C2), 27.0 (t, C10), 27.0 (t, C10).

IR $\tilde{v}$[cm^{-1}] = 3666 (w), 3420 (w), 2967 (m), 2924 (m), 2898 (m), 1703 (s), 1702 (s), 1684 (s), 1446 (s), 1420 (s), 1353 (s), 1276 (m), 1230 (m), 1174 (m), 1121 (m), 1069 (w), 986 (w), 833 (w), 761 (m), 741 (m).

HRMS (ESI) calcd: m/z = 475.18636 [M+H]$^+$, 497.16831 [M+Na]$^+$, found: 475.18645 [M+H]$^+$, 497.16843 [M+Na]$^+$.

Synthesis of Fmoc-(TBSO)-ProM1-OH

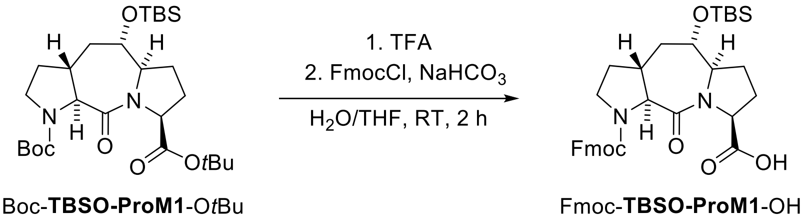

Boc-**TBSO-ProM1**-O*t*Bu (40 mg, 76.2 μmol, 1.0 eq) was dissolved under argon in 0.25 ml CH_2Cl_2 and mixed with TFA (0.25 ml, 0.325 mmol, 42.6 eq) at 0 °C. After 1 h stirring at RT, the volatile components were removed *in vacuo* and 0.7 ml sat. $NaHCO_3$ solution and 200 mg solid $NaHCO_3$ were added. A solution of Fmoc chloride (24 mg, 91.4 μmol, 1.2 eq) in 0.75 ml THF was added and the mixture stirred for 2 h at RT. The reaction mixture was then diluted with 5.0 ml water, washed with Et_2O (3 x 10 ml), acidified to pH = 2 with 10 wt% $KHSO_4$ solution and extracted with CH_2Cl_2 (4 x 15 ml). The combined organic phases were washed with 10 ml sat. NaCl solution, dried over Na_2SO_4 and the solvent was removed. The crude product was purified by column chromatography on ultrapure SiO_2 (CH_2Cl_2/MeOH = 50:1 to 20:1) to give a colorless oil which solidified upon storage.

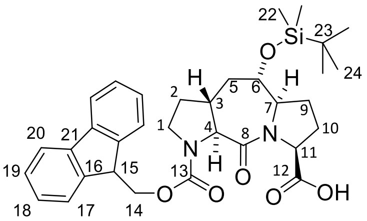

Yield 19 mg, 32.2 μmol, 42%.

MW 590.79 g mol^{-1} ($C_{33}H_{42}N_2O_6Si$).

R_f 0.57 (CH_2Cl_2/EtOH/HOAc = 9:1:0.05).

Smp 108 - 112 °C.

$[\alpha]^{20}_{\lambda}$ (c = 0.10, MeOH): -128.0° (365 nm), -85.0° (436 nm), -52.0° (546 nm), -47.3° (579 nm), -49.7° (589 nm).

^{1}H-NMR (600 MHz, $CDCl_3$, mixture of rotamers) δ [ppm] = 7.76 – 7.73, 7.64 – 7.52, 7.41 – 7.37, 7.32 – 7.29 (4 x m, 4 x 2.0H, H-17, H-18, H-19, H-20), 4.74 (dd, *J* = 5.3 Hz, 2.7 Hz, 0.6H, H-11), 4.60 (d, *J* = 5.7 Hz, 0.8H, H-14), 4.53 (dd, *J* = 9.7 Hz, 5.7 Hz, 0.6H, H-14), 4.41 (dd, *J* = 8.0 Hz, 2.9 Hz, 0.4H, H-11), 4.31 – 4.22 (m, 1.8H, H-4, H-14, H-15), 4.15 (t, *J* = 5.7 Hz, 0.4H, H-15), 3.89 (q, *J* = 8.6 Hz, 0.5H, H-4), 3.80 – 3.73 (m, 1.4H, H-1, H-7), 3.60 – 3.53 (m, 1.0H, H-6, H-7), 3.49 – 3.40 (m, 1.0H, H-1, H-6), 3.22 (dt, *J* = 11.2 Hz, 5.3 Hz, 0.4H, H-1), 2.30 – 2.19 (m, 3.0H, H-3, H-5, H-9, H-10), 2.13 – 1.83 (m, 4.0H, H-2, H-9, H-10), 1.64 – 1.59 (m, 1.0H, H-2, H-5), 1.50 – 1.44 (m, 0.5H, H-5), 1.37 – 1.31 (m, 0.5H, H-2), 0.91, 0.88 (2 x s, 9.0H, H-24), 0.07 (s, 6.0H, H-22).

^{13}C-NMR (150 MHz, $CDCl_3$, mixture of rotamers) δ [ppm] = 173.3 (s, C12), 172.3 (s, C8), 155.1 (s, C13), 154.8 (s, C13), 144.9, 144.5, 143.9, 143.7, 141.5, 141.4 (s, C16, C21), 127.8, 127.8, 127.6, 127.2, 127.2, 127.1, 125.4, 125.2, 125.2, 125.0, 120.1, 120.1, 119.9 (d, C17, C18, C19, C20), 73.2 (d, C6), 72.9 (d, C6), 67.8 (t, C14), 66.3 (t, C14), 64.0 (d, C4), 63.8 (d, C4), 63.7 (d, C7), 63.5 (d, C7), 61.4 (d, C11), 61.0 (d, C11), 47.9 (d, C15), 47.4 (d, C15), 46.9 (t, C1), 46.8 (t, C1), 43.0 (t, C5), 40.9 (d, C3), 40.1 (d, C3), 33.1 (t, C2), 32.4 (t, C2), 31.0 (t, C9), 30.8 (t, C9), 26.0 (t, C10), 25.9 (q, C24), 18.1 (s, C23), -3.8 (q, C22), -4.2 (q, C22), -4.6 (q, C22).

IR $\tilde{\nu}$[cm^{-1}] = 3675 (w), 2988 (m), 2971 (m), 2927 (m), 2901 (m), 1709 (w), 1685 (w), 1450 (w), 1408 (m), 1394 (w), 1382 (w), 1355 (w), 1250 (w), 1230 (w), 1183 (w), 1162 (w), 1074 (s), 1066 (s), 1057 (s), 892 (w), 866 (w), 837 (w), 776 (w), 761 (w), 740 (w), 419 (w), 407 (w).

HRMS (ESI) calcd: m/z = 591.28849 [M+H]$^+$, 613.27043 [M+Na]$^+$, found: 591.28884 [M+H]$^+$, 613.27070 [M+Na]$^+$.

Synthesis of Fmoc-(Me$_2$CO$_2$)-ProM1-OH

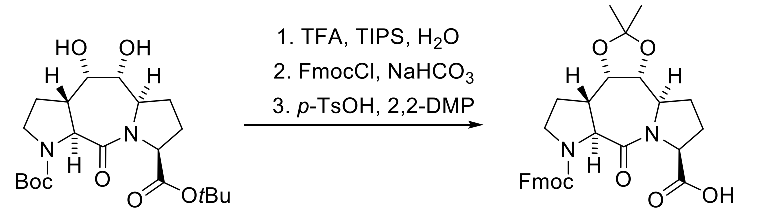

Boc-**(HO)$_2$-ProM1**-O*t*Bu   Fmoc-**(Me$_2$CO$_2$)-ProM1**-OH

Under argon, Boc-**(HO)$_2$-ProM1**-O*t*Bu (109 mg, 256 µmol, 1.0 eq) was dissolved in 1.4 ml CH_2Cl_2 and treated at 0 °C with water (33 µl, 1.82 mmol, 7.1 eq), triisopropylsilane (33 µl, 160 µmol, 0.6 eq) and finally with TFA (1.3 ml, 17.0 mmol, 67 eq). The reaction mixture was stirred for 1 h at RT and the volatile components were removed in oil pump vacuum. The residue was taken up together with 500 mg $NaHCO_3$ in 2.0 ml sat. $NaHCO_3$ solution (pH = 8), to which a solution of FmocCl (98 mg, 379 µmol, 1.5 eq) in 5.4 ml THF was added. After stirring overnight and diluting with H_2O, the THF was rapidly removed at the rotary evaporator. The aqueous phase was washed with Et_2O (2 x 10 ml) and saturated with Na_2SO_4. Extraction of the aqueous phase (adjusted to pH = 1 by addition of 10-w% KHSO4 solution) was performed with CH_2Cl_2 (5 x 10 ml) and the combined organic phases were washed with sat. NaCl solution and dried over Na2SO4, resulting in a cololess solid after solvent removal.

This solid was dissolved in 5.4 ml dry CH_2Cl_2/acetone (1:1) under argon and treated with 2,2-dimethoxypropane (2.7 ml, 22.0 mmol, 86 eq) and *para*-toluenesulfonic acid (43 mg, 250 µmol, 1.0 eq). After 6 h stirring at RT, the volatile components were removed at the rotary evaporator and the residue was dissolved in 10 ml of semi-sat. $NaHCO_3$ solution. At 0 °C the aqueous phase was washed with Et_2O (2 x 10 ml), saturated with Na_2SO_4 and adjusted to pH = 2 by addition of 10 wt% $KHSO_4$ solution. After extraction with CH_2Cl_2 (5 x 10 ml), the combined organic phases were washed (sat. NaCl-Lsg), dried (over Na_2SO_4) and the solvent removed to obtain a yellowish solid.

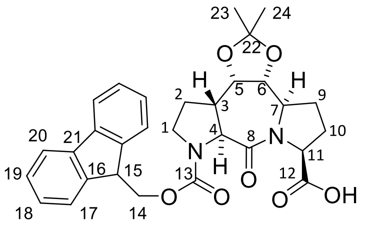

ield 74 mg, 139 µmol, 54%.

MW 532.59 g mol^{-1} ($C_{30}H_{32}N_2O_7$).

R$_f$ 0.27 (CH_2Cl_2/EtOH/HOAc = 9:1:0.05).

Smp 123 - 126 °C.

[α]$^{20}_{\lambda}$ (c = 0.26, MeOH): -181.2° (365 nm), -108.1° (436 nm), -66.2° (546 nm), -61.9° (579 nm), -64.2° (589 nm).

^{1}H-NMR (500 MHz, $CDCl_3$, mixture of rotamers) δ [ppm] = 7.77 – 7.71, 7.64 – 7.52, 7.41 – 7.36, 7.32 – 7.28 (4 x m, 4 x 2.0H, H-17, H-18, H-19, H-20), 4.76 (d, *J* = 10.4 Hz, 0.6H, H-4), 4.61 – 4.50 (m, 1.6H, H-11, H-14), 4.44 – 4.39 (m, 1.5H, H-4, H-5, H-14), 4.35 – 4.28 (m, 1.0H, H-5, H-11), 4.27 – 4.23 (m, 1.1H, H-14, H-15), 4.18 (t, *J* = 5.8 Hz, 0.4H, H-15), 4.14 – 4.09 (m, 0.6H, H-7), 3.90 (dd, *J* = 10.4 Hz, 4.6 Hz, 0.6H, H-6), 3.86 – 3.75 (m, 1.8H, H-1, H-6, H-7), 3.48 (dt, *J* = 11.3 Hz, 5.8 Hz, 0.6H, H-1), 3.32 (dt, *J* = 11.3 Hz, 5.8 Hz, 0.4H, H-1), 2.68 – 2.56 (m, 1.0H, H-3), 2.28 – 1.98 (m, 5.0H, H-2, H-9, H-10), 1.95 – 1.90 (m, 0.6H, H-2), 1.86 – 1.81 (m, 0.4H, H-2), 1.48 (s, 3.0H, H-23/24), 1.37, 1.36 (2x s, 3.0H, H-23/24).

^{13}C-NMR (150 MHz, $CDCl_3$, mixture of rotamers) δ [ppm] = 174.7 (s, C12), 174.4 (s, C12), 171.2 (s, C8), 171.2 (s, C8), 155.4 (s, C13), 154.8 (s, C13), 144.7, 144.4, 144.0, 143.9, 141.4, 141.4, 141.4 (s, C16, C21), 127.8, 127.8, 127.7, 127.6, 127.2, 127.2, 127.2, 127.0, 125.4, 125.2, 125.1, 125.0, 120.1, 119.8 (d, C17, C18, C19, C20), 108.9 (s, C22), 108.9 (s, C22), 77.4 (d, C6), 77.0 (d, C6), 75.2 (d, C5), 67.8 (t, C14), 66.6 (t, C14), 60.1 (d, C11), 59.8 (d, C11), 57.3 (d, C7), 57.1 (d, C4), 56.7 (d, C4), 47.5 (d, C15), 47.4 (d, C15), 46.7 (t, C1), 46.6 (t, C1), 42.9 (d, C3), 42.1 (d, C3), 29.3 (t, C9), 29.1 (t, C9), 28.9 (q, C23/24), 28.8 (q, C23/24), 28.8 (t, C2), 28.2 (t, C2), 26.8 (t, C10), 26.7 (t, C10), 26.3 (q, C23/24), 26.3 (q, C23/24).

**IR**	$\tilde{\nu}$[cm^{-1}] = 3407 (w), 3066 (w), 3040 (w), 2975 (w), 2952 (w), 2928 (w), 2882 (w), 1684 (s), 1449 (m), 1422 (s), 1381 (m), 1356 (m), 1273 (w), 1245 (m), 1221 (s), 1184 (s), 1123 (m), 1081 (m), 1035 (m), 992 (w), 919 (w), 867 (m), 768 (w), 760 (m), 740 (m), 685 (w), 621 (w), 545 (w), 510 (w).
**HRMS (ESI)**	calcd: m/z = 533.22823 $[M+H]^+$, 555.21017 $[M+Na]^+$, found: 533.22836 $[M+H]^+$, 555.21032 $[M+Na]^+$.

Synthesis of Fmoc-(Me$_2$CO$_2$)-ProM2-OH

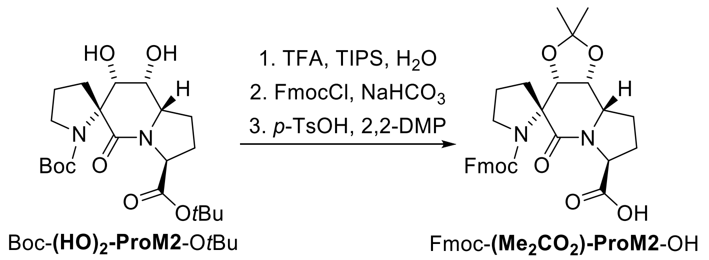

Under argon, Boc-**(HO)$_2$-ProM2**-O*t*Bu (20 mg, 47 µmol, 1.0 eq) was dissolved in 252 µl dry CH_2Cl_2 and mixed at 0 °C with water (6 µl, 333 µmol, 7.1 eq), triisopropylsilane (6 µl, 89 µmol, 0.6 eq) and trifluoroacetic acid (240 µl, 3.1 mmol, 66.4 eq). After 60 min reaction time at RT, the volatile components of the mixture were removed *in vacuo* and the residue was dissolved in 0.5 ml sat. $NaHCO_3$ solution. Solid $NaHCO_3$ (100 mg) and a solution of Fmoc chloride (18 mg, 70 µmol, 1.5 eq) in 1.0 ml THF were added, followed by stirring for 2.5 h at RT. After removal of the THF at the rotary evaporator, the reaction mixture was diluted with 5 ml water, washed with Et_2O (10 ml) and saturated with Na_2SO_4. The pH value was adjusted to 1 by adding drops of $KHSO_4$ solution (10 wt%) so that CH_2Cl_2 (2 x 10 ml) could be used for extraction. The combined organic phases were washed with 10 ml sat. NaCl solution, dried over Na_2SO_4 and the solvent was removed resulting in a yellow oil.

This oil was dissolved together with *para*-toluenesulfonic acid (8 mg, 47 µmol, 1.0 eq) in 3.0 ml 2,2-dimethoxypropane /acetone/CH_2Cl_2 (1:1:1) and the mixture was stirred for 1 h at RT. After removal of the solvent at the rotary evaporator, the residue was dissolved in 6 ml sat. $NaHCO_3$ solution and the above described extraction procedure was repeated. A light yellow solid was obtained.

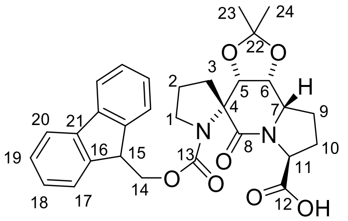

**Yield**	12 mg, 23 µmol, 48%.
**MW**	532.59 g mol^{-1} ($C_{30}H_{32}N_2O_7$).
**R$_f$**	0.31 (CH_2Cl_2/EtOH/HOAc = 9:1:0.05).
**Smp**	185 - 190 °C.
**$[\alpha]^{20}_{\lambda}$**	(c = 0.21, MeOH): +13.8° (365 nm), -21.0° (436 nm), -21.4° (546 nm), -24.8° (579 nm), -30.0° (589 nm).
**^{1}H-NMR**	(500 MHz, $CDCl_3$, mixture of rotamers) δ [ppm] = 7.75 (dd, *J* = 6.6 Hz, 2.0H, H-17/18/19/20), 7.67 – 7.55 (m, 2.0H, H-17/H-18/H-19/H-20), 7.42 – 7.29 (m, 4.0H, H-17/H-18/H-19/H-20), 5.10 (d, *J* = 8.1 Hz, 0.5H, H-6), 5.08 (dd, *J* = 11.2 Hz, 3.5 Hz, 0.5H, H-14), 4.71 (dd, *J* = 11.2 Hz, 2.6 Hz, 0.5H, H-14), 4.59 (dd, *J* = 8.0 Hz, 5.8 Hz, 0.5H, H-11), 4.53 (dd, *J* = 10.5 Hz, 6.9 Hz, 0.5H, H-14), 4.27 (t, *J* = 6.9 Hz, 0.5H, H-15), 4.20 – 4.12 (m, 1.0H, H-5, H-14), 4.11 – 4.08 (m, 0.5H, H-15), 3.78 – 3.73 (m 1.0H, H-1, H-5), 3.68 - 3.60 (m, 1.5H, H-1, H-7, H-11), 3.53 – 3.49 (m, 0.5H, H-1), 3.34 (q, *J* = 9.3 Hz, 0.5H, H-1), 3.20 (q, *J* = 8.1 Hz, 7.2 Hz, 0.5H, H-7), 2.82 (t, *J* = 8.1 Hz, 0.5H, H-6), 2.60 – 2.56 (m, 0.5H, H-3/9), 2.47 – 2.40 (m, 0.5H, H-3/9), 2.31 – 2.19 (m, 1.5H, H-3/9, H-10), 2.15 – 2.08 (m, 1.0H, H-10), 2.02 – 1.84 (m, 2.5H, H-2, H-3, H-9), 1.72 – 1.66 (m, 1.0H, H-2), 1.63 – 1.56 (m, 1.0H, H-3, H-9), 1.52 (s, 1.5H, H-23/24), 1.36 (s, 1.5H, H-23/24), 1.34 (s, 1.5H, H-23/24), 1.00 (s, 1.5H, H-23/24).
**^{13}C-NMR**	(125 MHz, $CDCl_3$, mixture of rotamers) δ [ppm] = 173.9 (s, C12), 170.5 (s, C8), 170.1 (s, C8), 155.5 (s, C13), 154.9 (s, C13), 144.7, 144.5, 144.0, 143.8, 141.4, 141.3, 141.3, 141.2 (s, C16, C21), 127.9, 127.8, 127.8, 127.6,

	127.5, 127.4, 127.2, 127.2, 125.6, 125.2, 124.6, 124.3, 120.1, 120.0, 119.8, 119.6 (d, C17, C18, C19, C20), 110.8 (s, C22), 110.4 (s, C22), 77.4 (d, C5), 77.3 (d, C5), 76.8 (d, C6), 76.8 (d, C6), 69.3 (s, C4), 68.2 (s, C4), 67.7 (t, C14), 64.1 (t, C14), 59.4 (d, C11), 59.2 (d, C7), 59.0 (d, C11), 58.8 (d, C7), 48.6 (t, C1), 48.2 (t, C1), 47.4 (d, C15), 47.3 (d, C15), 32.8 (t, C3/C9), 31.8 (t, C3/C9), 31.1 (t, C3/C9), 31.1 (t, C3/C9), 27.3 (t, C10), 27.2 (t, C10), 26.7 (q, C23/C24), 26.5 (q, C23/C24), 24.9 (q, C23/C24), 24.5 (q, C23/C24), 24.4 (t, C2), 23.6 (t, C2).
**IR**	$\tilde{\nu}$[cm^{-1}] = 3459 (w), 3066 (w), 3042 (w), 2983 (m), 2955 (m), 2936 (m), 2898 (w), 2882 (w), 1736 (m), 1700 (s), 1682 (s), 1419 (m), 1382 (w), 1342 (m), 1260 (m), 1211 (m), 1162 (m), 1149 (m), 1068 (m), 1007 (w), 978 (w), 937 (w), 872 (w), 743 (m).
**HRMS (ESI)**	calcd: m/z = 533.22823 [M+H]$^+$, 555.21017 [M+Na]$^+$, found: 533.22845 [M+H]$^+$, 555.21028 [M+Na]$^+$.

Synthesis of Fmoc-(MeO)$_2$-ProM1-OH

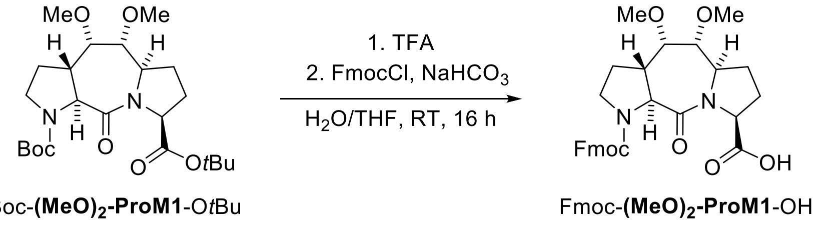

The Fmoc-procedure was applied on Boc-**(MeO)$_2$-ProM1**-O*t*Bu (40 mg, 88 µmol, 1.0 eq) giving rise to a colorless solid after extraction and drying *in vacuo*.

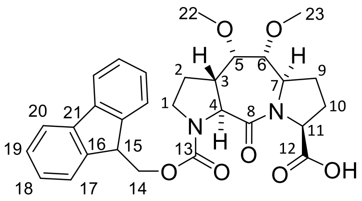

**Yield**	43 mg, 83 µmol, 94%.
**MW**	520.58 g mol^{-1} ($C_{29}H_{32}N_2O_7$).
**R$_f$**	0.31 (CH_2Cl_2/EtOH/HOAc = 9:1:0.05).
**Smp**	136 - 140 °C.
**[α]$^{20}_{\lambda}$**	(c = 0.18, MeOH): -152.6° (365 nm), -99.1° (436 nm), -58.7° (546 nm), -56.7° (579 nm), -61.7° (589 nm).
**^{1}H-NMR**	(500 MHz, $CDCl_3$, mixture of rotamers) δ [ppm] = 8.53 (br. s., 0.7H, OH), 7.76 – 7.72, 7.65 – 7.51, 7.40 – 7.35, 7.32 – 7.26 (4 x m, 4 x 2.0H, H-17, H-18, H-19, H-20), 4.73 – 4.71 (m, 1.0H, H-4, H-11), 4.54 – 4.48 (m, 2.0H, H-4, H-11, H-14), 4.37 – 4.31 (m, 1.0H, H-7, H-14), 4.28 – 4.24 (m, 1.0H, H-14, H-15), 4.20 – 4.16 (m, 1.0H, H-7, H-15), 3.86 – 3.78 (m, 2.0H, H-1, H-5), 3.61, 3.60 (2 x s, 3.0H, H-22), 3.43 (s, 3.0H, H-23), 3.42 – 3.39 (m, 0.5H, H-1), 3.28 (dt, *J* = 11.1 Hz, 5.7 Hz, 0.5H, H-1), 3.19 – 3.16 (m, 1.0H, H-6), 2.36 – 2.31 (m, 1.0H, H-3), 2.27 – 2.21 (m, 1.0H, H-9), 2.15 – 2.06 (m, 2.0H, H-9, H-10), 2.04 – 1.96 (m, 1.5H, H-2, H-10), 1.91 – 1.84 (m, 1.0H, H-2), 1.83 – 1.77 (m, 0.5H, H-2).
**^{13}C-NMR**	(125 MHz, $CDCl_3$, mixture of rotamers) δ [ppm] = 174.2 (s, C12), 174.1 (s, C12), 172.0 (s, C8), 172.0 (s, C8), 155.2 (s, C13), 155.0 (s, C13), 144.7, 144.5, 143.9, 143.8, 141.4, 141.4, 141.4, 141.3 (s, C16, C21), 127.8, 127.7, 127.7, 127.6, 127.2, 127.1, 127.1, 126.9, 125.4, 125.2, 125.1, 125.1, 120.0, 119.9, 119.9 (d, C17, C18, C19, C20), 85.3 (d, C6), 84.9 (d, C6), 75.6 (d, C5), 75.5 (d, C5), 67.7 (t, C14), 66.9 (t, C14), 62.4 (q, C22), 62.3 (q, C22), 60.9 (d, C11), 60.6 (d, C11), 58.4 (q, C23), 58.3 (q, C23), 56.8 (d, C4), 56.4 (d, C4), 56.1 (d, C7), 56.0 (d, C7), 47.6 (d, C15), 47.4 (d, C15), 46.7 (t, C1), 46.5 (t, C1), 46.3 (d, C3), 45.4 (d, C3), 29.4 (t, C9), 29.2 (t, C9), 28.3 (t, C2), 27.8 (t, C2), 26.8 (t, C10), 26.7 (t, C10).
**IR**	$\tilde{\nu}$[cm^{-1}] = 3456 (w), 3066 (w), 3042 (w), 2973 (m), 2949 (m), 2930 (m), 2900 (m), 2833 (w), 1706 (s), 1679 (s), 1446 (s), 1421 (s), 1357 (m), 1268 (w), 1231 (w), 1193 (m), 1177 (m), 1119 (s), 1079 (m), 1058 (w), 960 (w), 900 (w), 761 (w), 740 (m), 686 (w).
**HRMS (ESI)**	calcd: m/z = 521.22823 [M+H]$^+$, 543.21017 [M+Na]$^+$, found: 521.22833 [M+H]$^+$, 543.21008 [M+Na]$^+$.

Synthesis of Fmoc-(EtO)$_2$-ProM1-OH

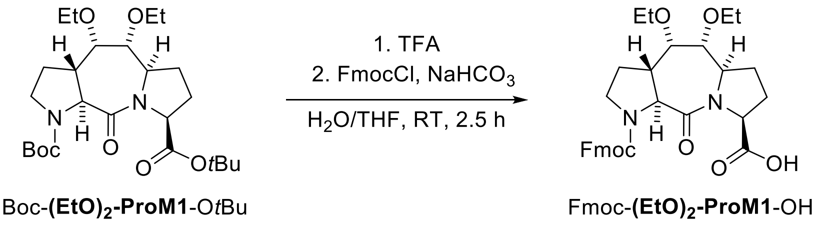

Boc-**(EtO)$_2$-ProM1**-O*t*Bu (128 mg, 265 µmol 1.0 eq) was used as a starting material for the general Fmoc-protocol while reducing the reaction time with the Fmoc-Cl solution to 2.5 h. As a result, a colorless solid was obtained after extraction and vacuum drying.

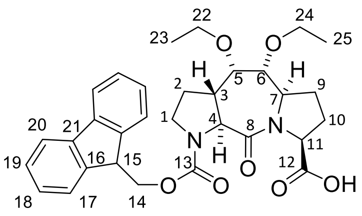

**Yield**	132 mg, 241 µmol, 91%.
**MW**	548.64 g mol^{-1} ($C_{31}H_{36}N_2O_7$).
**R$_f$**	0.45 (CH_2Cl_2/EtOH/HOAc = 9:1:0.05).
**Smp**	126 - 130 °C.
**[α]$^{20}_{λ}$**	(c = 0.17, MeOH): -148.4° (365 nm), -96.3° (436 nm), -57.7° (546 nm), -51.8° (579 nm), -52.0° (589 nm).
**^{1}H-NMR**	(500 MHz, $CDCl_3$, mixture of rotamers) δ [ppm] = 9.55 (br. s., 0.6H, OH), 7.76 – 7.72, 7.66 – 7.52, 7.40 – 7.35, 7.32 – 7.26 (4 x m, 4 x 2.0H, H-17, H-18, H-19, H-20), 4.76 (d, *J* = 10.1 Hz, 0.6H, H-4), 4.71 (dd, *J* = 7.6 Hz, 3.4 Hz, 0.5H, H-11), 4.59 (d, *J* = 9.8 Hz, 0.4H, H-4), 4.54 (dd, *J* = 9.8 Hz, 5.8 Hz, 0.5H, H-14), 4.51 – 4.48 (m, 1.0H, H-11, H-15), 4.40 – 4.18 (m, 3.0H, H-7, H-14, H-15), 3.99 – 3.93 (m, 1.0H, H-22), 3.87 – 3.80 (m, 2.0H, H-1, H-5), 3.70 – 3.64 (m, 1.0H, H-24), 3.58 – 3.50 (m, 1.0H, H-22), 3.45 – 3.39 (m, 1.6H, H-1, H-24), 3.32 – 3.26 (m, 1.4H, H-1, H-6),<br>2.38 – 2.30 (m, 1.0H, H-3), 2.26 – 2.20 (m, 1.0H, H-9), 2.16 – 1.88 (m, 4.0H, H-2, H-9, H-10), 1.85 – 1.74 (m, 1.0H, H-2), 1.24, 1.23, 1.22, 1.21, 1.20, 1.19, 1.17, 1.16 (8 x s, 6.0H, H-23, H-25).
**^{13}C-NMR**	(150 MHz, $CDCl_3$, mixture of rotamers) δ [ppm] = 174.3 (s, C12), 174.1 (s, C12), 172.0 (s, C8), 171.9 (s, C8), 155.2 (s, C13), 155.0 (s, C13), 144.7, 144.5, 143.9, 143.9, 141.4, 141.4, 141.3, 141.3 (s, C16, C21), 127.7, 127.7, 127.6, 127.2, 127.1, 127.0, 126.9, 125.4, 125.2, 125.1, 125.1, 120.0, 119.9 (d, C17, C18, C19, C20), 83.3 (d, C6), 82.9 (d, C6), 74.6 (d, C5), 69.6 (t, C22), 67.6 (t, C14), 67.0 (t, C14), 66.0 (t, C24), 60.9 (d, C11), 60.6 (d, C11), 56.9 (d, C4), 56.6 (d, C4), 56.1 (d, C7), 47.4 (d, C15), 47.4 (d, C15), 46.7 (t, C1), 46.5 (t, C1), 46.4 (d, C3), 45.4 (d, C3), 29.3 (t, C9), 29.2 (t, C9), 28.3 (t, C2), 27.8 (t, C2), 26.9 (t, C10), 26.7 (t, C10), 15.9 (q, C23/C25), 15.5 (q, C23/C25).
**IR**	$\tilde{v}$ [cm^{-1}] = 3445 (w), 3062 (w), 2972 (s), 2930 (m), 2901 (m), 1735 (w), 1705 (s), 1678 (s), 1477 (w), 1450 (s), 1419 (s), 1355 (m), 1331 (m), 1292 (w), 1249 (m), 1228 (m), 1180 (m), 1117 (s), 1089 (s), 1074 (s), 1066 (s), 1058 (s), 923 (w), 880 (w), 784 (w), 760 (m), 740 (m), 685 (w), 621 (w), 596 (w), 544 (w), 426 (w).
**HRMS (ESI)**	calcd: m/z = 549.25953 [M+H]$^+$, 571.24147 [M+Na]$^+$, found: 549.25964 [M+H]$^+$, 571.24132 [M+Na]$^+$.

Synthesis of Fmoc-(MeO)$_2$-ProM2-OH

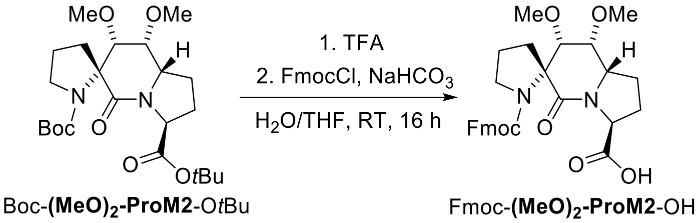

Following the Fmoc-procedure, Boc-**(MeO)$_2$-ProM2**-O*t*Bu (83 mg, 183 µmol, 1.0 eq) was re-protected. After extraction and column chromatography on ultrapure silica gel (CH_2Cl_2/MeOH = 20:1), a colorless solid was obtained.

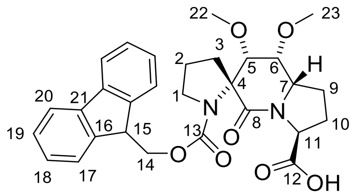

Yield 51 mg, 98 µmol, 54%.

MW 520.58 g mol^{-1} ($C_{29}H_{32}N_2O_7$).

R$_f$ 0.35 (CH_2Cl_2/EtOH/HOAc = 9:1:0.05).

Smp 94 - 98 °C.

$[\alpha]^{20}_{\lambda}$ (c = 0.21, MeOH): -6.7° (365 nm), -17.6° (436 nm), -14.3° (546 nm), -13.8° (579 nm), -15.5° (589 nm).

^{1}H-NMR (600 MHz, $CDCl_3$) δ [ppm] = 7.77 – 7.74, 7.62 – 7.55, 7.41 – 7.38, 7.32 – 7.29 (4 x m, 4 x 2.0H, H-17, H-18, H-19, H-20), 4.59 (t, *J* = 8.5 Hz, 1.0H, H-11), 4.38 (dd, *J* = 10.0 Hz, 6.2 Hz, 1.0H, H-14), 4.28 – 4.22 (m, 2.0H, H-14, H-15), 3.83 (dd, *J* = 7.7 Hz, 2.5 Hz, 1.0H, H-6), 3.80 – 3.76 (m, 1.0H, H-7), 3.74 – 3.69 (m, 2.0H, H-1, H-5), 3.59 – 3.56 (m, 1.0H, H-1), 3.55 (s, 3.0H, H-22), 3.43 (s, 3.0H, H-23), 2.59 – 2.55 (m, 1.0H, H-3), 2.31 – 2.24 (m, 4.0H, H-3, H-9, H-10), 2.12 – 2.06 (m, 1.0H, H-2), 1.94 – 1.87 (m, 1.0H, H-2), 1.80 -1.74 (m, 1.0H, H-9).

^{13}C-NMR (150 MHz, $CDCl_3$) δ [ppm] = 171.9 (s, C8/C12), 171.8 (s, C8/C12), 154.8 (s, C13), 144.0, 143.5, 141.3 (s, C16, C21), 127.8, 127.7, 127.1, 127.0, 125.2, 125.0, 120.0, 120.0 (d, C17, C18, C19, C20), 80.0 (d, C6), 78.6 (d, C5), 68.9 (s, C4), 67.6 (t, C14), 61.4 (q, C22), 60.7 (d, C11), 59.4 (d, C7), 58.2 (q, C23), 48.6 (t, C1), 47.1 (d, C15), 33.1 (t, C3), 29.9 (t, C9), 26.1 (t, C10), 23.4 (t, C2).

IR $\tilde{\nu}$[cm^{-1}] = 3445 (w), 2988 (s), 2975 (s), 2901 (m), 1742 (w), 1698 (m), 1656 (m), 1450 (m), 1407 (m), 1394 (m), 1354 (m), 1337 (m), 1250 (m), 1228 (m), 1193 (m), 1077 (s), 1066 (s), 1056 (s), 1028 (m), 892 (w), 761 (w), 741 (w), 621 (w), 427 (w).

HRMS (ESI) calcd: m/z = 521.22823 [M+H]$^+$, 543.21017 [M+Na]$^+$, found: 521.22833 [M+H]$^+$, 543.21010 [M+Na]$^+$.

Synthesis of Fmoc-(EtO)$_2$-ProM2-OH

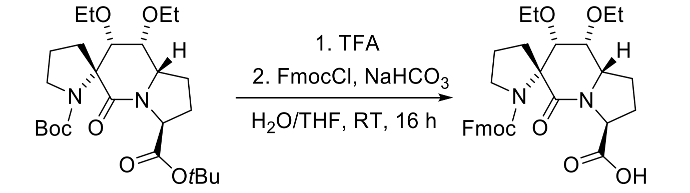

Boc-**(EtO)$_2$-ProM2**-O*t*Bu Fmoc-**(EtO)$_2$-ProM2**-OH

Boc-**(EtO)$_2$-ProM2**-O*t*Bu (133 mg, 276 µmol, 1.0 eq) was used as a starting material for the general Fmoc-procedure to yield a colorless solid after extraction and vacuum drying.

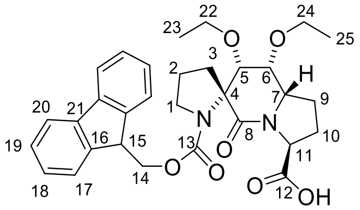

Yield 131 mg, 239 µmol, 87%.

MW 548.64 g mol^{-1} ($C_{31}H_{36}N_2O_7$).

R$_f$ 0.41 (CH_2Cl_2/EtOH/HOAc = 9:1:0.05).

Smp 58 - 61 °C.

[α]$^{20}_{\lambda}$ (c = 0.18, MeOH): -8.6° (365 nm), -15.4° (436 nm), -12.0° (546 nm), -12.0° (579 nm), -13.7° (589 nm).

^{1}H-NMR (500 MHz, $CDCl_3$, mixture of rotamers) δ [ppm] = 7.77 – 7.74, 7.62 – 7.54, 7.41 – 7.37, 7.32 – 7.28 (4 x m, 4 x 2.0H, H-17, H-18, H-19, H-20), 4.61 (t, *J* = 9.2 Hz, 1.0H, H-11), 4.37 (dd, *J* = 9.8 Hz, 6.4 Hz, 1.0H, H-14), 4.28 – 4.20 (m, 2.0H, H-14, H-15), 3.92 – 3.84 (m, 2.0H, H-6, H-22), 3.82 – 3.77 (m, 1.0H, H-7), 3.73 – 3.67 (m, 2.0H, H-1, H-5), 3.67 – 3.61 (m, 1.0H, H-24), 3.59 – 3.54 (m, 2.0H, H-1, H-22), 3.53 – 3.47 (m, 1.0H, H-24), 2.63 – 2.59 (m, 1.0H, H-3), 2.32 – 2.23 (m, 4.0H, H-3, H-9, H-10), 2.12 – 2.04 (m, 1.0H, H-2), 1.93 – 1.85 (m, 1.0H, H-2), 1.79 – 1.70 (m, 1.0H, H-9), 1.21, 1.20, 1.19, 1.18, 1.16 (5 x s, 6.0H, H-23, H-25).

^{13}C-NMR (150 MHz, $CDCl_3$, mixture of rotamers) δ [ppm] = 172.4 (s, C8), 171.6 (s, C12), 154.9 (s, C13), 144.1, 143.6, 141.4 (s, C16, C21), 127.8, 127.8, 127.1, 127.1, 125.2, 125.1, 120.1, 120.1 (d, C17, C18, C19, C20), 78.3 (d, C6), 77.7 (d, C5), 69.3 (t, C22), 69.1 (s, C4), 67.6 (t, C14), 65.9 (t, C24), 61.2 (d, C11), 59.7 (d, C7), 48.7 (t, C1), 47.2 (d, C15), 33.4 (t, C3), 29.8 (t, C9), 26.0 (t, C10), 23.4 (t, C2), 15.8 (q, C23/C25), 15.6 (q, C23/C25).

IR $\tilde{\nu}$ [cm^{-1}] = 3426 (w), 3070 (w), 3041 (w), 2973 (w), 2929 (w), 2894 (w), 1732 (w), 1698 (m), 1656 (m), 1449 (m), 1407 (s), 1353 (m), 1335 (m), 1281 (w), 1189 (m), 1114 (s), 1073 (m), 1049 (m), 1003 (w), 937 (w), 879 (w), 760 (m), 740 (s), 670 (w), 644 (w), 621 (w), 543 (w), 499 (w), 425 (w).

HRMS (ESI) calcd: m/z = 549.25953 [M+H]$^+$, 571.24147 [M+Na]$^+$, found: 549.25968 [M+H]$^+$, 571.24135 [M+Na]$^+$.

Stereochemical Assignments

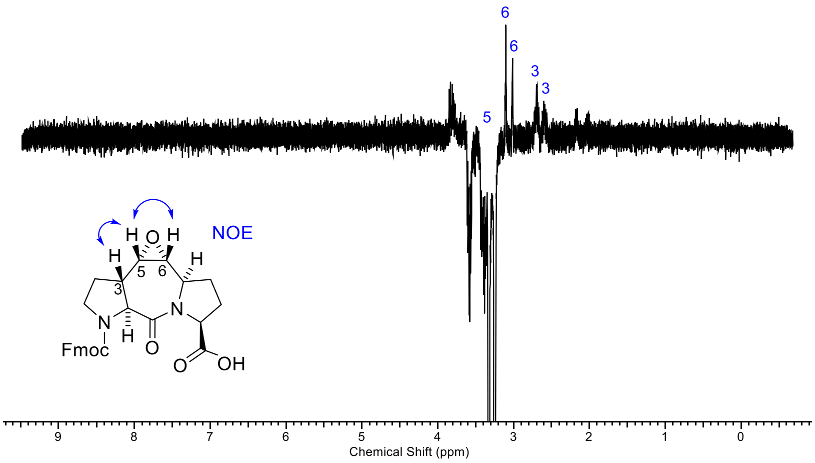

Figure S1. 1D-NOE NMR spectrum (500 MHz, $CDCl_3$) of Fmoc-**ep-ProM1**-OH and observed NOE.

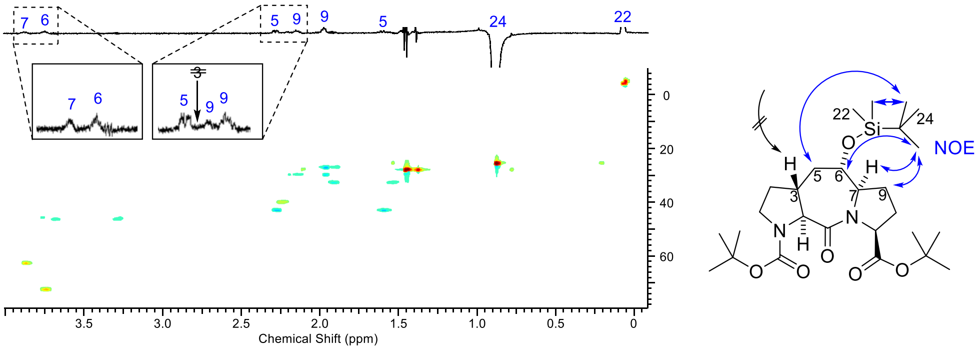

Figure S2. Correlation of 1D-NOE with HMQC-spectrum (500 MHz, $CDCl_3$) of Boc-**(TBSO)-ProM1**-O*t*Bu.

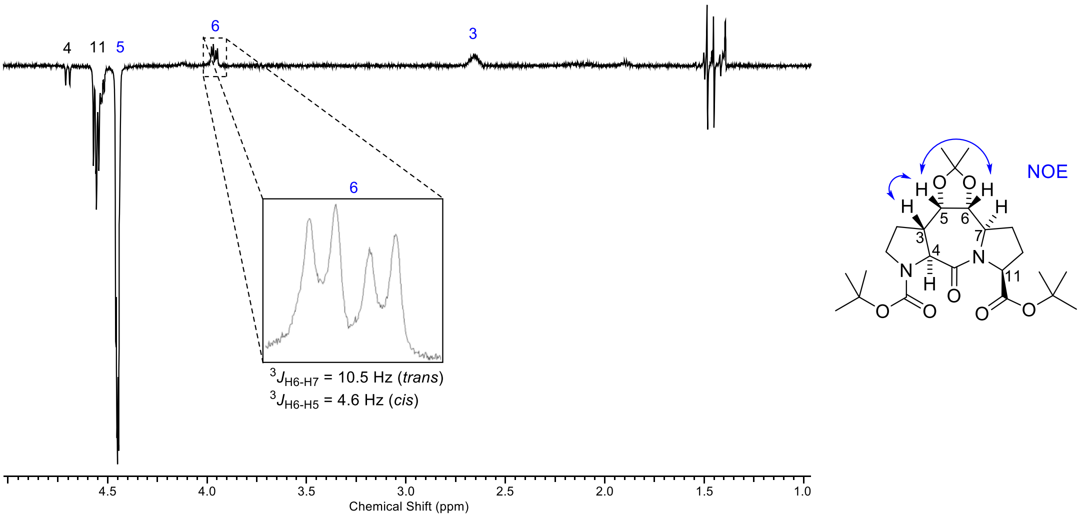

Figure S3. 1D-NOE spectrum (500 MHz, $CDCl_3$) of Boc-α-**(Me_2CO_2)-ProM1**-O*t*Bu, observed NOE and dd signal of H-6.

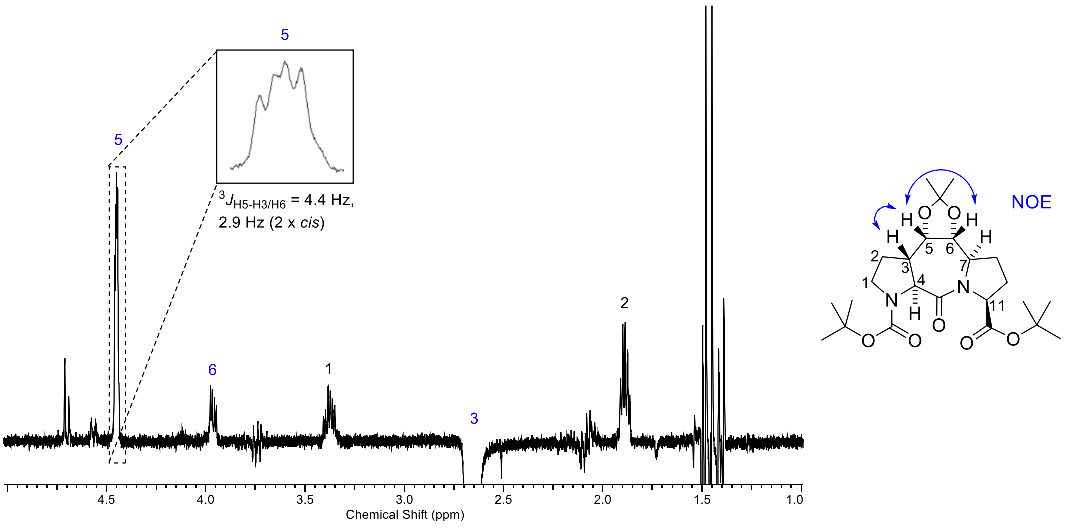

Figure S4. 1D-NOE spectrum (500 MHz, $CDCl_3$) of Boc-α-**(Me₂CO₂)-ProM1**-O*t*Bu, observed NOE and dd signal of H-5.

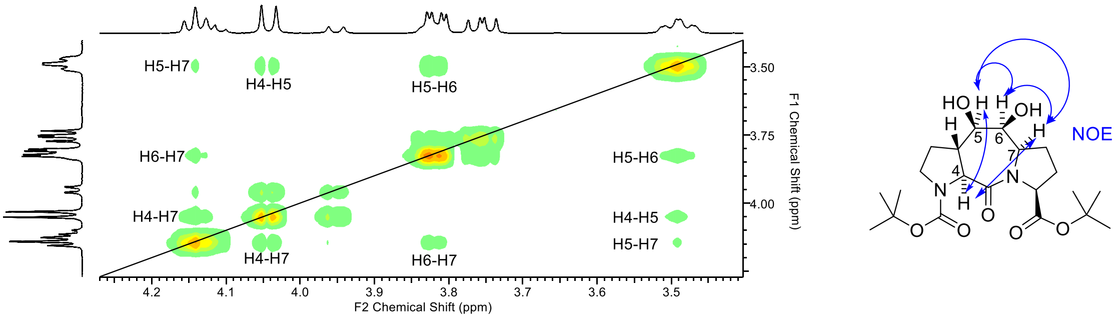

Figure S5. NOESY spectrum (500 MHz, $CDCl_3$) of Boc-β-**(HO)₂-ProM1**-O*t*Bu and NOE indicated in blue.

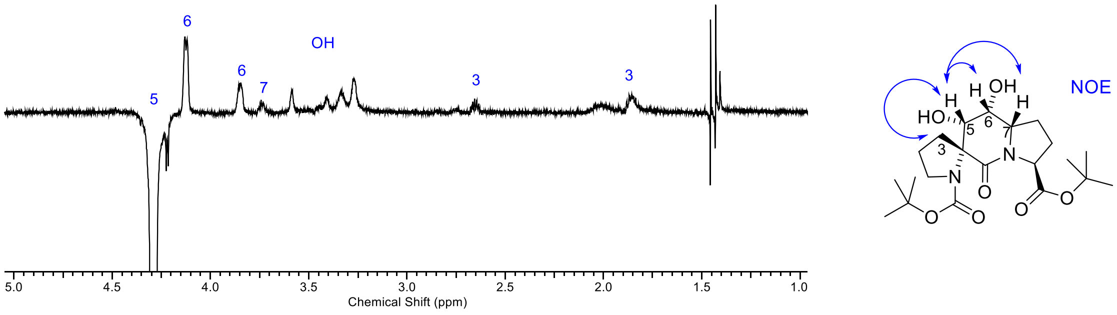

Figure S6. 1D-NOE spectrum (600 MHz, $CDCl_3$) of Boc-α-**(HO)₂-ProM2**-O*t*Bu and observed NOE in blue.

Solid Phase Peptide Syntheses

General conditions - All peptides were prepared by an automated peptide synthesizer (Multisyntech Syro I). Solid phase peptide syntheses were performed with NovaPEG low loaded Rink amide resin (*Merck*, loading: 0.18 – 0.23 mmol/g) to avoid chain aggregation effects.[4] Swelling of resins and all standard coupling reactions were performed in DMF as solvent using equimolar amounts (8 – 16 eq) of Fmoc-protected amino acids (Fmoc-Gly, Fmoc-Pro, Fmoc-Arg(Pbf), IRIS Biotech), diisopropylcarbodiimide (DIC) and oxyma following the Fmoc/*t*Bu strategy. The Fmoc protecting group was cleaved using 30% v/v piperidine in DMF at the end of each coupling cycle.
For sequences containing non-standard amino acids (Hyp or ProMs) a three-step procedure was carried out. Firstly, the C-terminal part of the sequence including Pro, Arg or Gly was synthesized automatically. Secondly, the Fmoc-protected ProMs or Fmoc-Hyp were coupled manually to the free N-termini of the previously installed peptide chains (*vide infra*) and thirdly, the remaining N-terminal part of the sequence was produced by automated synthesis again.

Cleavage from Resin and Purification - Generally, the N-termini of peptides were either acetylated or capped with Biotin-Ebes (*vide infra*) before the peptides were cleaved from the resin by shaking with conc. trifluoroacetic acid/triisopropylsilane/water (95:2.5:2.5, v/v/v) at RT for 3 h. After filtration and washing of the resin with TFA the peptides were precipitated by adding the combined filtrates to cold Et_2O (-20 °C, overnight). The precipitates were washed with Et_2O, dissolved in *t*BuOH/water (1:3, v/v), lyophilized and redissolved in HPLC buffer. To suppress triple helix formation, the peptides were incubated 10 min at 80 °C prior to semipreparative RP-HPLC (Hitachi Elite LaChrom system, column: Macherey Nagel VP 250/8 Nucleodur 100-5 C18ec) using a linear gradient of 10 → 40% B in A (A: 0.1% TFA in H_2O, B: 0.08% TFA in MeCN) and a flow rate of 1.5 ml/min. Relevant fractions were pooled, lyophilized and the desired peptides were obtained as colorless foams. The product identity was confirmed by LC-ESI-MS analysis (gradient: 10 → 60% MeCN in H_2O + 0.1% HCO_2H; Chromolith Performance RP-18e, 100-4.6 mm, *Merck*; LTQ-XL, *Thermo Scientific*). For each mass spectrum the whole range of LC retention times (0 - 18 min) was scanned to check purity (see section ESI-MS spectra of peptides).

Protocol for manual coupling - The Fmoc-protected ProM (2 eq) and HATU (2 eq) were dissolved in DMF/CH_2Cl_2 (9:1). After 5 min incubation at RT the active ester solution was pipetted on the pre-swollen resin and the coupling reaction was initiated by addition of DIPEA (4 eq). The resin was shaken at RT for 2 h, washed (DMF, CH_2Cl_2, MeOH, then Et_2O) and dried *in vacuo*.

N-terminal Acetylation - To cap the *N*-terminus, an excess of acetic anhydride (20 eq) and DIPEA (20 eq) in CH_2Cl_2 was added to the resin, which was shaken 30 min at RT. After removing the solution the resin was washed and dried.

N-terminal capping with Biotin-Ebes - A solution of Fmoc-Ebes (2 eq) and Oxyma (2 eq) in DMF was pipetted to the resin and the reaction was started by addition of DIC (2 eq). After shaking at RT overnight the supernatant solution was removed, the resin was washed and dried. In the following, the Fmoc group was removed (30% v/v piperidine in DMF) and the free N-terminus capped with (+)-biotin (3 eq) under standard coupling conditions.

CD spectra and Thermal Denaturation Experiments

Experimental setup - Vacuum-dried peptides were dissolved in phosphate-buffered saline (10 mM Na_2HPO_4, 10 mM NaH_2PO_4, 150 mM NaCl, pH = 7.0) and the solutions (60 µM peptide) were incubated at 4 °C for at least 48 h for trimerization. CD measurements were performed on a *Jasco* J-715 spectropolarimeter using a quartz cuvette (*Hellma Analytics*, 1 mm path length) and spectra were recorded at 4 °C (1 nm band with, scan from 260 to 200 nm) indicating a maximum at 225 nm, which is characteristic of triple helix formation.[5] To study thermal transitions the ellipticity at 225 nm was monitored while increasing the temperature from 4 to 70 °C at a rather slow heating rate of 12 °C h^{-1} which was controlled electronically (*Jasco* PTC-348 WI Peltier element). According to the literature this heating rate still corresponds to non-equilibrium conditions, however, the generated data can be used for internal comparison of the peptides.[6]

Data interpretation - For T_m-determination a two-state-model was employed in which three peptide chains combine to a triple helix assuming a previously reported mathematical model (*vide infra*).[7][8] Constants were set (c = 0.00006, R = 8.31) and auxiliary variables (P, U, V, K) were used to describe the temperature dependence of the folded fraction F. Thermal transition curves were fitted by optimizing the parameters θ_{min}, θ_{max}, ΔH, and T_m using the Generalized Reduced Gradient algorithm implemented in the solver tool of MS Excel.[9]. An error of ±1.0 °C was estimated for the resulting T_m values by repeating denaturation experiments.

$$F_{exp} = \frac{\theta - \theta_{min}}{\theta_{max} - \theta_{min}}$$

$$F_{calc}(T) = U + V + 1$$

$$U = \sqrt[3]{-\frac{p}{2} + \sqrt{\frac{p^2}{4} + \frac{p^3}{27}}}$$

$$V = -\sqrt[3]{\frac{p}{2} + \sqrt{\frac{p^2}{4} + \frac{p^3}{27}}}$$

$$p = \frac{1}{3c^2K}$$

$$K = \exp\left(\frac{\Delta H}{RT}\left(\frac{T}{T_m} - 1\right) - \ln(0.75c^2)\right)$$

CD spectra

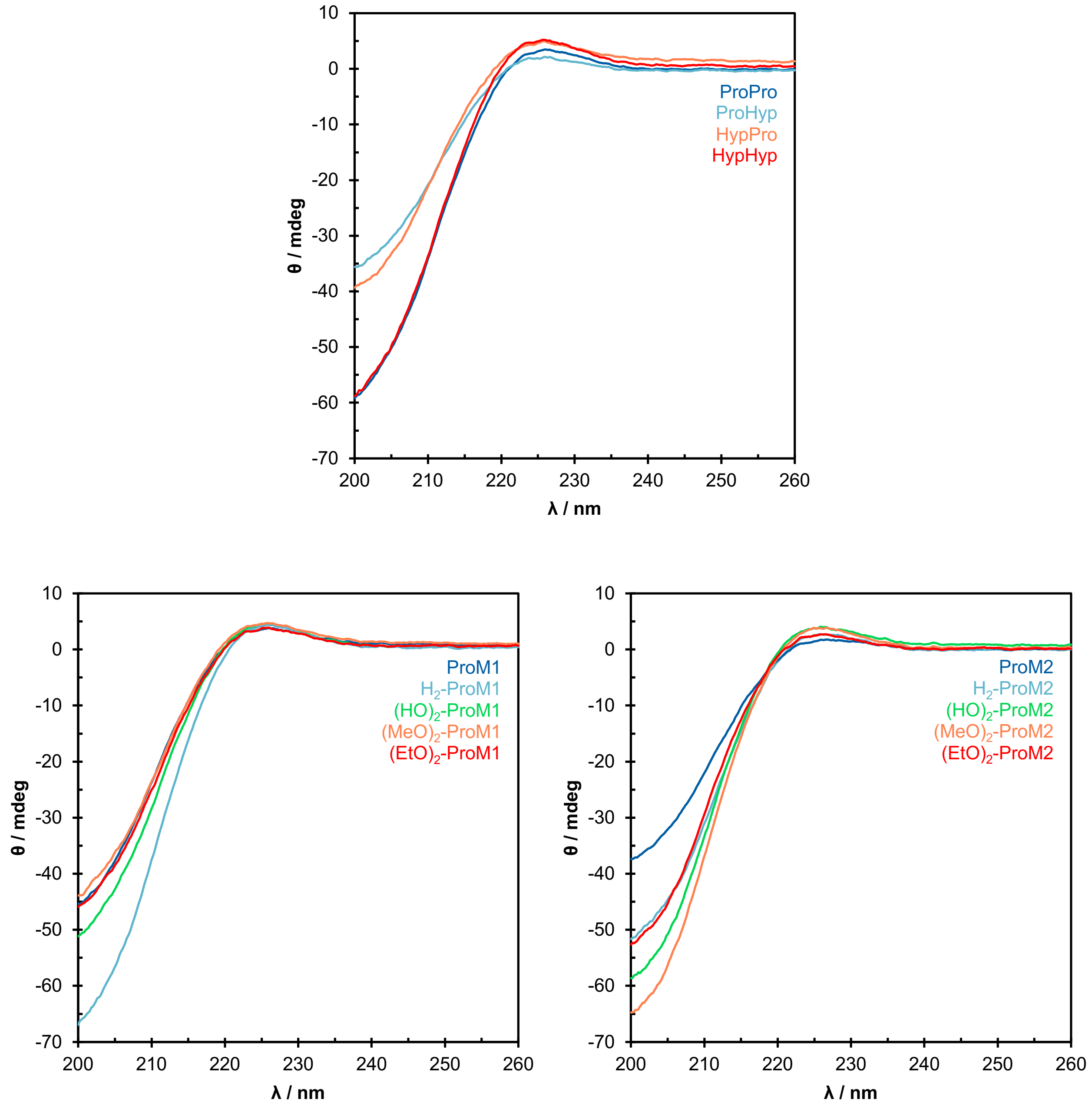

Figure S7. CD spectra of triple-helical host-guest-peptides in phosphate-buffered saline (10 mM sodium phosphate, 150 mM NaCl, pH = 7.0) at 4 °C. Different dipeptides [XY] were incorporated into the sequence Ac-(PPG)$_5$-PRG-PPG-XYG-(PPG)$_3$-NH$_2$ (see color code).

Additional Denaturation Experiments

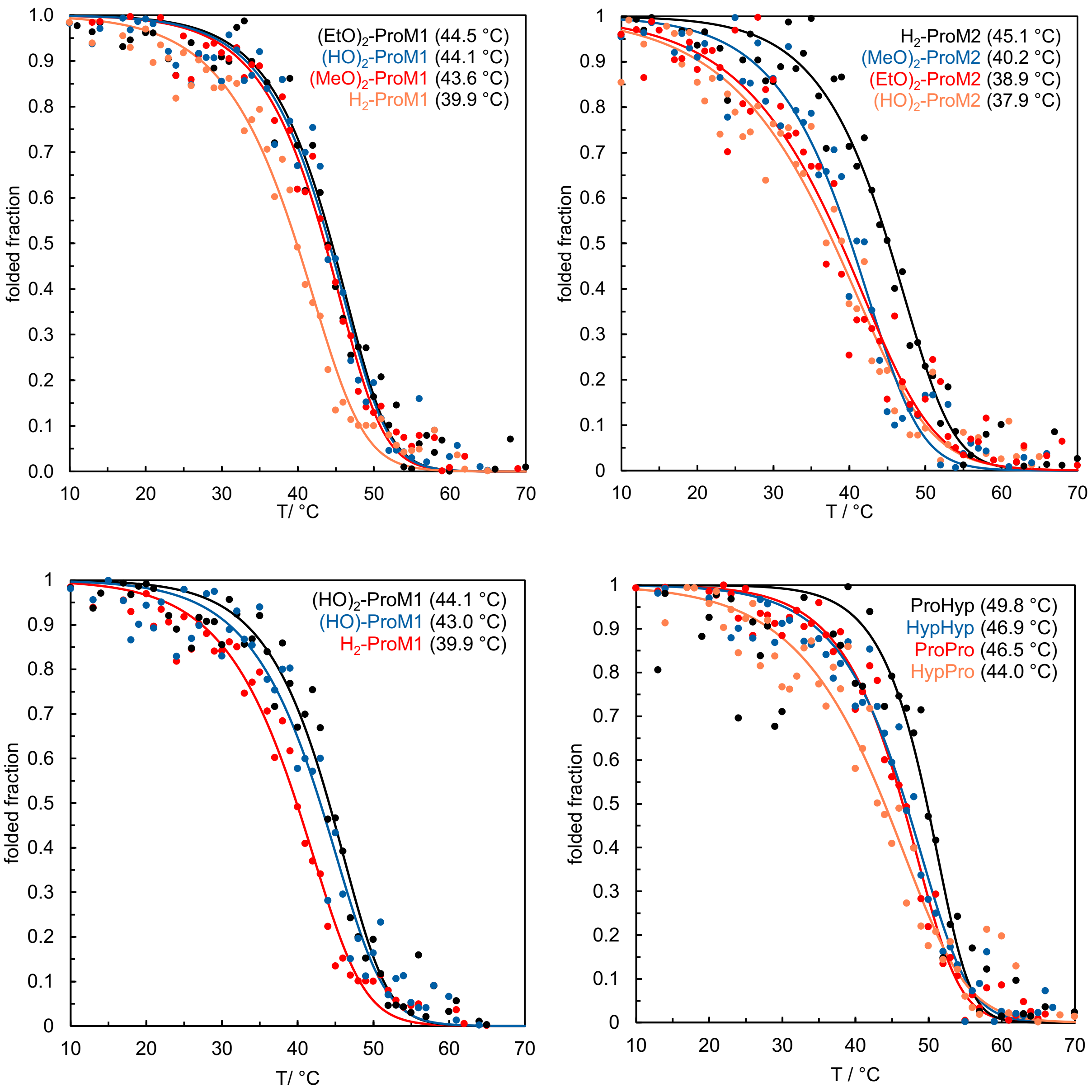

Figure S8. Thermal CD denaturation experiments of alkoxy-ProM1-substituted (top left), alkoxy-ProM2-substituted (top right), hydroxy-ProM1-substituted (bottom left) and 4*R*-hydroxyproline-substituted peptides (bottom right) with T_m-values given in parentheses (sequence: Ac-(PPG)$_5$-PRG-PPG-[XY]G-(PPG)$_3$-NH$_2$, 12 °C h^{-1} heating rate, PBS-buffer).

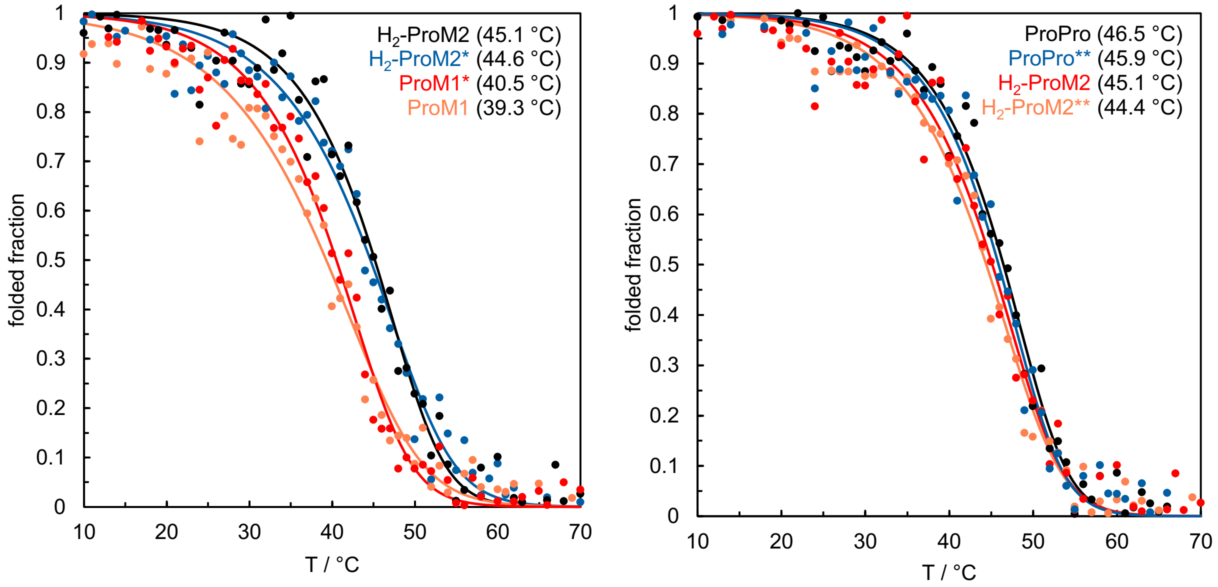

Figure S9. Denaturation curves of Ac-$(PPG)_5$-PRG-PPG-[XY]G-$(PPG)_3$-NH_2 host-guest peptides; substitution-site altered peptides Ac-$(PPG)_3$-[XY]G-PPG-PRG-$(PPG)_5$-NH_2 indicated with (*, see left); and arginine-free peptides Ac-$(PPG)_7$-[XY]G-$(PPG)_3$-NH_2 indicated with (**, see right). Changing the substitution site within the peptide sequence did not significantly change the corresponding T_m-values. Based on the arginine-proline-substitution experiment, no interaction between ProMs and the arginine side chain seemed to affect triple-helical stability.

Computational Methods and Primary Results

Conformational Search and Preoptimization - To generate collagen-relevant geometries a two-step procedure was carried out using *Schrödinger Suite 2017-1*. Firstly, the mixed torsional/low-mode sampling method in *MacroModel* was employed to find lowest-energy conformations within a 30 $kJmol^{-1}$ window of *OPLS_2005* force field energy.[10] The conformational search was started with *N*-acetylated methyl esters (Ac-[dipeptide]-OMe) to simulate a peptidic environment and to avoid intramolecular H bonds, respectively. For each investigated diproline derivative 10-100 structures were obtained, of which a set of up to four collagen-relevant conformers was selected (all-*trans* amides, ψ_{Yyy} close to 180°, *endo*/*endo*, *endo*/*exo*, *exo*/*endo* or *exo*/*exo* puckered diproline rings). Secondly, the geometry of each conformer from this set was optimized in vacuum at the level of density functional theory (DFT) using B3LYP-D3/6-311++G** containing a dispersion-corrected functional, which has been recommended for proline-derived systems.[11][12] To avoid convergence problems and to ensure time-efficient calculations the GVB-DIIS algorithm, an SCF level shift of 0.5 and the fine DFT grid density were employed in *Schrödinger's Jaguar* module.[13] These parameter adjustments have been recommended by the software manufacturer. Frequency calculations were carried out on all optimized geometries resulting in no imaginary frequencies and thus in the presence of true minima on the potential energy surface.

Geometry Optimization - For the correct determination of relative energies further calculations in aqueous solution were performed for each conformer using the *Gaussian 16* software package.[14] The Schrödinger geometries served as input and were refined at the B3LYP-D3/6-31G* level of theory and frequency calculations again revealed no imaginary frequencies.[12] Solvent effects were taken into account by employing the polarized continuum model (PCM) and the basis set was reduced to limit the computational cost of all the DFT-calculations. Finally, the main chain torsional angles of the lowest-energy conformers and the relative energies for each set of conformers for all diproline derivatives were evaluated (see Table S3 and section Optimized Geometries and Cartesian Coordinates).

Method Validation - The procedure was performed on small test molecules (Ac-Pro-OMe, Ac-Hyp-OMe, see Tables S1-2). As a result, the values for main chain torsional angles as key geometric properties and the *endo*-/*exo*-energy difference for the ring flip could be reproduced.

Table S1. Energies and structural parameters from DFT-Calculations of this work (B3LYP-D3/6-31G*, H_2O-PCM).

trans-Ac-[**amino acid**]-OMe	conformer	energy [hartree]	relative energy [$kJmol^{-1}$]	φ [°]	ψ [°]
**Pro**	endo	-593.163425	0.00	-68 (Lit: -70)[a]	154 (Lit: 152)[a]
	exo	-593.162790	1.67	-56 (Lit: -59)[a]	142 (Lit: 143)[a]
**(4R)-Hyp**	endo	-668.375908	2.58	-66	150
	exo	-668.376891	0.00	-57 (Lit: -57)[b]	141 (Lit: 151)[b]

[a] DFT-calculations (B3LYP/6-31G*, *in vacuo*) from reference [15]; [b] Crystal structure parameters from reference [16].

Table S2. Relative energies [$kJmol^{-1}$] of *trans,exo* conformers of proline and hydroxyproline (E(*trans, endo*) = 0, H_2O-PCM).

method	reference	termini	**Pro**	**(*4R*)-Hyp**
B3LYP-D3/6-31G*// B3LYP-D3/6-31G*	(this work)	Ac-…-OMe	1.67	-2.58
B3LYP/6-311+G**//B3LYP/6-31+G*	[15]	Ac-…-OMe	1.72	-
HF/6-31G*//HF/6-31G*	[17]	Ac-…-NHMe	1.59	-2.09
PBE0/6-31G*//PBE0/6-31G*	[17]	Ac-…-NHMe	1.63	-2.64
MP2/6-31G*//HF/6-31G*	[17]	Ac-…-NHMe	2.55	-3.01

Primary Results

Table S3. Energies and Structural Parameters from DFT-Calculations (B3LYP-D3/6-31G*, H_2O-PCM).

Ac-[dipeptide]-OMe	conformer	energy [hartree]	relative energy [kJmol^{-1}]	φ_{Xxx} [°]	ψ_{Xxx} [°]	φ_{Yyy} [°]	ψ_{Yyy} [°]
**ProM1**	exo/endo	-994.121528	0.00	-67.1	170.2	-73.5	159.6
	exo/exo	-994.119942	4.16	-67.4	174.5	-45.7	140.4
**H_2-ProM1**	exo/endo	-995.362719	0.00	-67.9	169.2	-79.6	162.5
	exo/exo	-995.362296	1.11	-68.9	173.7	-50.9	141.8
**(HO)-ProM1**	exo/endo	-1070.576017	0.61	-68.3	170.5	-81.2	163.2
	exo/exo	-1070.576251	0.00	-68.8	175.3	-52.8	143.9
**$(HO)_2$-ProM1**	exo/endo	-1145.794932	1.13	-68.7	170.4	-82.0	164.1
	exo/exo	-1145.795361	0.00	-69.3	174.6	-52.8	144.1
**$(MeO)_2$-ProM1**	exo/endo	-1224.408294	1.08	-68.7	171.0	-81.1	163.7
	exo/exo	-1224.408706	0.00	-69.5	175.4	-52.6	144.5
**$(EtO)_2$-ProM1**	exo/endo	-1303.053157	1.11	-68.9	170.6	-80.8	163.3
	exo/exo	-1303.053580	0.00	-69.3	175.1	-52.4	144.2
**ProM2**	endo/exo	-994.128309	0.00	-64.9	153.9	-71.7	154.9
	exo/exo	-994.127018	3.39	-53.4	140.5	-75.7	152.5
**H_2-ProM2**	endo/endo	-995.358689	10.19	-64.6	153.1	-100.8	171.1
	endo/exo	-995.362571	0.00	-64.4	151.9	-64.5	151.2
	exo/endo	-995.358010	11.97	-48.0	132.4	-104.8	168.7
	exo/exo	-995.362211	0.95	-42.7	127.2	-69.4	148.4
**$(HO)_2$-ProM2**	endo/endo	-1145.798831	11.10	-61.4	149.7	-96.3	169.9
	endo/exo	-1145.801349	4.49	-61.3	152.7	-60.8	149.6
	exo/endo	-1145.800501	6.71	-51.8	138.2	-94.6	167.6
	exo/exo	-1145.803058	0.00	-44.6	134.4	-62.0	145.0
**$(MeO)_2$-ProM2**	endo/endo	-1224.395669	11.03	-61.5	157.1	-98.4	169.3
	endo/exo	-1224.397622	5.90	-60.8	157.8	-60.3	146.6
	exo/endo	-1224.397973	4.98	-54.0	150.4	-97.8	165.8
	exo/exo	-1224.399871	0.00	-53.4	154.0	-60.9	144.7
**$(EtO)_2$-ProM2**	endo/endo	-1303.043505	4.65	-61.2	159.9	-98.4	169.5
	endo/exo	-1303.045276	0.00	-61.9	162.5	-61.0	147.2
	exo/endo	-1303.043128	5.64	-56.5	161.8	-99.4	166.8
	exo/exo	-1303.044861	1.09	-56.4	165.2	-60.7	144.9
**ProPro**	endo/endo	-917.929142	0.00	-70.7	157.7	-65.5	156.8
	endo/exo	-917.928414	1.91	-70.2	129.2	-58.2	146.1
	exo/endo	-917.927674	3.85	-61.5	149.0	-66.8	155.3
	exo/exo	-917.927832	3.44	-59.5	127.3	-59.4	145.1
**ProHyp**	endo/endo	-993.141497	3.37	-70.9	158.1	-64.4	154.8
	endo/exo	-993.142782	0.00	-70.3	129.7	-58.1	144.8
	exo/endo	-993.139763	7.92	-60.0	127.1	-77.1	156.7
	exo/exo	-993.142202	1.52	-59.7	129.8	-57.9	144.0
**HypPro**	endo/endo	-993.141908	0.90	-69.5	156.4	-65.2	157.2
	endo/exo	-993.141526	1.90	-69.5	129.2	-57.9	146.3
	exo/endo	-993.141850	1.05	-61.0	126.5	-77.8	159.6
	exo/exo	-993.142251	0.00	-60.6	126.7	-59.5	145.2
**HypHyp**	endo/endo	-1068.354298	4.30	-70.0	156.5	-64.6	155.2
	endo/exo	-1068.355935	0.00	-70.4	129.1	-57.8	144.8
	exo/endo	-1068.354258	4.40	-60.8	126.8	-76.5	157.1

Determination of the ring flip preferences in Yyy-position - For ProM1-derivatives, the DFT energy of the *exo*/*endo*-conformer was substracted from the *exo*/*exo*-conformer; for ProM2-derivatives and diproline derivatives (ProPro, ProHyp, HypPro, HypHyp) the DFT energy difference of the *endo*/*exo*- minus the *endo*/*endo*-conformer was formed. The results are presented in Figure 3. For Ac-ProM2-OMe no energy difference could be determined because no *endo* ring flip at Yyy could be found in the initial conformational search.

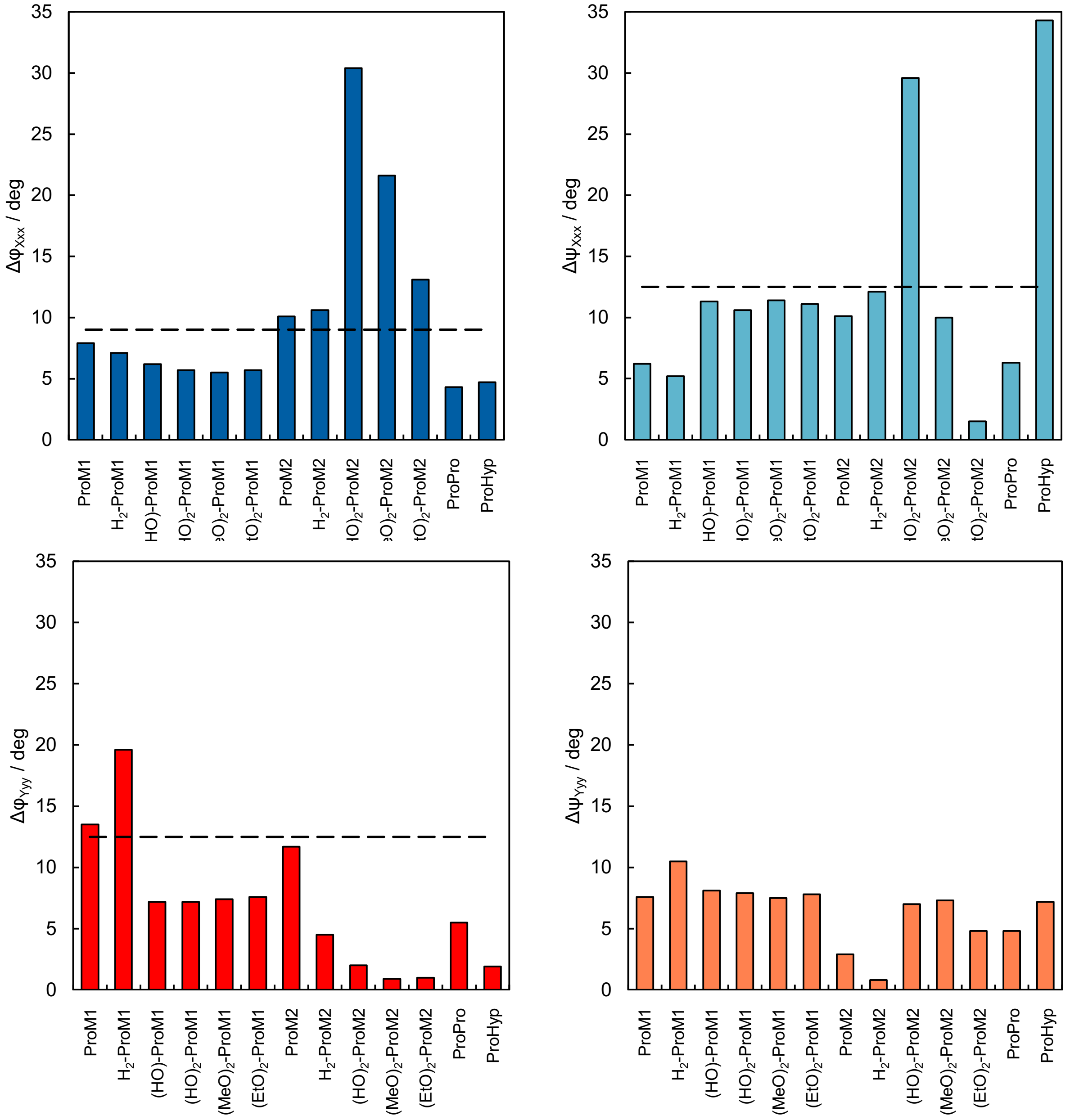

Figure S10. Deviation of main chain torsional angles of DFT-calculated lowest-energy Ac-[dipeptide]-OH structures from averaged main chain values of $(PPG)_{10}$ as reference.[18] Significant deviation was indicated with dashed lines (threshold: $\Delta\varphi_{Xxx}$ = 9°, $\Delta\psi_{Xxx}$ = 12.5° and $\Delta\varphi_{Yyy}$ = 12.5°), except for ψ_{Yyy} due to general C-terminal flexibility and similarity of values.

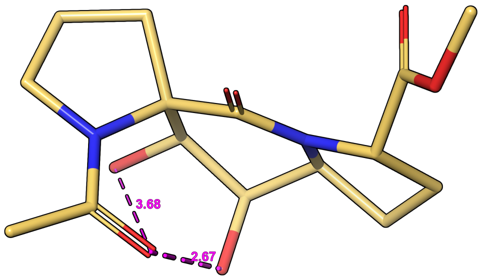

Figure S11. DFT-optimized structure of *exo*/*exo*-Ac-**$(HO)_2$-ProM2**-OMe and indicated O-O-distances (3.68 and 2.67 Å) for intramolecular H bond(s).

Ranking Procedure

Theoretical results (DFT-optimized geometries, alignments) of the Ac-[dipeptide]-OMe systems were assessed with respect to the ability of adopting a collagen-like conformation. The results were compared in four categories (main chain, ring flip, adaptability and sterics) and ranked on the basis of significant differences. "+" means above average, "o" average, "-" below average in terms of triple helix suitability.

(1) Main chain: Differences of the calculated torsional angles from the reference triple helix $(PPG)_{10}$ (φ_{Xxx} = -75°, ψ_{Xxx} = 164° and φ_{Yyy} = -60°; PDB: 1k6f) were determined. A threshold of $\Delta\varphi_{Xxx}$ = 9°, $\Delta\psi_{Xxx}$ = 12.5° and $\Delta\varphi_{Yyy}$ = 12.5° was set to identify significant deviation (see Figure S10). No deviation in any torsion angle was judged as "+", one deviation as "o", two as "-"and in case of two deviations >25° as "--".

(2) Ring flip: The *exo* ring flip propensity at Yyy-position was calculated for each dipeptide (relative energy of *exo*/*exo*-conformer minus *exo*/*endo*-conformer for ProM1-derivatives; *endo*/*exo*-conformer minus *endo*/*endo*-conformer for all other diproline derivatives, see Figure 3). An *endo* propensity $\Delta E_{exo\text{-}endo} > 0$ was judged as "-", minor *exo*-preference ($0 > \Delta E_{exo\text{-}endo} > -2$ kJmol^{-1}) as "o" and major *exo*-preference ($\Delta E_{exo\text{-}endo} < -2$ kJmol^{-1}) as "+". If no conformer with *endo*-flip at Yyy could be found, a "+" was given.

(3) Adaptability: Proposed correction term to account for the ability to undergo small changes in torsional angles without developing significant ring strain. Thus, tricyclic ProMs were considered as "o" and unbridged dipeptides as "+".

(4) Sterics: Lowest-energy conformers were aligned with $(PPG)_{10}$. Sterical clashes with neighbored strands were defined as "--", whereas absence of sterical clashes was defined as "o".

The results from the ranking categories were summed up and presented in Table 1.

X-ray Crystal Structure Parameters of ProM-derivatives

Crystallization - X-ray measurements of the dipeptides were performed with a *Bruker* D8 Venture diffractometer. Single crystals were obtained by vapor diffusion of *n*-heptane into an EtOAc-solution of the sample.

Structures

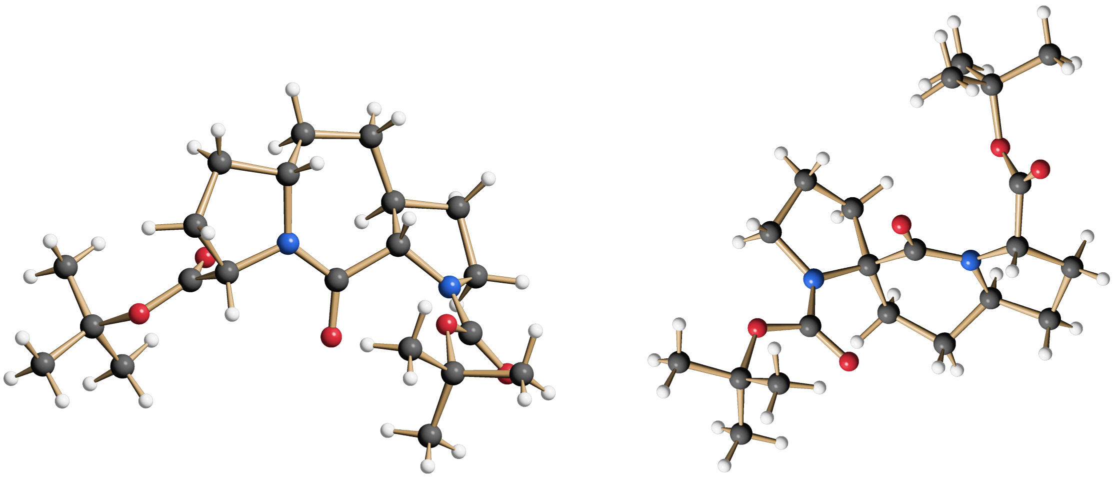

Figure S12. Boc-**H$_2$-ProM1**-O*t*Bu (left) and Boc-**H$_2$-ProM2**-O*t*Bu (right) in the crystalline state.

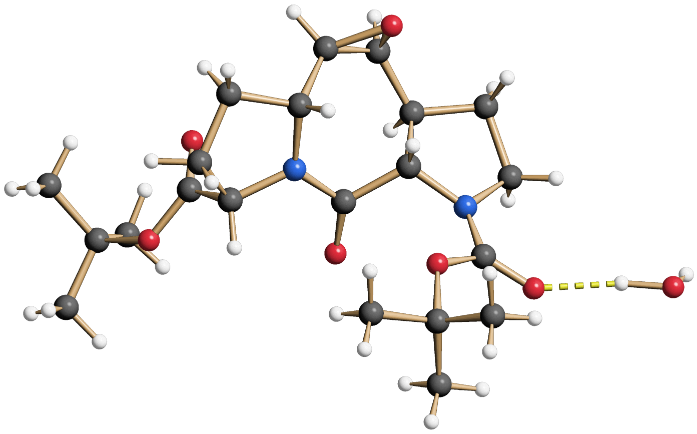

Figure S13. Boc-**ep-ProM1**-O*t*Bu in the crystalline state.

Parameters

Table S4a. Crystal data and structure refinement for Boc-**H_2-ProM1**-O*t*Bu.

Empirical formula	C21 H34 N2 O5	
Moiety formula	C21 H34 N2 O5	
Formula weight	394.50	
Temperature	100(2) K	
Wavelength	1.54178 Å	
Crystal system	Orthorhombic	
Space group	$P2_12_12_1$	
Unit cell dimensions	a = 6.7571(2) Å	a= 90°.
	b = 14.5506(5) Å	b= 90°.
	c = 23.3180(7) Å	g = 90°.
Volume	2292.62(12) $Å^3$	
Z	4	
Density (calculated)	1.143 Mg/m^3	
Absorption coefficient	0.659 mm^{-1}	
F(000)	856	
Crystal size	0.250 x 0.200 x 0.050 mm^3	
Theta range for data collection	3.580 to 72.437°.	
Index ranges	-7<=h<=8, -17<=k<=15, -28<=l<=28	
Reflections collected	23724	
Independent reflections	4506 [R(int) = 0.0423]	
Completeness to theta = 67.679°	100.0 %	
Absorption correction	Semi-empirical from equivalents	
Max. and min. transmission	0.7536 and 0.5431	
Refinement method	Full-matrix least-squares on F^2	
Data / restraints / parameters	4506 / 0 / 259	
Goodness-of-fit on F^2	1.073	
Final R indices [I>2sigma(I)]	R1 = 0.0506, wR2 = 0.1186	
R indices (all data)	R1 = 0.0546, wR2 = 0.1213	
Absolute structure parameter	0.06(8)	
Extinction coefficient	n/a	
Largest diff. peak and hole	0.396 and -0.352 e.$Å^{-3}$	

Table S4b. Crystal data and structure refinement for Boc-**H_2-ProM2**-O*t*Bu.

Empirical formula	C21 H34 N2 O5	
Moiety formula	C21 H34 N2 O5	
Formula weight	394.50	
Temperature	100(2) K	
Wavelength	1.54178 Å	
Crystal system	Monoclinic	
Space group	$P2_1$	
Unit cell dimensions	a = 6.2794(2) Å	a= 90°.
	b = 14.4643(4) Å	b= 100.1970(10)°.
	c = 12.2286(3) Å	g = 90°.
Volume	1093.15(5) Å^3	
Z	2	
Density (calculated)	1.199 Mg/m^3	
Absorption coefficient	0.691 mm^{-1}	
F(000)	428	
Crystal size	0.200 x 0.200 x 0.100 mm^3	
Theta range for data collection	3.672 to 72.182°.	
Index ranges	-7<=h<=7, -17<=k<=17, -15<=l<=15	
Reflections collected	39100	
Independent reflections	4315 [R(int) = 0.0474]	
Completeness to theta = 67.679°	99.9 %	
Absorption correction	Semi-empirical from equivalents	
Max. and min. transmission	0.7536 and 0.5511	
Refinement method	Full-matrix least-squares on F^2	
Data / restraints / parameters	4315 / 1 / 259	
Goodness-of-fit on F^2	1.070	
Final R indices [I>2sigma(I)]	R1 = 0.0269, wR2 = 0.0645	
R indices (all data)	R1 = 0.0277, wR2 = 0.0651	
Absolute structure parameter	0.00(4)	
Extinction coefficient	n/a	
Largest diff. peak and hole	0.136 and -0.220 e.Å^{-3}	

Table S4c. Crystal data and structure refinement for Boc-**ep-ProM1**-O*t*Bu.

Empirical formula	C21 H34 N2 O7	
Moiety formula	C21 H32 N2 O6, H2 O	
Formula weight	426.50	
Temperature	100(2) K	
Wavelength	1.54178 Å	
Crystal system	Orthorhombic	
Space group	$P2_12_12_1$	
Unit cell dimensions	a = 10.8849(2) Å	a= 90°.
	b = 11.5201(2) Å	b= 90°.
	c = 18.2766(4) Å	g = 90°.
Volume	2291.80(8) $Å^3$	
Z	4	
Density (calculated)	1.236 Mg/m^3	
Absorption coefficient	0.765 mm^{-1}	
F(000)	920	
Crystal size	0.400 x 0.300 x 0.200 mm^3	
Theta range for data collection	4.537 to 72.225°.	
Index ranges	-13<=h<=12, -14<=k<=14, -22<=l<=17	
Reflections collected	26900	
Independent reflections	4514 [R(int) = 0.0520]	
Completeness to theta = 67.679°	100.0 %	
Absorption correction	Semi-empirical from equivalents	
Max. and min. transmission	0.7536 and 0.4666	
Refinement method	Full-matrix least-squares on F^2	
Data / restraints / parameters	4514 / 0 / 285	
Goodness-of-fit on F^2	1.090	
Final R indices [I>2sigma(I)]	R1 = 0.0354, wR2 = 0.0907	
R indices (all data)	R1 = 0.0371, wR2 = 0.0919	
Absolute structure parameter	0.01(7)	
Extinction coefficient	n/a	
Largest diff. peak and hole	0.336 and -0.337 e.$Å^{-3}$	

X-ray Crystal Structure Parameters of ProM2-CMP and Structural Alignment

Crystallization of ProM2-CMP and **X-ray structure elucidation** - Aqueous solutions of lyophilized peptide (250 µM with respect to trimer) were mixed with phosphate buffer (1.1 M $(NH_4)_2HPO_4$, 0.1 M Tris, pH = 7.5) in a 1:1 (v/v) ratio. Peptide crystals were grown at RT by vapor diffusion and picked for crystal structural analysis.

Table S6. Data collection and Refinement Parameters for the ProM2-modified CMP.

Parameter	Value
**Data collection**	
Space group	P 2_1
Cell dimensions	
a, *b*, *c* (Å)	26.08, 22.85, 53.43
α, β, γ (°)	90.00, 103.83, 90.00
Wavelength (Å)	0.8266
Resolution (Å)	25.26 - 0.81 (0.839 - 0.81)*
R_{sym} or R_{merge}	0.03824 (0.8166)*
I / σI	17.74 (1.17)*
Completeness (%)	92.26 (55.12)*
Redundancy	6.1 (3.3)*
$CC_{½}$	1 (0.605)*
**Refinement**	
No. reflections	57691 (3409)*
Reflections used for R-free	1994 (119)*
R_{work} / R_{free} (%)	0.128 / 0.136
No. atoms	842
Protein	678
Water	164
B-factors	9.55
Protein	8.37
Heteroatoms	6.61
Water	15.03
Ramachandran	
favored (%)	98.68
allowed (%)	1.32
outliers (%)	0.0
R.m.s. deviations	
Bond lengths (Å)	0.021
Bond angles (°)	1.91

* Values in parentheses are for highest-resolution shell.

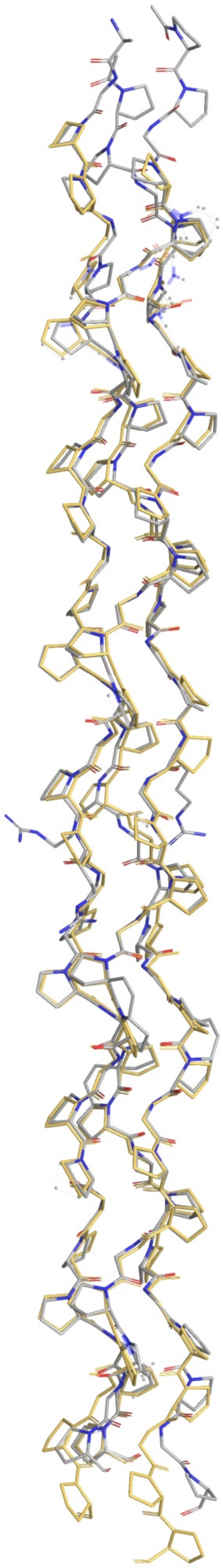

Figure S15. Crystal structural alignment of Cα-atoms of the nine central amino acid triplets in Ac-(PPG)$_5$-PRG-PPG-**ProM2**G-(PPG)$_3$-NH$_2$ (grey) with Cα-atoms of (PPG)$_{10}$ (yellow); RMSD = 0.4557 Å (over Cα-atoms).[18]

HSP47 binding assay and K_D-determination

Expression of HSP47 – HSP47 was essential expressed and purified as before, with some smaller adjustments (Oecal et al. JBC 2016). A canine-derived synthetic HSP47 gene coding for residues $_{36}$LSP…RDEL$_{418}$ was was transformed in *E. coli* BL21 (DE3), cells grown to OD_{600} of 0.8 and protein expression induced with 0.5 mM isopropyl-ß-d-thio-galactoside (IPTG). After 3 h incubation at 37°C / 180 rpm cells were harvested and pellets stored at -20 °C. Cells were suspended in lysis buffer (TBS [50mM Tris•HCl, 150mM NaCl, pH 7.5] + 0.1 mM PMSF, 100µg/ml DNAse I, 0.5 mM DTT) and sonicated on ice. The cleared lysate was purified via Ni-NTA Superflow (Qiagen) resin with several wash steps (TBS, TBS containing 0.5M NaCl (total), TBS + 10 and 50mM Imidazol). After elution with 250mM Imidazol in TBS, the aliquots were directly adjusted to 2 mM DTT (final). To precipitate contaminants ammonium sulphate was added to a final concentration of 1.5 M and the solution cleared by centrifugation. The supernatant was concentrated and subjected to size exclusion chromatography (SEC) using a Superdex 200 increase column (GE Healthcare) in TBS + 2mM DTT. Fractions containing the protein of interest were pooled and the solution stored at -80 °C after flash freezing in liquid N_2.

Biolayer Interferometry – HSP47 binding studies were performed on a BLItz system (forte BIO) using high precision streptavidin SA biosensors (forte BIO). After 10 min re-hydration the biosensors were loaded with solutions of biotinylated peptide (10 µM with respect to trimer) for 60 s. For kinetic measurements, the biosensors were treated with fresh solutions of HSP47 (90 nM, 180 nM, 360 nM, 470 nM, 710 nM and 1.4 µM) for 60 s. Dissociation was carried out in PBS buffer (pH = 7.5, t = 120 s) and regeneration of biosensors in McIlvain buffer (pH = 6, t = 40 s). Unspecific binding and degradation effects of HSP47 could be ruled out by repeating the assay for 1.4 µM HSP47 before and after each dilution series. The spectral response was detected (in nm) and fitted for association and dissociation using a 1:1 Langmuir binding model in *Origin 2018pro*.[19]

Data interpretation

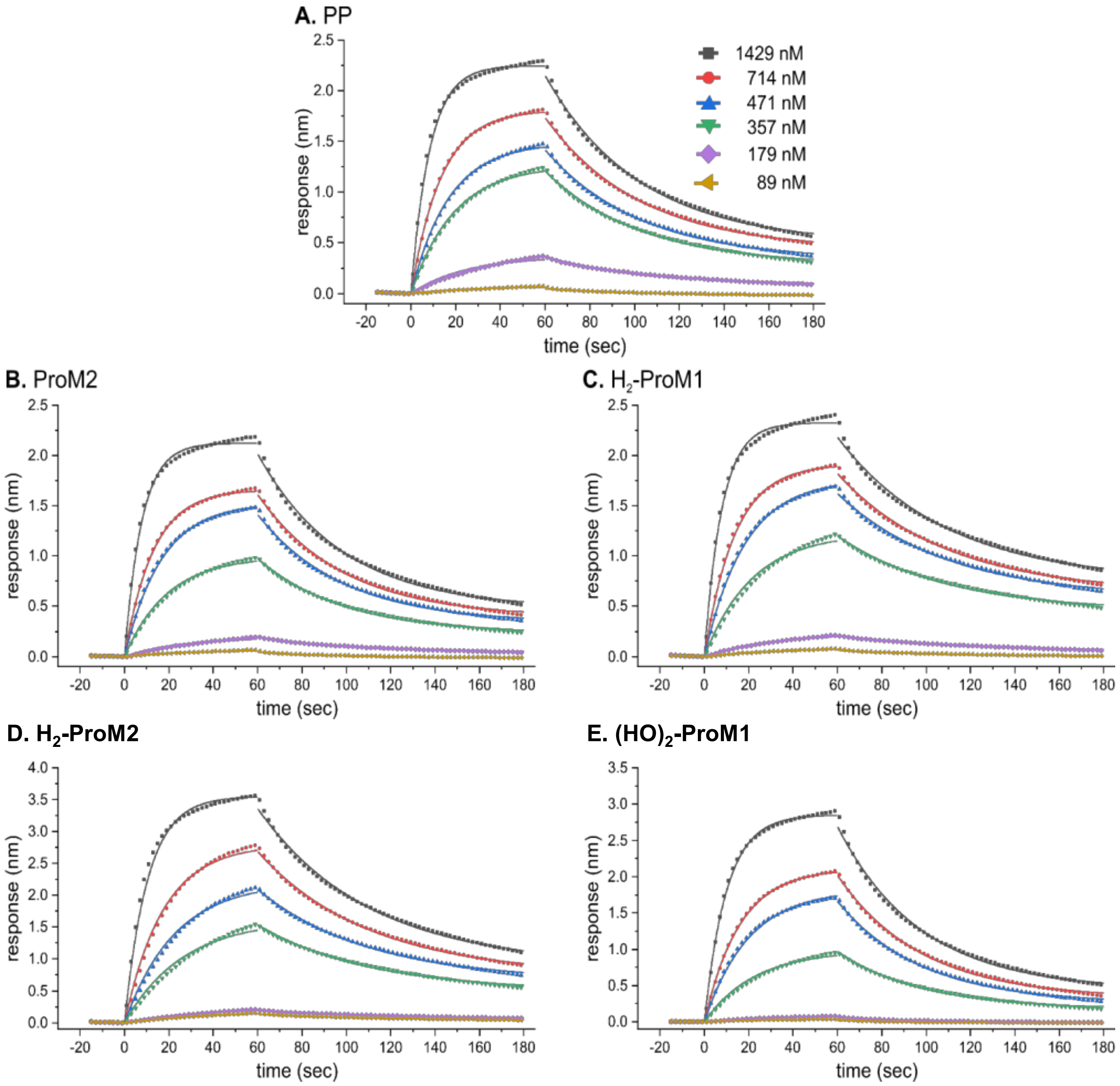

Figure S16. Biolayer Interferometry: Association and Dissociation curves of HSP47 (see color code for concentrations) interacting with immobilized triple-helical peptide (XY = **PP** (A), **ProM2** (B), **H_2-ProM1** (C), **H_2-ProM2** (D), **$(HO)_2$-ProM1** (E)) and fitted with *Origin 2018pro*.[19] For clarity only every 10th datapoint is shown; solid lines indicate fitting curves.

Table S7. Kinetic parameters from BLI curve fitting.

peptide[a], XY =	K_D [nM][b]	k_{off} [s^{-1}]	k_{on} [L $mmol^{-1}s^{-1}$]
PP	370.3 ± 1.8	0.023 ± 7.18E-5	62.10 ± 0.25
ProM2	361.7 ± 1.8	0.026 ± 8.81E-5	71.88 ± 0.26
H_2-ProM2	353.0 ± 2.5	0.019 ± 6.92E-5	53.83 ± 0.32
H_2-ProM1	268.9 ± 1.6	0.020 ± 9.20E-5	74.38 ± 0.30
$(HO)_2$-ProM1	502.3 ± 2.2	0.026 ± 7.70E-5	51.76 ± 0.16

[a] peptide sequence: Biotin-Ebes-$(PPG)_5$PRGPPG[XY]G$(PPG)_3$-NH_2 with XY indicating the (modified) diproline unit. [b] determined by $K_D = k_{off}/k_{on}$; errors were calculated based on the rules of propagation of uncertainty.

NMR spectra

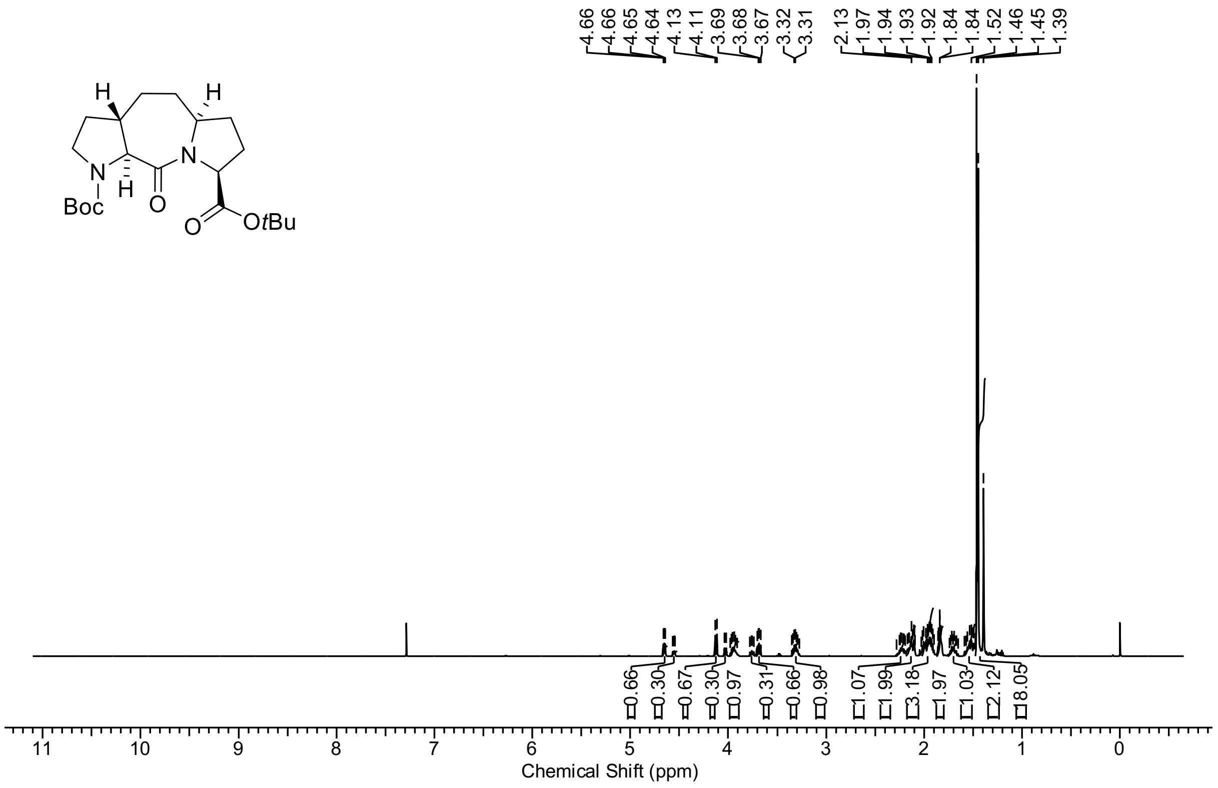

Figure S17. ^{1}H-NMR spectrum of Boc-**H$_2$-ProM1**-O*t*Bu in $CDCl_3$ at 500 MHz (full view).

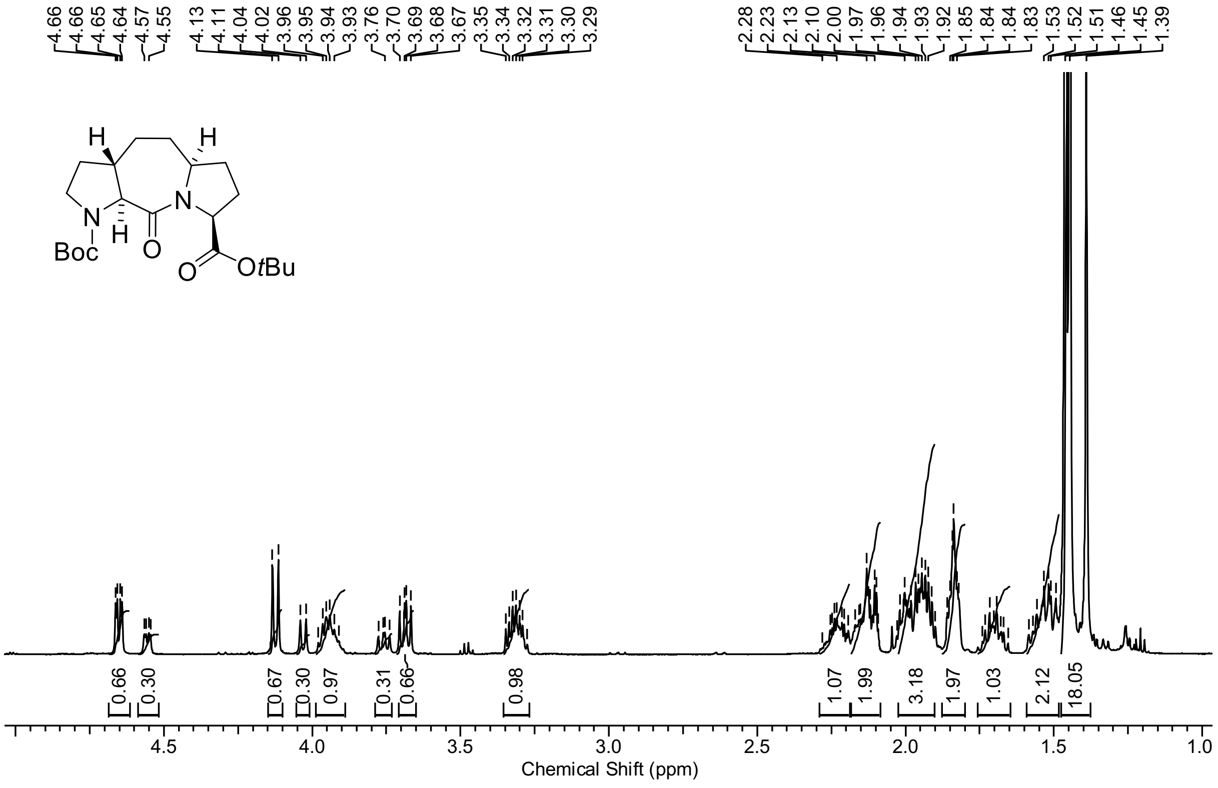

Figure S18. ^{1}H-NMR spectrum of Boc-**H$_2$-ProM1**-O*t*Bu in $CDCl_3$ at 500 MHz (zoom in).

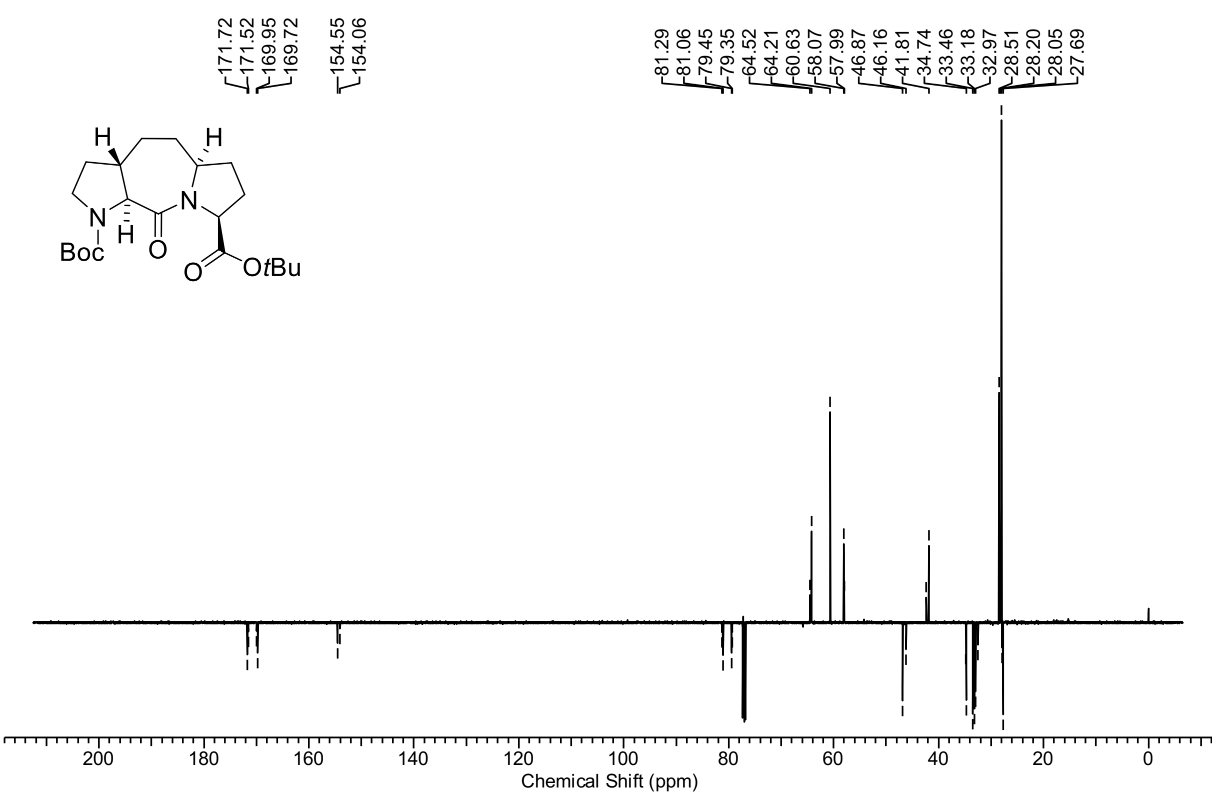

Figure S19. ^{13}C-APT-NMR spectrum of Boc-**H$_2$-ProM1**-O*t*Bu in $CDCl_3$ at 125 MHz.

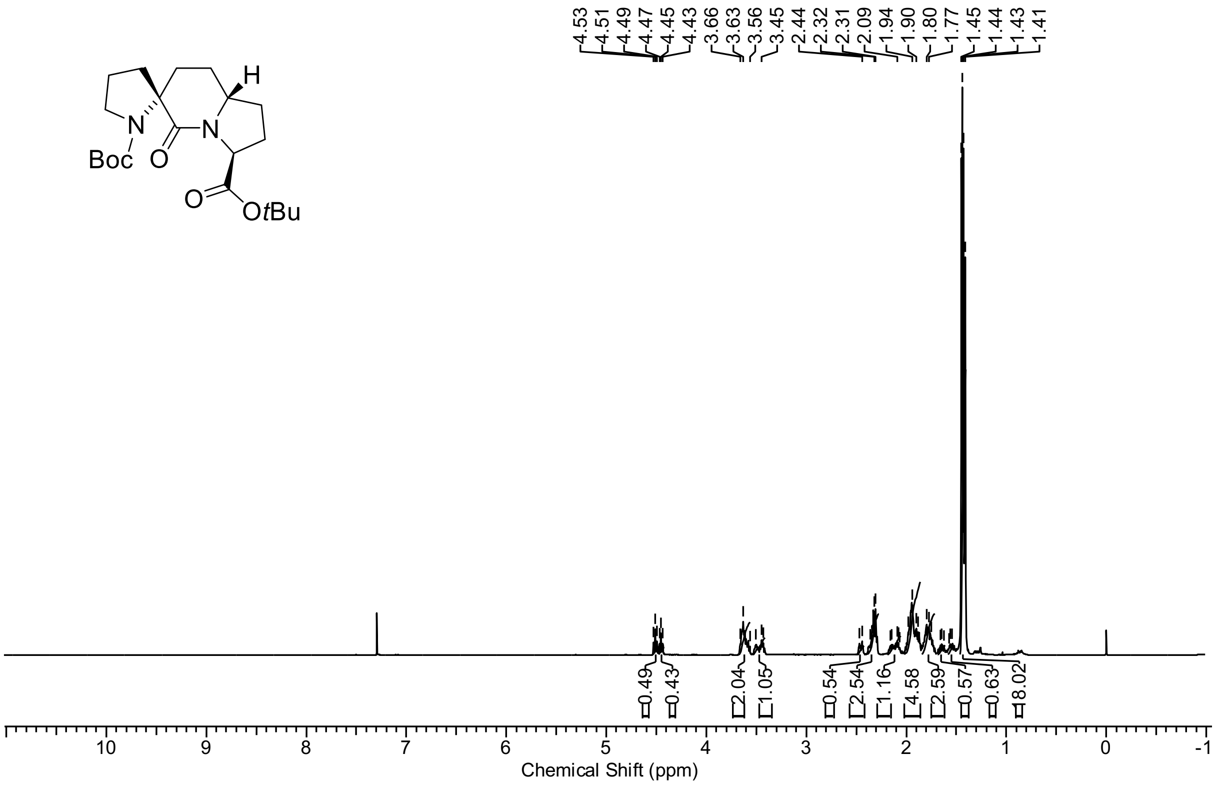

Figure S20. ^{1}H-NMR spectrum of Boc-**H$_2$-ProM2**-O*t*Bu in $CDCl_3$ at 500 MHz (full view).

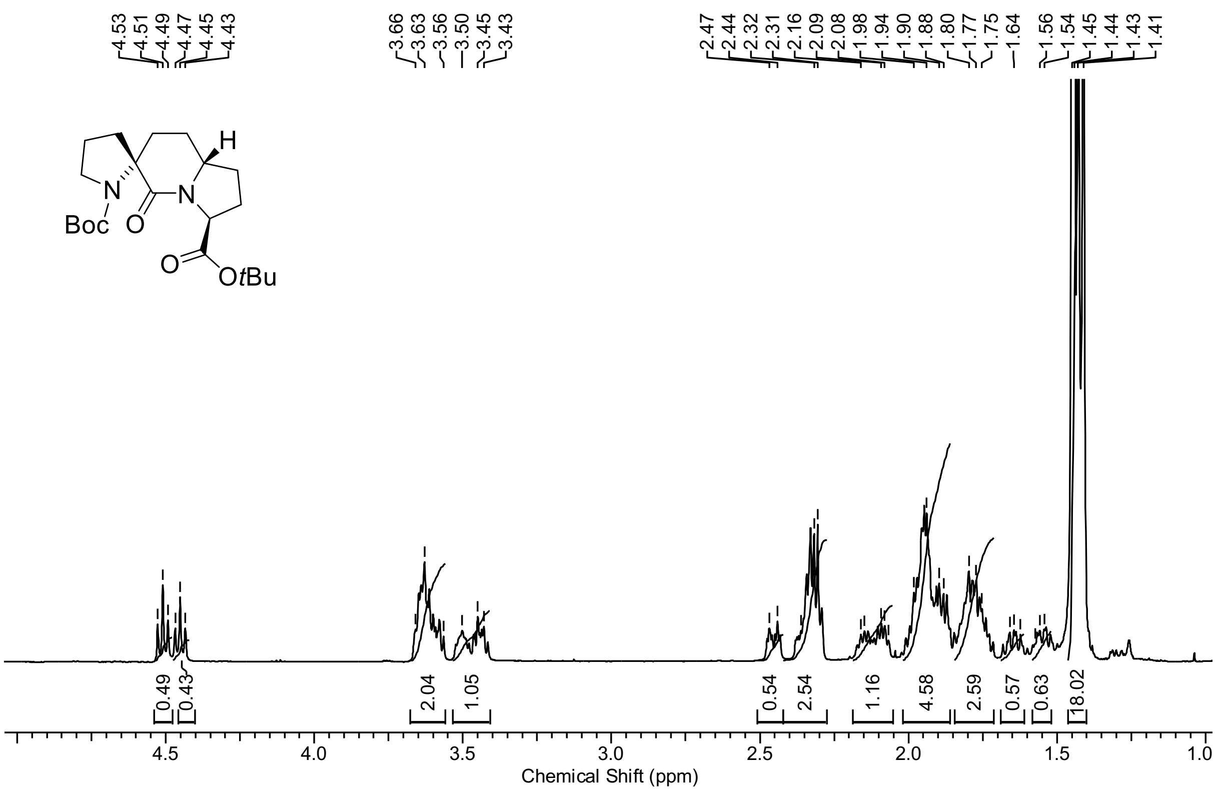

Figure S21. ^{1}H-NMR spectrum of Boc-**H$_2$-ProM2**-O*t*Bu in $CDCl_3$ at 500 MHz (zoom in).

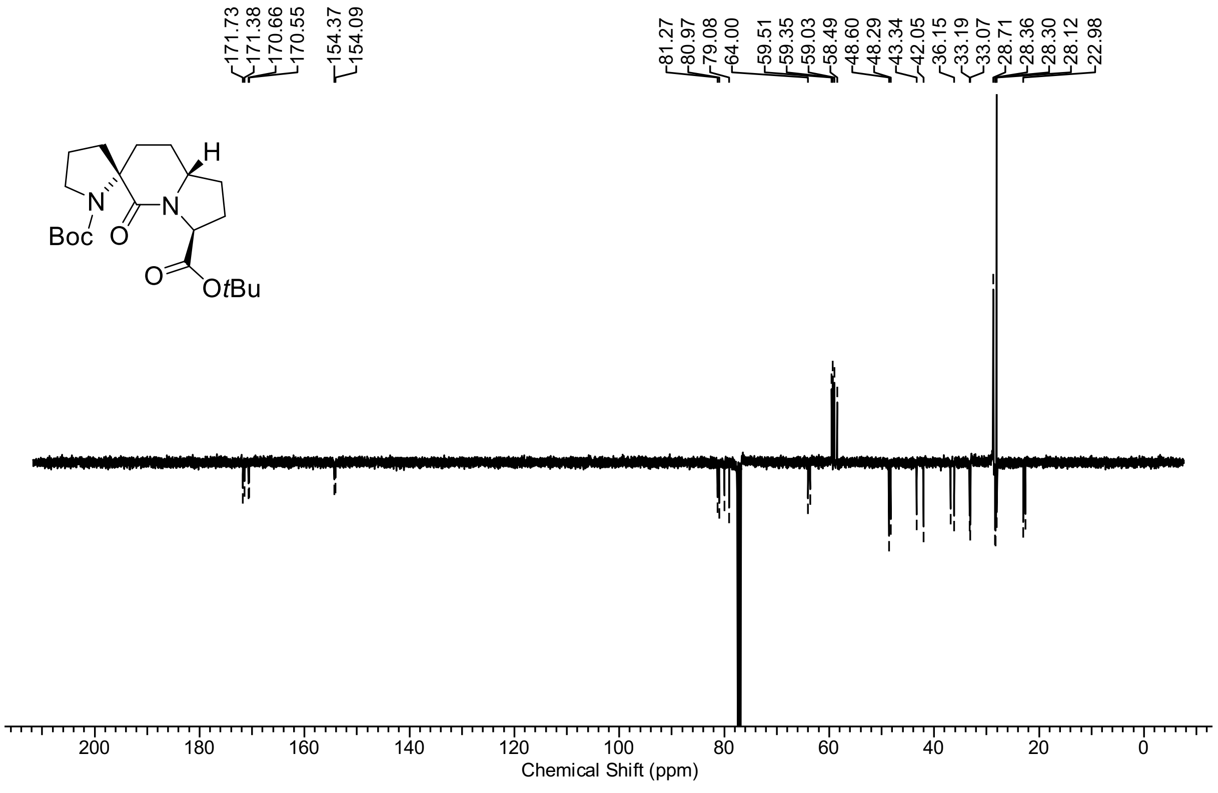

Figure S22. ^{13}C-APT-NMR spectrum of Boc-**H$_2$-ProM2**-O*t*Bu in $CDCl_3$ at 100 MHz.

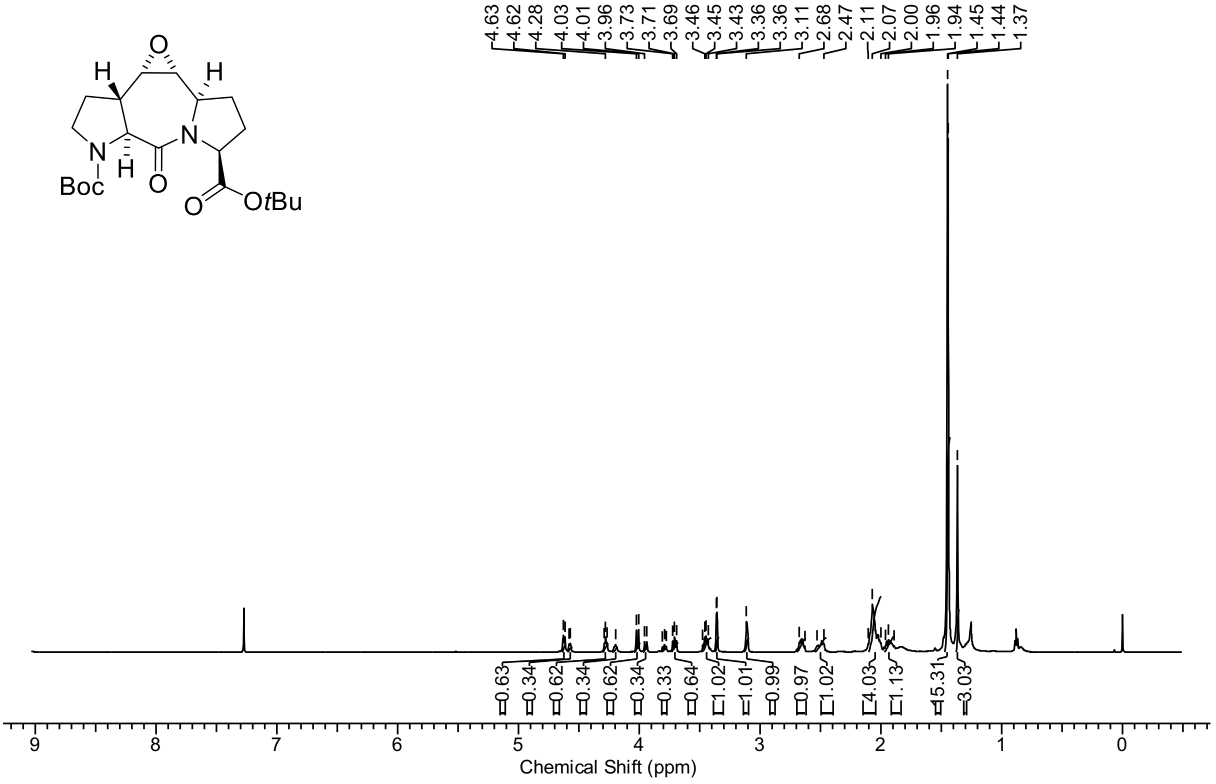

Figure S23. ^{1}H-NMR spectrum of Boc-**ep-ProM1**-O*t*Bu in $CDCl_3$ at 600 MHz (full view).

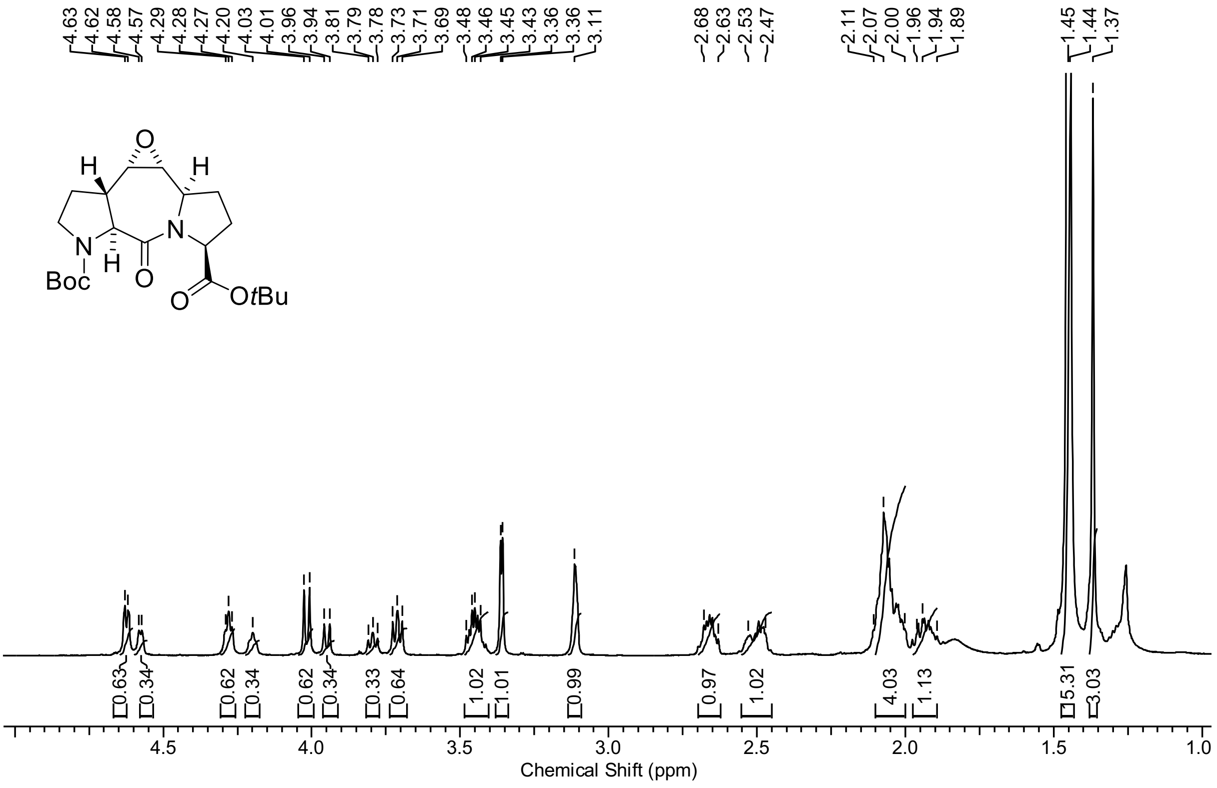

Figure S24. ^{1}H-NMR spectrum of Boc-**ep-ProM1**-O*t*Bu in $CDCl_3$ at 600 MHz (zoom in).

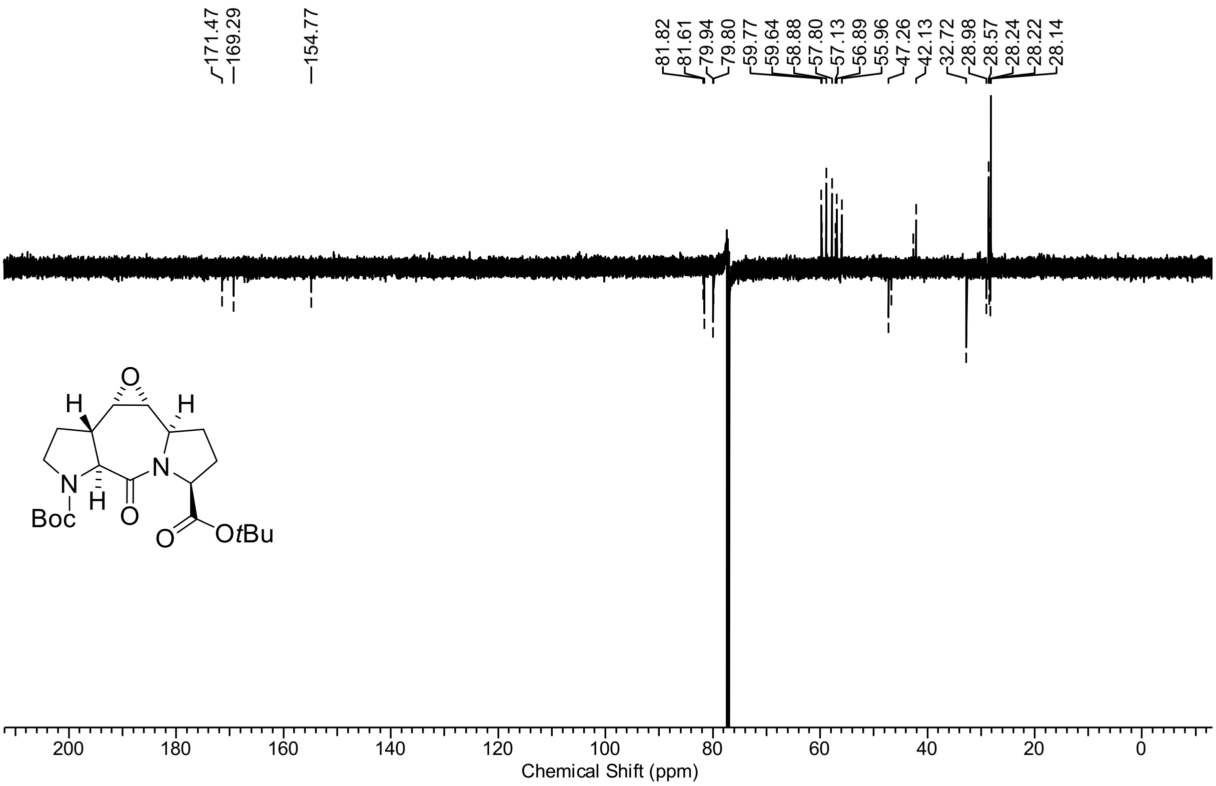

Figure S25. ^{13}C-APT-NMR spectrum of Boc-**ep-ProM1**-O*t*Bu in $CDCl_3$ at 150 MHz.

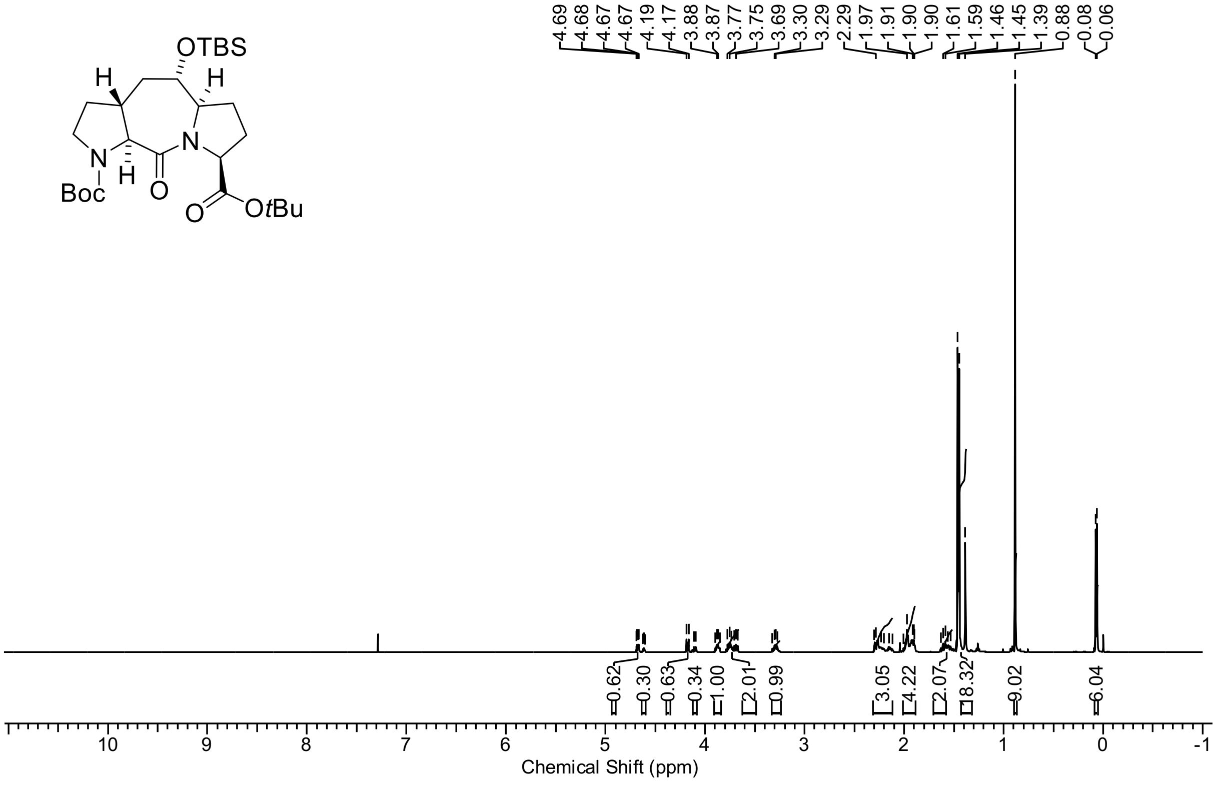

Figure S26. ^{1}H-NMR spectrum of Boc-**TBSO-ProM1**-O*t*Bu in $CDCl_3$ at 500 MHz (full view).

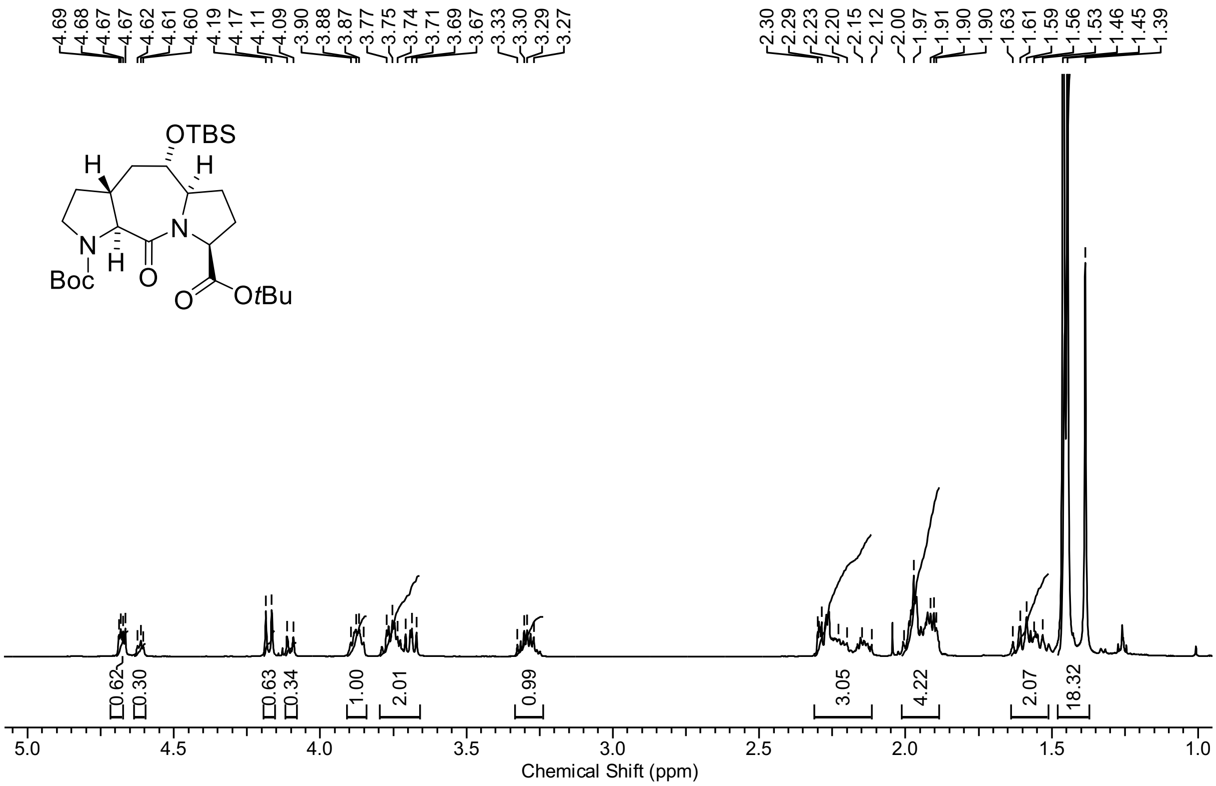

Figure S27. ^{1}H-NMR spectrum of Boc-**TBSO-ProM1**-O*t*Bu in $CDCl_3$ at 500 MHz (zoom in).

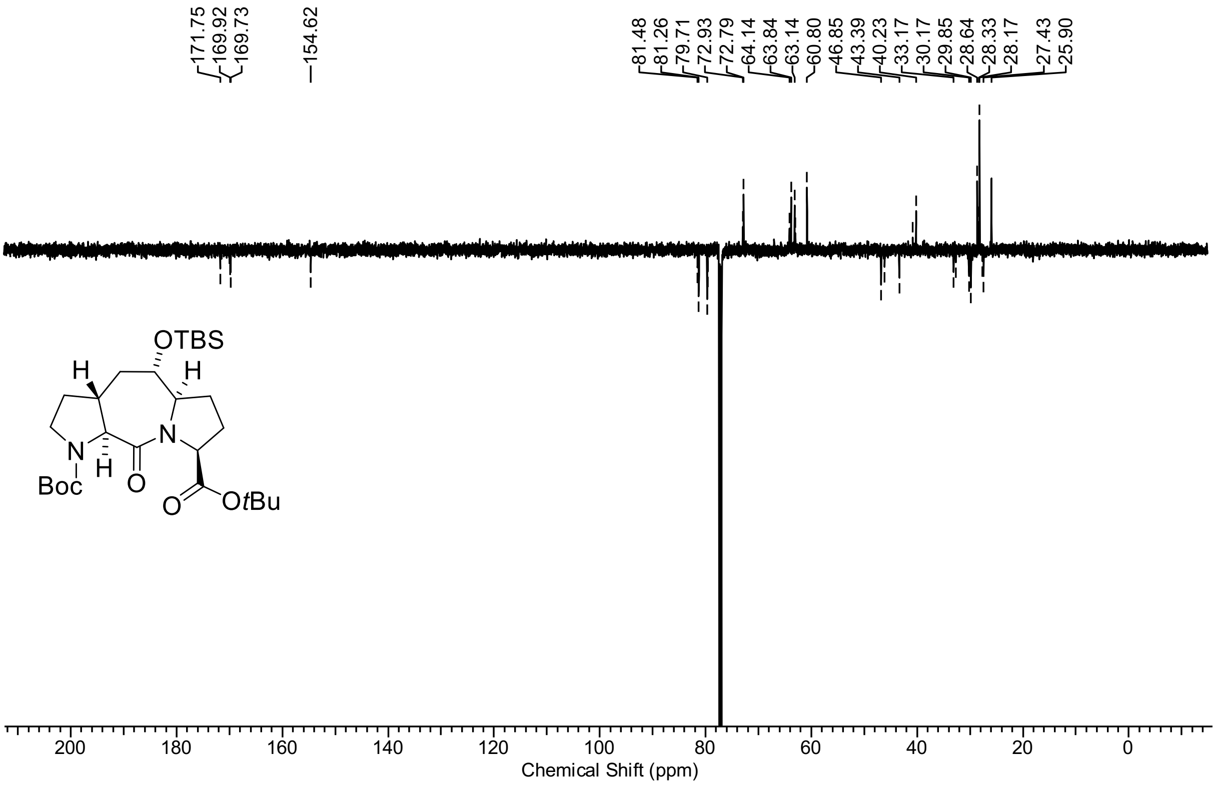

Figure S28. ^{13}C-APT-NMR spectrum of Boc-**TBSO-ProM1**-O*t*Bu in $CDCl_3$ at 150 MHz.

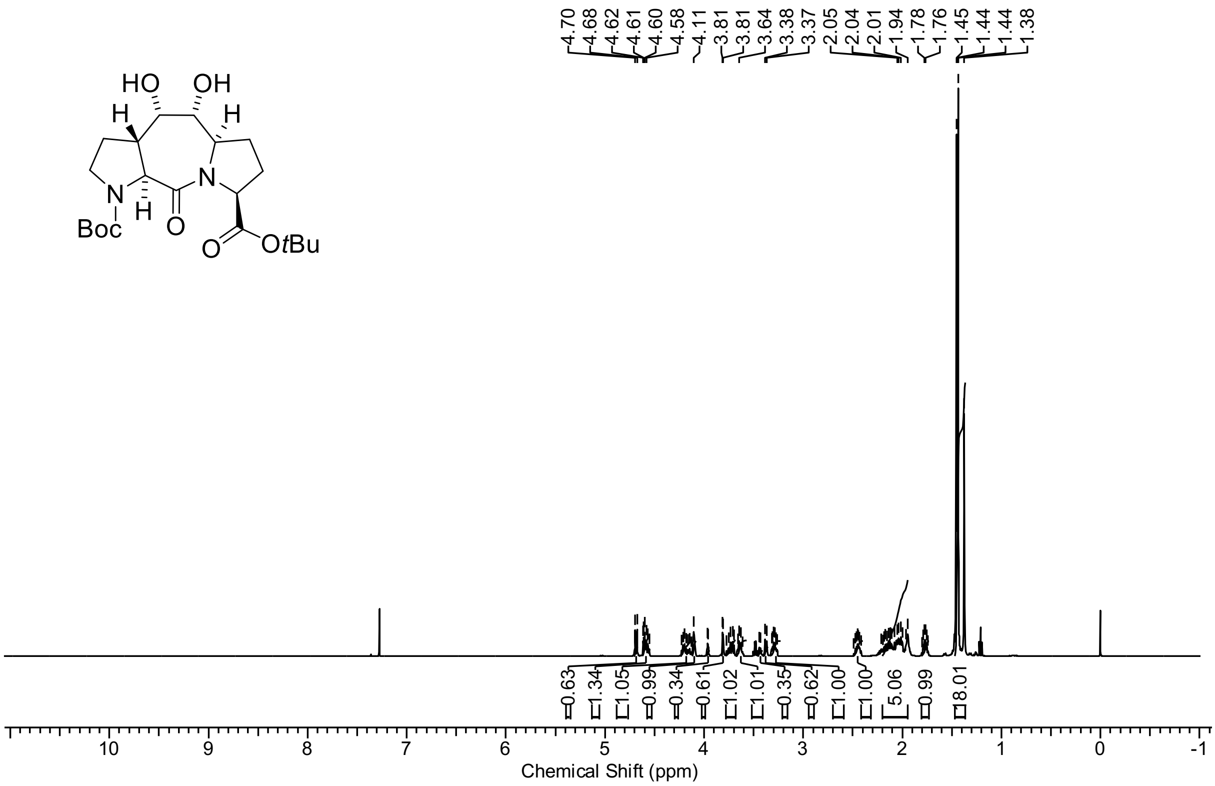

Figure S29. ^{1}H-NMR spectrum of Boc-α-**(HO)$_2$-ProM1**-O*t*Bu in $CDCl_3$ at 500 MHz (full view).

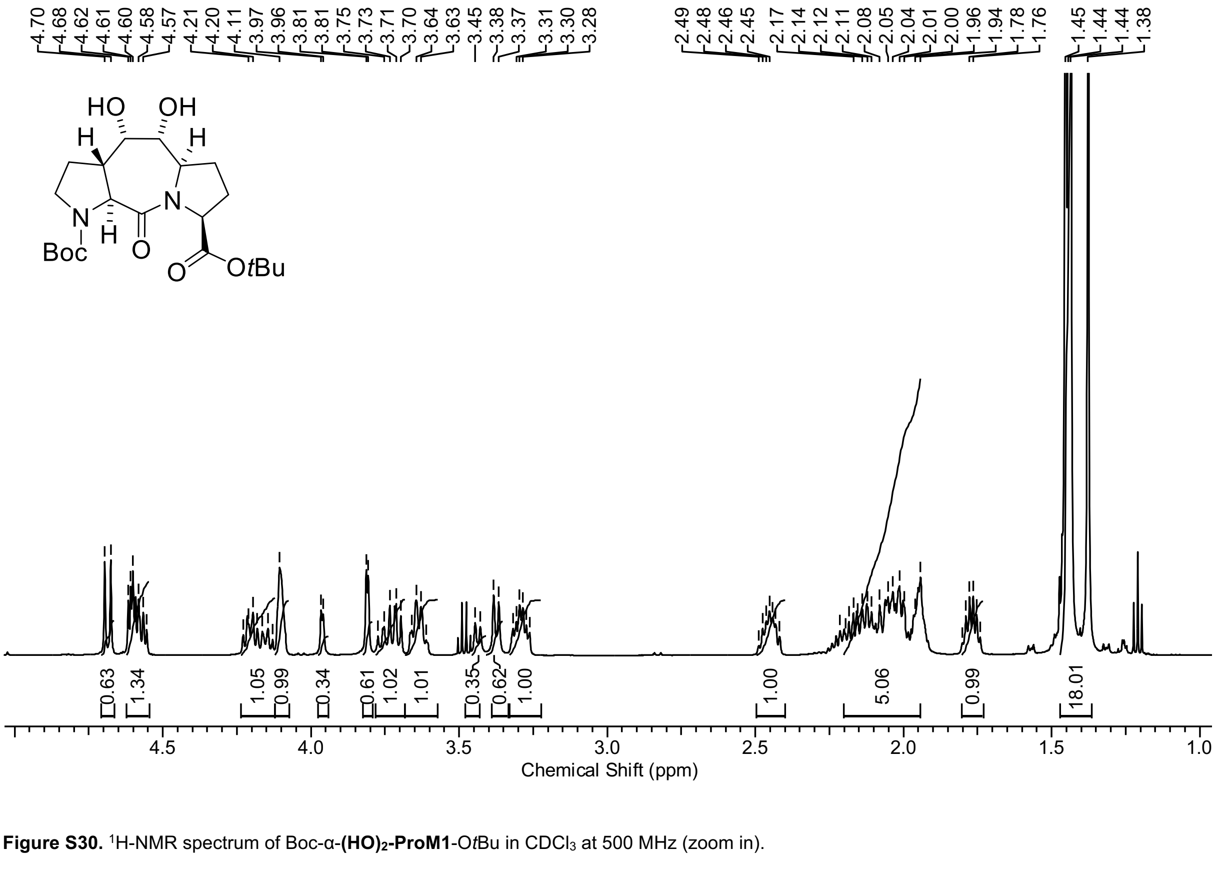

Figure S30. ^{1}H-NMR spectrum of Boc-α-**(HO)$_2$-ProM1**-O*t*Bu in $CDCl_3$ at 500 MHz (zoom in).

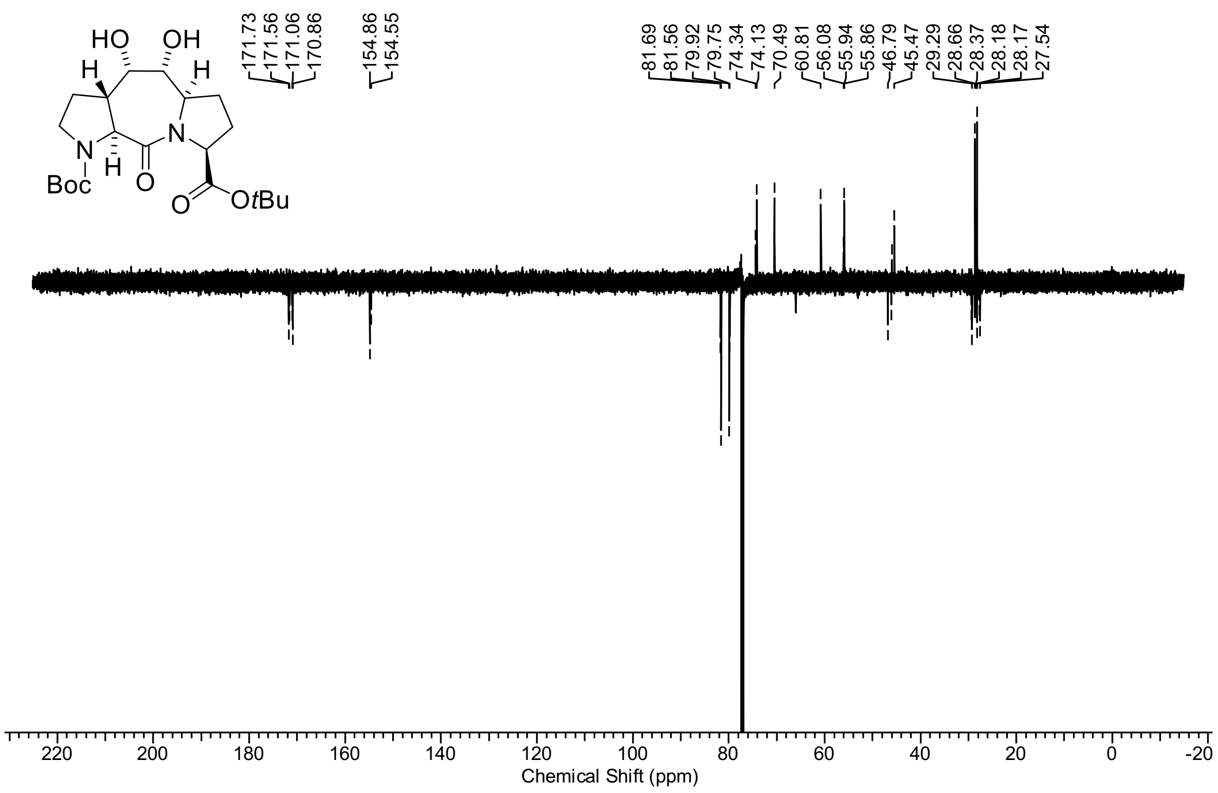

Figure S31. ^{13}C-APT-NMR spectrum of Boc-α-**(HO)$_2$-ProM1**-O*t*Bu in $CDCl_3$ at 150 MHz.

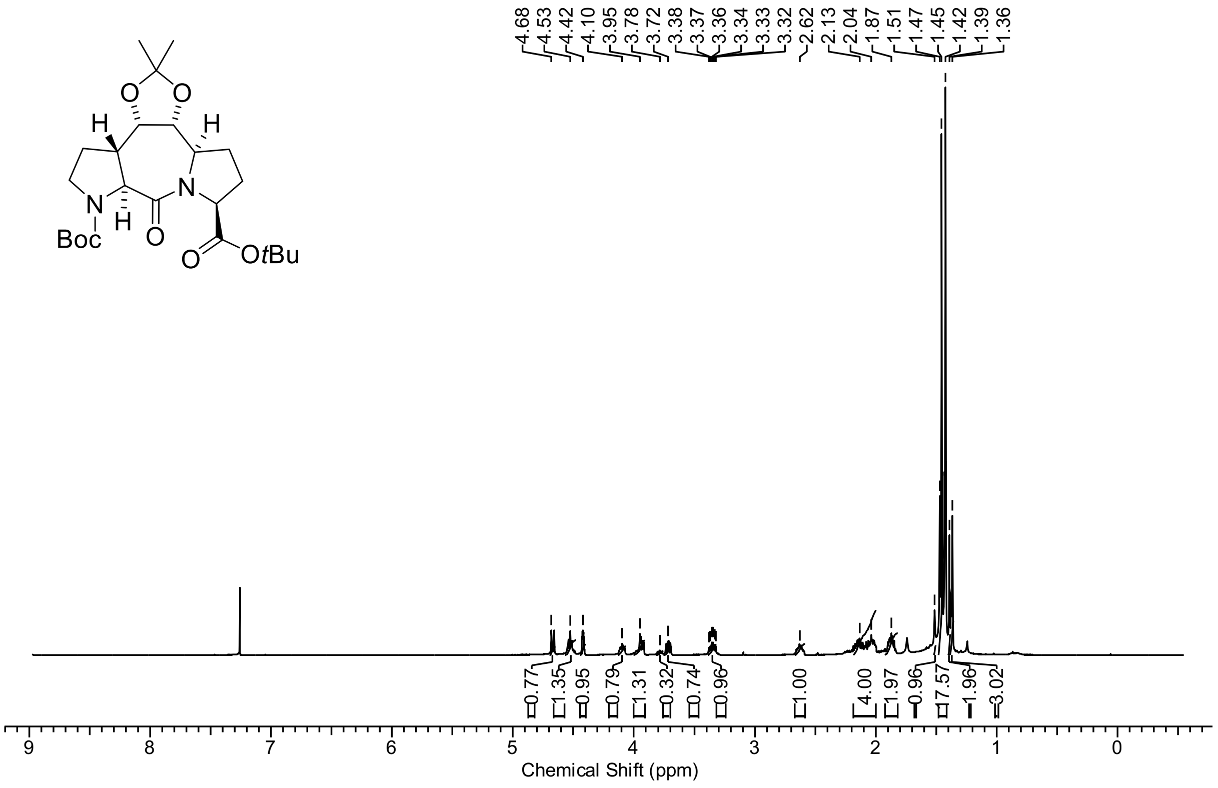

Figure S32. ^{1}H-NMR spectrum of Boc-**(Me$_2$CO$_2$)-ProM1**-O*t*Bu in $CDCl_3$ at 500 MHz (full view).

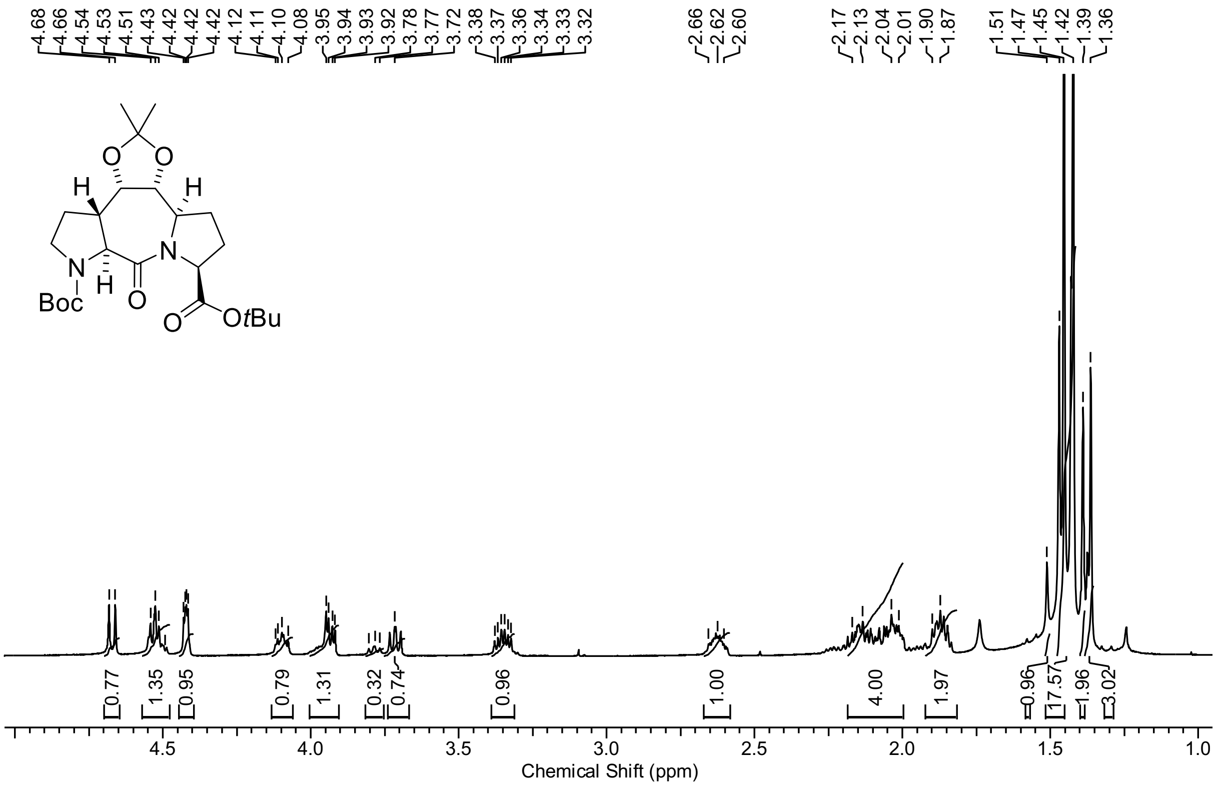

Figure S33. ^{1}H-NMR spectrum of Boc-**(Me$_2$CO$_2$)-ProM1**-O*t*Bu in $CDCl_3$ at 500 MHz (zoom in).

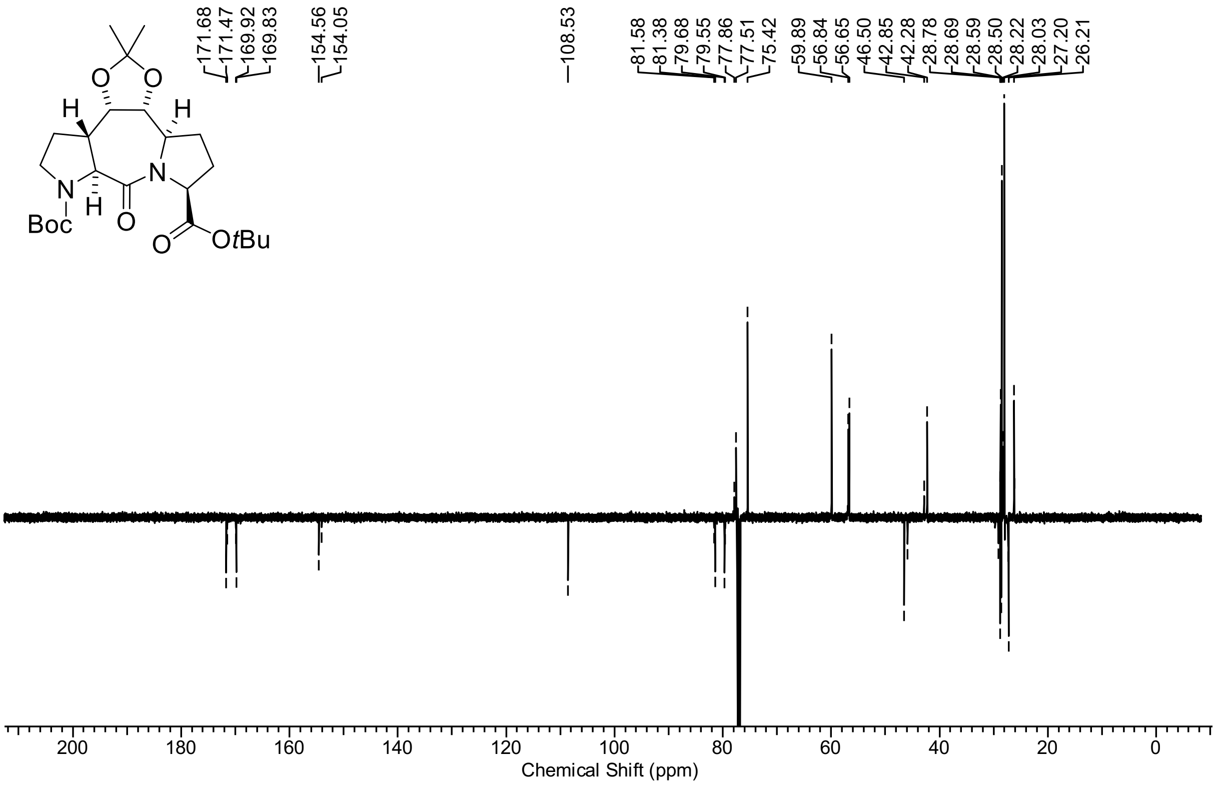

Figure S34. ^{13}C-APT-NMR spectrum of Boc-**(Me$_2$CO$_2$)-ProM1**-O*t*Bu in $CDCl_3$ at 125 MHz.

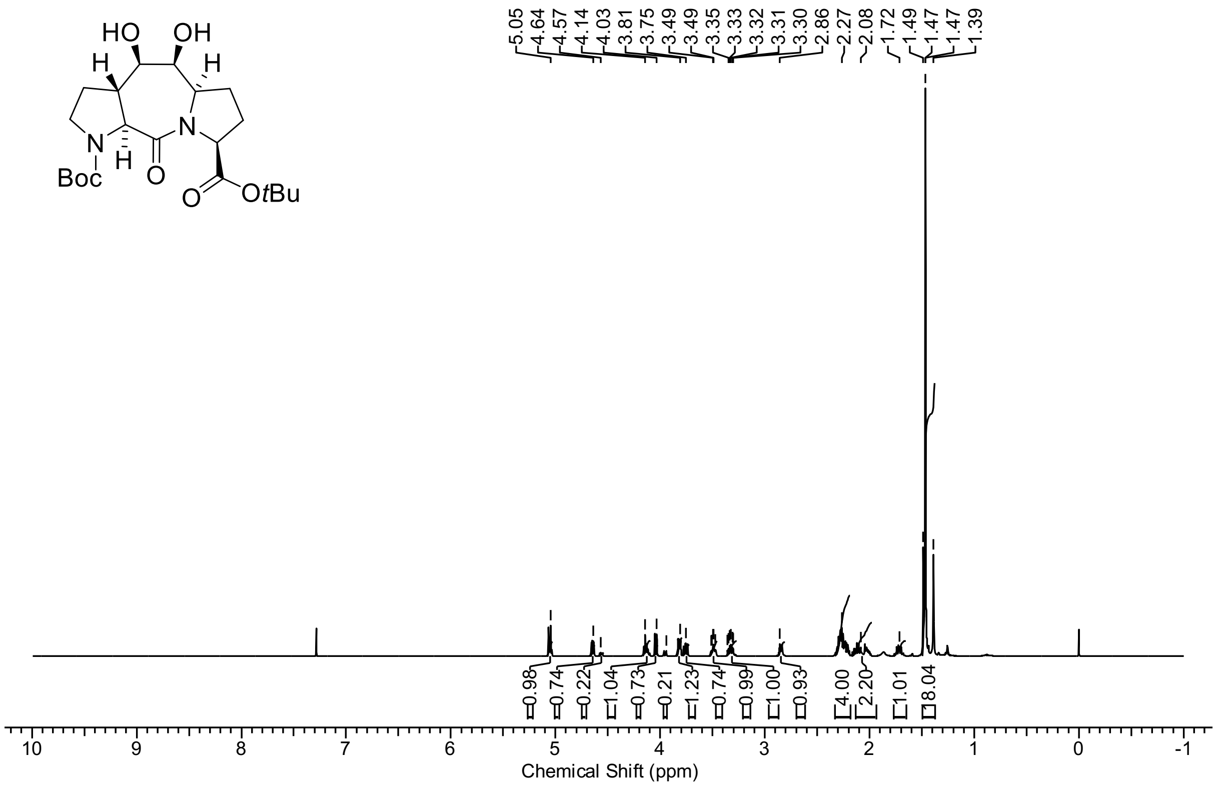

Figure S35. ^{1}H-NMR spectrum of Boc-β-**(HO)$_2$-ProM1**-O*t*Bu in $CDCl_3$ at 500 MHz (full view).

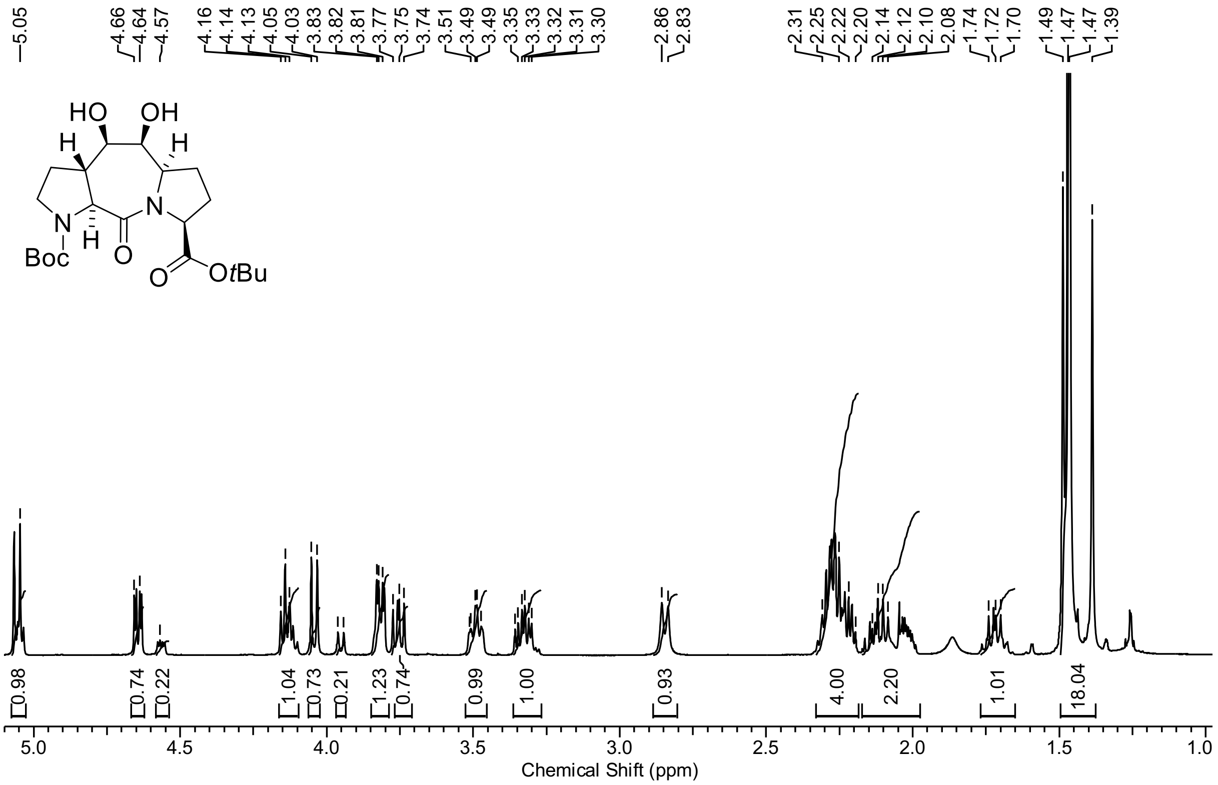

Figure S36. ^{1}H-NMR spectrum of Boc-β-**(HO)$_2$-ProM1**-O*t*Bu in $CDCl_3$ at 500 MHz (zoom in).

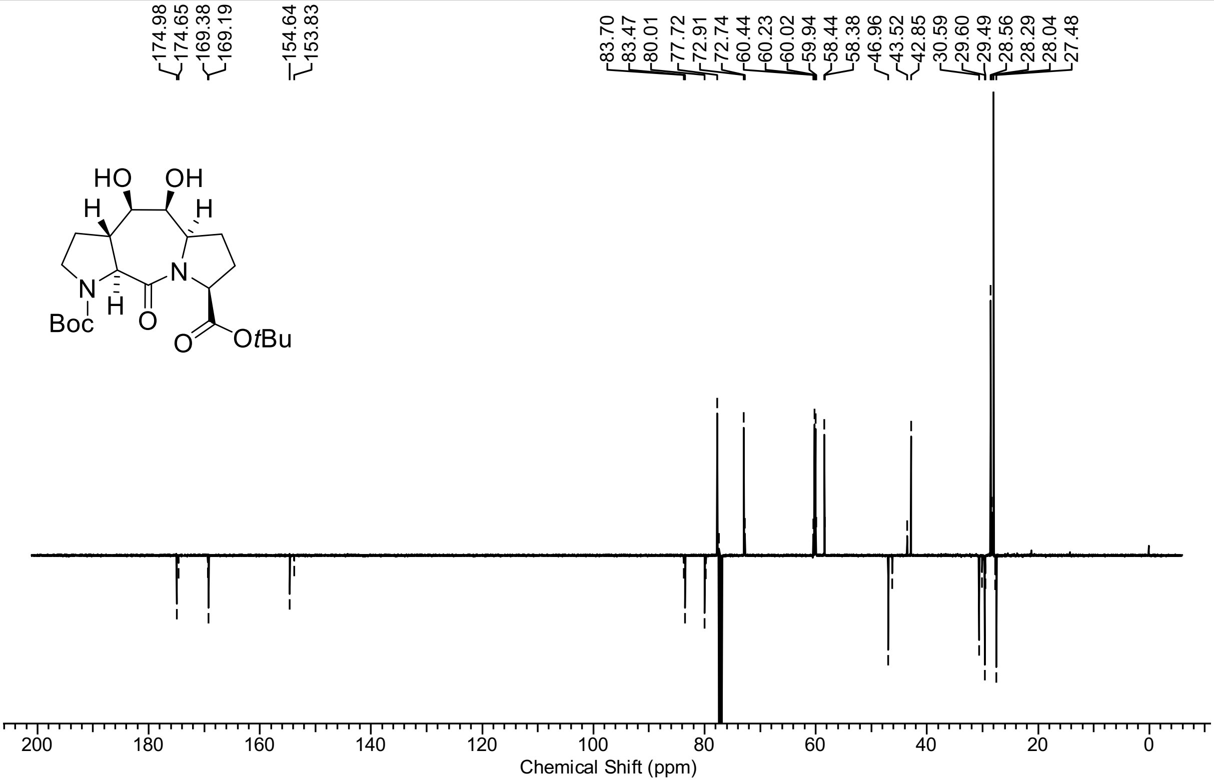

Figure S37. ^{13}C-APT-NMR spectrum of Boc-β-**(HO)₂-ProM1**-O*t*Bu in $CDCl_3$ at 125 MHz.

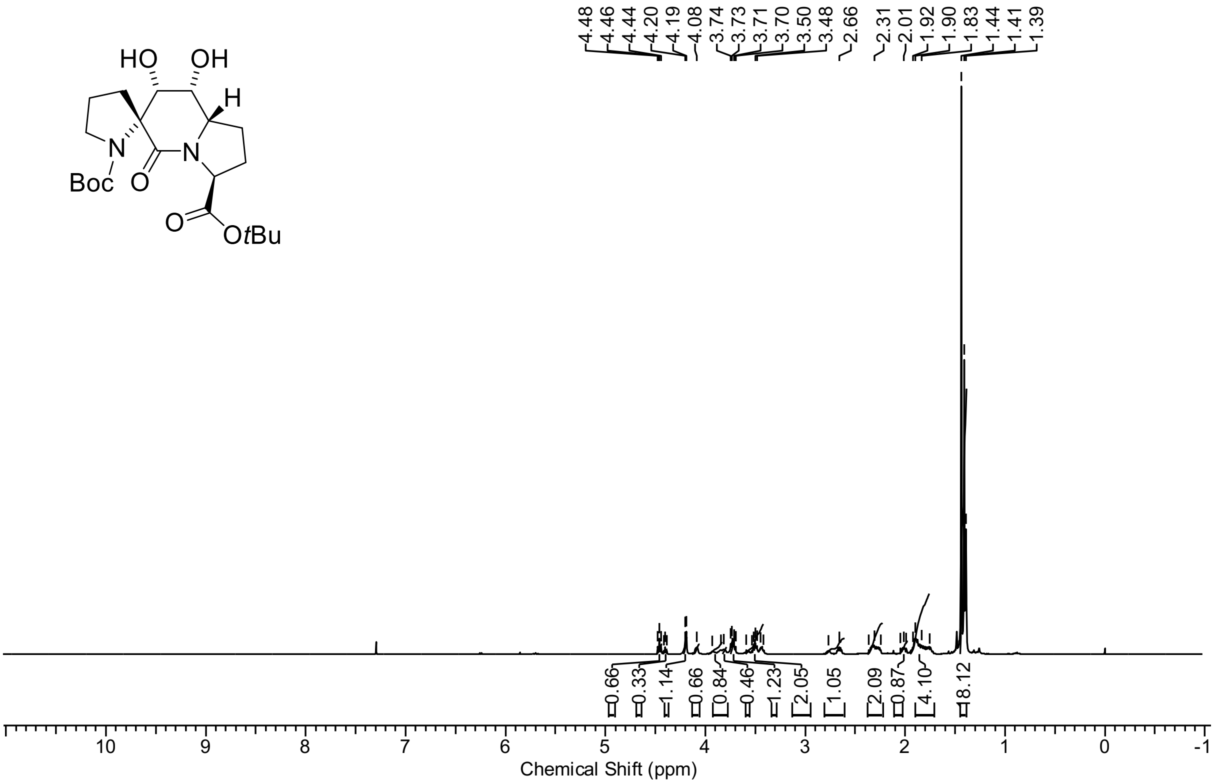

Figure S38. ^{1}H-NMR spectrum of Boc-α-**(HO)₂-ProM2**-O*t*Bu in $CDCl_3$ at 500 MHz (full view).

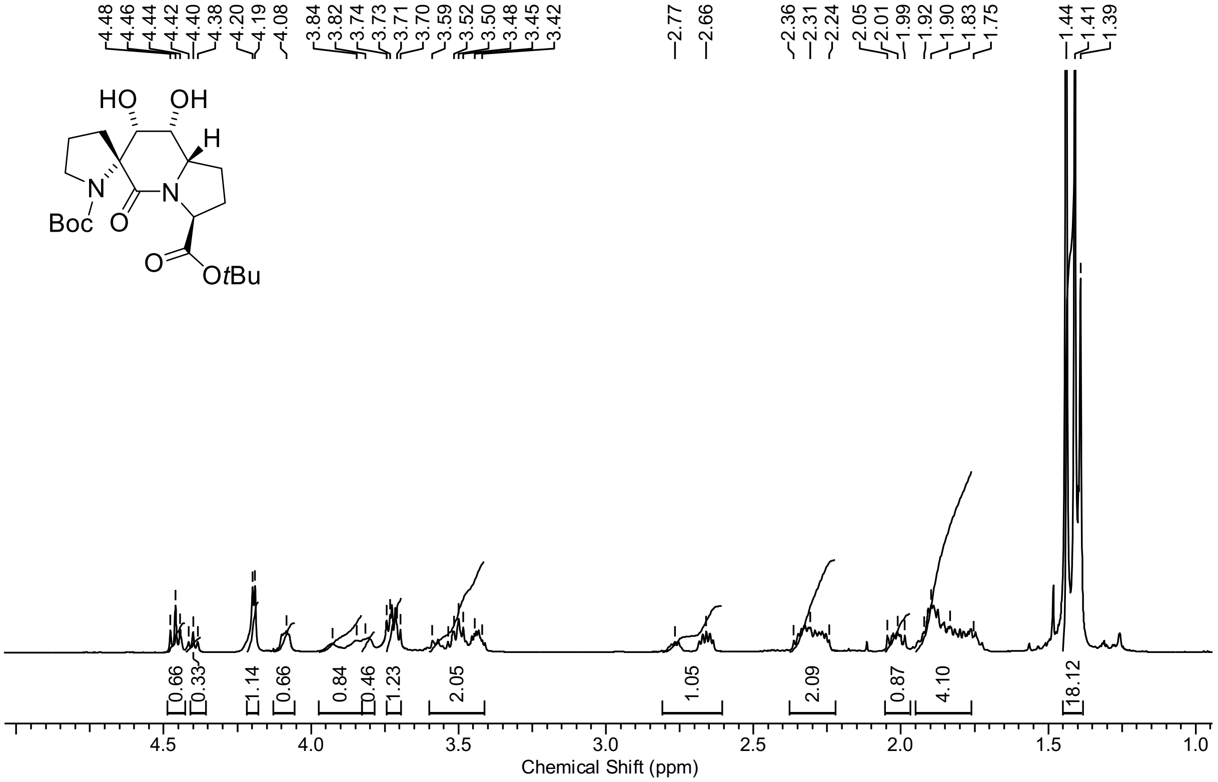

Figure S39. ^{1}H-NMR spectrum of Boc-α-**(HO)$_2$-ProM2**-O*t*Bu in $CDCl_3$ at 500 MHz (zoom in).

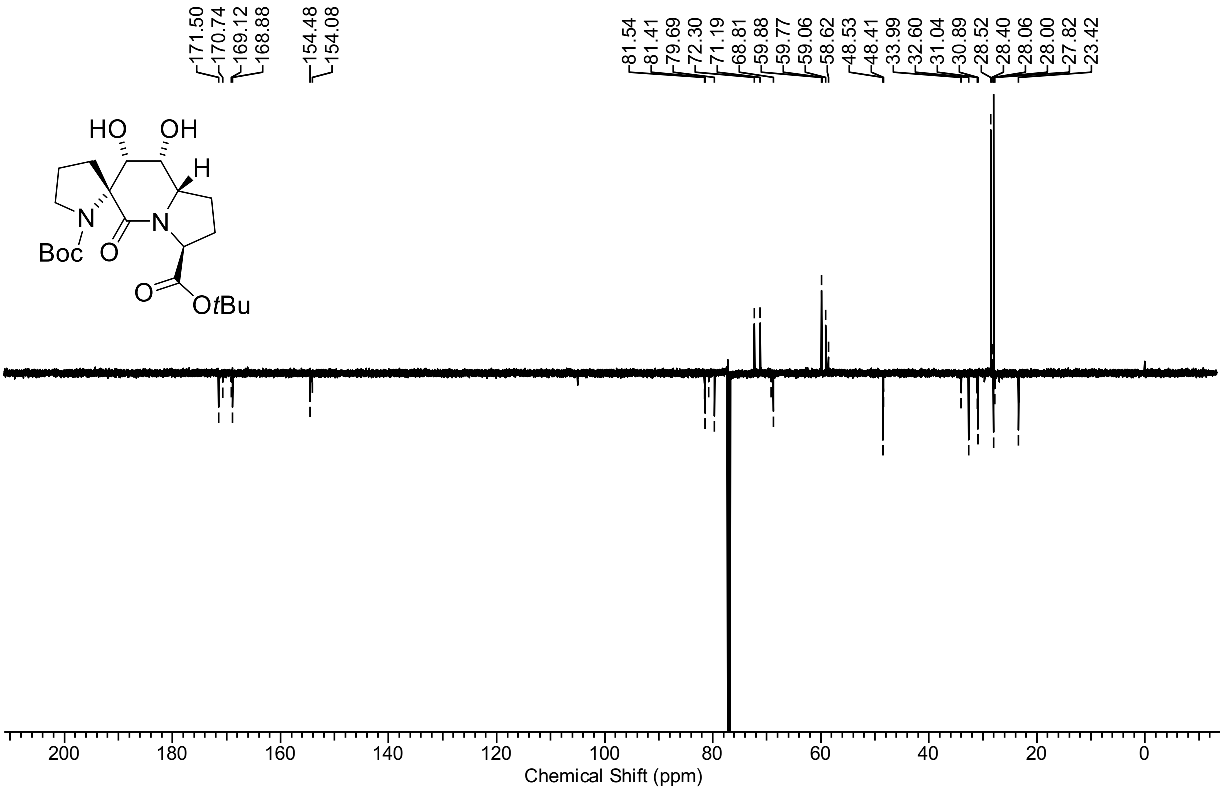

Figure S40. ^{13}C-APT-NMR spectrum of Boc-α-**(HO)$_2$-ProM2**-O*t*Bu in $CDCl_3$ at 125 MHz.

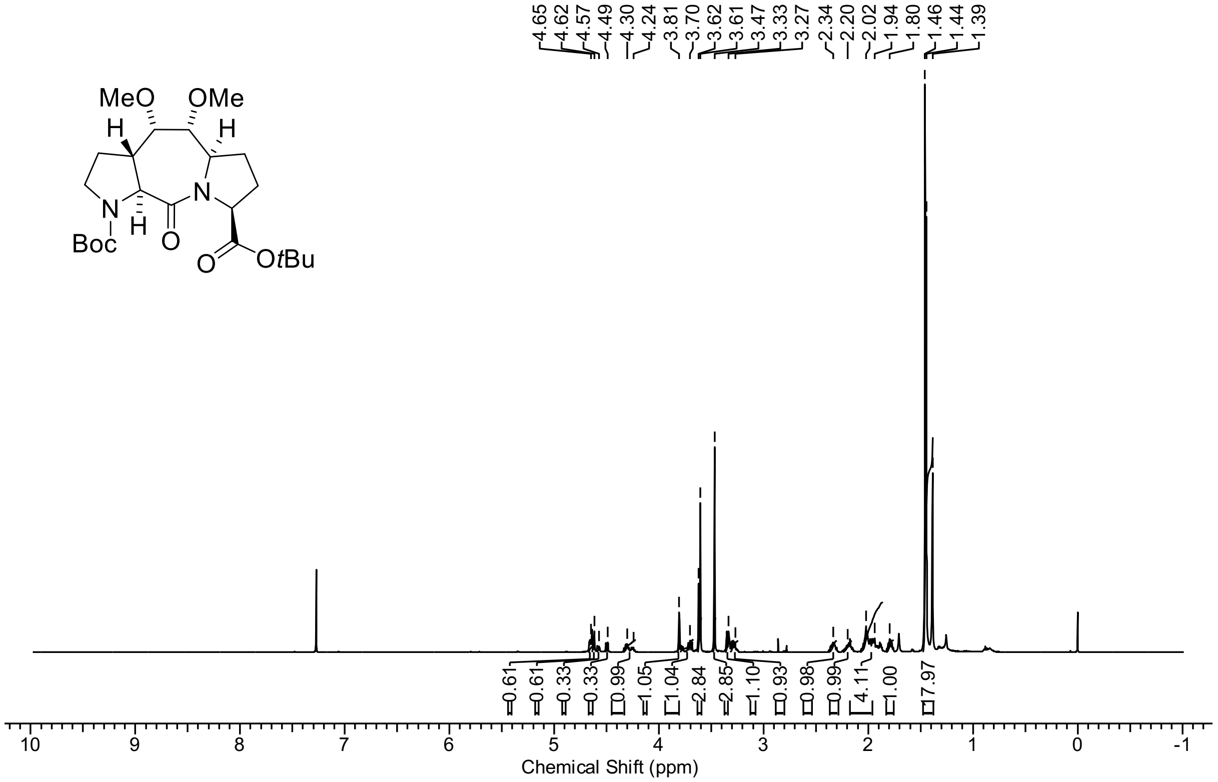

Figure S41. ^{1}H-NMR spectrum of Boc-**(MeO)$_2$-ProM1**-O*t*Bu in $CDCl_3$ at 500 MHz (full view).

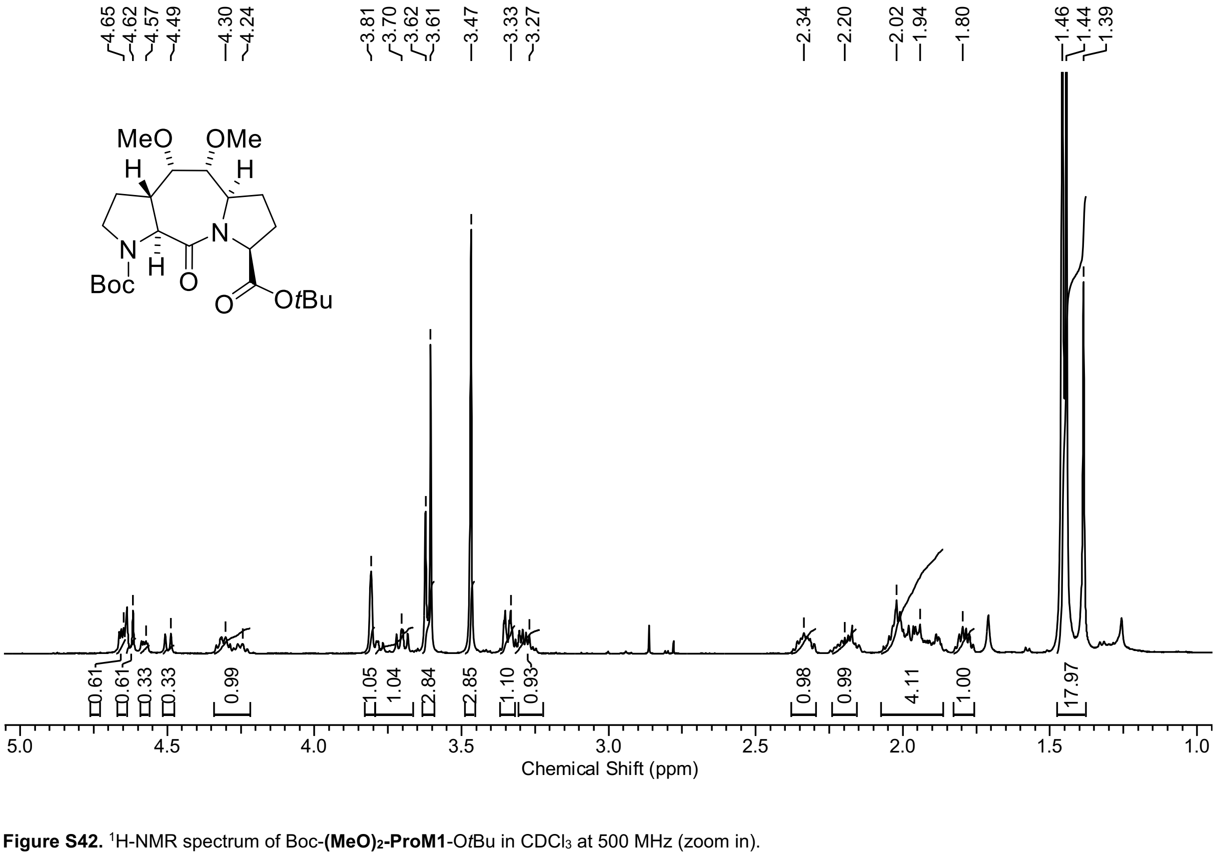

Figure S42. ^{1}H-NMR spectrum of Boc-**(MeO)$_2$-ProM1**-O*t*Bu in $CDCl_3$ at 500 MHz (zoom in).

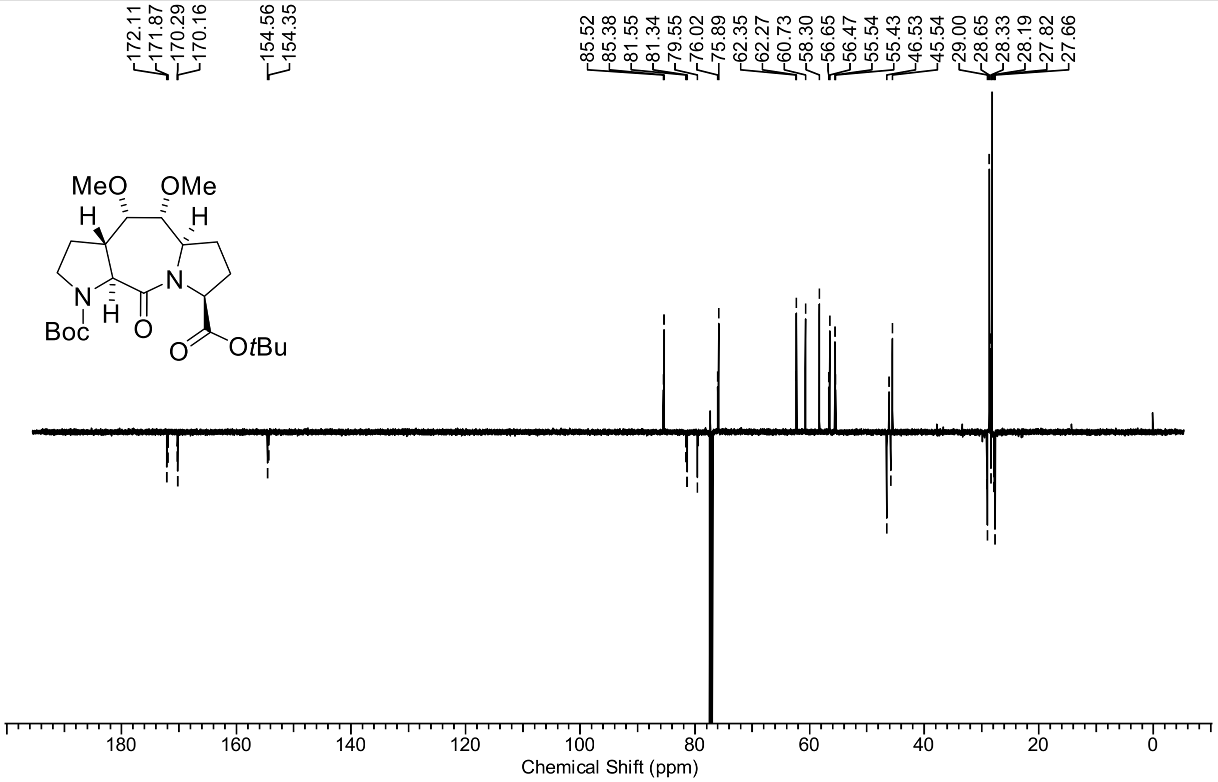

Figure S43. ^{13}C-APT-NMR spectrum of Boc-**(MeO)$_2$-ProM1**-O*t*Bu in $CDCl_3$ at 125 MHz.

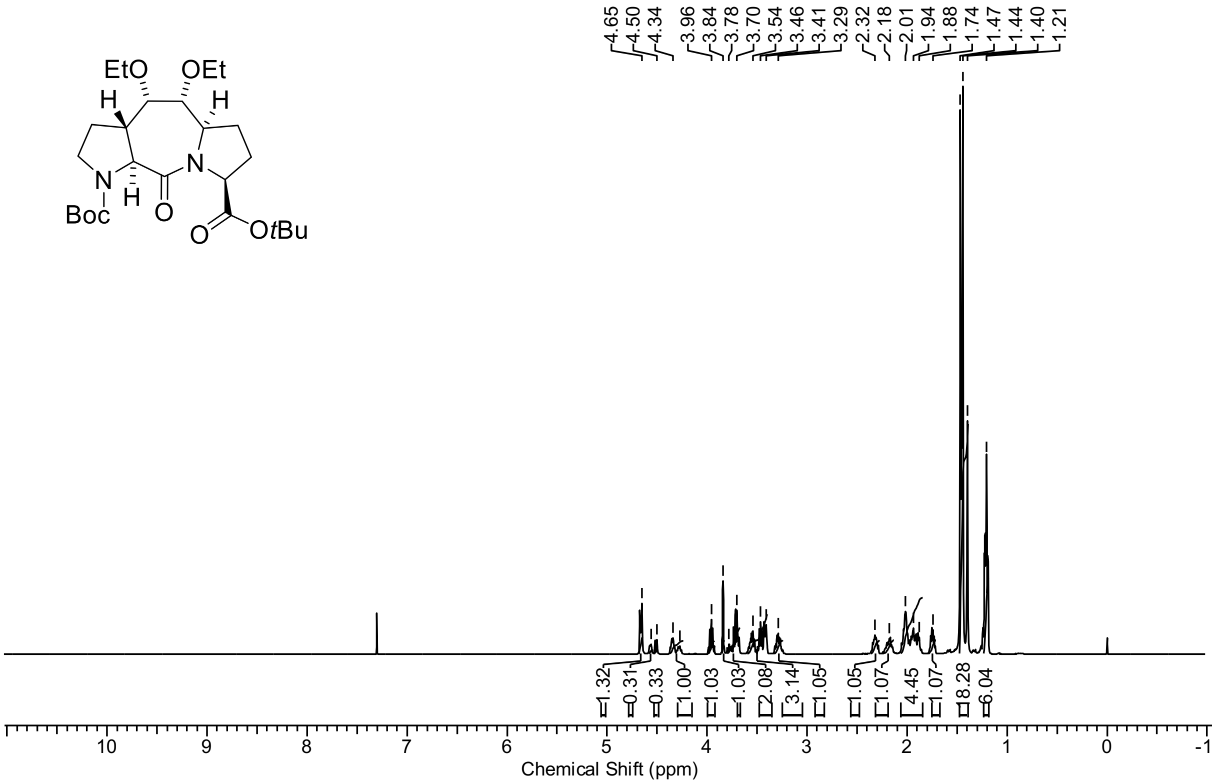

Figure S44. ^{1}H-NMR spectrum of Boc-**(EtO)$_2$-ProM1**-O*t*Bu in $CDCl_3$ at 500 MHz (full view).

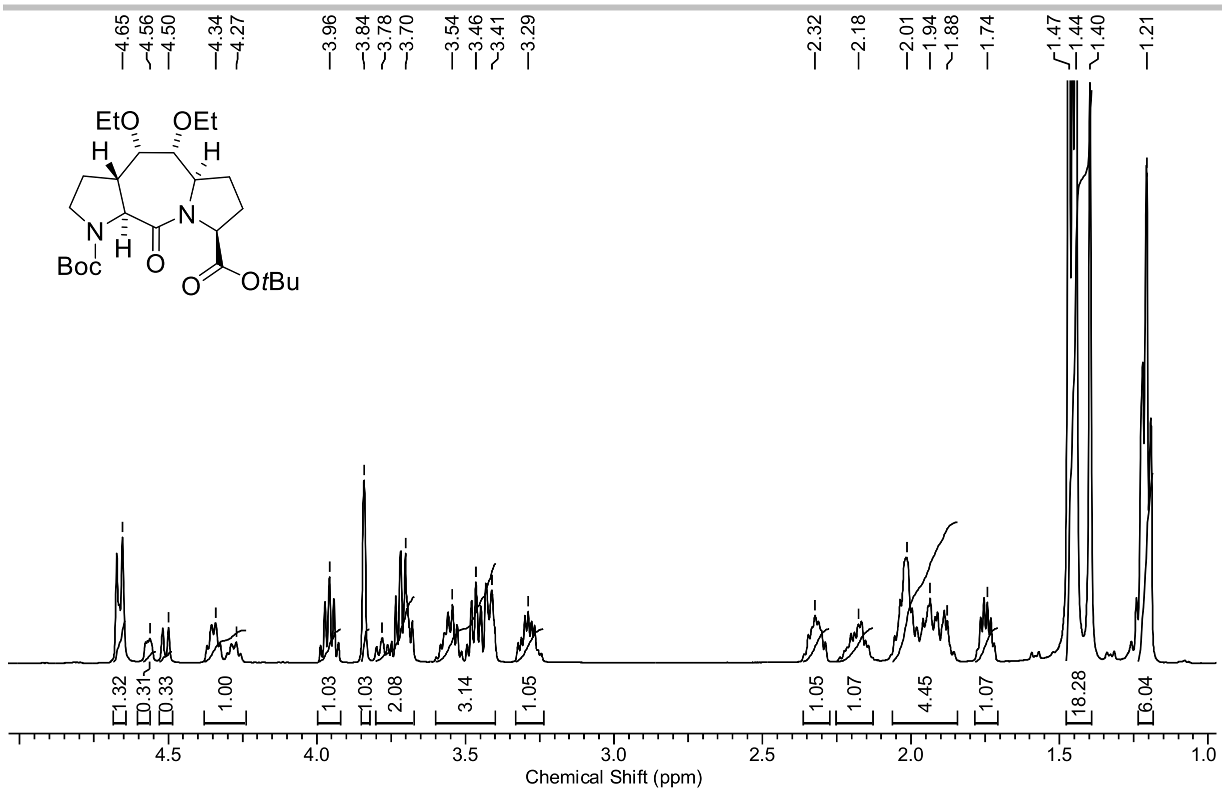

Figure S45. ^{1}H-NMR spectrum of Boc-**(EtO)$_2$-ProM1**-O*t*Bu in $CDCl_3$ at 500 MHz (zoom in).

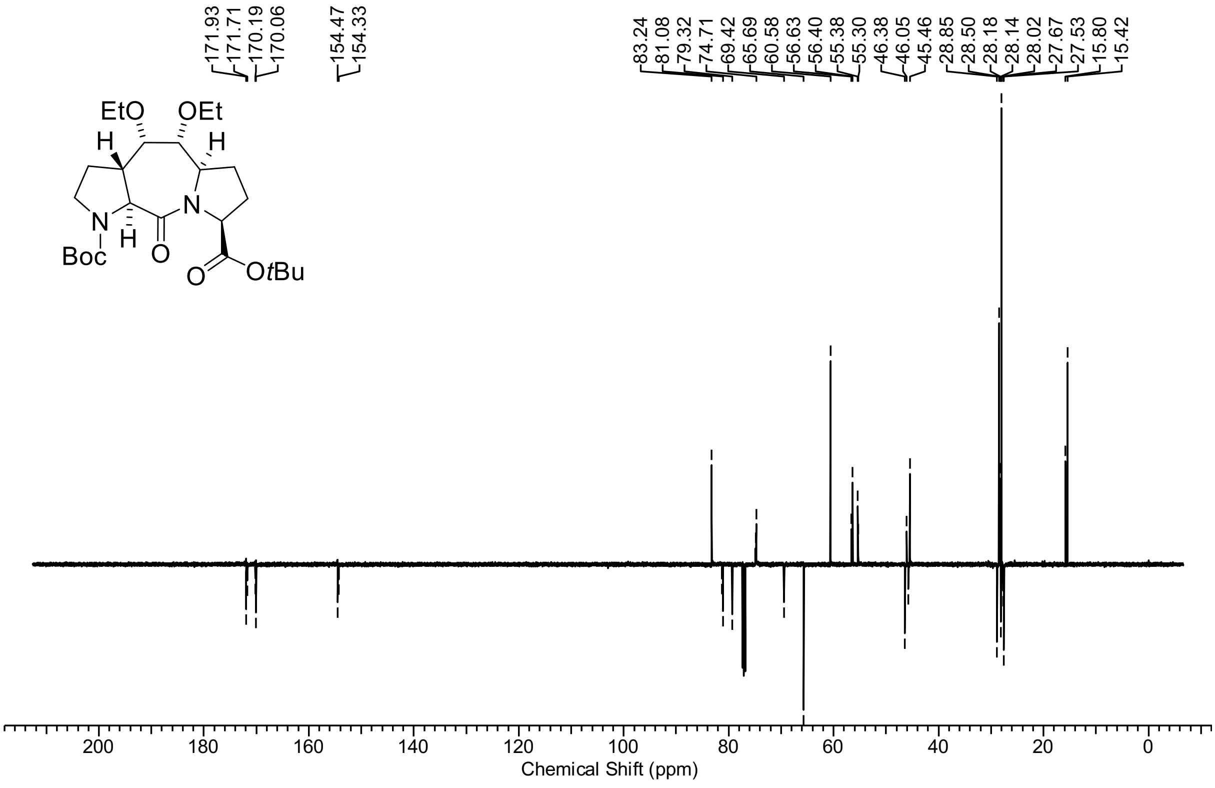

Figure S46. ^{13}C-APT-NMR spectrum of Boc-**(EtO)$_2$-ProM1**-O*t*Bu in $CDCl_3$ at 125 MHz.

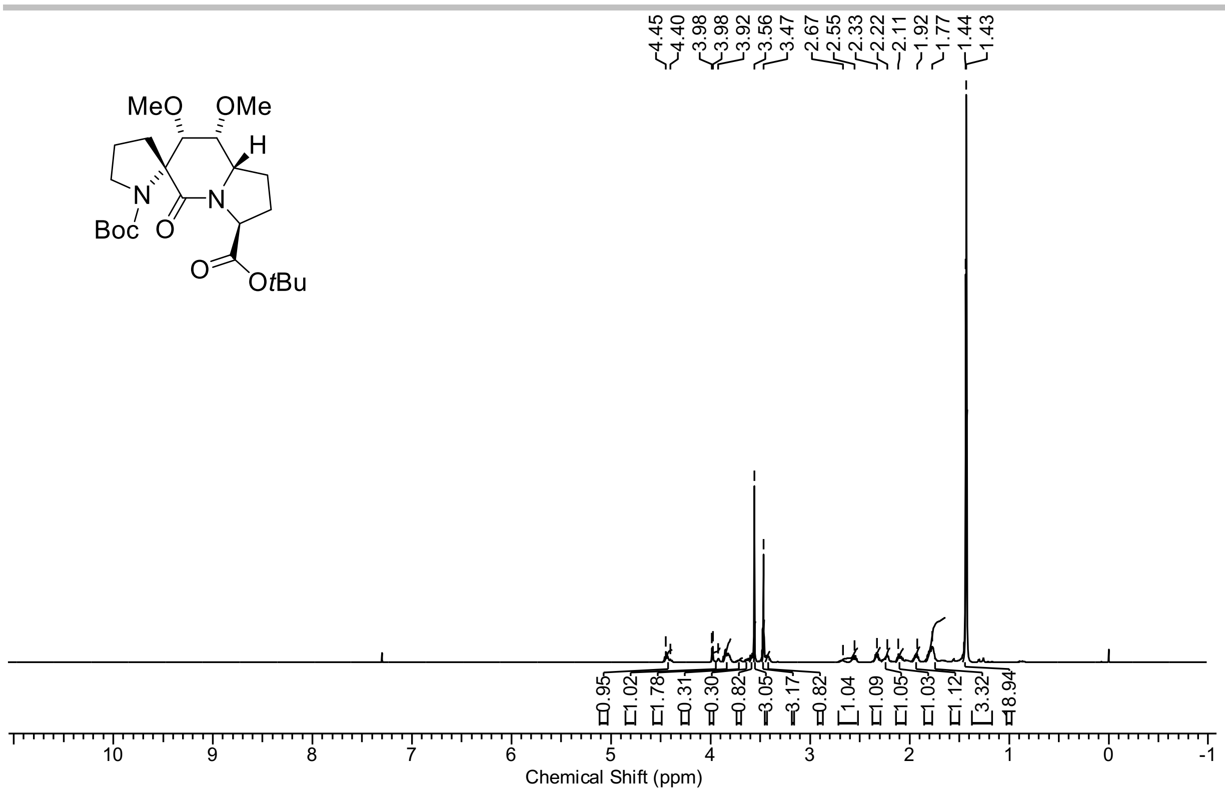

Figure S47. ^{1}H-NMR spectrum of Boc-**(MeO)$_2$-ProM2**-O*t*Bu in $CDCl_3$ at 500 MHz (full view).

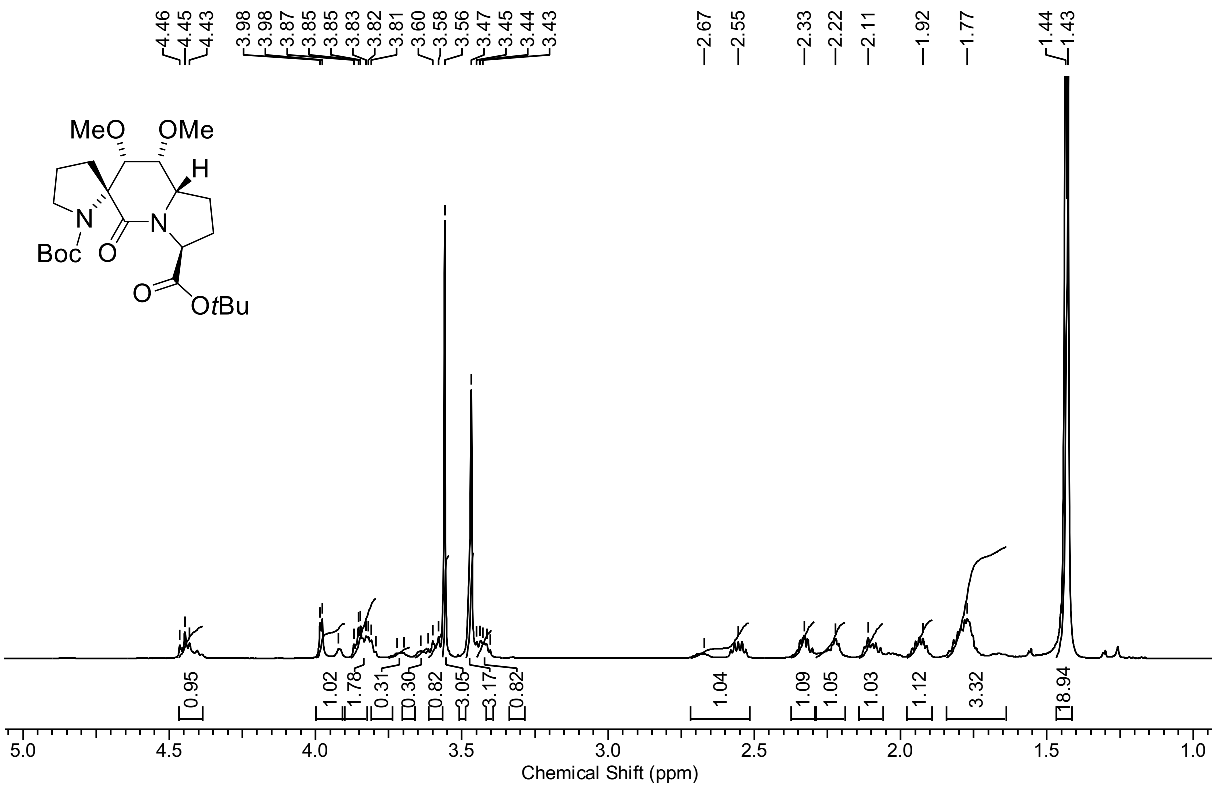

Figure S48. ^{1}H-NMR spectrum of Boc-**(MeO)$_2$-ProM2**-O*t*Bu in $CDCl_3$ at 500 MHz (zoom in).

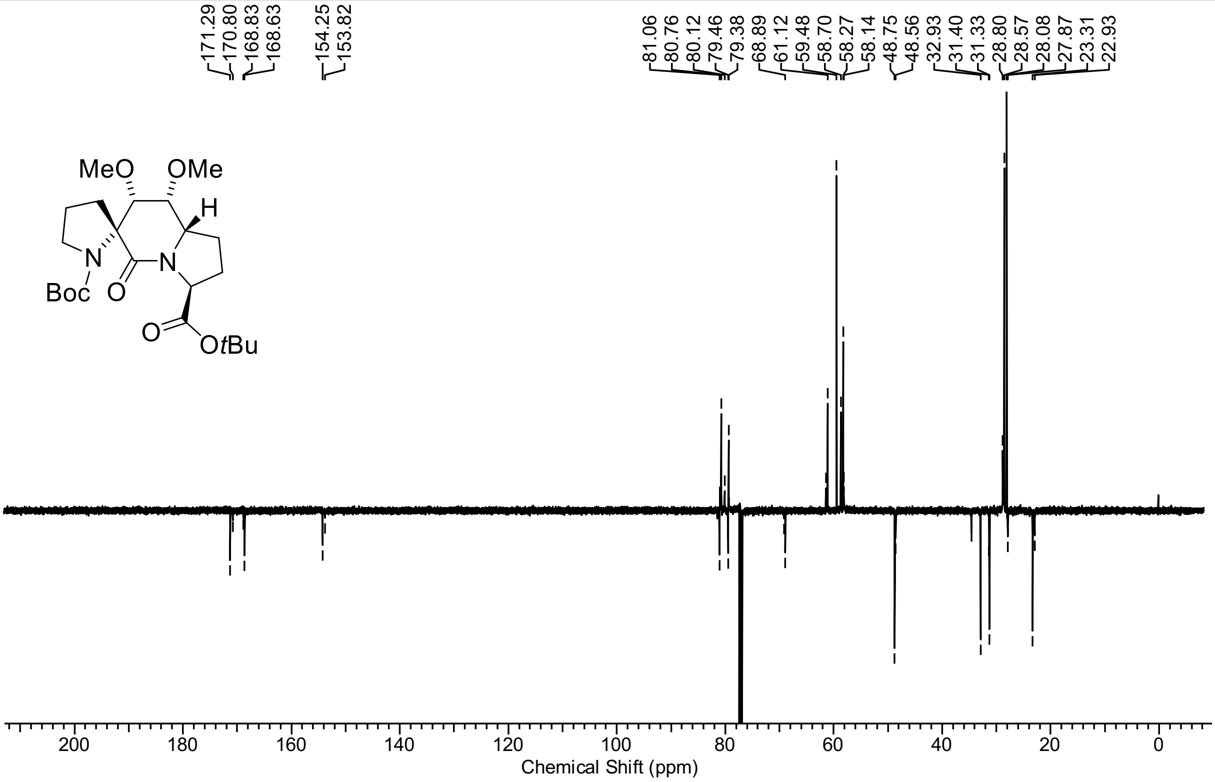

Figure S49. ^{13}C-APT-NMR spectrum of Boc-**(MeO)$_2$-ProM2**-O*t*Bu in $CDCl_3$ at 125 MHz.

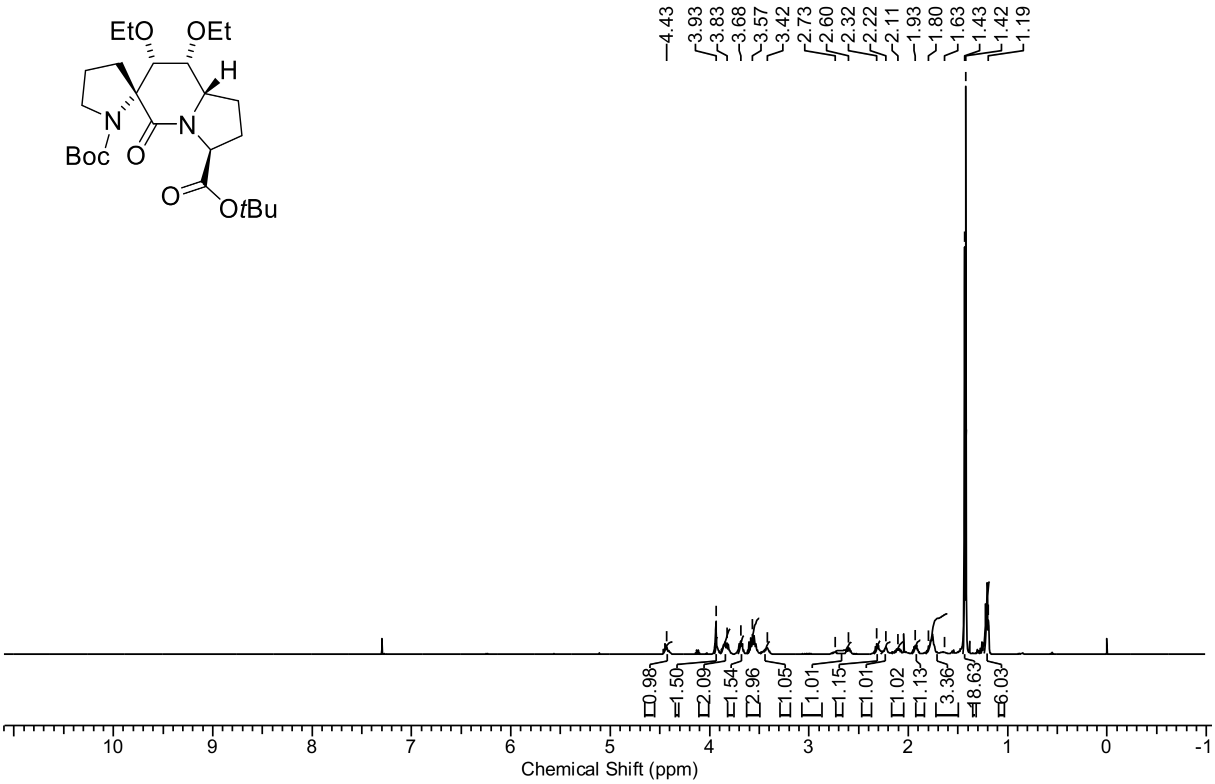

Figure S50. ^{1}H-NMR spectrum of Boc-**(EtO)$_2$-ProM2**-O*t*Bu in $CDCl_3$ at 500 MHz (full view).

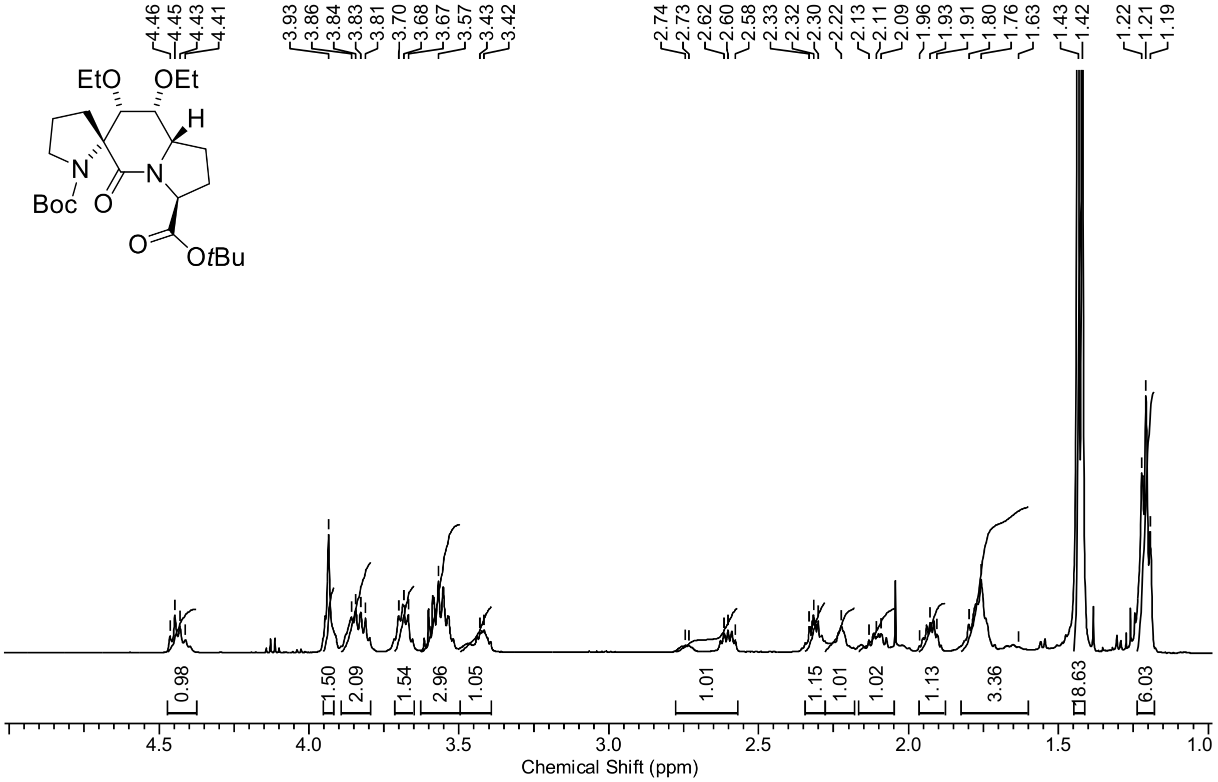

Figure S51. ^{1}H-NMR spectrum of Boc-**(EtO)$_2$-ProM2**-O*t*Bu in $CDCl_3$ at 500 MHz (zoom in).

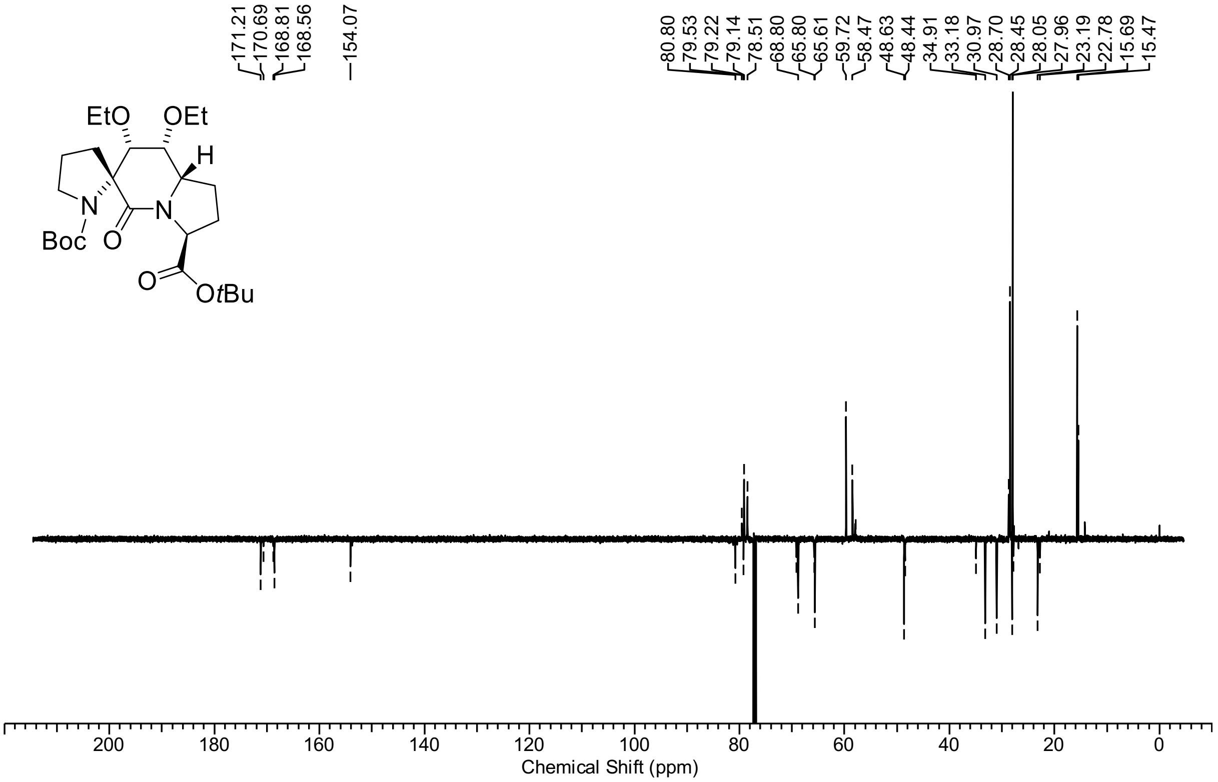

Figure S52. ^{13}C-APT-NMR spectrum of Boc-**(EtO)$_2$-ProM2**-O*t*Bu in $CDCl_3$ at 125 MHz.

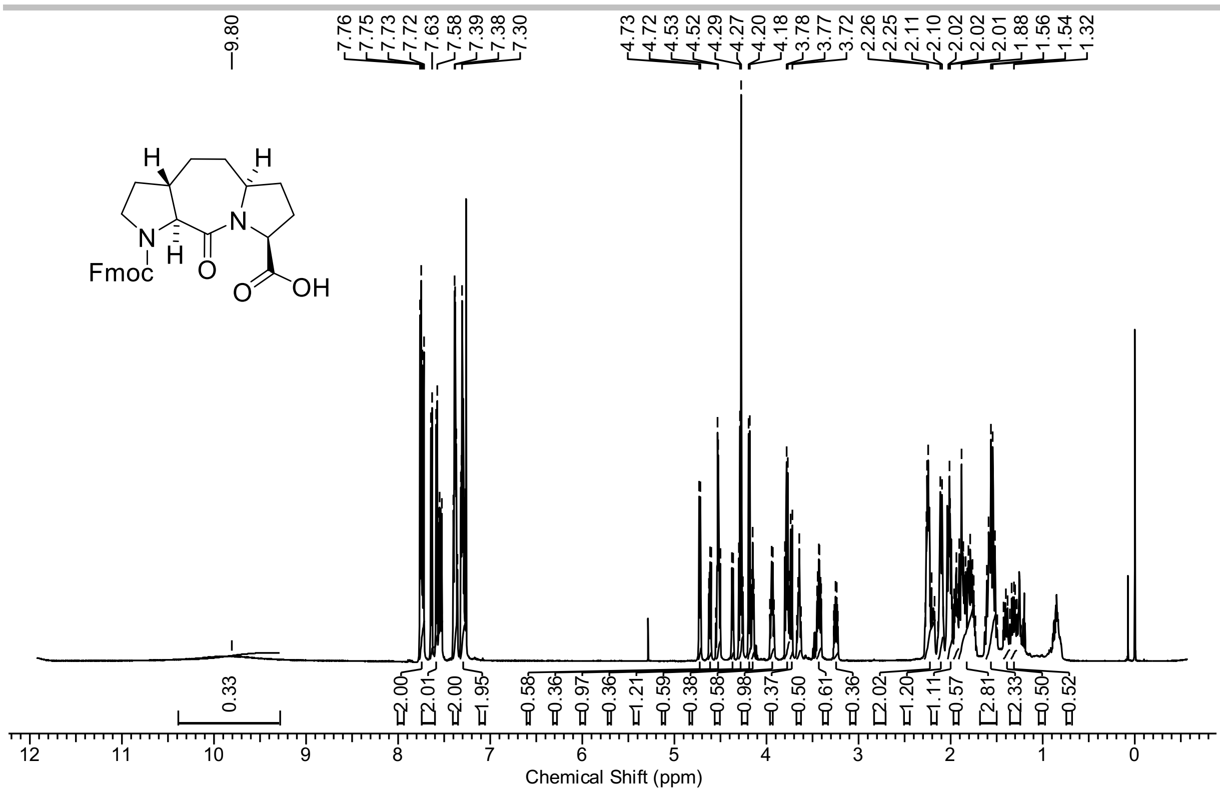

Figure S53. ^{1}H-NMR spectrum of Fmoc-**H_2-ProM1**-OH in $CDCl_3$ at 600 MHz (full view).

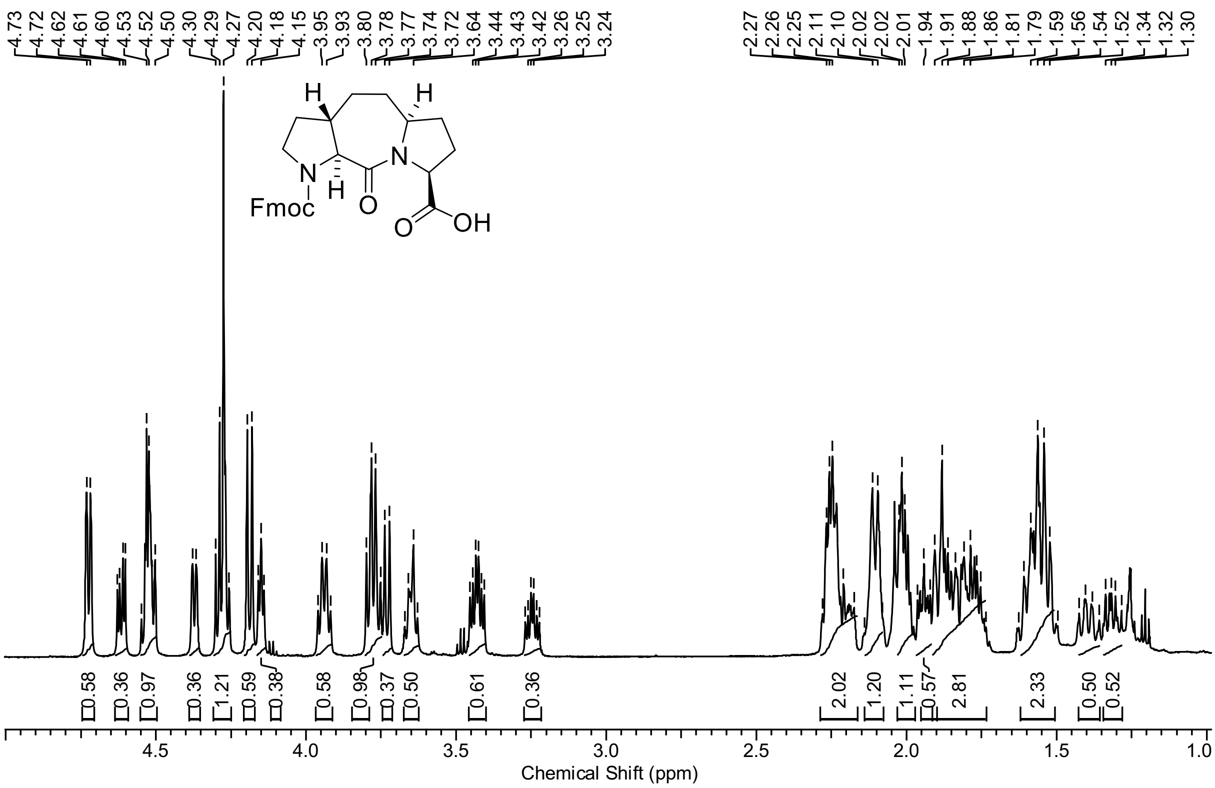

Figure S54. ^{1}H-NMR spectrum of Fmoc-**H_2-ProM1**-OH in $CDCl_3$ at 600 MHz (zoom in).

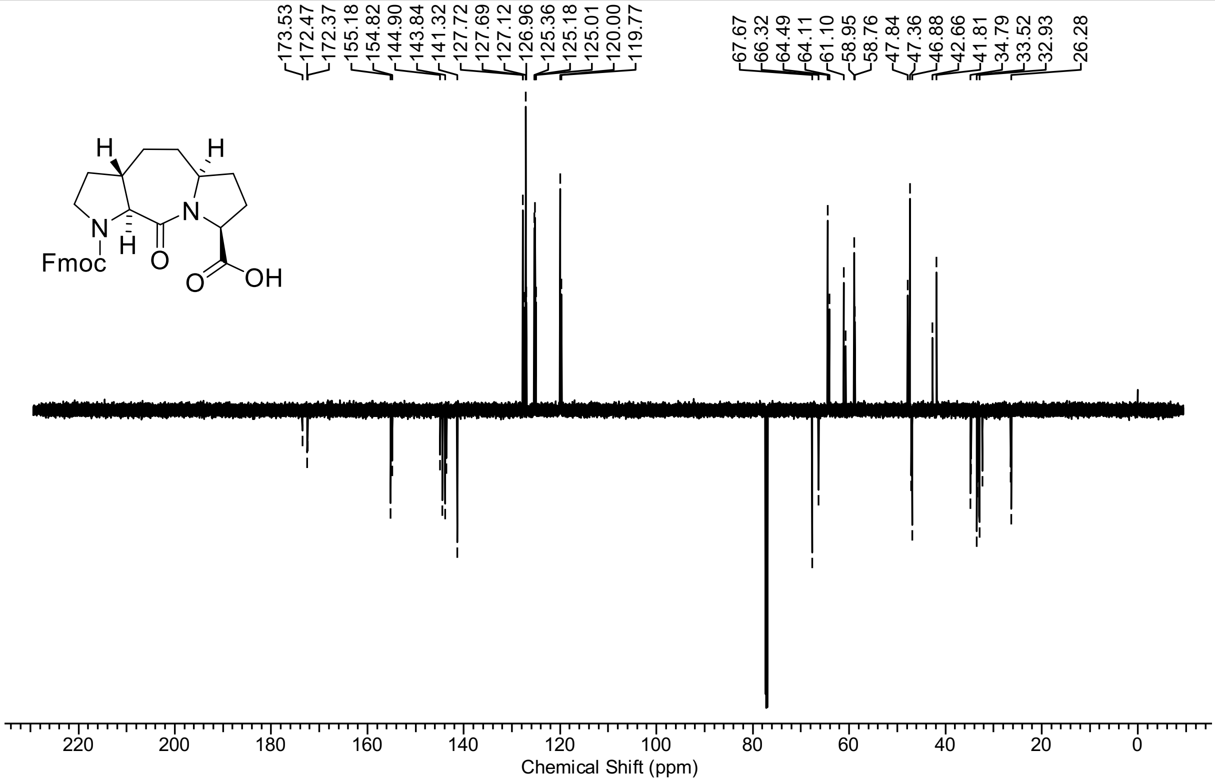

Figure S55. ^{13}C-APT-NMR spectrum of Fmoc-**H$_2$-ProM1**-OH in $CDCl_3$ at 150 MHz.

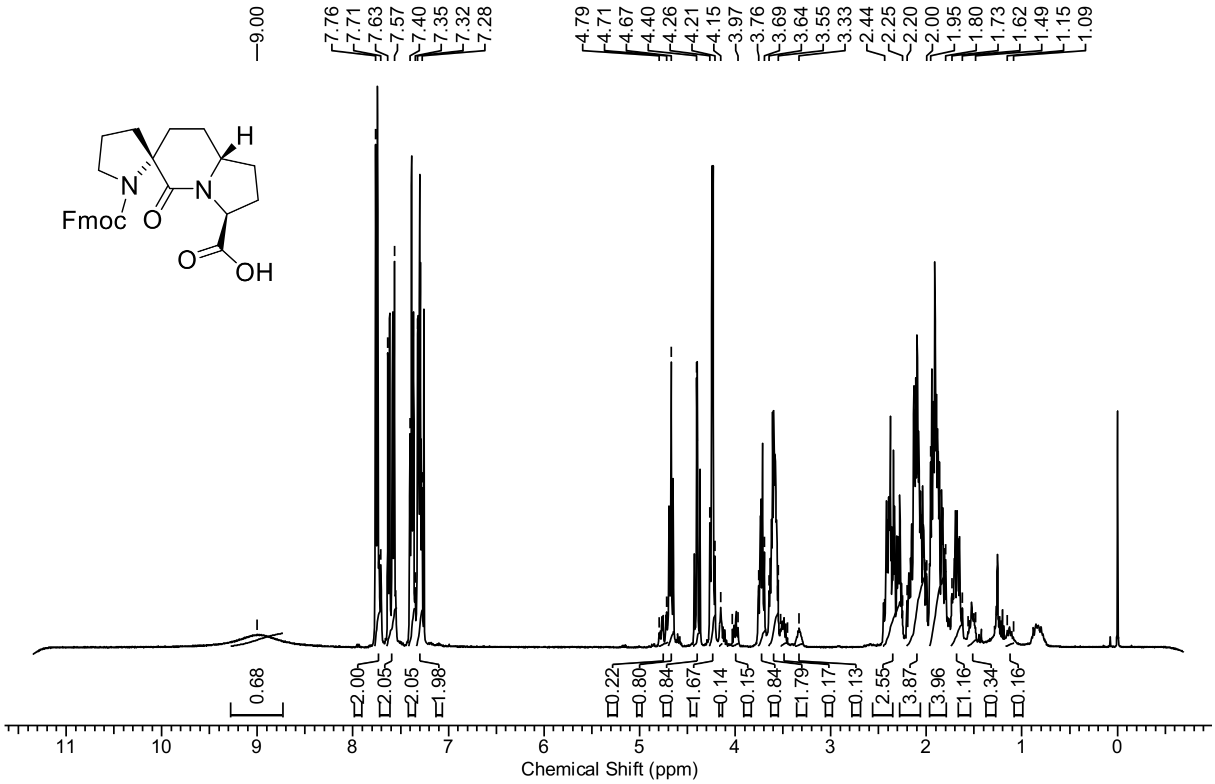

Figure S56. ^{1}H-NMR spectrum of Fmoc-**H$_2$-ProM2**-OH in $CDCl_3$ at 400 MHz (full view).

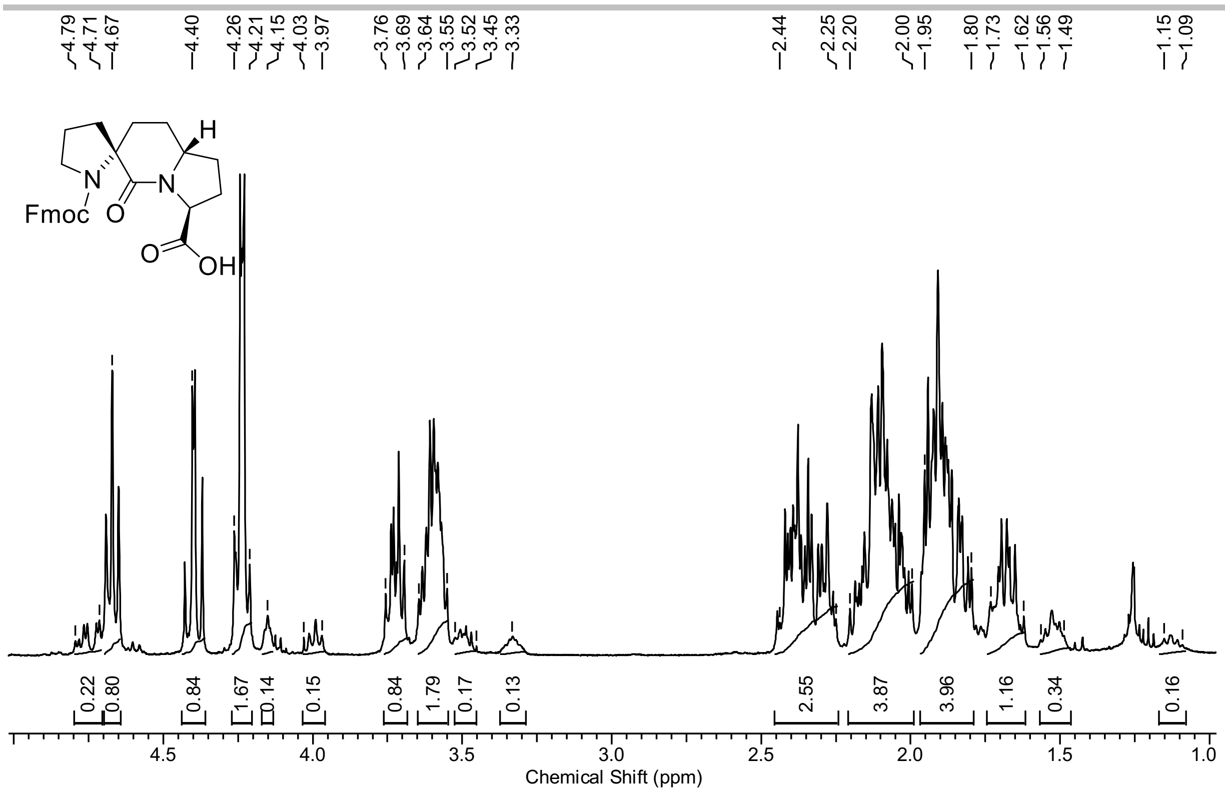

Figure S57. ^{1}H-NMR spectrum of Fmoc-**H$_2$-ProM2**-OH in $CDCl_3$ at 400 MHz (zoom in).

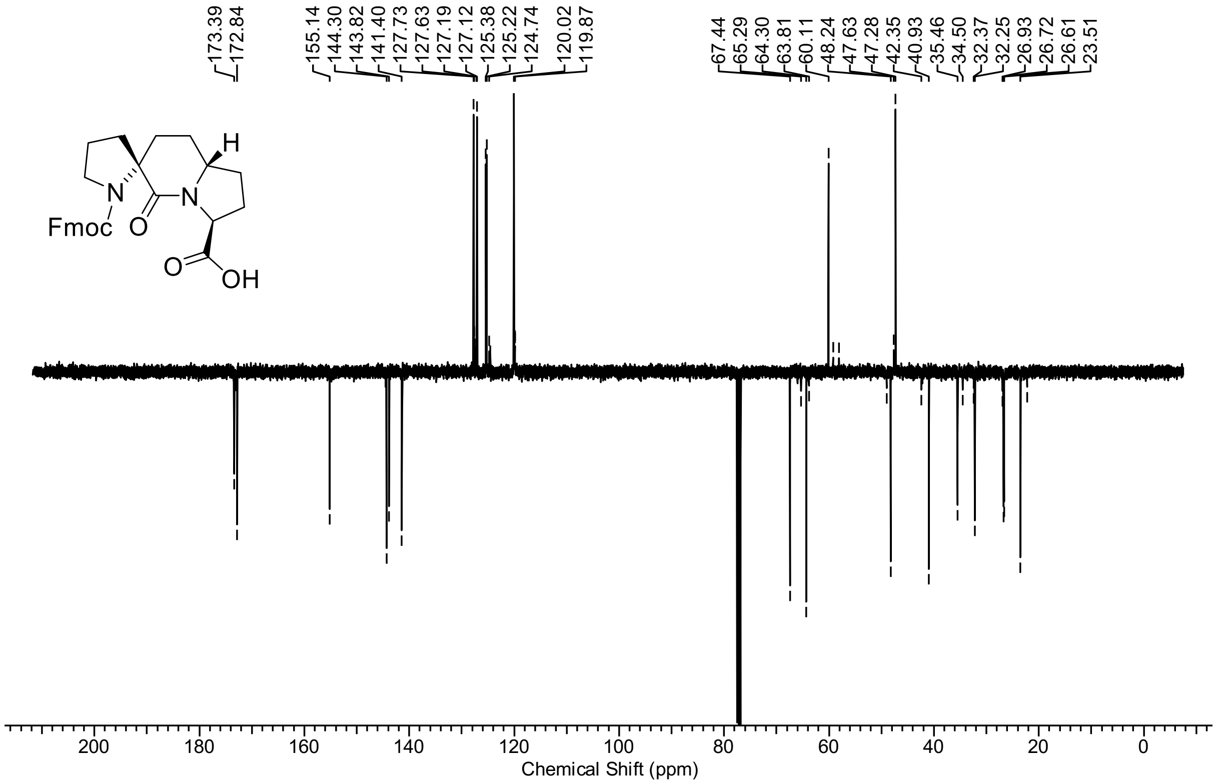

Figure S58. ^{13}C-APT-NMR spectrum of Fmoc-**H$_2$-ProM2**-OH in $CDCl_3$ at 100 MHz.

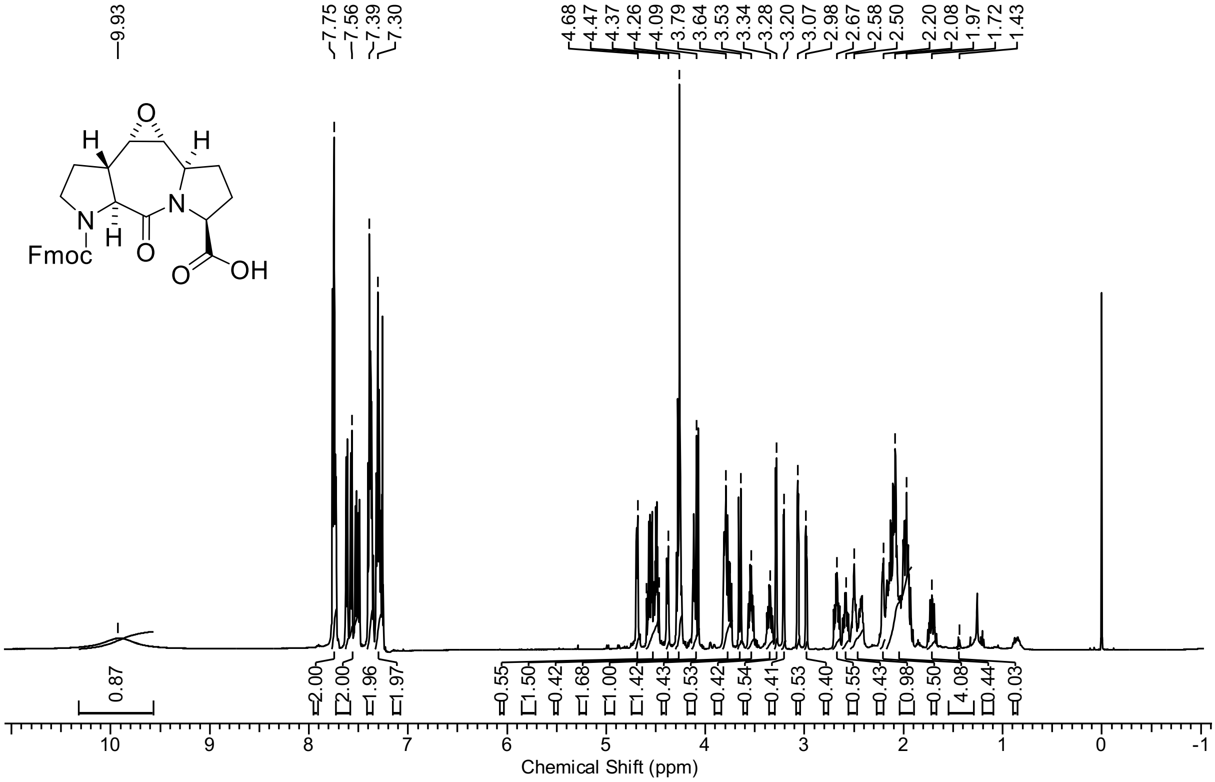

Figure S59. ^{1}H-NMR spectrum of Fmoc-**ep-ProM1**-OH in $CDCl_3$ at 500 MHz (full view).

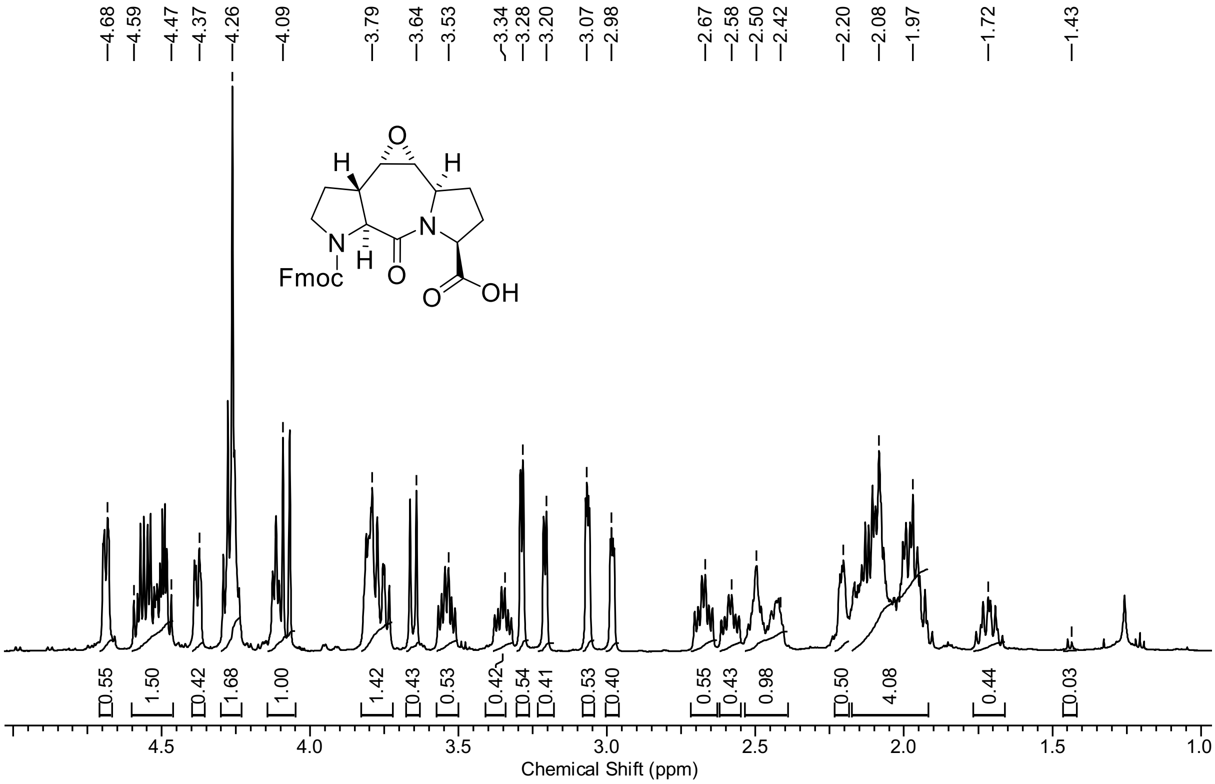

Figure S60. ^{1}H-NMR spectrum of Fmoc-**ep-ProM1**-OH in $CDCl_3$ at 500 MHz (zoom in).

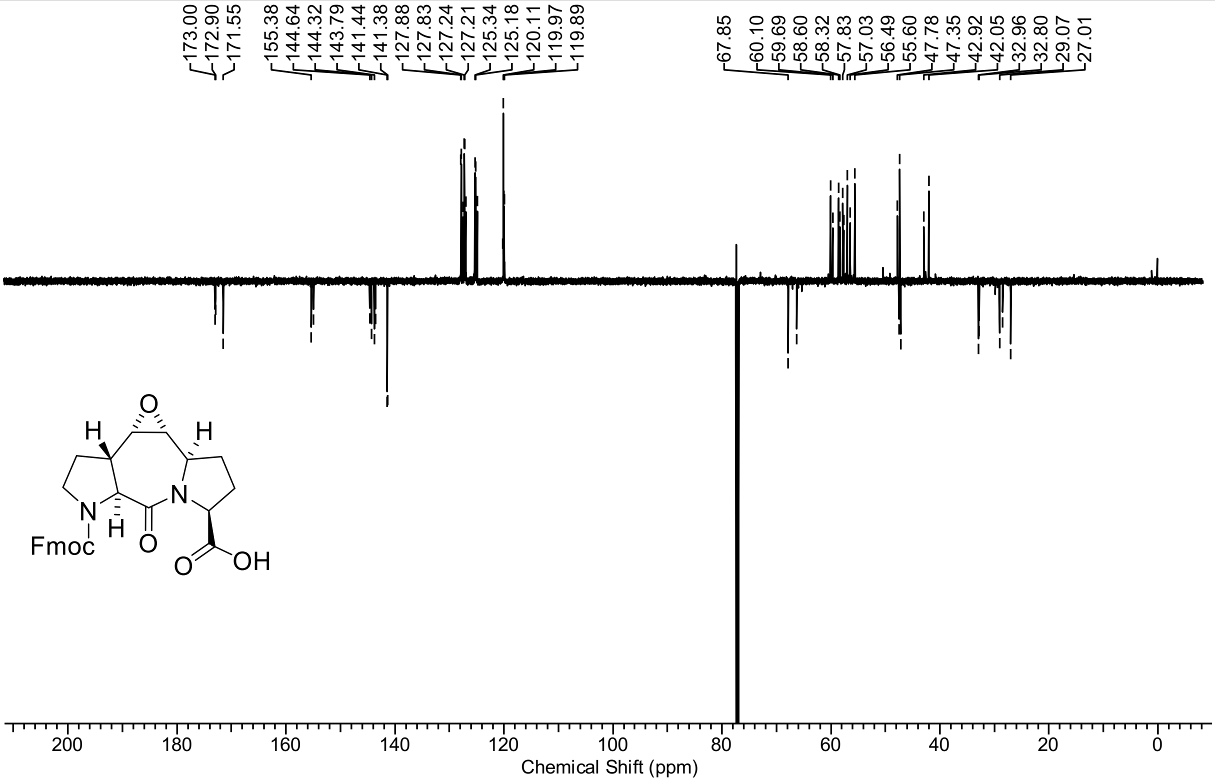

Figure S61. ^{13}C-APT-NMR spectrum of Fmoc-**ep-ProM1**-OH in $CDCl_3$ at 125 MHz.

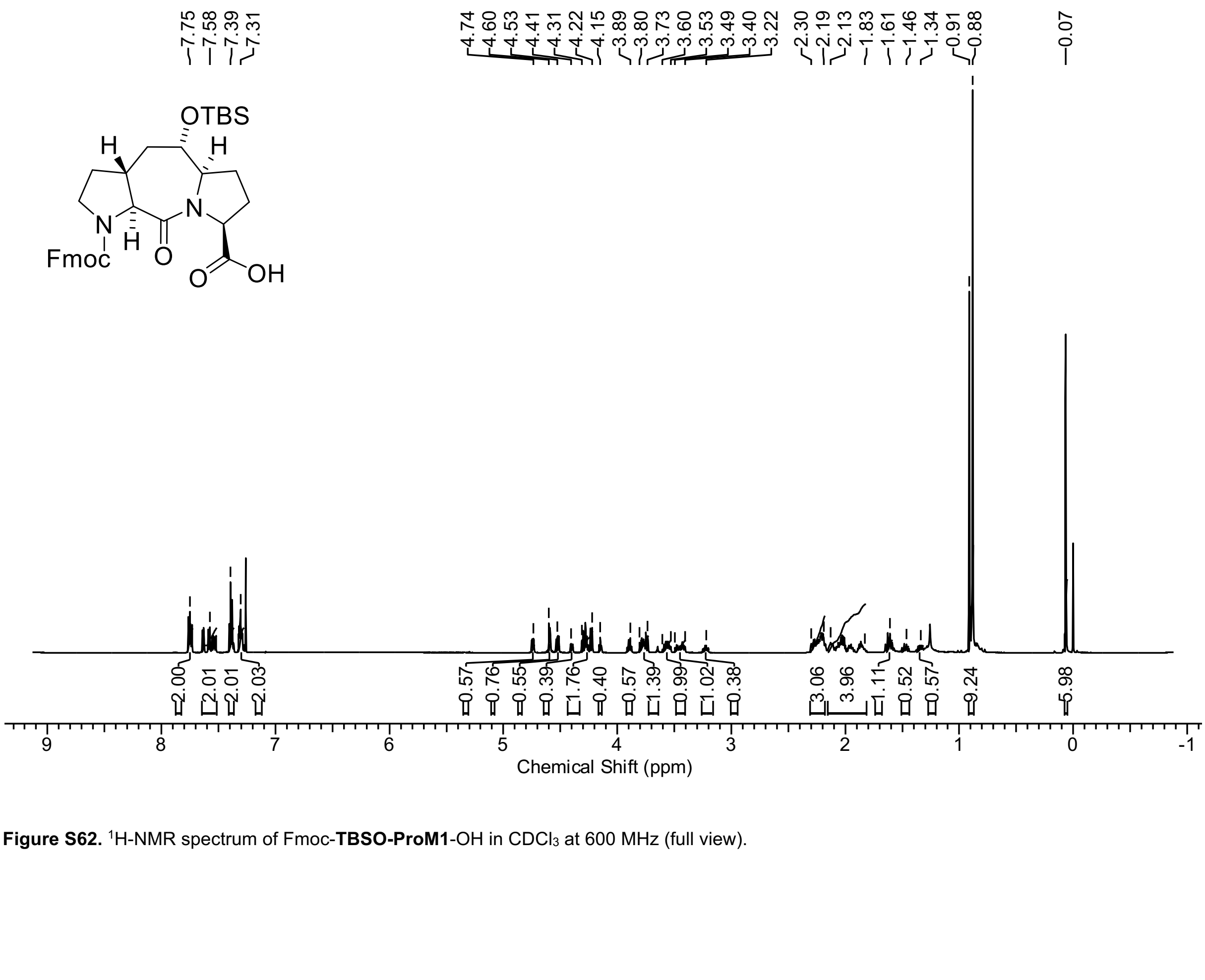

Figure S62. ^{1}H-NMR spectrum of Fmoc-**TBSO-ProM1**-OH in $CDCl_3$ at 600 MHz (full view).

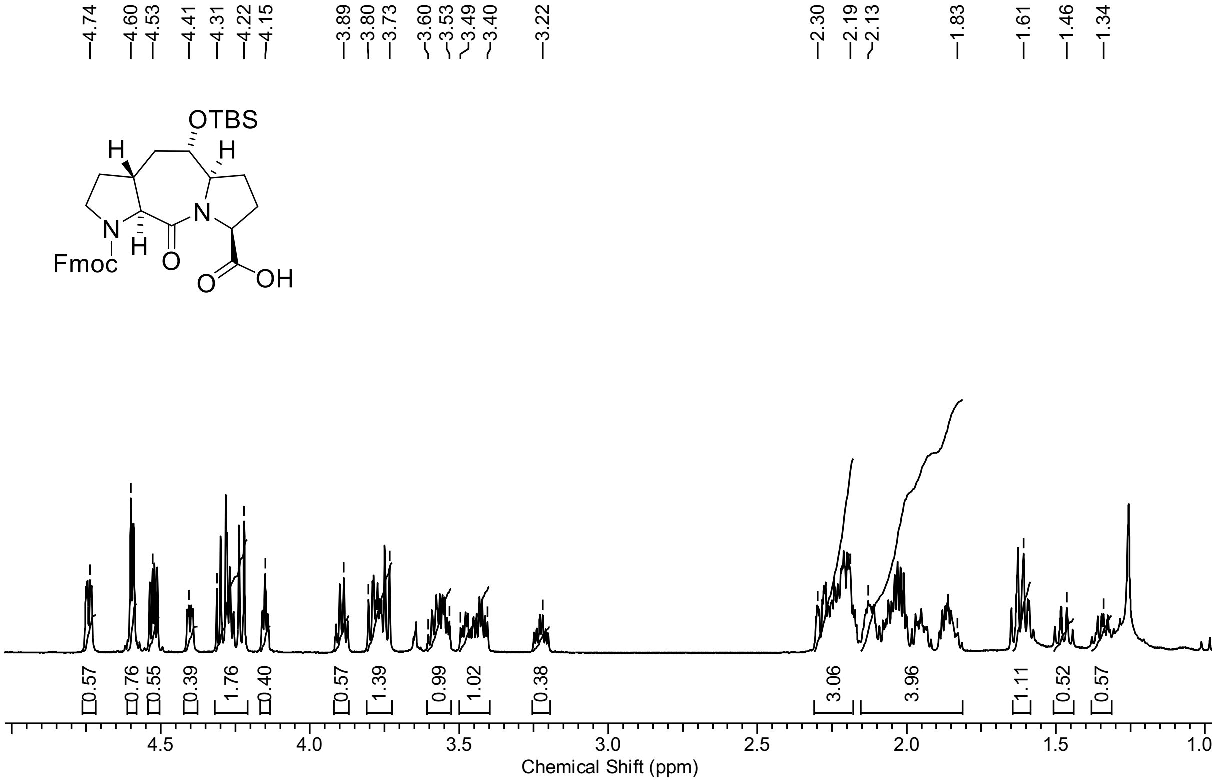

Figure S63. ^{1}H-NMR spectrum of Fmoc-**TBSO-ProM1**-OH in $CDCl_3$ at 600 MHz (zoom in).

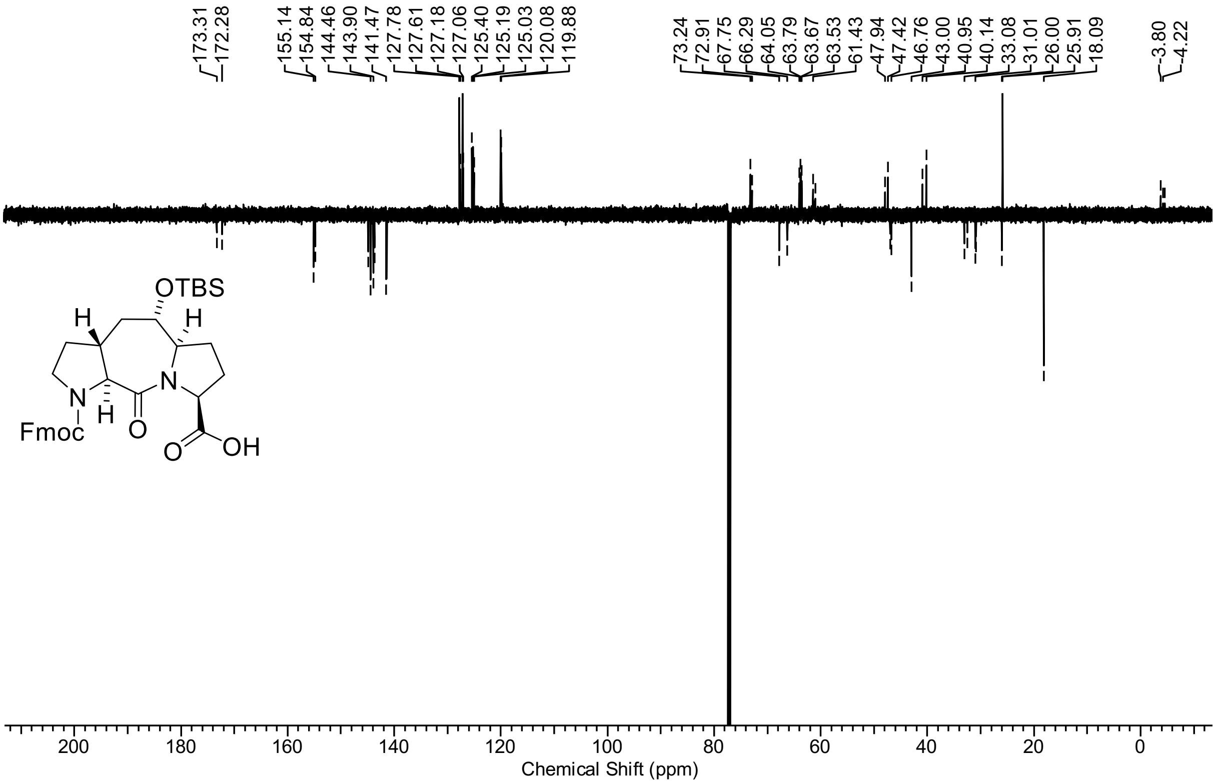

Figure S64. ^{13}C-APT-NMR spectrum of Fmoc-**TBSO-ProM1**-OH in $CDCl_3$ at 150 MHz.

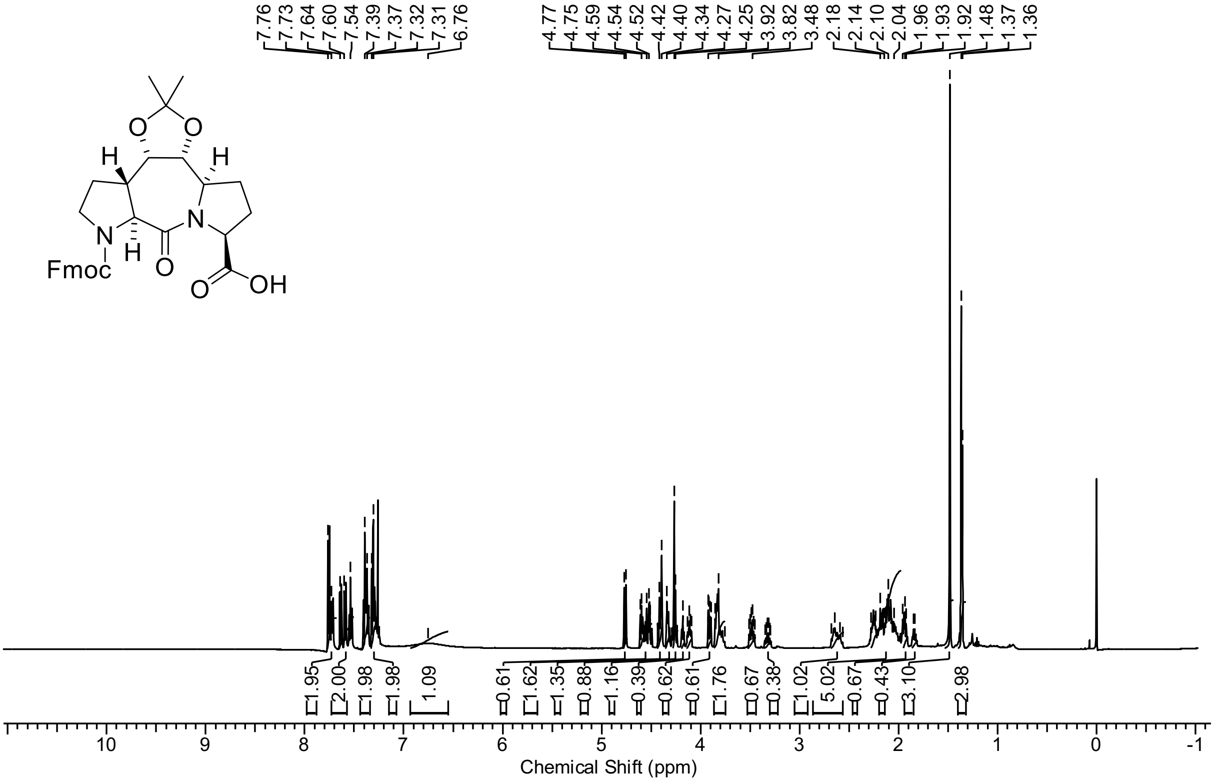

Figure S65. ^{1}H-NMR spectrum of Fmoc-**(Me_2CO_2)-ProM1**-OH in $CDCl_3$ at 500 MHz (full view).

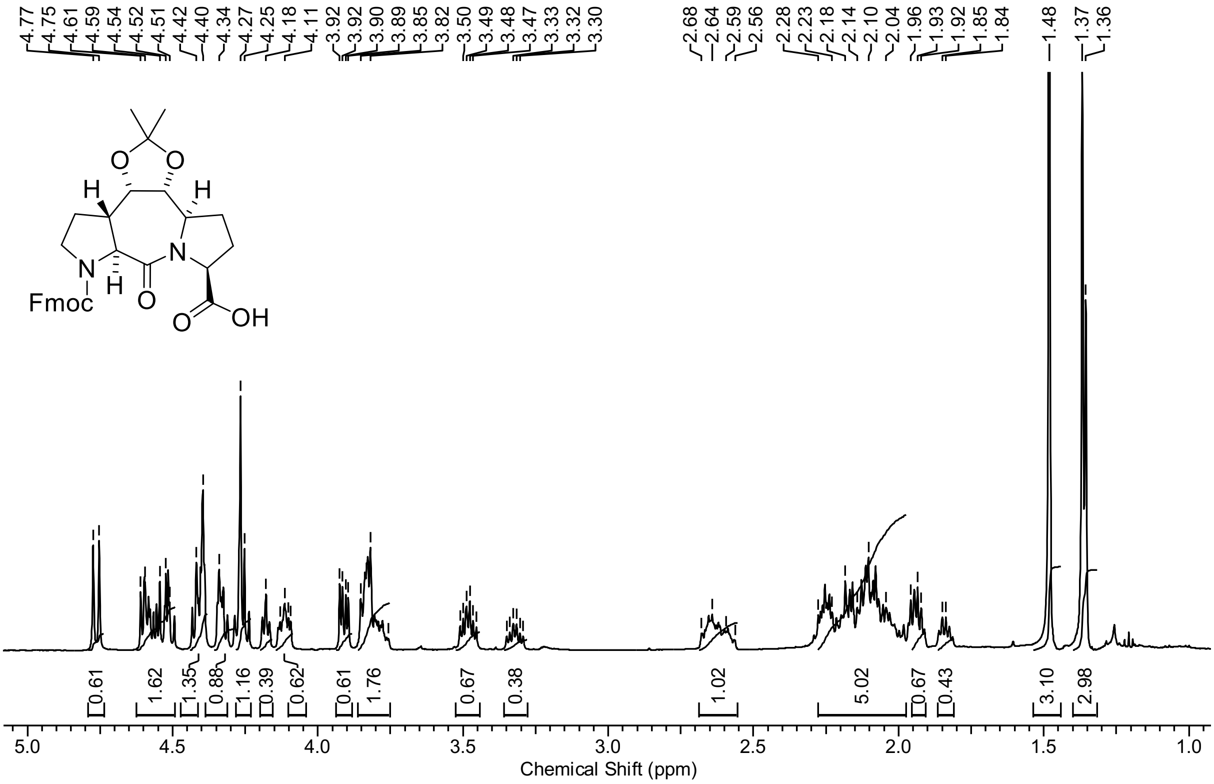

Figure S66. ^{1}H-NMR spectrum of Fmoc-**(Me_2CO_2)-ProM1**-OH in $CDCl_3$ at 500 MHz (zoom in).

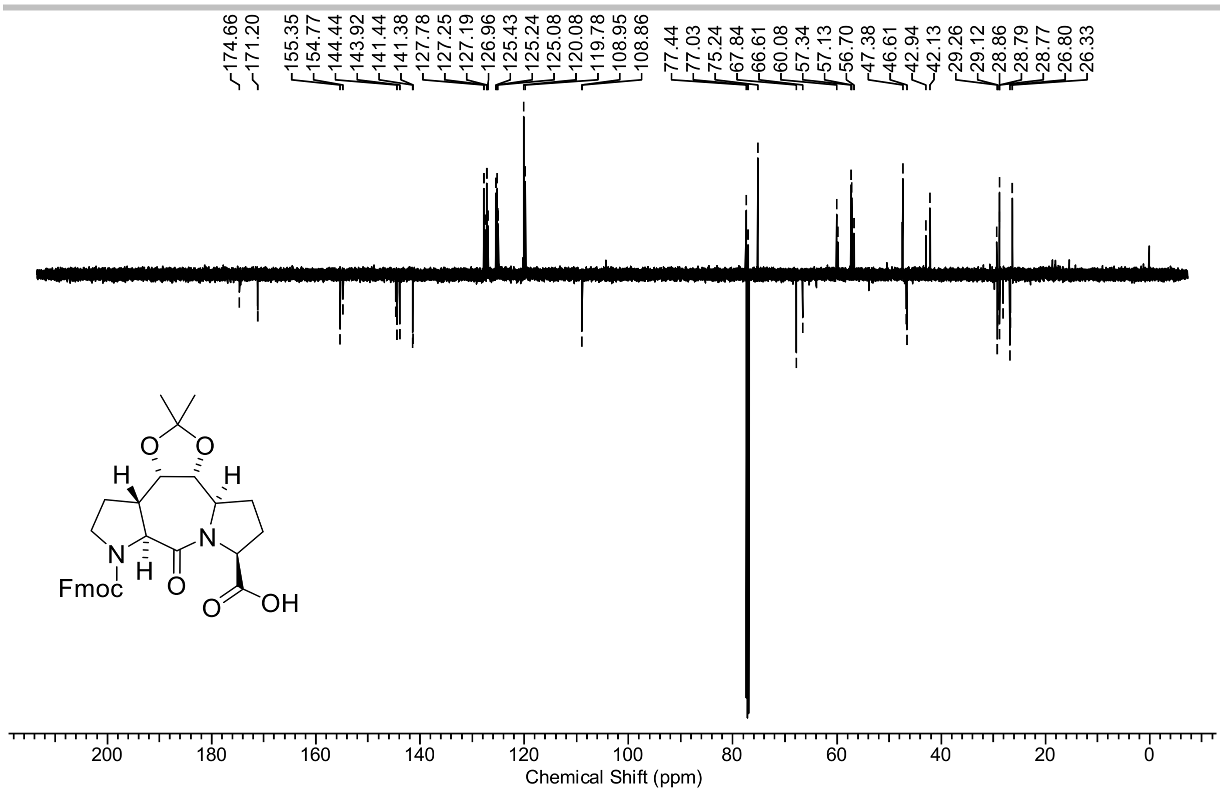

Figure S67. ^{13}C-APT-NMR spectrum of Fmoc-**(Me$_2$CO$_2$)-ProM1**-OH in $CDCl_3$ at 150 MHz.

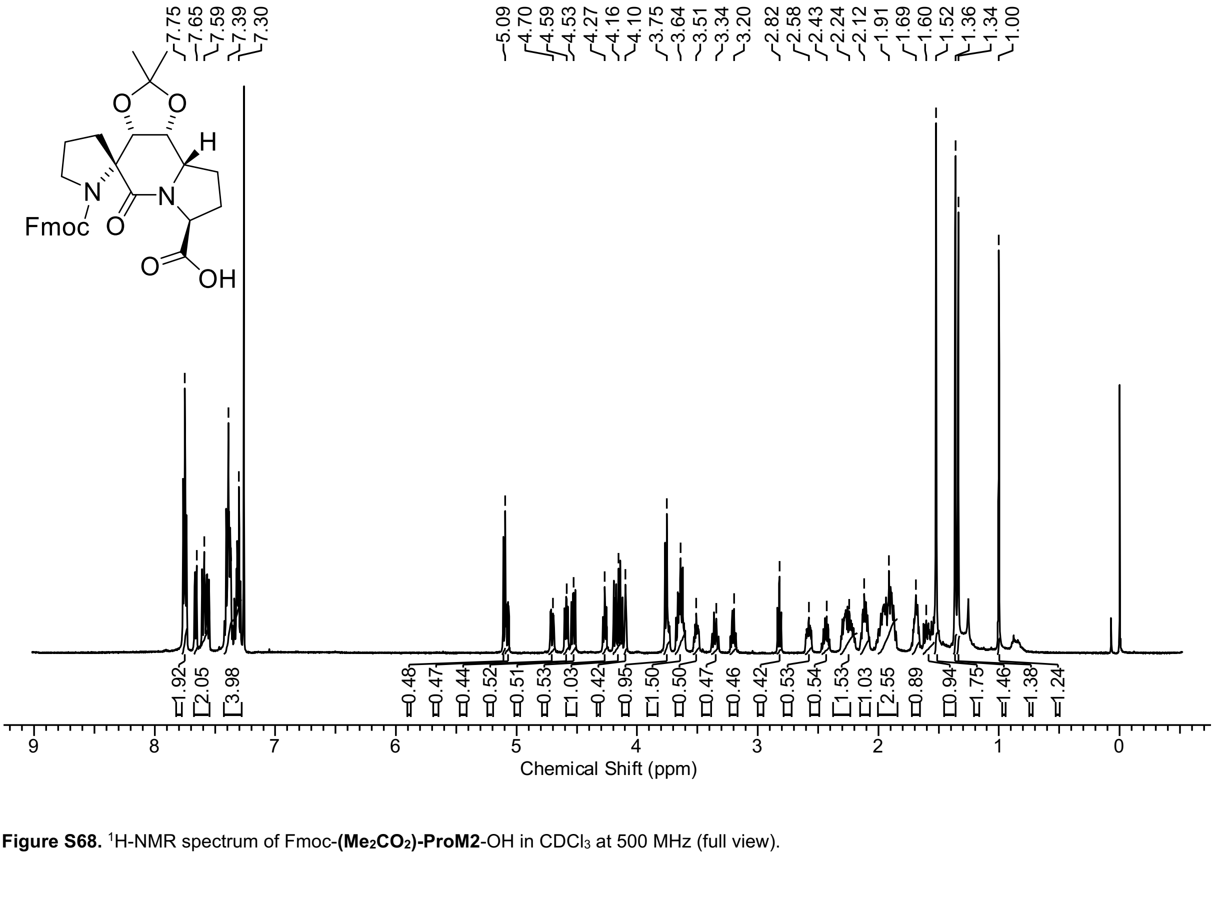

Figure S68. ^{1}H-NMR spectrum of Fmoc-**(Me$_2$CO$_2$)-ProM2**-OH in $CDCl_3$ at 500 MHz (full view).

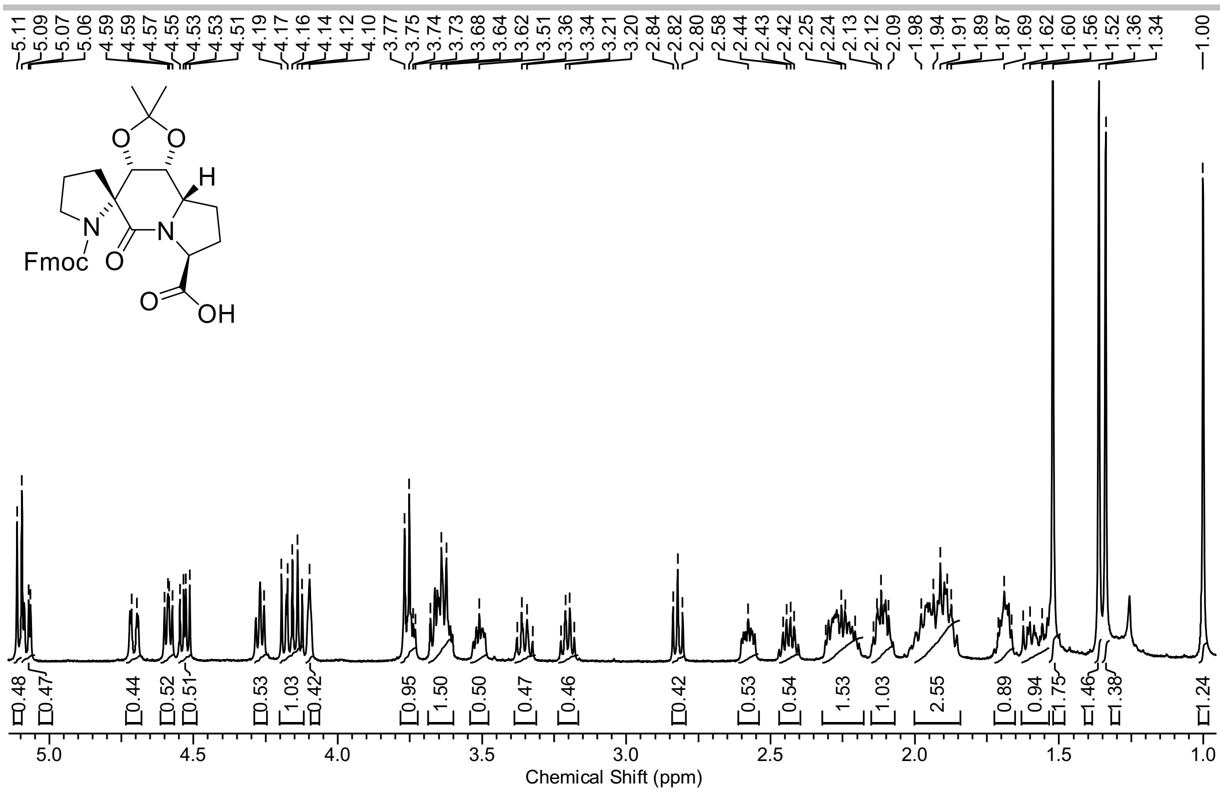

Figure S69. ^{1}H-NMR spectrum of Fmoc-**(Me_2CO_2)-ProM2**-OH in $CDCl_3$ at 500 MHz (zoom in).

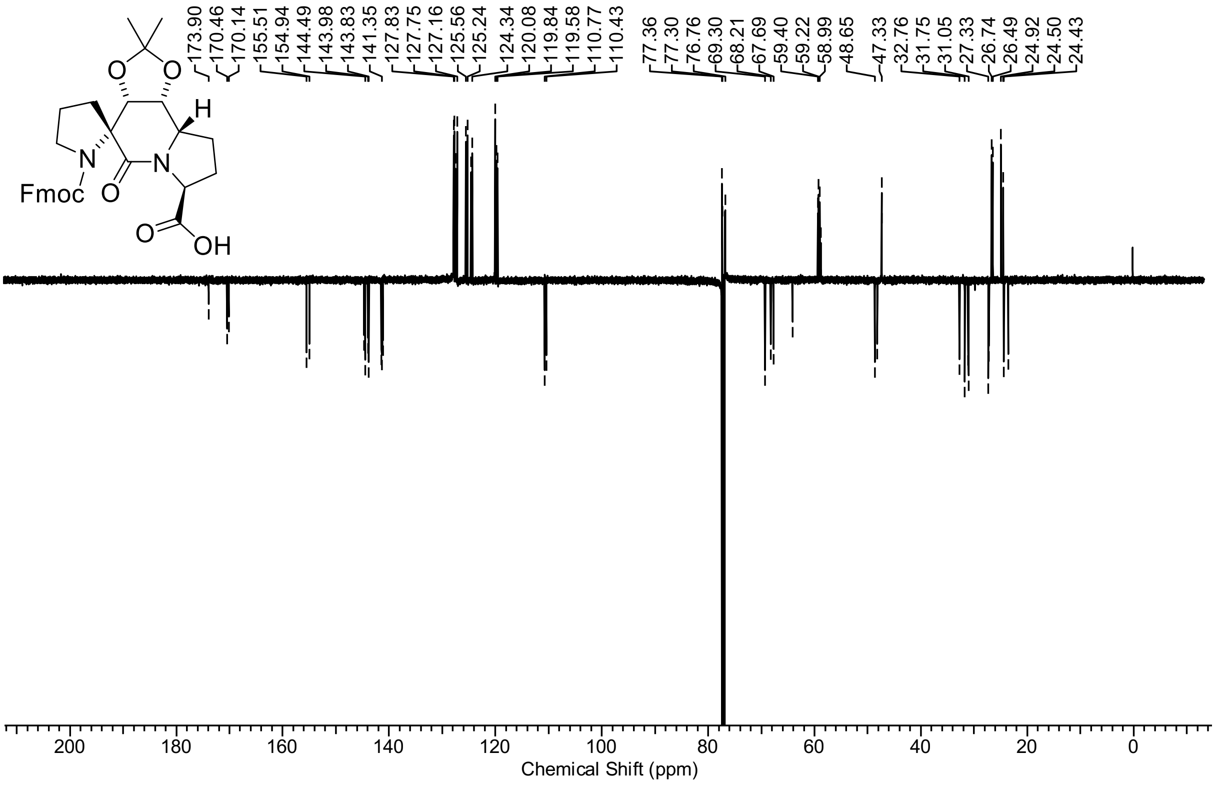

Figure S70. ^{13}C-APT-NMR spectrum of Fmoc-**(Me_2CO_2)-ProM2**-OH in $CDCl_3$ at 125 MHz.

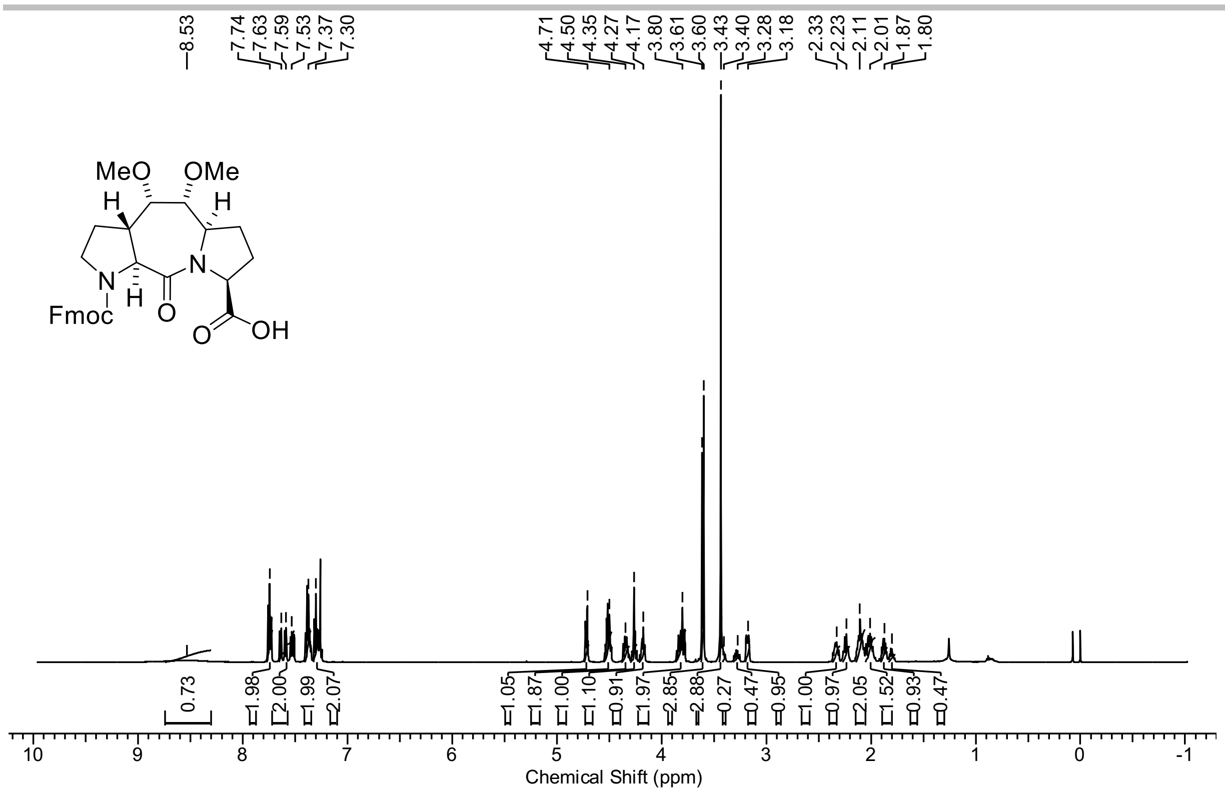

Figure S71. ^{1}H-NMR spectrum of Fmoc-**(MeO)$_2$-ProM1**-OH in $CDCl_3$ at 500 MHz (full view).

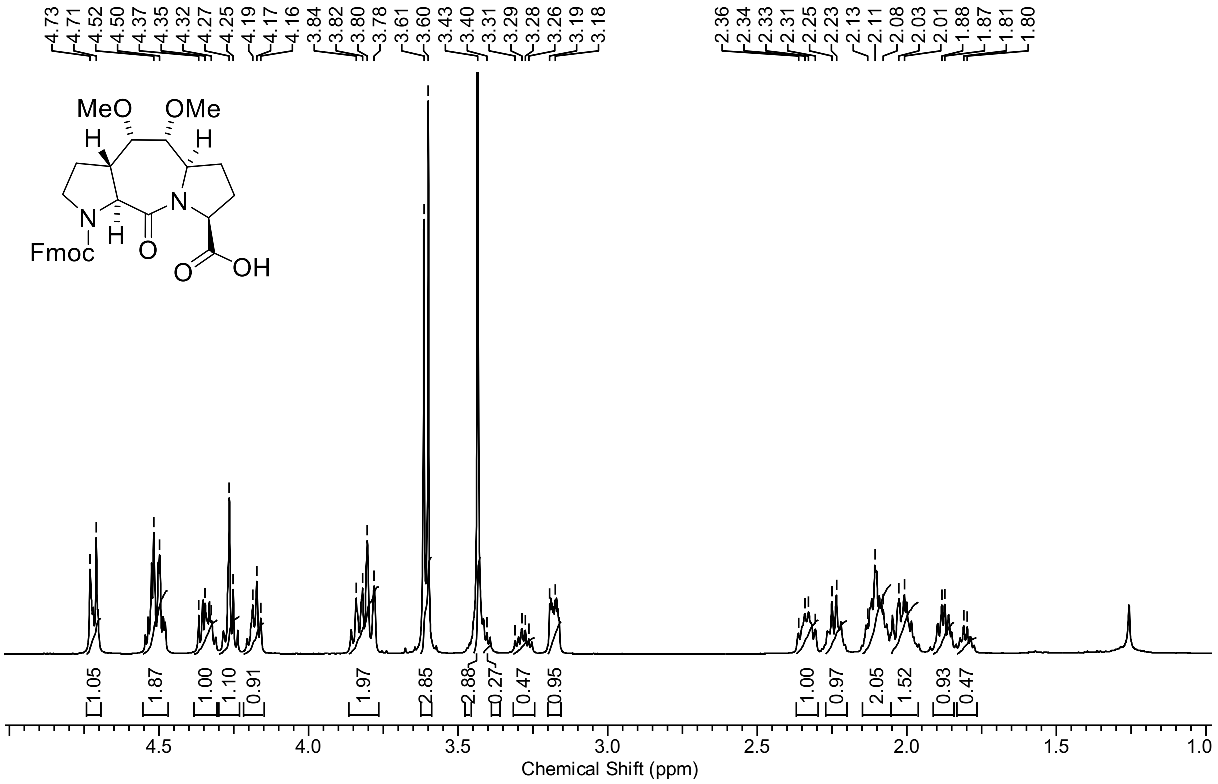

Figure S72. ^{1}H-NMR spectrum of Fmoc-**(MeO)$_2$-ProM1**-OH in $CDCl_3$ at 500 MHz (zoom in).

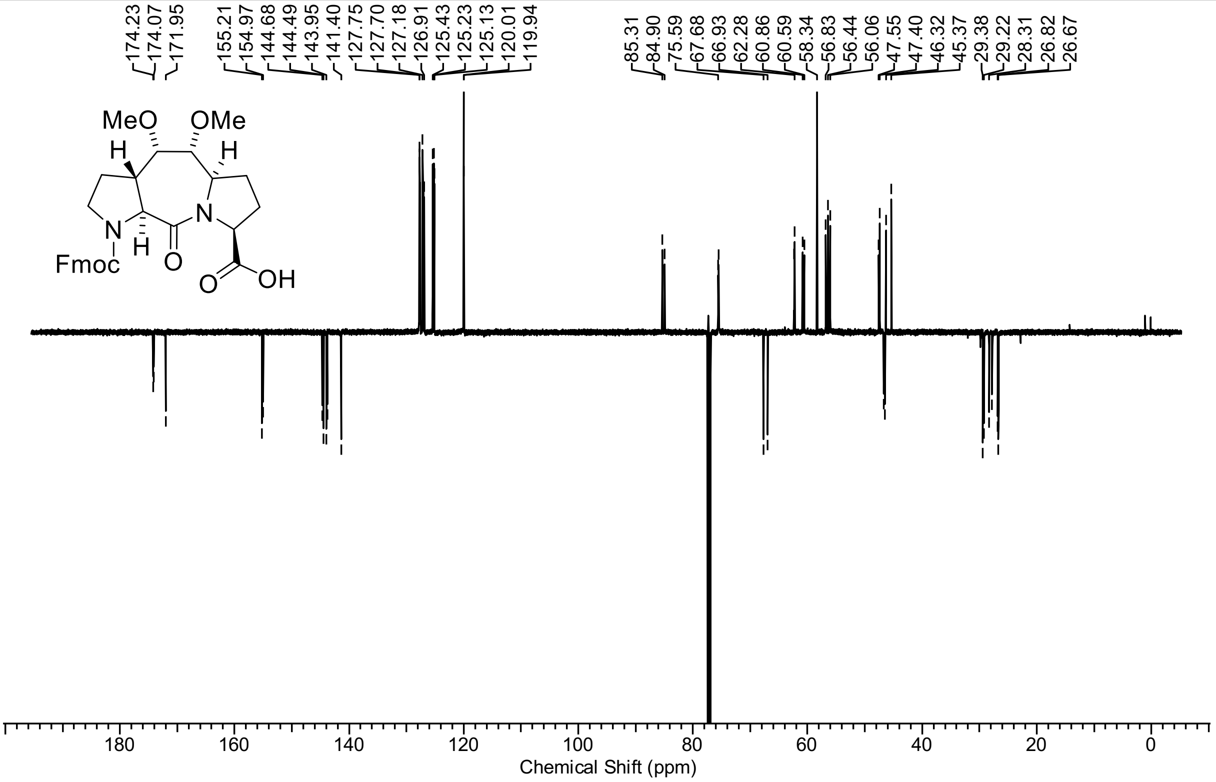

Figure S73. ^{13}C-APT-NMR spectrum of Fmoc-**(MeO)$_2$-ProM1**-OH in $CDCl_3$ at 125 MHz.

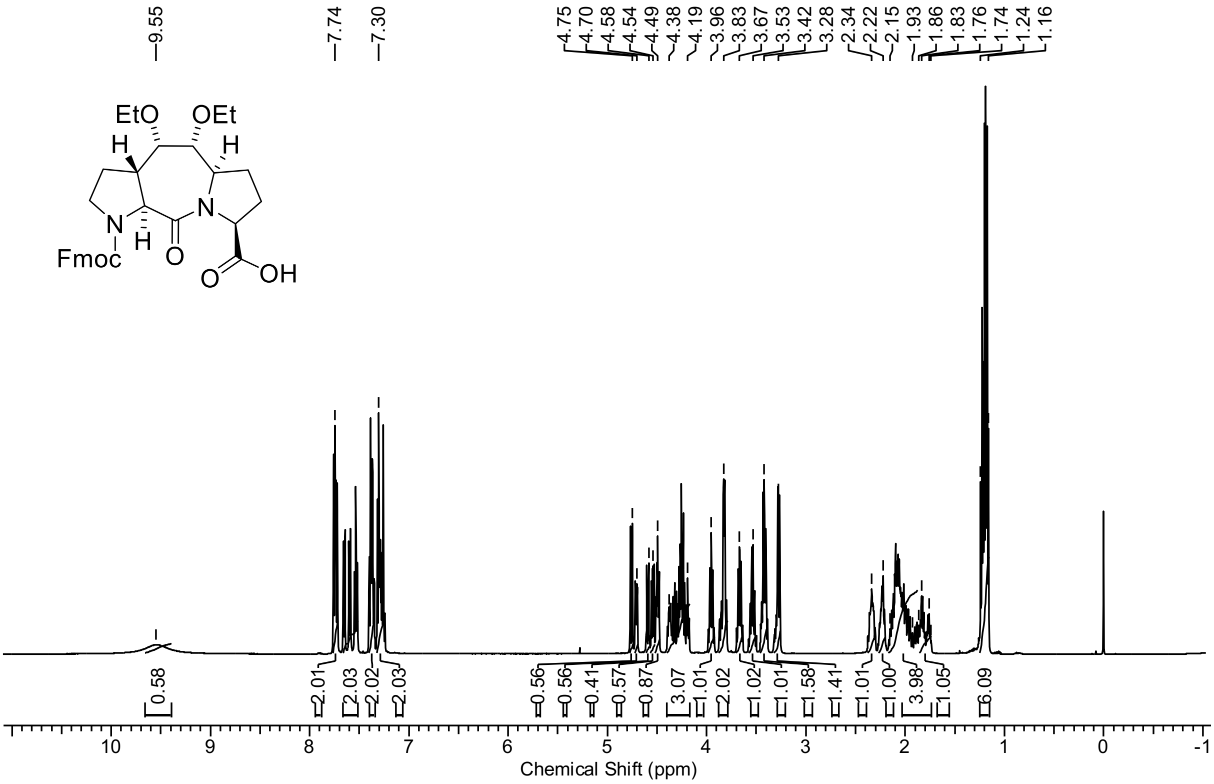

Figure S74. ^{1}H-NMR spectrum of Fmoc-**(EtO)$_2$-ProM1**-OH in $CDCl_3$ at 500 MHz (full view).

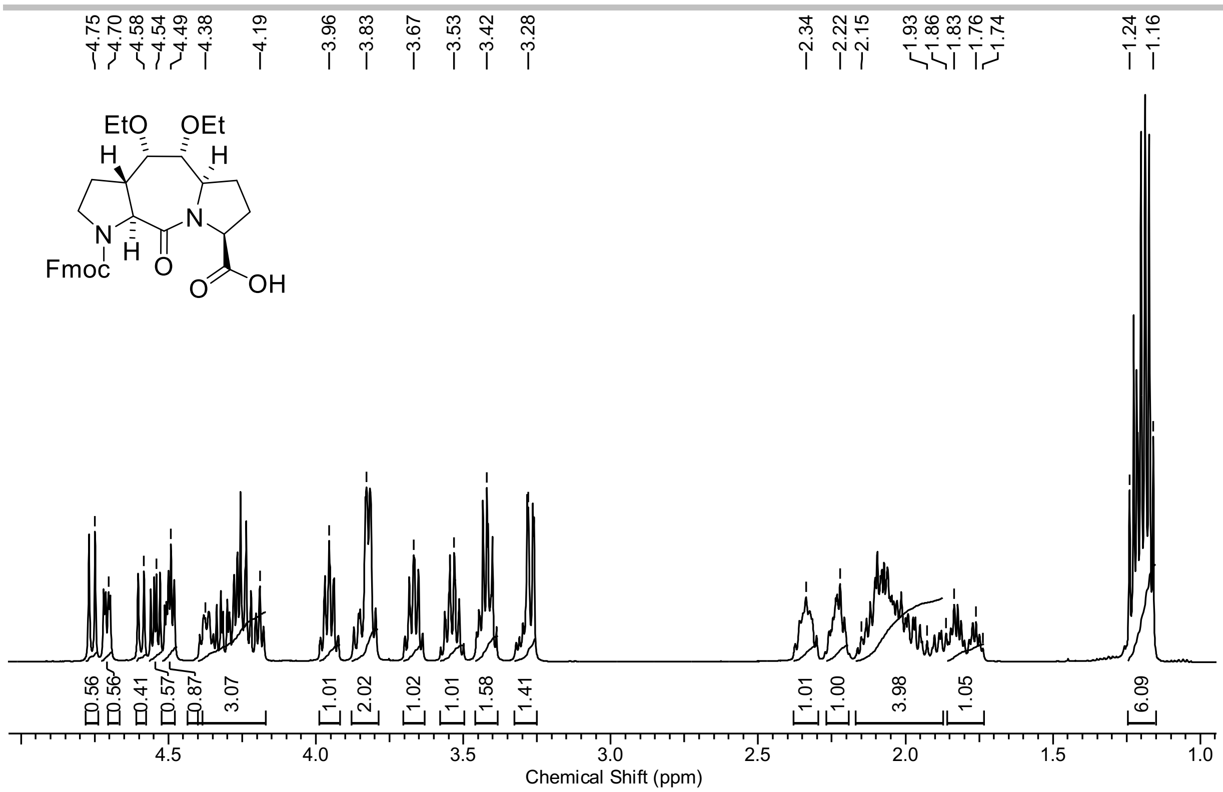

Figure S75. ^{1}H-NMR spectrum of Fmoc-**(EtO)$_2$-ProM1**-OH in $CDCl_3$ at 500 MHz (zoom in).

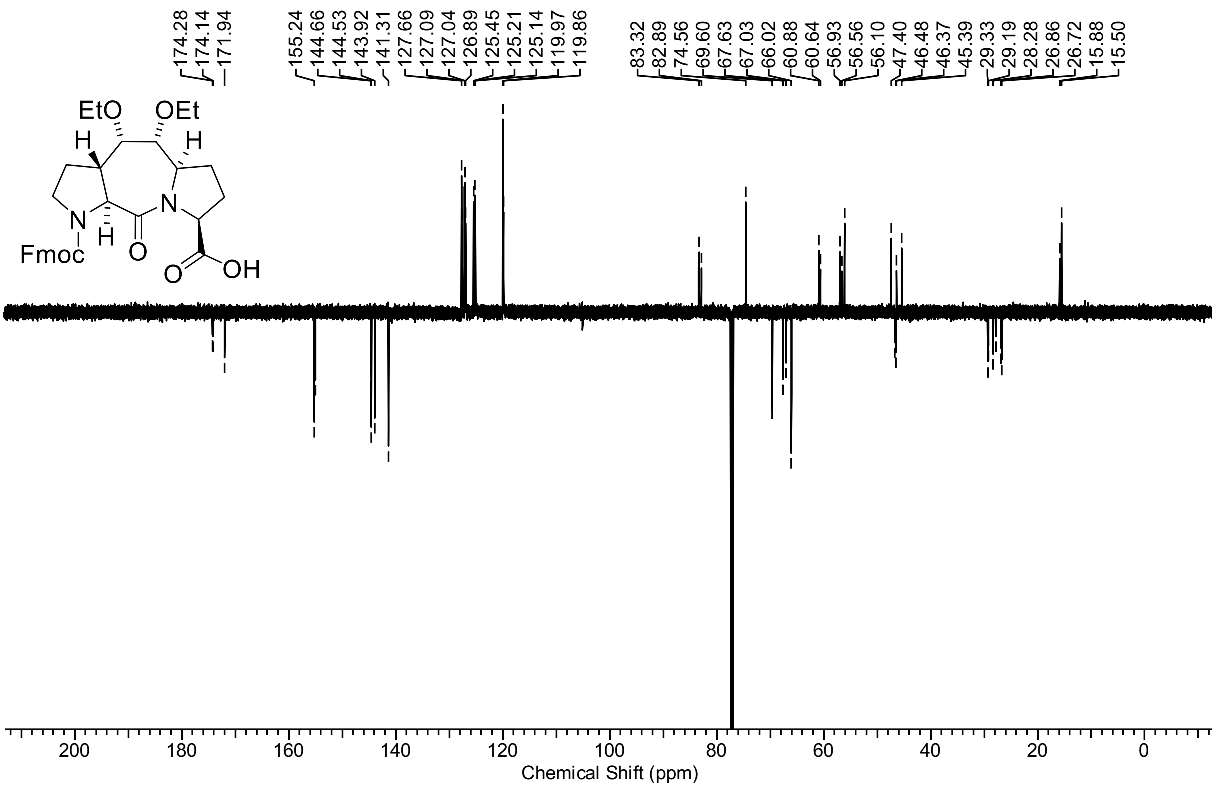

Figure S76. ^{13}C-APT-NMR spectrum of Fmoc-**(EtO)$_2$-ProM1**-OH in $CDCl_3$ at 150 MHz.

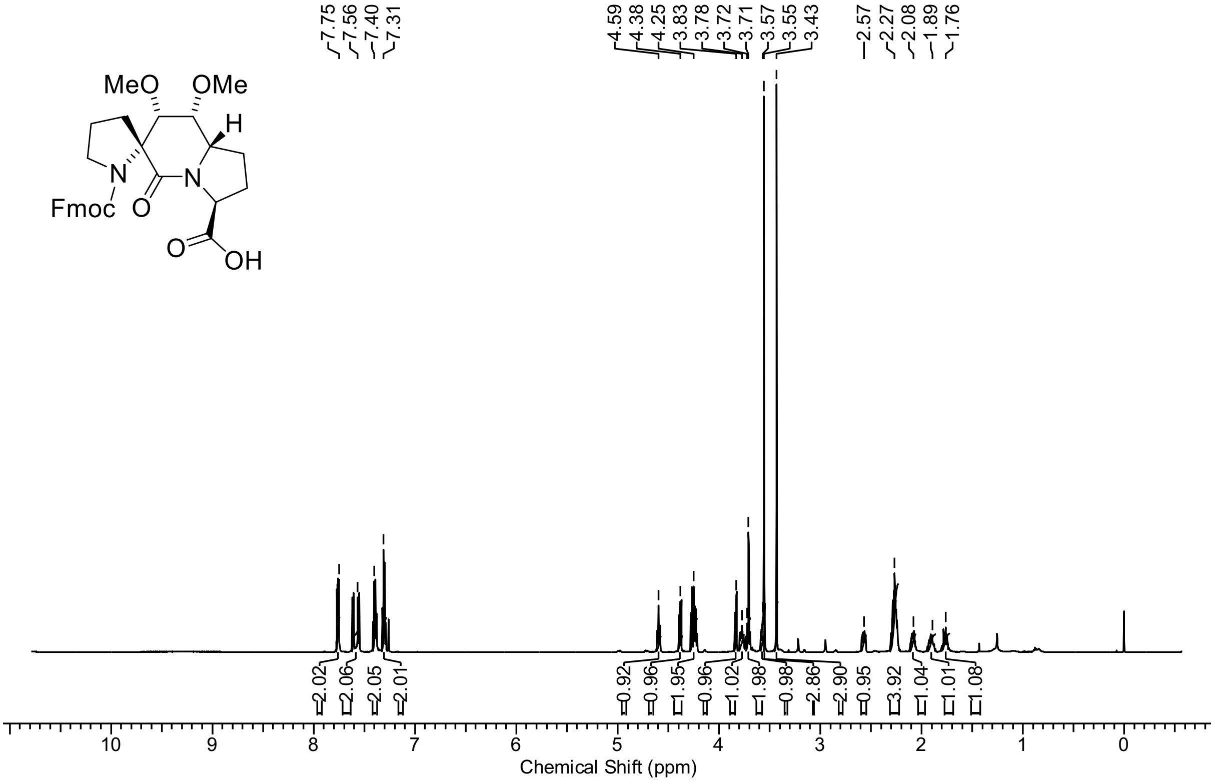

Figure S77. ^{1}H-NMR spectrum of Fmoc-**(MeO)$_2$-ProM2**-OH in $CDCl_3$ at 600 MHz (full view).

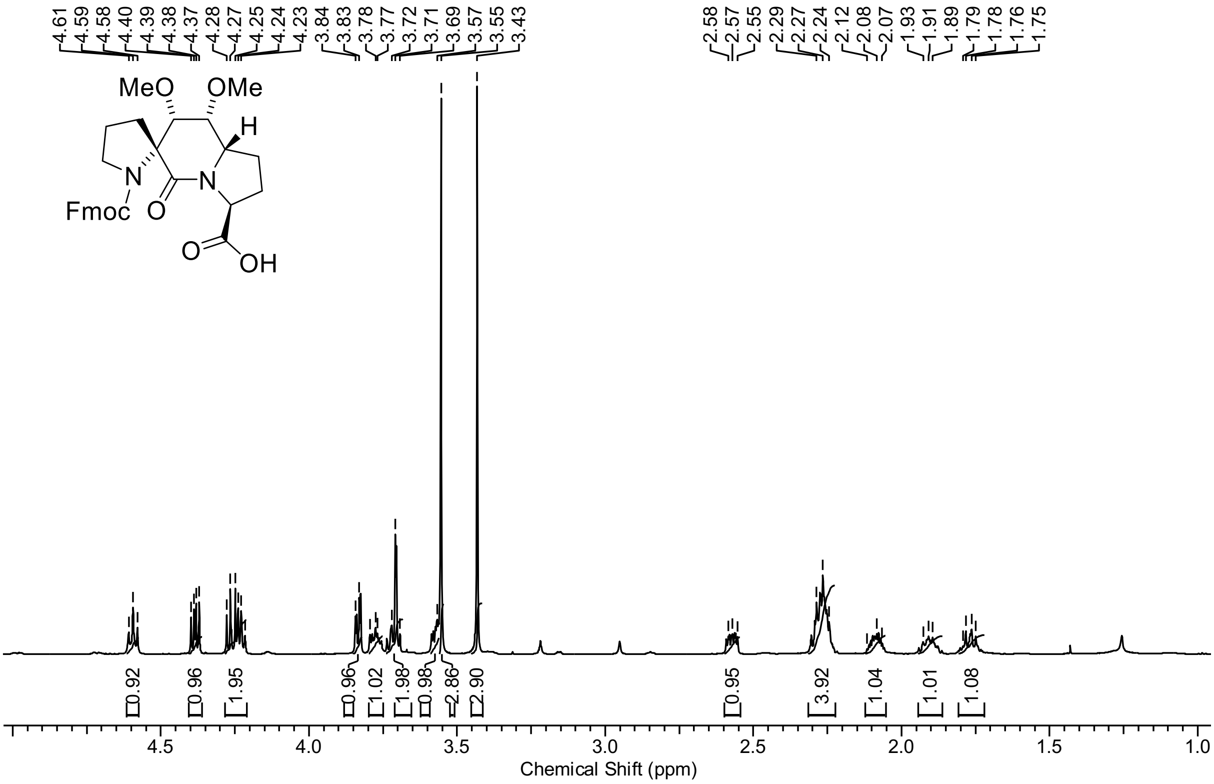

Figure S78. ^{1}H-NMR spectrum of Fmoc-**(MeO)$_2$-ProM2**-OH in $CDCl_3$ at 600 MHz (zoom in).

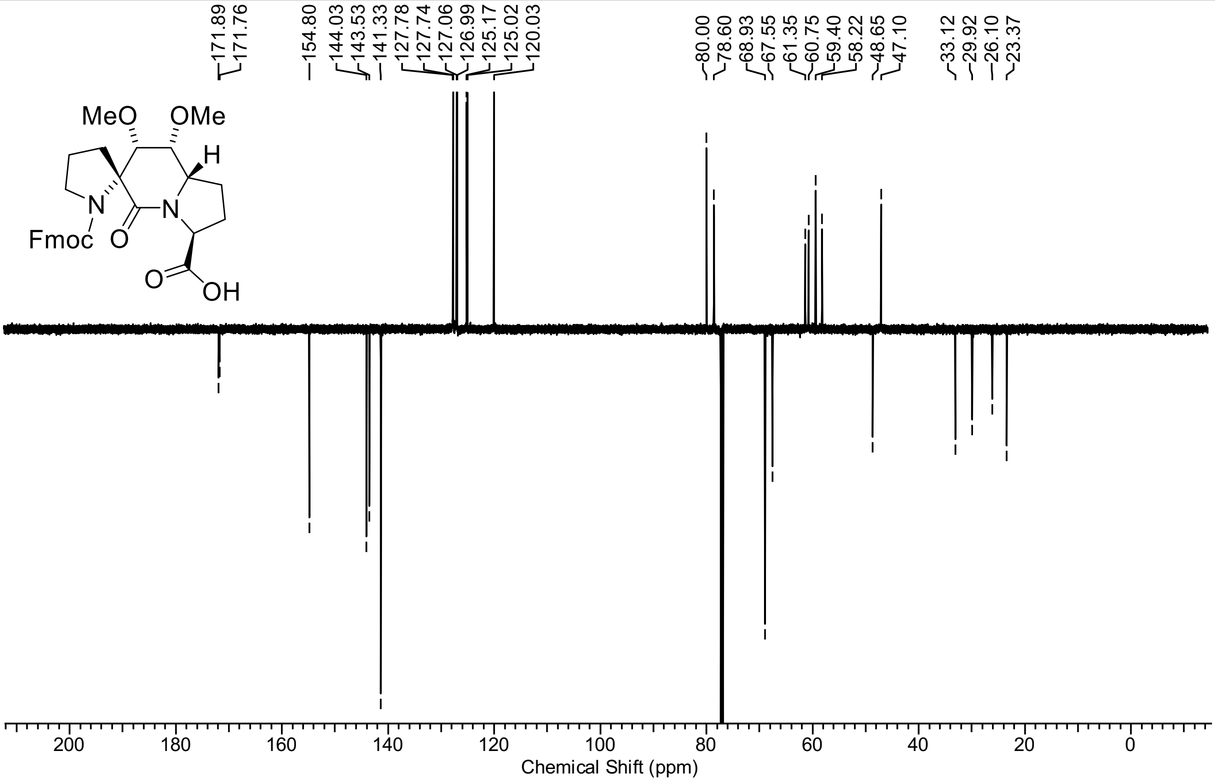

Figure S79. ^{13}C-APT-NMR spectrum of Fmoc-**(MeO)$_2$-ProM2**-OH in $CDCl_3$ at 150 MHz.

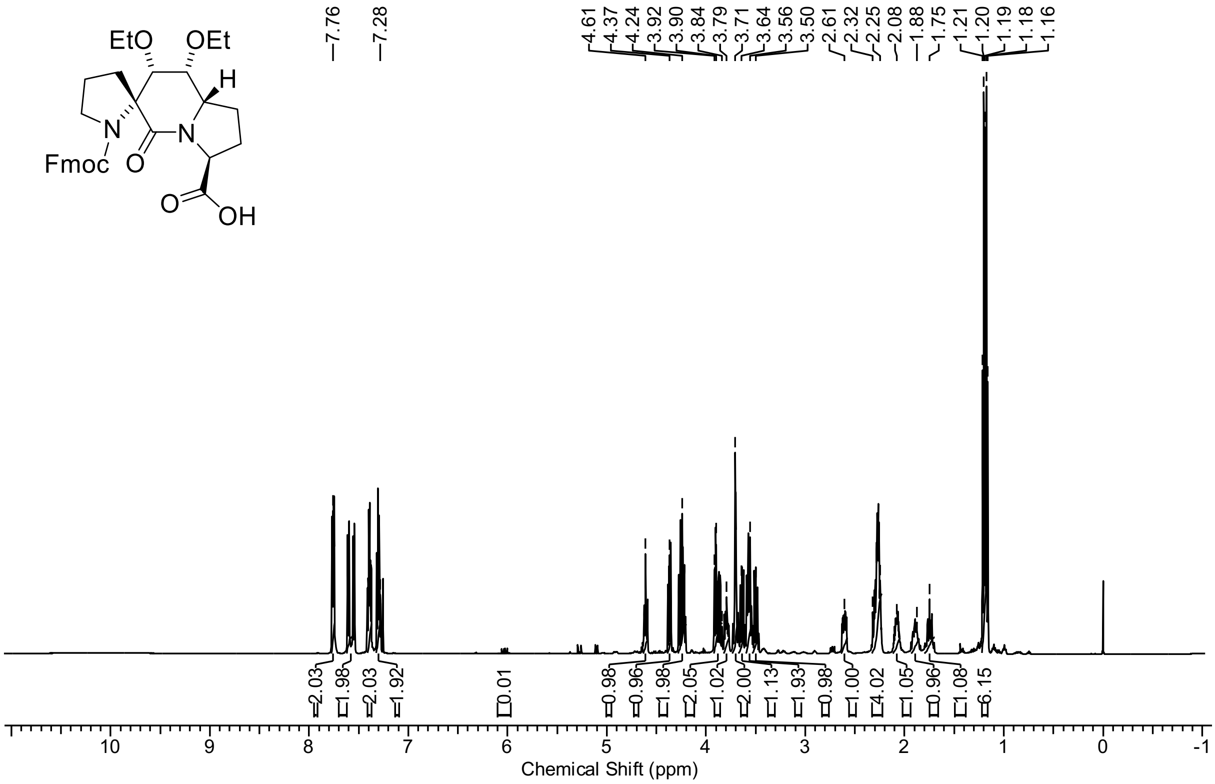

Figure S80. ^{1}H-NMR spectrum of Fmoc-**(EtO)$_2$-ProM2**-OH in $CDCl_3$ at 500 MHz (full view).

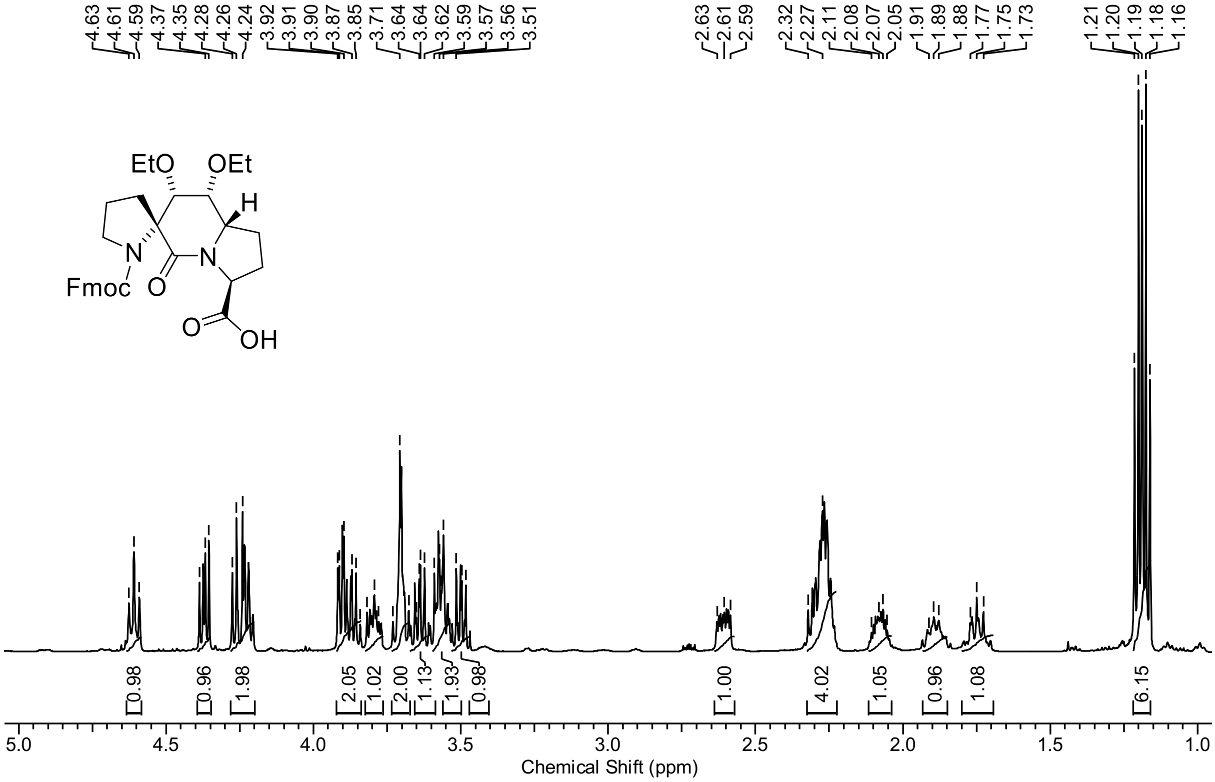

Figure S81. ^{1}H-NMR spectrum of Fmoc-**(EtO)$_2$-ProM2**-OH in $CDCl_3$ at 500 MHz (zoom in).

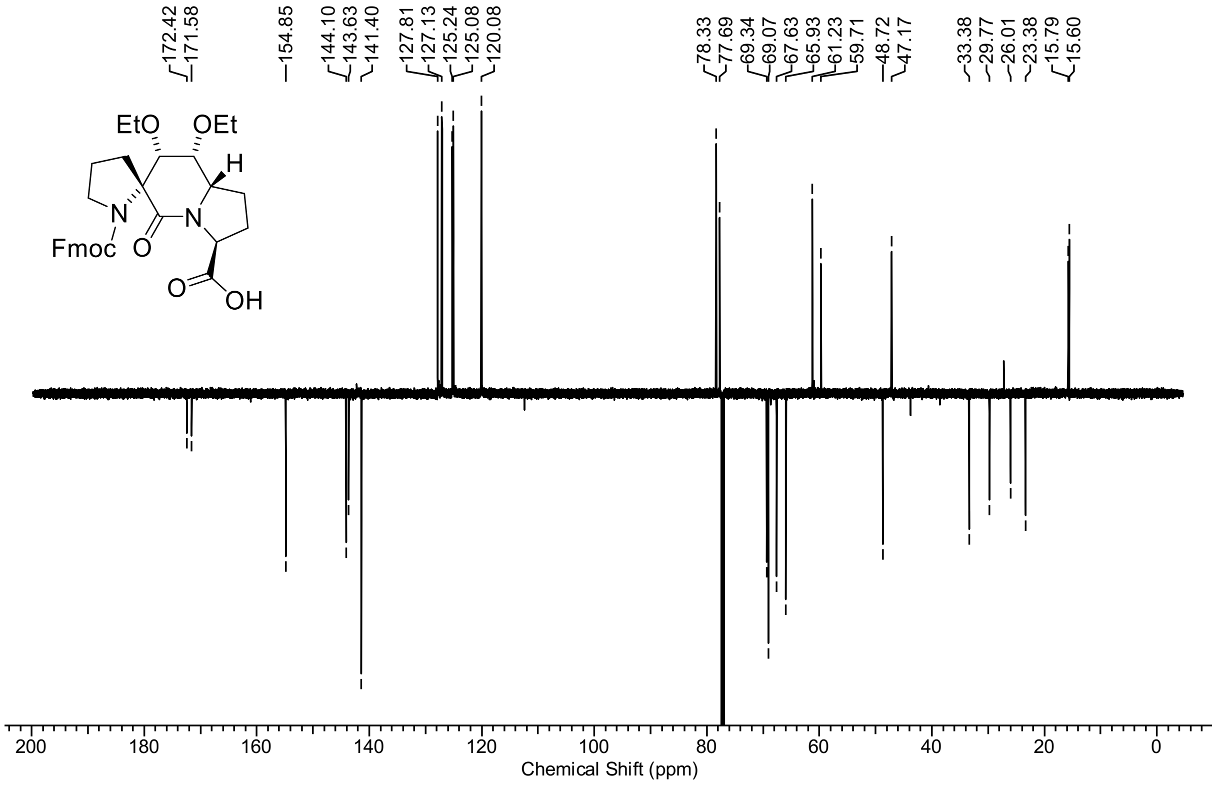

Figure S82. ^{13}C-APT-NMR spectrum of Fmoc-**(EtO)$_2$-ProM2**-OH in $CDCl_3$ at 150 MHz.

ESI-MS spectra of peptides

Ac-PPG-PPG-PPG-PPG-PPG-PRG-PPG-PPG-PPG-PPG-PPG-NH_2

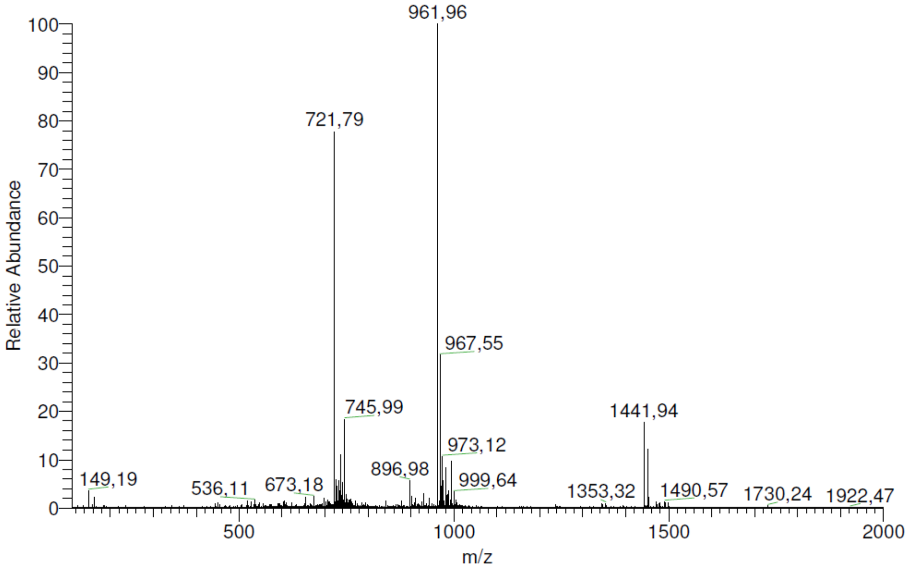

MS (ESI):	$[M+4H]^{4+}$	$[M+3H]^{3+}$	$[M+2H+NH_4]^{3+}$	$[M+2H]^{2+}$	$[M+H+NH_4]^{2+}$
m/z calcd for $C_{135}H_{197}N_{37}O_{34}$	721.1	961.2	966.8	1441.3	1449.8
found	721.8	962.0	967.5	1441.9	1450.3

Ac-PPG-PPG-PPG-PPG-PPG-PRG-PPG-POG-PPG-PPG-PPG-NH_2

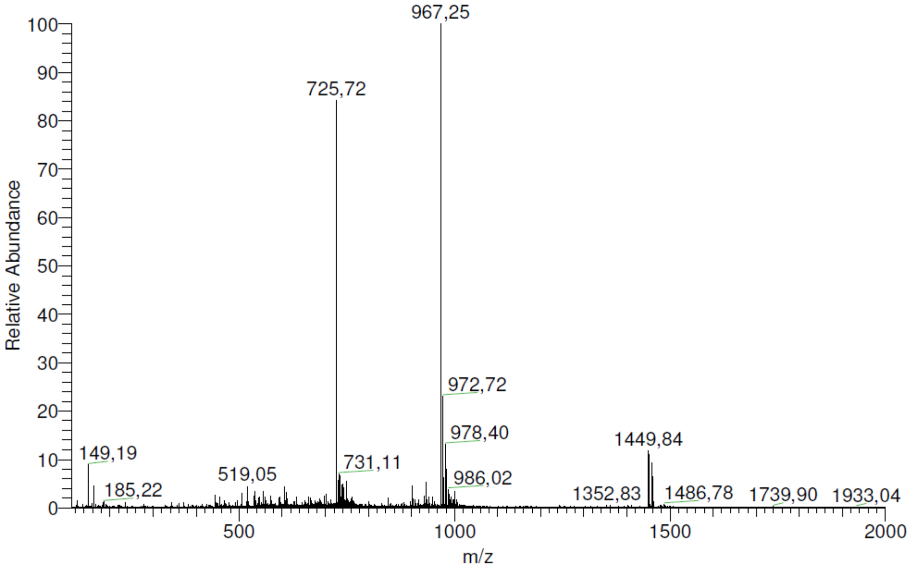

MS (ESI):	$[M+4H]^{4+}$	$[M+3H]^{3+}$	$[M+2H+NH_4]^{3+}$	$[M+2H]^{2+}$	$[M+H+NH_4]^{2+}$
m/z calcd for $C_{135}H_{197}N_{37}O_{35}$	725.1	966.5	972.2	1449.3	1457.8
found	725.7	967.3	972.6	1449.8	1458.3

Ac-PPG-PPG-PPG-PPG-PPG-PRG-PPG-OPG-PPG-PPG-PPG-NH$_2$

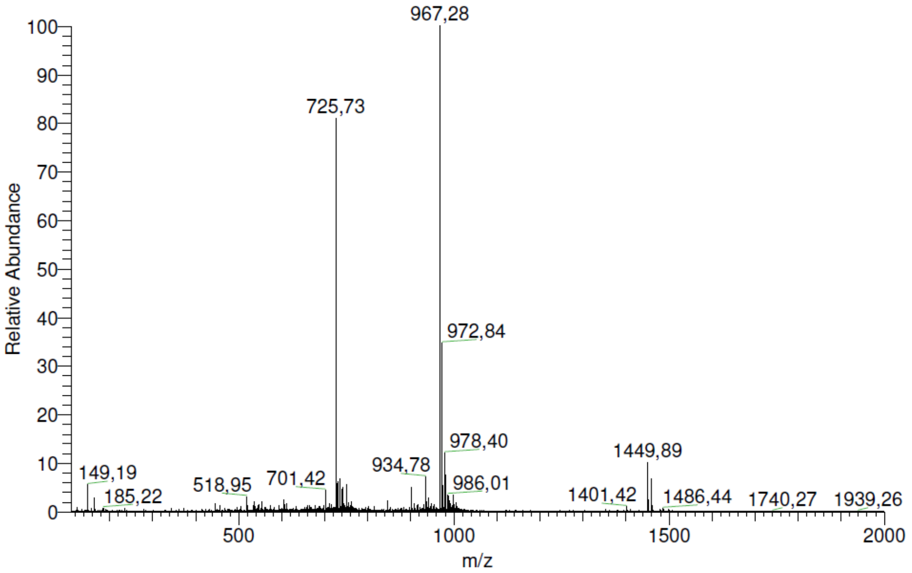

MS (ESI):	$[M+4H]^{4+}$	$[M+3H]^{3+}$	$[M+2H+NH_4]^{3+}$	$[M+2H]^{2+}$	$[M+H+NH_4]^{2+}$
m/z calcd for $C_{135}H_{197}N_{37}O_{35}$	725.1	966.5	972.2	1449.3	1457.8
found	725.7	967.3	972.8	1449.9	1458.8

Ac-PPG-PPG-PPG-PPG-PPG-PRG-PPG-OOG-PPG-PPG-PPG-NH$_2$

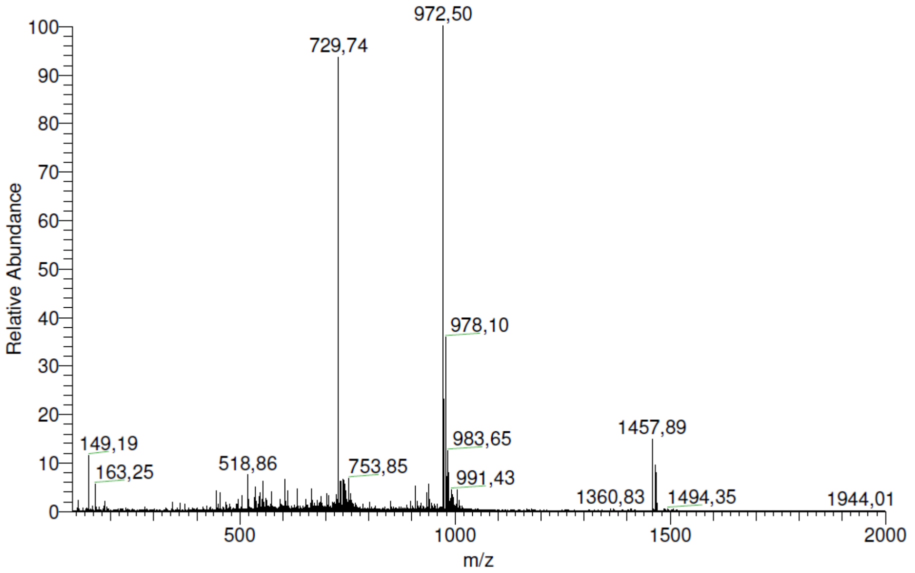

MS (ESI):	$[M+4H]^{4+}$	$[M+3H]^{3+}$	$[M+2H+NH_4]^{3+}$	$[M+2H]^{2+}$	$[M+H+NH_4]^{2+}$
m/z calcd for $C_{135}H_{197}N_{37}O_{36}$	729.1	971.8	977.5	1457.2	1465.8
found	729.7	972.5	978.2	1457.8	1466.3

Ac-PPG-PPG-PPG-PPG-PPG-PRG-PPG-[ProM1]G-PPG-PPG-PPG-NH$_2$

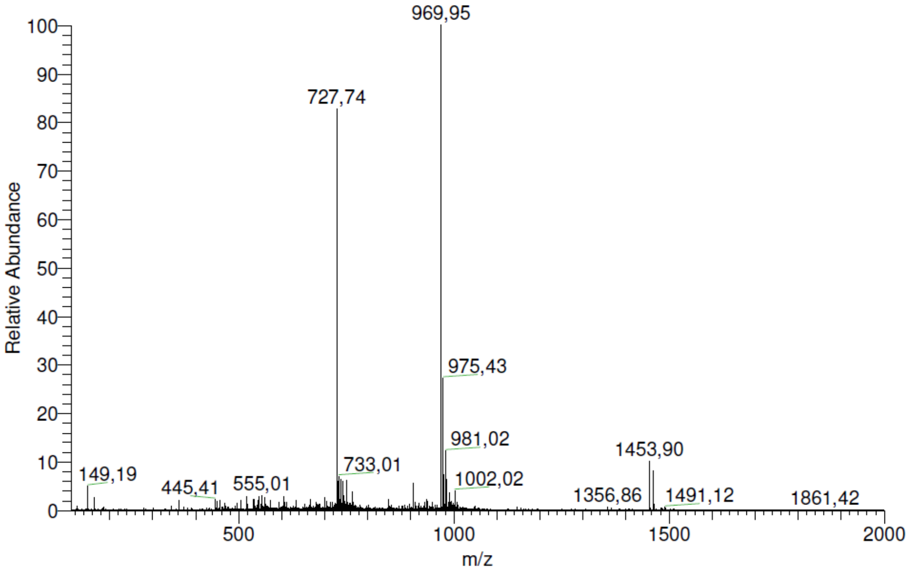

MS (ESI):	$[M+4H]^{4+}$	$[M+3H]^{3+}$	$[M+2H+NH_4]^{3+}$	$[M+2H]^{2+}$	$[M+H+NH_4]^{2+}$
m/z calcd for $C_{137}H_{197}N_{37}O_{34}$	727.1	969.2	974.8	1453.3	1461.8
found	727.7	969.9	975.4	1453.9	1462.3

Ac-PPG-PPG-PPG-PPG-PPG-PRG-PPG-[H$_2$-ProM1]G-PPG-PPG-PPG-NH$_2$

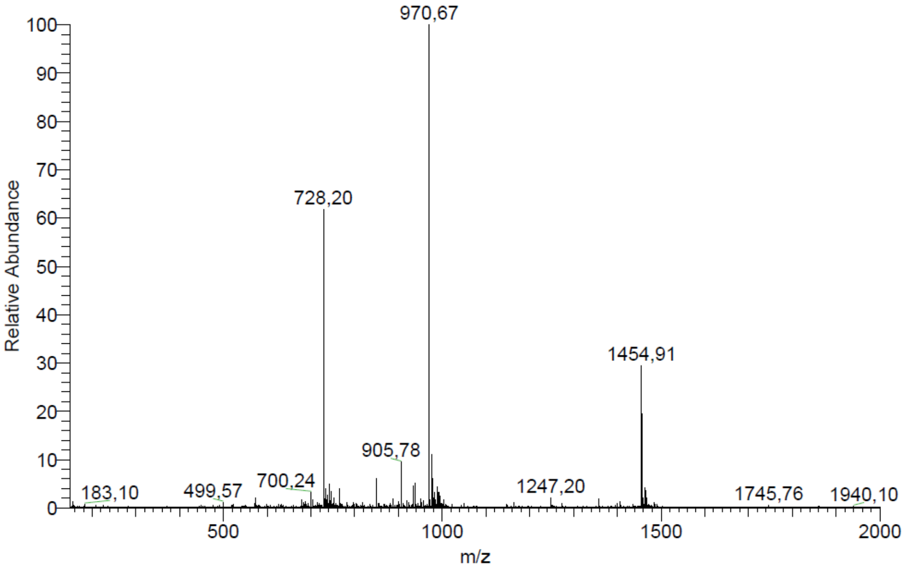

MS (ESI):	$[M+4H]^{4+}$	$[M+3H]^{3+}$	$[M+2H]^{2+}$
m/z calcd for $C_{137}H_{199}N_{37}O_{34}$	727.6	969.8	1454.3
found	728.2	970.6	1454.9

Ac-PPG-PPG-PPG-PPG-PPG-PRG-PPG-[HO-ProM1]G-PPG-PPG-PPG-NH$_2$

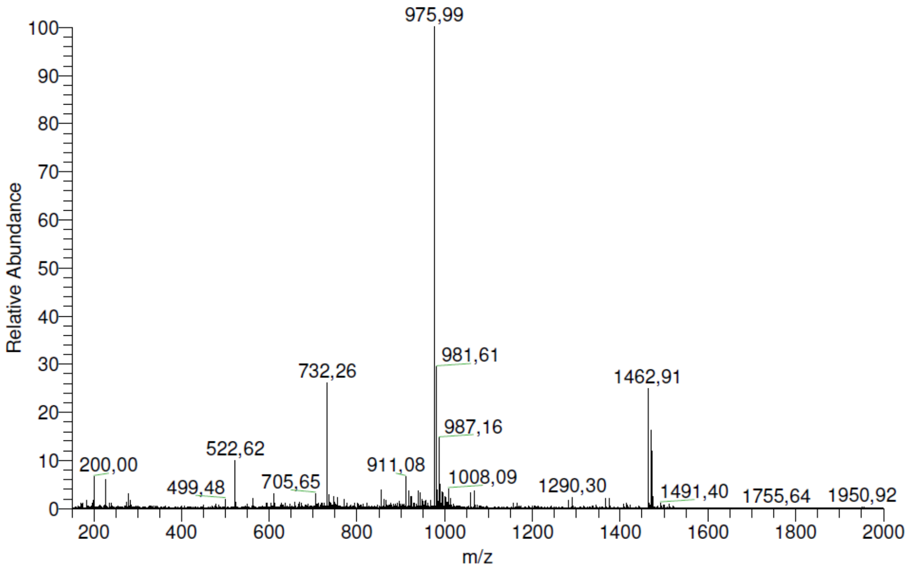

MS (ESI):	[M+4H]$^{4+}$	[M+3H]$^{3+}$	[M+2H+NH$_4$]$^{3+}$	[M+2H]$^{2+}$	[M+H+NH$_4$]$^{2+}$
m/z calcd for $C_{137}H_{199}N_{37}O_{35}$	731.6	975.2	980.9	1462.3	1470.8
found	732.3	976.0	981.6	1462.9	1471.4

Ac-PPG-PPG-PPG-PPG-PPG-PRG-PPG-[(HO)$_2$-ProM1]G-PPG-PPG-PPG-NH$_2$

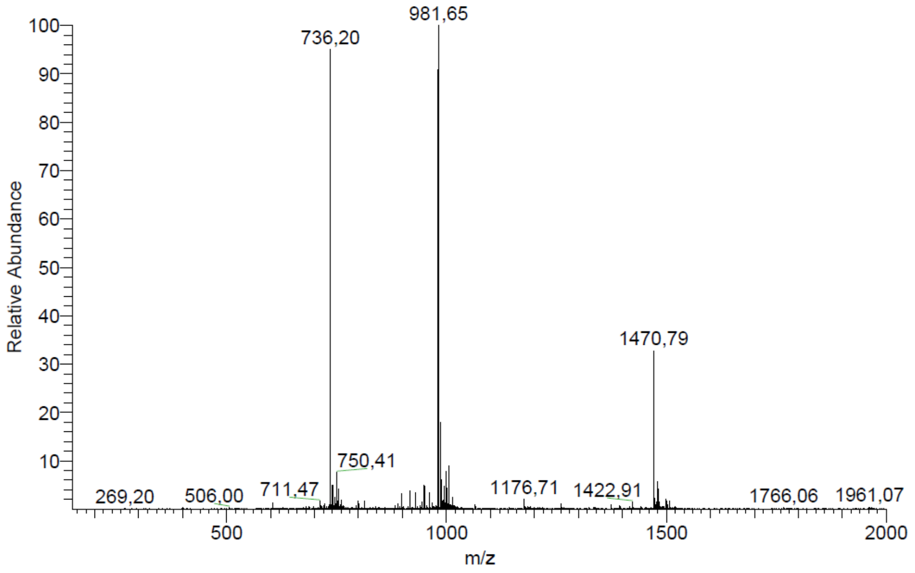

MS (ESI):	[M+4H]$^{4+}$	[M+3H]$^{3+}$	[M+2H]$^{2+}$
m/z calcd for $C_{137}H_{199}N_{37}O_{36}$	735.6	980.5	1470.3
found	736.2	981.6	1470.8

Ac-PPG-PPG-PPG-PPG-PPG-PRG-PPG-[(MeO)$_2$-ProM1]G-PPG-PPG-PPG-NH$_2$

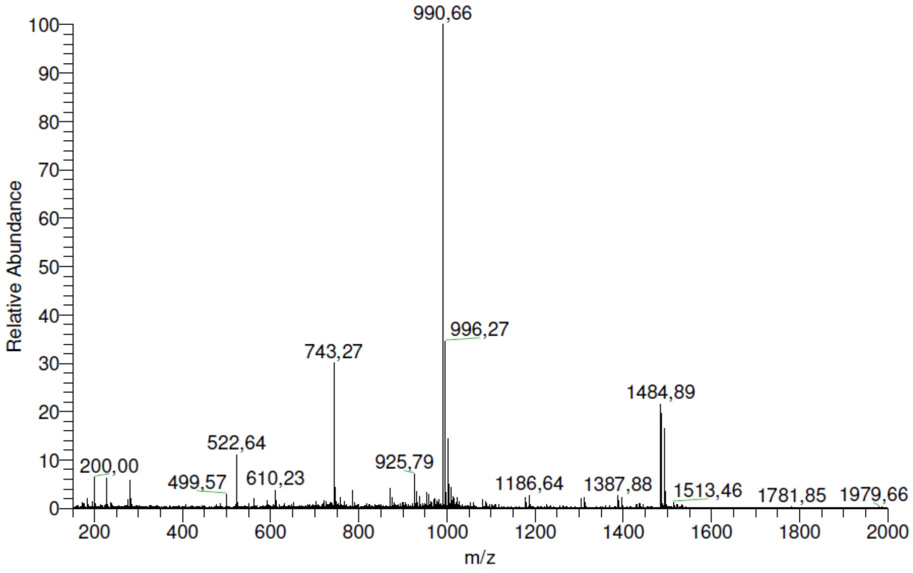

MS (ESI):	$[M+4H]^{4+}$	$[M+3H]^{3+}$	$[M+2H+NH_4]^{3+}$	$[M+2H]^{2+}$	$[M+H+NH_4]^{2+}$
m/z calcd for $C_{139}H_{203}N_{37}O_{36}$	742.6	989.9	995.5	1484.3	1492.8
found	743.3	990.7	996.3	1484.8	1493.4

Ac-PPG-PPG-PPG-PPG-PPG-PRG-PPG-[(EtO)$_2$-ProM1]G-PPG-PPG-PPG-NH$_2$

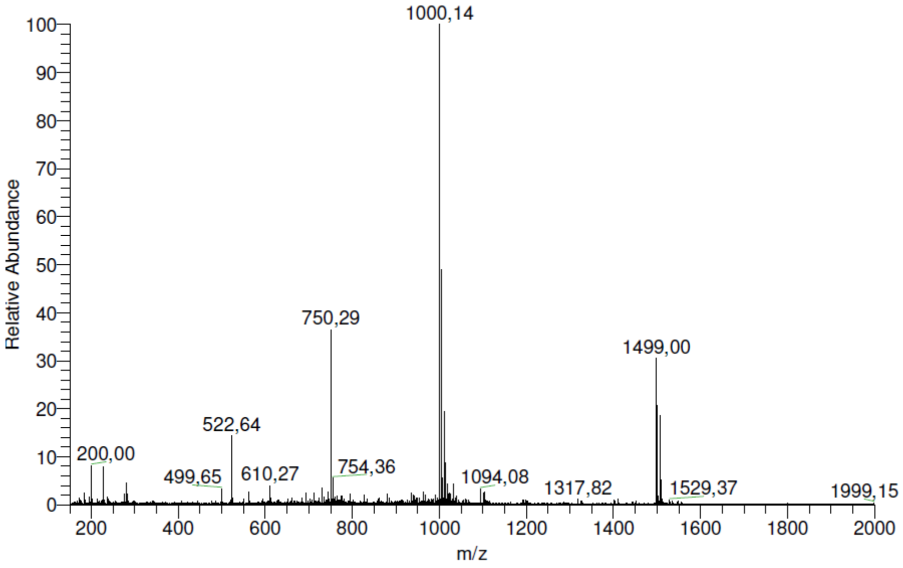

MS (ESI):	$[M+4H]^{4+}$	$[M+3H]^{3+}$	$[M+2H+NH_4]^{3+}$	$[M+2H]^{2+}$	$[M+H+NH_4]^{2+}$
m/z calcd for $C_{141}H_{207}N_{37}O_{36}$	749.7	999.2	1004.9	1498.3	1506.8
found	750.3	1000.1	1005.5	1499.0	1507.5

Ac-PPG-PPG-PPG-PPG-PPG-PRG-PPG-[ProM2]G-PPG-PPG-PPG-NH$_2$

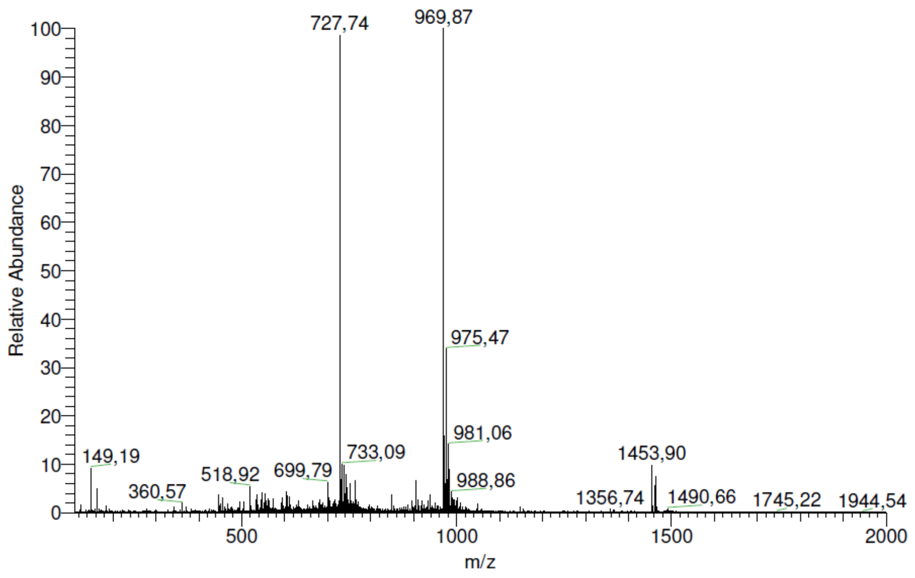

MS (ESI):	$[M+4H]^{4+}$	$[M+3H]^{3+}$	$[M+2H+NH_4]^{3+}$	$[M+2H]^{2+}$	$[M+H+NH_4]^{2+}$
m/z calcd for $C_{137}H_{197}N_{37}O_{34}$	727.1	969.2	974.8	1453.3	1461.8
found	727.7	969.9	975.4	1453.9	1462.6

Ac-PPG-PPG-PPG-PPG-PPG-PRG-PPG-[H$_2$-ProM2]G-PPG-PPG-PPG-NH$_2$

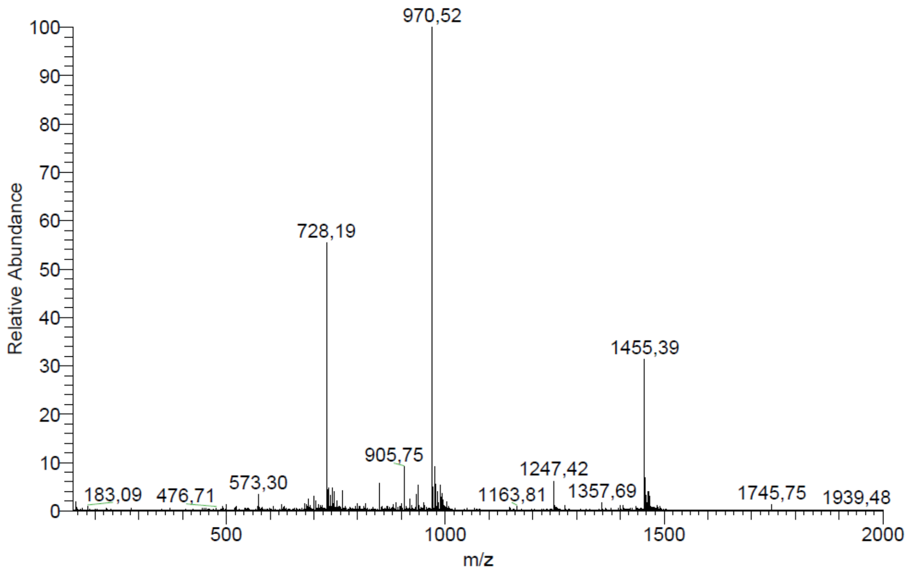

MS (ESI):	$[M+4H]^{4+}$	$[M+3H]^{3+}$	$[3M+7H]^{7+}$	$[M+2H]^{2+}$	$[3M+5H]^{5+}$
m/z calcd for $C_{137}H_{199}N_{37}O_{34}$	727.6	969.8	1246.7	1454.3	1744.9
found	728.1	970.5	1247.4	1455.3	1745.7

Ac-PPG-PPG-PPG-PPG-PPG-PRG-PPG-[(HO)$_2$-ProM2]G-PPG-PPG-PPG-NH$_2$

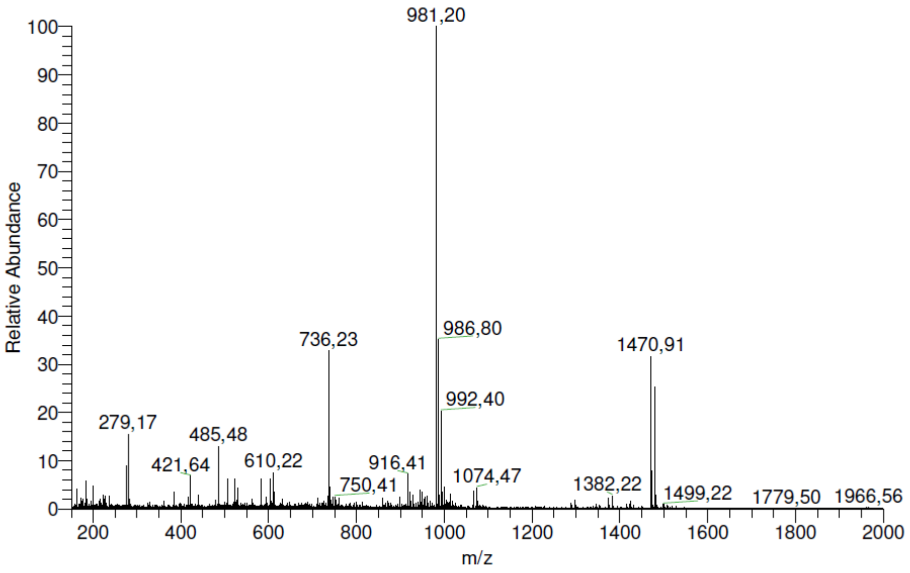

MS (ESI):	$[M+4H]^{4+}$	$[M+3H]^{3+}$	$[M+2H+NH_4]^{3+}$	$[M+2H]^{2+}$	$[M+H+NH_4]^{2+}$
m/z calcd for $C_{137}H_{199}N_{37}O_{36}$	735.6	980.5	986.2	1470.3	1478.8
found	736.2	981.2	986.8	1470.9	1479.3

Ac-PPG-PPG-PPG-PPG-PPG-PRG-PPG-[(MeO)$_2$-ProM2]G-PPG-PPG-PPG-NH$_2$

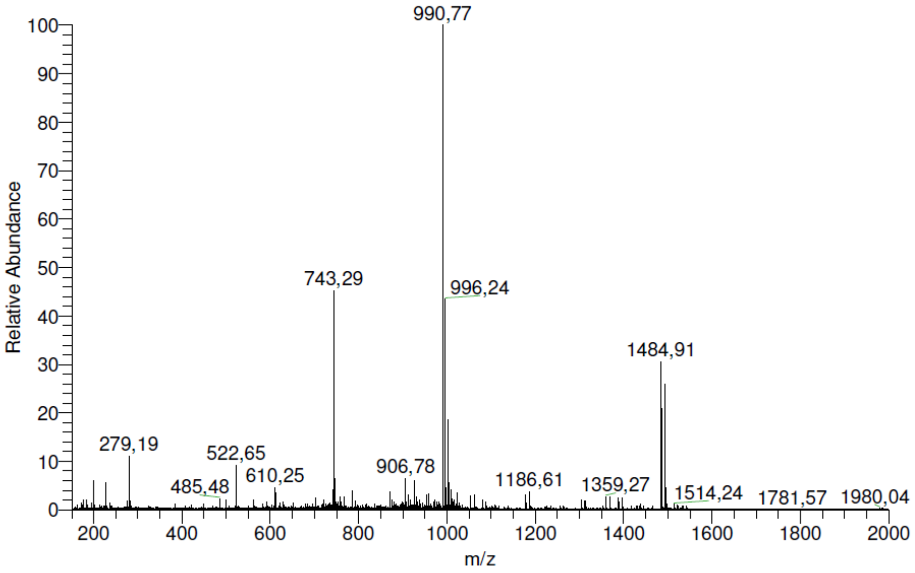

MS (ESI):	$[M+4H]^{4+}$	$[M+3H]^{3+}$	$[M+2H+NH_4]^{3+}$	$[M+2H]^{2+}$	$[M+H+NH_4]^{2+}$
m/z calcd for $C_{139}H_{203}N_{37}O_{36}$	742.6	989.9	995.5	1484.3	1492.8
found	743.3	990.7	996.2	1484.9	1493.4

Ac-PPG-PPG-PPG-PPG-PPG-PRG-PPG-[(EtO)$_2$-ProM2]G-PPG-PPG-PPG-NH$_2$

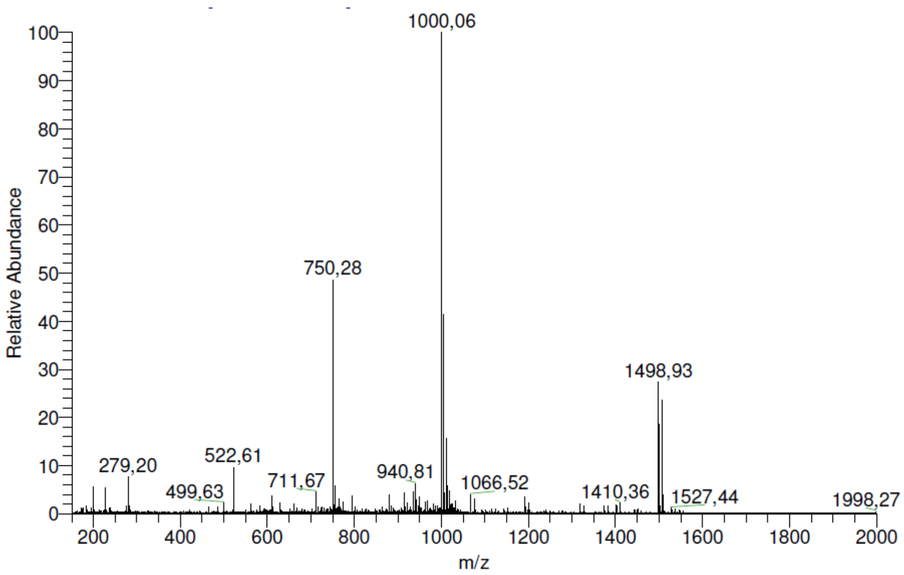

MS (ESI):	[M+4H]$^{4+}$	[M+3H]$^{3+}$	[M+2H+NH$_4$]$^{3+}$	[M+2H]$^{2+}$	[M+H+NH$_4$]$^{2+}$
m/z calcd for $C_{141}H_{207}N_{37}O_{36}$	749.7	999.2	1004.9	1498.3	1506.8
found	750.3	1000.0	1005.6	1498.9	1507.4

Ac-PPG-PPG-PPG-[ProM1]G-PPG-PRG-PPG-PPG-PPG-PPG-PPG-NH$_2$

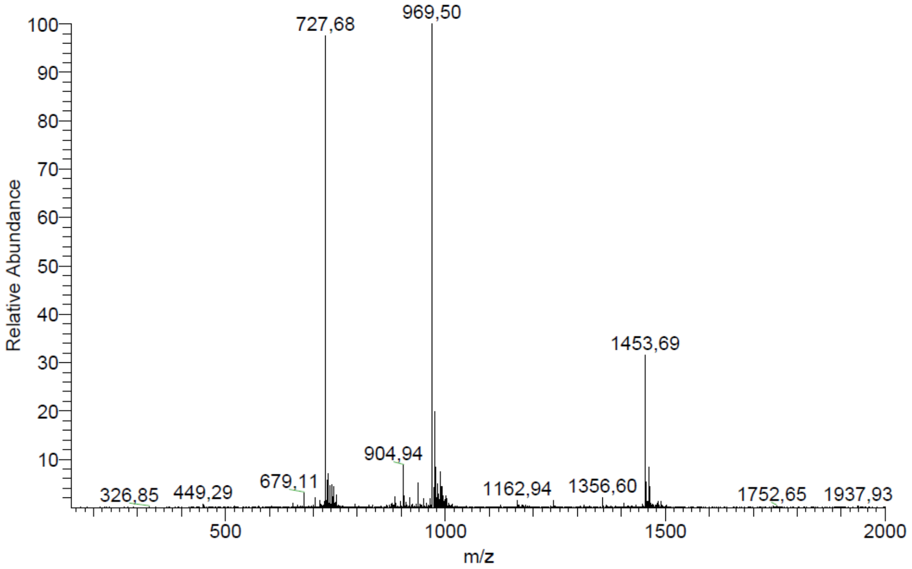

MS (ESI):	[M+4H]$^{4+}$	[M+3H]$^{3+}$	[M+2H]$^{2+}$
m/z calcd for $C_{137}H_{197}N_{37}O_{34}$	727.1	969.2	1453.3
found	727.6	969.5	1453.6

Ac-PPG-PPG-PPG-[H2-ProM2]G-PPG-PRG-PPG-PPG-PPG-PPG-PPG-NH2

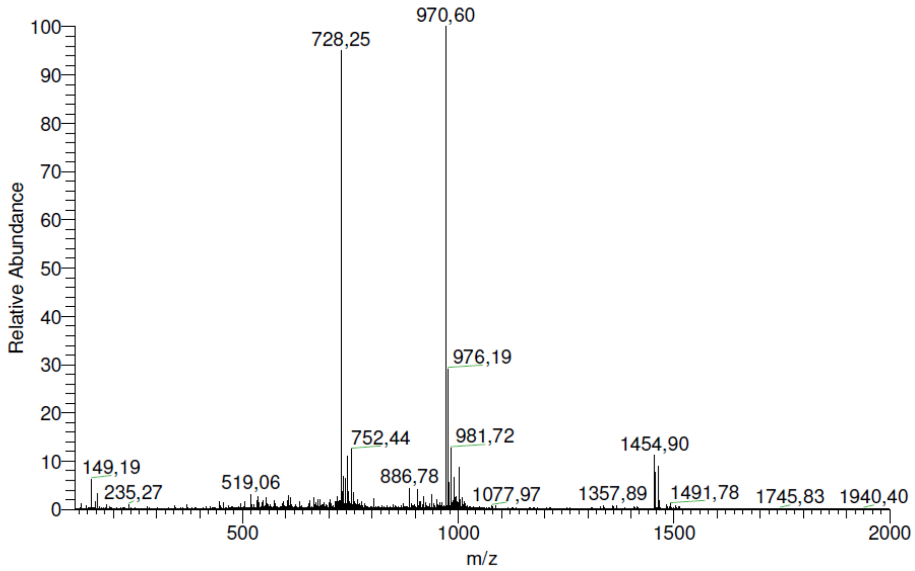

MS (ESI):	$[M+4H]^{4+}$	$[M+3H]^{3+}$	$[M+2H+NH_4]^{3+}$	$[M+2H]^{2+}$	$[M+H+NH_4]^{2+}$
m/z calcd for $C_{137}H_{199}N_{37}O_{34}$	727.6	969.8	975.5	1454.3	1462.8
found	728.3	970.6	976.2	1454.9	1463.3

Ac-PPG-PPG-PPG-PPG-PPG-PPG-PPG-PPG-PPG-PPG-PPG-NH2

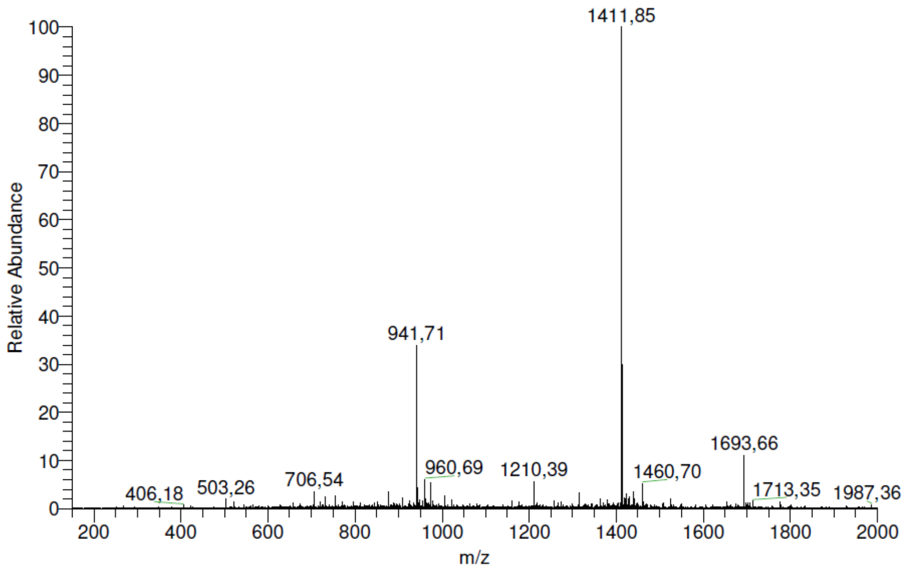

MS (ESI):	$[M+4H]^{4+}$	$[M+3H]^{3+}$	$[M+2H]^{2+}$	$[3M+5H]^{5+}$
m/z calcd for $C_{134}H_{192}N_{34}O_{34}$	706.4	941.5	1411.7	1693.8
found	706.5	941.7	1411.8	1693.6

Ac-PPG-PPG-PPG-PPG-PPG-PPG-PPG-[H2ProM2]G-PPG-PPG-PPG-NH2

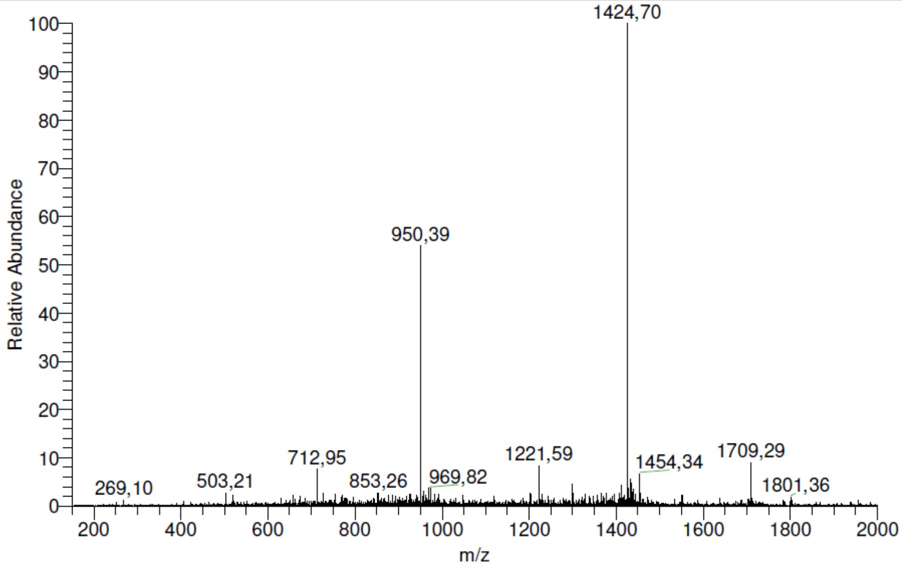

MS (ESI):	$[M+4H]^{4+}$	$[M+3H]^{3+}$	$[3M+7H]^{7+}$	$[M+2H]^{2+}$	$[3M+5H]^{5+}$
m/z calcd for $C_{136}H_{194}N_{34}O_{34}$	712.9	950.2	1221.3	1424.7	1709.5
found	712.9	950.3	1221.5	1424.7	1709.2

Biotin-Ebes-PPG-PPG-PPG-PPG-PPG-PRG-PPG-PPG-PPG-PPG-PPG-NH_2

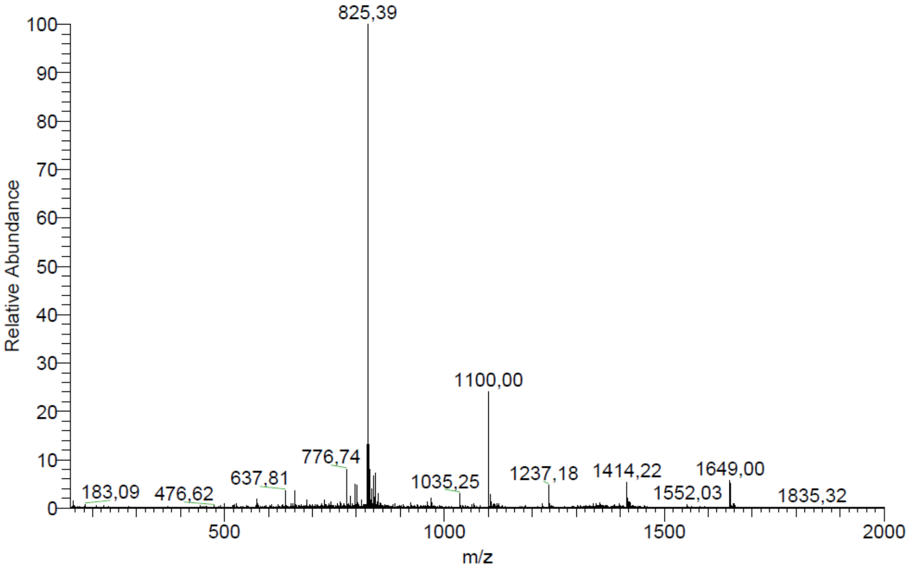

MS (ESI):	$[M+4H]^{4+}$	$[M+3H]^{3+}$	$[3M+7H]^{7+}$	$[M+2H]^{2+}$
m/z calcd for $C_{153}H_{227}N_{41}O_{39}S$	824.7	1099.2	1413.0	1648.4
found	825.4	1100.0	1414.2	1649.0

Biotin-Ebes-PPG-PPG-PPG-PPG-PPG-PRG-PPG-[H_2-ProM1]G-PPG-PPG-PPG-NH_2

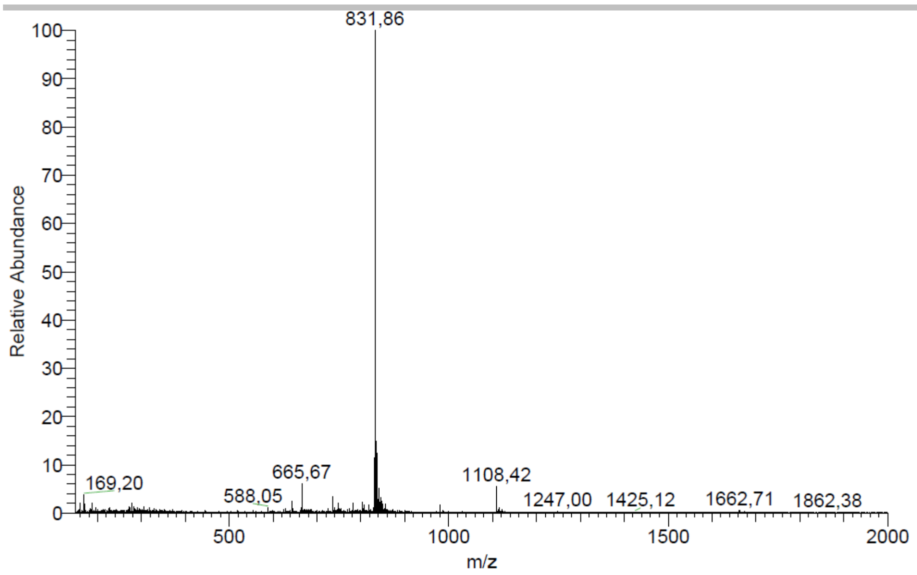

MS (ESI):	$[M+5H]^{5+}$	$[M+4H]^{4+}$	$[M+3H]^{3+}$
m/z calcd for $C_{155}H_{229}N_{41}O_{39}S$	665.2	831.2	1107.9
found	665.6	831.8	1108.4

Biotin-Ebes-PPG-PPG-PPG-PPG-PPG-PRG-PPG-[ProM2]G-PPG-PPG-PPG-NH_2

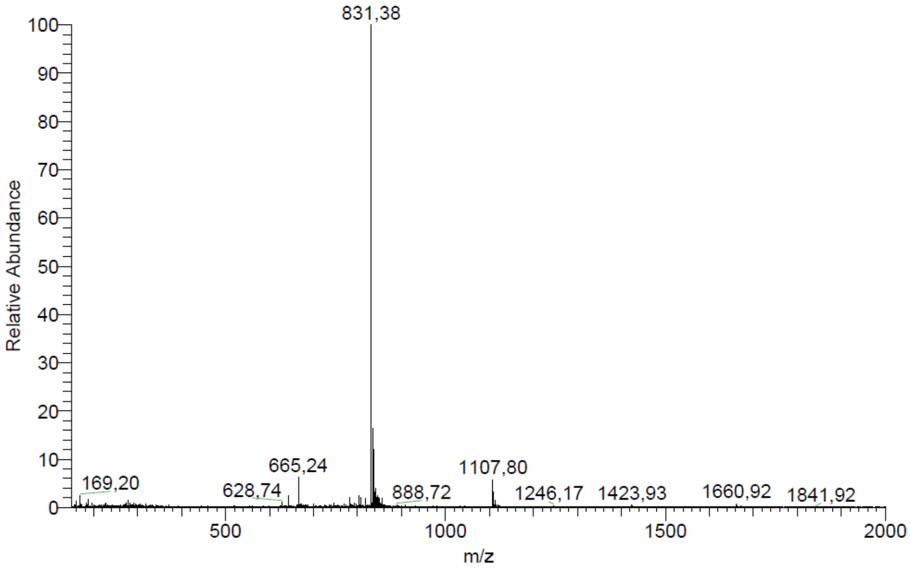

MS (ESI):	$[M+5H]^{5+}$	$[M+4H]^{4+}$	$[M+3H]^{3+}$
m/z calcd for $C_{155}H_{227}N_{41}O_{39}S$	664.7	830.7	1107.2
found	665.2	831.3	1107.8

Biotin-Ebes-PPG-PPG-PPG-PPG-PPG-PRG-PPG-[H_2-ProM2]G-PPG-PPG-PPG-NH_2

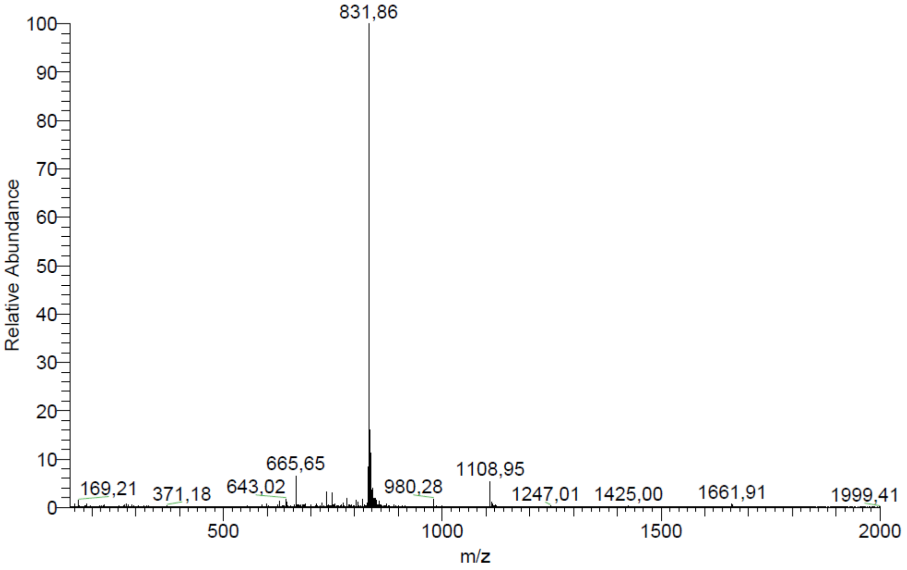

MS (ESI):	$[M+5H]^{5+}$	$[M+4H]^{4+}$	$[M+3H]^{3+}$
m/z calcd for $C_{155}H_{229}N_{41}O_{39}S$	665.1	831.1	1107.9
found	665.6	831.8	1108.9

Biotin-Ebes-PPG-PPG-PPG-PPG-PPG-PRG-PPG-[(HO)$_2$-ProM1]G-PPG-PPG-PPG-NH$_2$

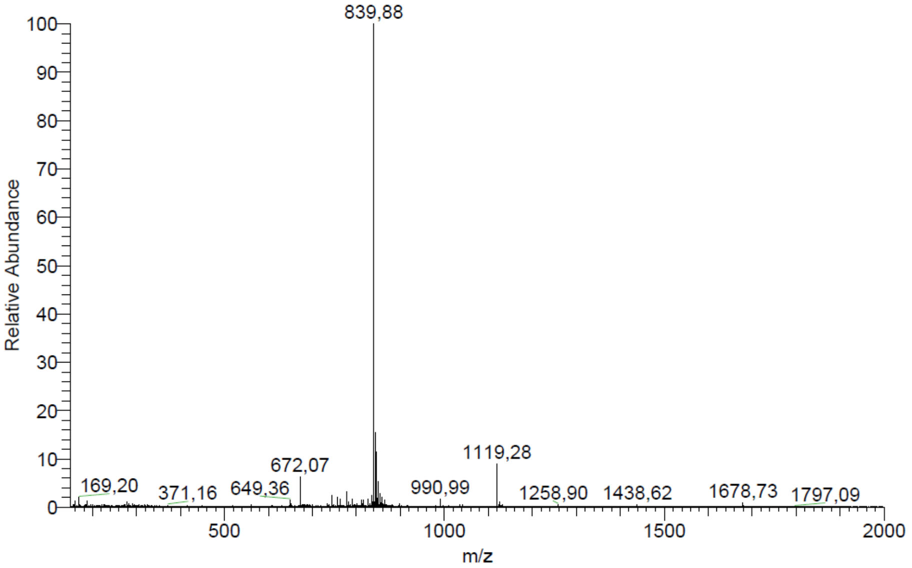

MS (ESI):	$[M+5H]^{5+}$	$[M+4H]^{4+}$	$[M+3H]^{3+}$
m/z calcd for $C_{155}H_{229}N_{41}O_{41}S$	671.5	839.2	1118.6
found	672.0	839.8	1119.3

Optimized Structures and Cartesian Coordinates

ProM1 exo/endo

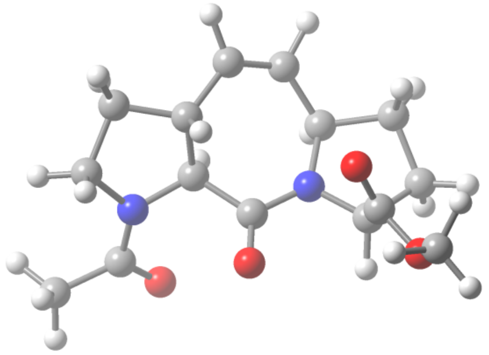

N	0.8214207	0.2392209	-0.7605703
C	0.9101634	1.6824769	-1.1063256
C	0.3551379	2.6871452	-0.1161275
C	-0.5664608	2.5345577	0.8396722
C	-1.3316194	1.2744566	1.1300346
C	-1.5082736	0.3849000	-0.1218455
C	-0.2519040	-0.4551359	-0.2994373
N	-2.7101419	-0.3883369	0.1526286
C	-3.3814128	0.0573478	1.3867057
C	-2.7789138	1.4492471	1.6220660
C	2.4341739	1.8817068	-1.2843628
C	2.9435217	0.5135893	-1.7439771
C	2.0907172	-0.4630542	-0.9087345
C	-3.2186838	-1.2063225	-0.8085143
C	-4.5112057	-1.9301979	-0.4752050
O	-2.6520887	-1.3403522	-1.8982277
O	-0.1730628	-1.6400520	0.0266800
C	2.7214753	-0.7277110	0.4585962
O	2.4870845	-0.1001423	1.4715953
O	3.6181248	-1.7228611	0.3746199
C	4.3423366	-2.0220654	1.5872846
H	-0.7936075	0.6874050	1.8904150
H	-1.6633425	1.0051058	-1.0134689
H	0.4175245	1.8358008	-2.0787800
H	0.8007599	3.6734534	-0.2409406
H	-0.7984495	3.3899984	1.4722608
H	-3.1490662	-0.6218096	2.2174261
H	-4.4652548	0.0843049	1.2583813
H	-2.8370796	1.7622146	2.6681361
H	-3.2980211	2.1934046	1.0064873
H	2.8757093	2.1405847	-0.3156965
H	2.6531613	2.6889944	-1.9875559
H	4.0155704	0.3712301	-1.5869434
H	2.7285139	0.3523975	-2.8053150
H	1.9435568	-1.4229821	-1.4075420
H	-4.4813515	-2.3823582	0.5213034
H	-5.3587254	-1.2346003	-0.4993206
H	-4.6762877	-2.7055961	-1.2248068
H	4.9067376	-1.1462930	1.9175278
H	3.6497580	-2.3267084	2.3759415
H	5.0161673	-2.8391179	1.3317642

ProM1 exo/exo

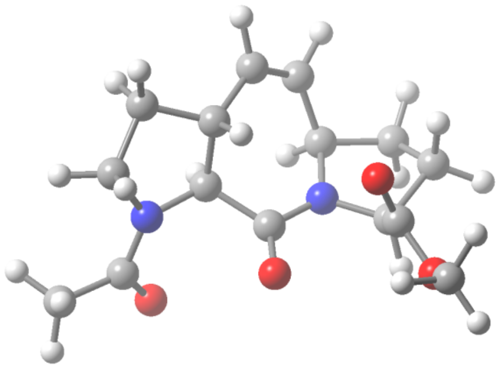

N	0.9106148	0.5000179	-0.7961499
C	0.8908436	1.9785340	-0.9270352
C	0.5345468	2.7234355	0.3516387
C	-0.3309831	2.3378033	1.2956941
C	-1.1214723	1.0562411	1.3042114
C	-1.3986913	0.4777836	-0.1060754
C	-0.1546338	-0.2982994	-0.5237732
N	-2.5885748	-0.3396782	0.0710858
C	-3.1729680	-0.1799410	1.4148516
C	-2.5308416	1.1208258	1.9171047
C	2.3214249	2.2643601	-1.4301616
C	3.1682211	1.1762281	-0.7600846
C	2.2596322	-0.0690151	-0.8332655
C	-3.1616694	-0.9400189	-1.0069088
C	-4.4318839	-1.7314123	-0.7510060
O	-2.6676852	-0.8334893	-2.1342967
O	-0.0634496	-1.5248930	-0.4701841
C	2.4977682	-0.9844981	0.3650825
O	2.1687612	-0.7216710	1.5047918
O	3.1921021	-2.0746486	0.0097861
C	3.5311307	-2.9764893	1.0843645
H	-0.5502293	0.3009937	1.8668154
H	-1.6071129	1.2810342	-0.8221604
H	0.1718716	2.2533642	-1.7086959
H	1.0275197	3.6876580	0.4654596
H	-0.4752826	2.9835975	2.1608418
H	-2.9023970	-1.0294783	2.0554675
H	-4.2621076	-0.1199965	1.3659471
H	-2.5162949	1.1884842	3.0083820
H	-3.0745510	1.9869810	1.5218802
H	2.6531326	3.2765794	-1.1853926
H	2.3531725	2.1501600	-2.5187251
H	3.3428517	1.4306697	0.2910257
H	4.1326157	1.0088680	-1.2443075
H	2.4198381	-0.6355715	-1.7557815
H	-4.3353613	-2.3875843	0.1200071
H	-5.2753455	-1.0554720	-0.5657016
H	-4.6520225	-2.3285369	-1.6372508
H	4.0931813	-3.7863362	0.6205027
H	2.6218253	-3.3591424	1.5549209
H	4.1400455	-2.4642591	1.8336148

H_2-ProM1 exo/endo

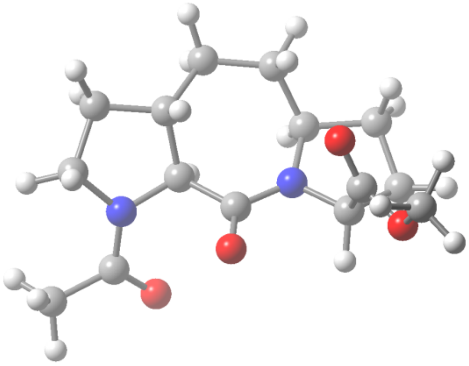

N	-0.8115203	0.1361555	0.7002508
C	-0.9362789	1.5658155	1.0951239
C	-0.6343244	2.5641281	-0.0371357
C	0.8391363	2.7279178	-0.4373293
C	1.4813616	1.4361205	-0.9390884
C	1.5538133	0.3478916	0.1618983
C	0.2762033	-0.4916627	0.1793771
N	2.7410506	-0.4308213	-0.1652669
C	3.4351497	0.0966905	-1.3499710
C	2.9396440	1.5465494	-1.4096892
C	-2.4137615	1.6659257	1.5421223
C	-2.8381970	0.2297068	1.8712006
C	-2.0577098	-0.6026225	0.8340625
C	3.1963784	-1.3686236	0.7076243
C	4.4630470	-2.1103478	0.3165929
O	2.6069948	-1.5934954	1.7704243
O	0.2178205	-1.6391637	-0.2654239
C	-2.8003069	-0.6845244	-0.4996254
O	-2.6222076	0.0491169	-1.4517437
O	-3.7205493	-1.6609733	-0.4621811
C	-4.5417332	-1.7997377	-1.6417104
H	0.8828206	1.0399311	-1.7719395
H	1.6853291	0.8063968	1.1506910
H	-0.2699471	1.7542344	1.9474923
H	-1.0171831	3.5402767	0.2872781
H	-1.2271600	2.2565722	-0.9070041
H	1.4185159	3.1041206	0.4181070
H	0.9063963	3.4916599	-1.2220727
H	3.1414811	-0.4614688	-2.2491861
H	4.5184822	0.0220109	-1.2385991
H	3.0290359	1.9832300	-2.4084117
H	3.5148694	2.1648117	-0.7092149
H	-3.0152434	2.0487503	0.7098264
H	-2.5324963	2.3531140	2.3835396
H	-3.9176469	0.0692208	1.8115066
H	-2.5033017	-0.0599255	2.8724088
H	-1.8616202	-1.6213323	1.1741428
H	4.5916990	-2.9548900	0.9952644
H	4.4215125	-2.4724511	-0.7157429
H	5.3377044	-1.4546910	0.4038635
H	-3.9202525	-2.0333998	-2.5098287
H	-5.2252558	-2.6205564	-1.4273796
H	-5.0954519	-0.8761941	-1.8289485

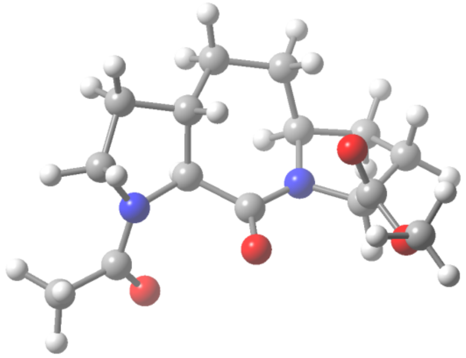

N	0.9044005	0.4327008	-0.8120859
C	0.9591078	1.9128247	-0.8974864
C	0.8265227	2.5862294	0.4822139
C	-0.6036195	2.5872170	1.0524282
C	-1.2252459	1.1968749	1.2035528
C	-1.4340250	0.4683560	-0.1532108
C	-0.1711085	-0.3268016	-0.4808010
N	-2.6040933	-0.3730199	0.0549368
C	-3.1649236	-0.2120261	1.4048208
C	-2.6276711	1.1600594	1.8296121
C	2.3253617	2.1250794	-1.5733445
C	3.2020335	0.9962728	-1.0109427
C	2.2250955	-0.1968718	-0.8802847
C	-3.1510886	-1.0450252	-0.9924583
C	-4.3872928	-1.8777739	-0.7003619
O	-2.6648758	-0.9675390	-2.1262046
O	-0.0905619	-1.5504863	-0.3540046
C	2.5131209	-0.9872845	0.3934637
O	2.2529020	-0.5966853	1.5151421
O	3.1656635	-2.1261052	0.1228726
C	3.5499765	-2.9146401	1.2690557
H	-0.5580268	0.5754725	1.8173741
H	-1.6418108	1.1830574	-0.9583692
H	0.1555662	2.2656812	-1.5529854
H	1.1639509	3.6258576	0.3896868
H	1.5046675	2.0811133	1.1803982
H	-1.2535869	3.1943584	0.4057736
H	-0.5918407	3.0785383	2.0333020
H	-2.8000509	-1.0055369	2.0708909
H	-4.2556664	-0.2542298	1.3860920
H	-2.6081440	1.2878262	2.9156626
H	-3.2522209	1.9530143	1.3994852
H	2.7357625	3.1188680	-1.3757318
H	2.2160079	2.0114913	-2.6574227
H	3.5821944	1.2702850	-0.0217984
H	4.0550430	0.7505527	-1.6469671
H	2.2825691	-0.8718432	-1.7387679
H	-4.5972460	-2.5007370	-1.5711628
H	-4.2510625	-2.5129574	0.1809517
H	-5.2537301	-1.2321493	-0.5135193
H	4.2063798	-2.3375491	1.9253188
H	2.6625582	-3.2266427	1.8256753
H	4.0740528	-3.7806531	0.8661718

H_2-ProM1 exo/exo

(HO)-ProM1 exo/endo

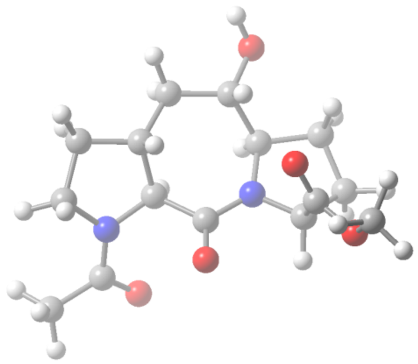

N	-0.7552903	-0.0612345	0.6715417
C	-0.9598555	1.3645671	1.0265037
C	-0.7205625	2.3378898	-0.1479820
C	0.7440688	2.5416038	-0.5588566
C	1.4591611	1.2711116	-1.0063962
C	1.6023543	0.2442432	0.1439260
C	0.3725989	-0.6590921	0.1973437
N	2.8312678	-0.4771289	-0.1561325
C	3.4907083	0.0394300	-1.3656067
C	2.9064108	1.4520063	-1.4893880
C	-2.4407974	1.3987647	1.4761297
C	-2.7799846	-0.0487009	1.8494622
C	-1.9589631	-0.8652144	0.8336598
C	3.3425617	-1.3490363	0.7542060
C	4.6503197	-2.0299262	0.3897080
O	2.7680830	-1.5627301	1.8271018
O	0.3756687	-1.8271251	-0.1912856
C	-2.6969018	-1.0321155	-0.4942672
O	-2.5508512	-0.3300696	-1.4753488
O	-3.5708662	-2.0471959	-0.4161224
C	-4.3836887	-2.2733462	-1.5880762
H	-0.2987202	1.6350794	1.8599812
H	1.7087379	0.7547114	1.1098810
H	0.8857571	0.7995518	-1.8169554
O	-1.2634179	3.5858090	0.2990349
H	-1.2929398	1.9465915	-1.0022804
H	1.2819667	2.9913201	0.2867270
H	0.7649740	3.2741908	-1.3769473
H	3.2326474	-0.5758432	-2.2379594
H	4.5765599	0.0383648	-1.2540134
H	2.9653771	1.8466018	-2.5075362
H	3.4429518	2.1364133	-0.8208420
H	-3.0626355	1.7255530	0.6368788
H	-2.5956465	2.1034727	2.2947226
H	-2.4258128	-0.2902222	2.8567979
H	-3.8490315	-0.2704921	1.8001763
H	-1.7010219	-1.8594261	1.2029132
H	4.8297637	-2.8362788	1.1023910
H	4.6287321	-2.4363147	-0.6266221
H	5.4846407	-1.3205469	0.4462966
H	-3.7507415	-2.5087606	-2.4473397
H	-4.9834880	-1.3870398	-1.8098458
H	-5.0246488	-3.1187569	-1.3405370
H	-1.1827764	4.2177587	-0.4329881

(HO)-ProM1 exo/exo

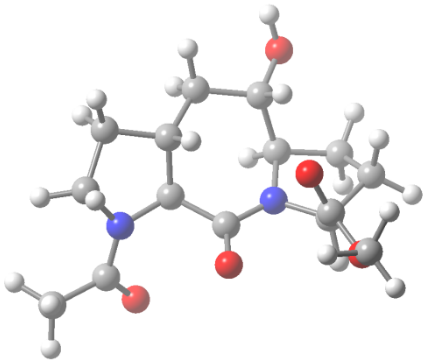

N	0.8494518	0.1381175	-0.8350620
C	1.0392456	1.5957285	-0.9911004
C	0.9965903	2.3228727	0.3732680
C	-0.4255795	2.4554840	0.9467057
C	-1.1752716	1.1388651	1.1483832
C	-1.4754961	0.3971539	-0.1826506
C	-0.2933817	-0.5158891	-0.4969047
N	-2.7117713	-0.3276096	0.0704524
C	-3.2366326	-0.0679317	1.4198517
C	-2.5645441	1.2593637	1.7931036
C	2.4068240	1.6545651	-1.6924718
C	3.1850450	0.4777623	-1.0869955
C	2.1057501	-0.6134847	-0.8903665
C	-3.3314162	-0.9852308	-0.9458215
C	-4.6319797	-1.6934439	-0.6091477
O	-2.8559461	-0.9913986	-2.0863678
O	-0.3222569	-1.7379940	-0.3472506
C	2.3363100	-1.3718604	0.4135181
O	2.0938101	-0.9259005	1.5185732
O	2.9166706	-2.5581343	0.1883359
C	3.2488825	-3.3269422	1.3642690
H	0.2583778	2.0098876	-1.6370756
H	-1.6301989	1.1049426	-1.0057554
H	-0.5637269	0.4726686	1.7725794
O	1.5427350	3.6254395	0.1490191
H	1.6224893	1.7528494	1.0759847
H	-1.0006103	3.1079258	0.2753349
H	-0.3547953	2.9743371	1.9122741
H	-2.9419217	-0.8704894	2.1092078
H	-4.3264354	-0.0039783	1.4131479
H	-2.5156191	1.4181258	2.8739722
H	-3.1160787	2.0949451	1.3448259
H	2.8999791	2.6156063	-1.5444820
H	2.2618518	1.4985827	-2.7668502
H	3.6044125	0.7620683	-0.1164367
H	4.0025856	0.1258786	-1.7195870
H	2.0864335	-1.3268041	-1.7187986
H	-4.5394145	-2.3068356	0.2930297
H	-5.4347589	-0.9668979	-0.4356827
H	-4.9095992	-2.3252590	-1.4541654
H	2.3425083	-3.5640022	1.9271612
H	3.9374735	-2.7674017	2.0023577
H	3.7201492	-4.2371049	0.9950009
H	1.5269573	4.0992274	0.9959031

(HO)$_2$-ProM1 exo/endo

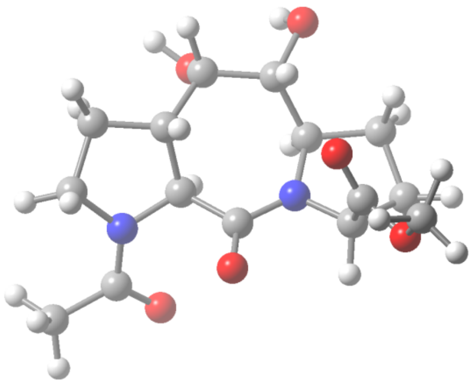

N	-0.8244921	-0.1697612	0.6701337
C	-0.9367800	1.2778064	0.9773184
C	-0.6817557	2.2009763	-0.2411618
C	0.7801333	2.3116206	-0.7048509
C	1.4182589	0.9781607	-1.0928478
C	1.5313780	0.0033932	0.1038220
C	0.2652274	-0.8422950	0.2098278
N	2.7236290	-0.7843859	-0.1762980
C	3.3714650	-0.3764452	-1.4311058
C	2.8598093	1.0564949	-1.6198664
C	-2.4003731	1.4193310	1.4603884
C	-2.8294150	0.0035250	1.8653249
C	-2.0774758	-0.8862676	0.8567005
C	3.2111515	-1.6295104	0.7709102
C	4.4811109	-2.3869808	0.4245007
O	2.6456151	-1.7600603	1.8618689
O	0.2105707	-2.0240443	-0.1309237
C	-2.8379767	-1.0192311	-0.4624397
O	-2.6403573	-0.3521353	-1.4591314
O	-3.7932199	-1.9549176	-0.3583640
C	-4.6315970	-2.1347807	-1.5205897
O	1.4849166	2.9458668	0.3764012
O	-1.1433953	3.5145231	0.0651279
H	-0.2383576	1.5372599	1.7814661
H	-1.2916660	1.8241272	-1.0682620
H	0.7792254	2.9735853	-1.5804778
H	0.7896426	0.5195041	-1.8679723
H	1.6769368	0.5607810	1.0350966
H	3.0533898	-1.0244450	-2.2588438
H	4.4586176	-0.4302920	-1.3505690
H	3.4495438	1.7389760	-0.9972829
H	2.9136407	1.3942226	-2.6583426
H	-3.0207299	1.7781890	0.6329998
H	-2.4880340	2.1427761	2.2727231
H	-3.9112979	-0.1478436	1.8333890
H	-2.4783215	-0.2419926	2.8726738
H	-1.8861855	-1.8901117	1.2401317
H	4.6412096	-3.1560681	1.1815733
H	4.4209168	-2.8527366	-0.5644516
H	5.3454774	-1.7121569	0.4220451
H	-5.3403083	-2.9170756	-1.2513500
H	-5.1551778	-1.2050615	-1.7571193
H	-4.0276613	-2.4408800	-2.3784120
H	2.1846321	3.5034542	0.0054394
H	-0.4214554	3.9161736	0.5832793

(HO)$_2$-ProM1 exo/exo

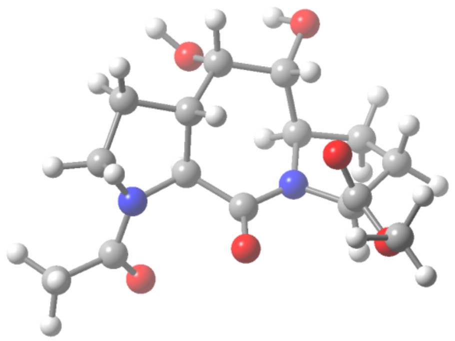

N	0.9000606	0.0349482	-0.8668829
C	0.9706750	1.5042409	-1.0117410
C	0.8917936	2.2242033	0.3628598
C	-0.5285956	2.2432719	0.9640275
C	-1.1682973	0.8649687	1.1502982
C	-1.4251686	0.1211993	-0.1872481
C	-0.1846049	-0.7042470	-0.5149055
N	-2.6070793	-0.6893919	0.0658677
C	-3.1210653	-0.5055394	1.4305671
C	-2.5507901	0.8631275	1.8214398
C	2.3238413	1.6818074	-1.7160236
C	3.2008154	0.5676762	-1.1243607
C	2.2150518	-0.6079335	-0.9111692
C	-3.1903956	-1.3715273	-0.9553737
C	-4.4344116	-2.1748293	-0.6189331
O	-2.7275636	-1.3246611	-2.1002845
O	-0.1195874	-1.9246792	-0.3612936
C	2.5040932	-1.3185110	0.4080809
O	2.2153025	-0.8722519	1.5024328
O	3.1862383	-2.4536140	0.2115204
C	3.5767345	-3.1659228	1.4053898
O	-1.3044915	3.0722381	0.0808218
O	1.3417834	3.5702253	0.2545764
H	0.1544241	1.8600937	-1.6464780
H	1.5621881	1.7108296	1.0593705
H	-0.4470936	2.7288695	1.9452465
H	-0.4821351	0.2616811	1.7596004
H	-1.6364983	0.8320699	-0.9915923
H	-2.7463699	-1.2960806	2.0946113
H	-4.2122619	-0.5298605	1.4481193
H	-3.1770466	1.6546783	1.3940474
H	-2.4958920	1.0063746	2.9039830
H	2.1915510	1.5285454	-2.7923106
H	2.7318005	2.6805158	-1.5546646
H	4.0333559	0.2816713	-1.7703612
H	3.6134980	0.8835833	-0.1607287
H	2.2571092	-1.3353365	-1.7261352
H	-5.2814245	-1.5094125	-0.4136491
H	-4.2851525	-2.8039189	0.2646881
H	-4.6822278	-2.8021652	-1.4764755
H	4.2115339	-2.5356586	2.0332242
H	2.6907977	-3.4673530	1.9699370
H	4.1260584	-4.0400179	1.0575947
H	-1.9698052	3.5443681	0.6027923
H	0.5812121	4.0576611	-0.1133515

(MeO)2-ProM1 exo/endo

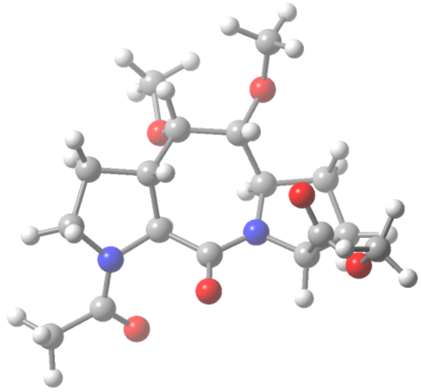

N	-0.8029864	-0.5491937	0.7089307
C	-0.9279129	0.8894555	1.0574543
C	-0.6870732	1.8326030	-0.1425624
C	0.7897519	1.9938211	-0.5583102
C	1.4047758	0.6805908	-1.0445787
C	1.5433067	-0.3582216	0.0965010
C	0.2766177	-1.2024985	0.1964258
N	2.7245862	-1.1336254	-0.2568968
C	3.3526248	-0.6499458	-1.4957588
C	2.8328970	0.7888978	-1.5972210
C	-2.3889335	0.9995389	1.5557323
C	-2.7907308	-0.4291649	1.9395479
C	-2.0452276	-1.2847141	0.8981651
O	0.2097368	-2.3709889	-0.1854936
C	3.2354938	-2.0260032	0.6329577
C	4.4953474	-2.7644776	0.2151566
O	2.6987574	-2.2141354	1.7300940
C	-2.8260501	-1.3953893	-0.4111392
O	-2.6588046	-0.6970286	-1.3918692
O	-3.7624128	-2.3511313	-0.3171090
C	-4.6178297	-2.5136438	-1.4693707
H	-0.2216837	1.1390428	1.8564188
H	0.7550962	0.2653369	-1.8260325
H	1.7149972	0.1453997	1.0530778
O	1.5774388	2.4725729	0.5294676
O	-1.2137943	3.1027038	0.2460789
C	1.7517507	3.8844583	0.5806128
C	-1.7285622	3.8726366	-0.8316494
H	-1.2577104	1.4262877	-0.9902809
H	0.8285719	2.7151324	-1.3874640
H	3.0271065	-1.2531035	-2.3539776
H	4.4411388	-0.7027802	-1.4328162
H	2.8614645	1.1776040	-2.6189457
H	3.4219859	1.4478039	-0.9508587
H	-3.0226756	1.3600654	0.7395178
H	-2.4802722	1.7092947	2.3794125
H	-3.8708683	-0.5949418	1.9236483
H	-2.4177564	-0.6910053	2.9348982
H	-1.8358286	-2.2950833	1.2537548
H	5.3617072	-2.0924869	0.2344373
H	4.6691419	-3.5772080	0.9219843
H	4.4131401	-3.1708612	-0.7980589
H	-5.3071515	-3.3165451	-1.2106455
H	-5.1625312	-1.5876057	-1.6700242
H	-4.0237125	-2.7836857	-2.3459584
H	2.4346368	4.0834342	1.4112372
H	2.2036756	4.2624721	-0.3484143
H	0.8028368	4.4013152	0.7550212
H	-0.9566955	4.1196055	-1.5740681
H	-2.5495002	3.3464666	-1.3401782
H	-2.1091578	4.8021801	-0.4010500

(MeO)2-ProM1 exo/exo

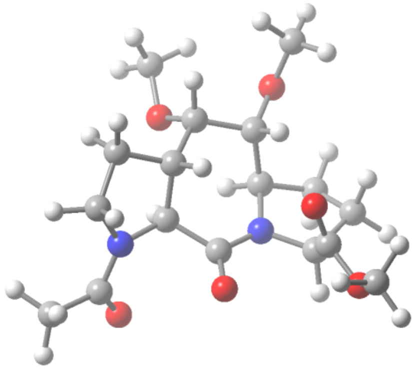

N	-0.8095620	-0.4768534	0.9357531
C	-1.0057454	0.9640612	1.2077002
C	-1.0271217	1.7790537	-0.1101870
C	0.3945612	2.0179794	-0.6764277
C	1.1206409	0.7163193	-1.0327165
C	1.4813867	-0.1374739	0.2144148
C	0.3220240	-1.0881092	0.4939397
N	2.7124784	-0.8205207	-0.1546143
C	3.1673848	-0.4512908	-1.5033292
C	2.4732310	0.8959957	-1.7362572
C	-2.3423663	0.9532149	1.9639307
C	-3.1391419	-0.1736146	1.2906912
C	-2.0629708	-1.2349527	0.9537597
O	0.3520671	-2.2923810	0.2373630
C	3.3859437	-1.5467426	0.7764275
C	4.6792694	-2.2037862	0.3268954
O	2.9627958	-1.6524271	1.9328788
C	-2.3323212	-1.8581021	-0.4128514
O	-2.1134290	-1.3047771	-1.4739687
O	-2.9170348	-3.0568130	-0.2936458
C	-3.2892093	-3.6994371	-1.5320534
H	-0.2011162	1.3440464	1.8415061
H	0.4595893	0.1251313	-1.6794560
H	1.6666818	0.4994597	1.0838458
O	1.2032107	2.7241795	0.2614166
O	-1.6594426	3.0271397	0.1632060
C	1.2126850	4.1406375	0.1176150
C	-2.3082260	3.6095191	-0.9596473
H	-1.6096408	1.2125915	-0.8516005
H	0.2963761	2.6104765	-1.5976421
H	2.8415619	-1.1998122	-2.2381681
H	4.2559269	-0.3796866	-1.5463487
H	2.3673074	1.1377471	-2.7975111
H	3.0340483	1.6978525	-1.2447566
H	-2.8436303	1.9200228	1.9165010
H	-2.1533891	0.7076634	3.0144808
H	-3.6015293	0.1918149	0.3678451
H	-3.9273098	-0.5870363	1.9232924
H	-2.0175796	-2.0280858	1.7046783
H	5.0020701	-2.8979062	1.1042771
H	4.5549846	-2.7429871	-0.6178212
H	5.4640577	-1.4521672	0.1796538
H	-2.4014983	-3.8814194	-2.1430066
H	-3.7566009	-4.6403597	-1.2440325
H	-3.9917528	-3.0737137	-2.0882103
H	1.9423199	4.5211051	0.8378208
H	1.5274091	4.4341466	-0.8947315
H	0.2300421	4.5737874	0.3288062
H	-1.6035287	3.8640338	-1.7634696
H	-3.0778334	2.9374809	-1.3663988
H	-2.7828629	4.5284351	-0.6069529

(EtO)$_2$-ProM1 exo/endo

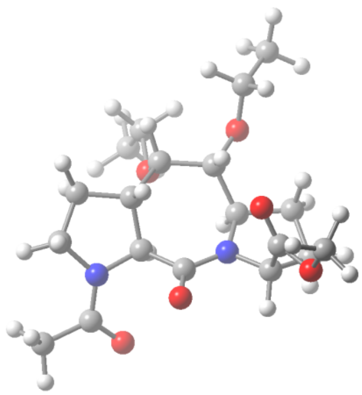

N	-0.7818460	-0.8962652	0.7251517
C	-0.9133949	0.5576645	0.9989054
C	-0.7365712	1.4373133	-0.2585367
C	0.7171174	1.5915166	-0.7491138
C	1.3341442	0.2622972	-1.1868904
C	1.5330881	-0.7078179	0.0044909
C	0.2844004	-1.5616703	0.2006618
N	2.7126243	-1.4856451	-0.3498009
C	3.2869452	-1.0621381	-1.6360316
C	2.7390772	0.3607636	-1.7975098
C	-2.3542070	0.6779868	1.5505072
C	-2.7208963	-0.7324292	2.0263451
C	-2.0047134	-1.6353019	1.0042106
C	3.2734243	-2.3158646	0.5698838
C	4.5308400	-3.0557833	0.1472165
O	2.7819722	-2.4490832	1.6959956
O	0.2182160	-2.7488258	-0.1191606
C	-2.8355170	-1.8279914	-0.2642018
O	-2.7221381	-1.1788387	-1.2856297
O	-3.7483898	-2.7938110	-0.0822815
C	-4.6469596	-3.0310712	-1.1877118
H	-0.1787012	0.8606284	1.7525012
H	1.7334652	-0.1470710	0.9229484
H	0.6627746	-0.2060945	-1.9184799
O	1.5358763	2.1443054	0.2778643
O	-1.2517698	2.7233130	0.0872698
C	1.7506830	3.5560973	0.2063216
C	-1.8918632	3.4122181	-0.9865159
C	2.6685699	3.9385271	1.3540612
C	-2.3448379	4.7654961	-0.4673147
H	-1.3414724	0.9832845	-1.0563130
H	0.7061993	2.2659828	-1.6171785
H	2.9409126	-1.7171925	-2.4468603
H	4.3777744	-1.0949360	-1.6109465
H	2.7244974	0.6925958	-2.8393871
H	3.3395582	1.0632853	-1.2099709
H	-3.0254956	0.9867635	0.7429358
H	-2.4216031	1.4301260	2.3380435
H	-3.7983964	-0.9117876	2.0625592
H	-2.3058985	-0.9351647	3.0188672
H	-1.7670266	-2.6217026	1.4062873
H	4.4202338	-3.5189319	-0.8385269
H	5.3847099	-2.3694197	0.0981350
H	4.7447865	-3.8250669	0.8907218
H	-5.2179849	-2.1266721	-1.4123336
H	-4.0842690	-3.3357579	-2.0736256
H	-5.3089060	-3.8316217	-0.8594498
H	0.7926552	4.0837026	0.2762930
H	2.2110507	3.8112306	-0.7611589
H	-2.7496091	2.8217223	-1.3447564
H	-1.2007197	3.5368278	-1.8337769
H	2.8706387	5.0148205	1.3370188
H	2.2055087	3.6869819	2.3142999
H	3.6214384	3.4033660	1.2795055
H	-2.8664439	5.3196451	-1.2547824
H	-3.0259031	4.6401831	0.3810666
H	-1.4853323	5.3582878	-0.1362209

(EtO)$_2$-ProM1 exo/exo

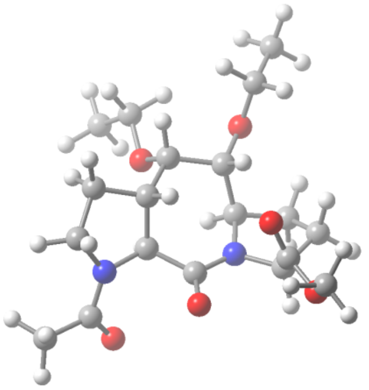

N	-0.4438785	1.0318147	0.9548016
C	0.9496649	0.5958397	1.1929701
C	1.6859657	0.3326102	-0.1446403
C	1.2987767	-1.0288274	-0.7717539
C	-0.1908072	-1.1326330	-1.1156997
C	-1.1015463	-1.1572500	0.1426030
C	-1.4763617	0.2800630	0.4876530
N	-2.2408589	-1.9751272	-0.2469627
C	-2.1080786	-2.4921568	-1.6172184
C	-0.5969816	-2.4097558	-1.8643221
C	1.5081005	1.7799659	1.9953859
C	0.8118827	3.0032759	1.3817580
C	-0.6063662	2.4855744	1.0371398
C	-3.1725943	-2.3204725	0.6806594
C	-4.3122306	-3.2063201	0.2082367
O	-3.0819674	-1.9355358	1.8516643
O	-2.5841848	0.7679334	0.2597611
C	-1.0737302	3.0492148	-0.3016687
O	-0.6702720	2.6678905	-1.3842027
O	-1.9215201	4.0713857	-0.1299422
C	-2.3659507	4.7274880	-1.3369876
H	0.9655067	-0.3210772	1.7867269
H	-0.5884135	-1.6284860	0.9856141
H	-0.4619963	-0.2606212	-1.7247969
O	1.6189947	-2.0950250	0.1173683
O	3.0854503	0.3575756	0.1200541
C	2.8765161	-2.7375626	-0.1079896
C	3.8904963	0.7903824	-0.9771048
C	2.9972420	-3.8718311	0.8945211
C	5.3405904	0.7702260	-0.5267898
H	1.4151853	1.1349980	-0.8461782
H	1.8680808	-1.1424881	-1.7052284
H	-2.6624036	-1.8591808	-2.3232305
H	-2.4946498	-3.5103317	-1.6931600
H	-0.3449482	-2.3736877	-2.9278543
H	-0.0943842	-3.2702664	-1.4101083
H	2.5954839	1.8312729	1.9376048
H	1.2176715	1.6662062	3.0453365
H	1.3275357	3.3093745	0.4656051
H	0.7736354	3.8641839	2.0523268
H	-1.3374165	2.7439744	1.8077048
H	-4.7627712	-2.8312932	-0.7164716
H	-3.9554867	-4.2249745	0.0143119

H	-5.0682360	-3.2445604	0.9939311
H	-1.5118899	5.1317696	-1.8860701
H	-2.9054359	4.0205456	-1.9722731
H	-3.0261829	5.5290489	-1.0078193
H	3.6927317	-2.0166908	0.0143413
H	2.9137587	-3.1241488	-1.1385273
H	3.5864158	1.8044170	-1.2801621
H	3.7498312	0.1300761	-1.8459126
H	3.9449977	-4.4028117	0.7555056
H	2.9637220	-3.4832511	1.9180549
H	2.1756732	-4.5853625	0.7689283
H	5.9938645	1.1166325	-1.3345375
H	5.4809864	1.4239891	0.3405372
H	5.6427638	-0.2448501	-0.2472084

ProM2 endo/exo

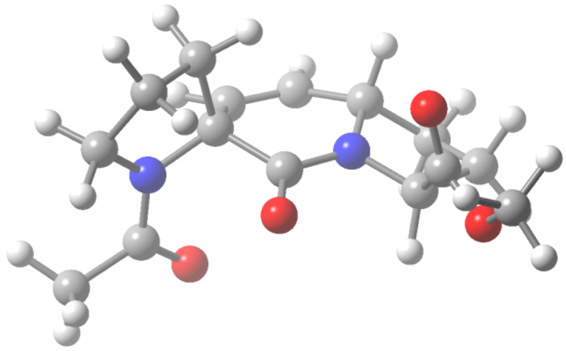

C	0.1862733	-0.1710176	0.0490401
N	-0.8497763	0.6868168	-0.0774514
C	-0.8305650	2.0121515	-0.7159933
C	0.5643913	2.5404143	-0.8558978
C	1.6226763	1.7339381	-0.7995992
C	1.4926672	0.2348044	-0.6678497
C	1.5352330	-0.4569154	-2.0631782
C	2.1180566	-1.8417113	-1.7643399
C	3.1857829	-1.5425700	-0.7052647
N	2.6385368	-0.3807065	0.0184019
C	-1.7943849	2.8027331	0.1832481
C	-2.8871570	1.7637714	0.4870424
C	-2.0985765	0.4330758	0.6325315
H	-1.2865711	1.9258596	-1.7164036
C	-2.8500343	-0.7408062	0.0187457
C	2.9469568	-0.0290771	1.2972344
C	4.1068524	-0.7671233	1.9426225
O	2.3171780	0.8467614	1.8993426
O	0.0791806	-1.2367297	0.6622432
O	-3.6555607	-1.3113815	0.9274399
O	-2.7742833	-1.0824470	-1.1438678
C	-4.4830058	-2.3913645	0.4441163
H	0.6710763	3.6067731	-1.0354206
H	2.6349730	2.1109442	-0.9207272
H	0.5453916	-0.4794996	-2.5273743
H	2.2038722	0.1131408	-2.7183597
H	2.5349400	-2.3309613	-2.6485199
H	1.3453818	-2.4859189	-1.3353984
H	4.1478286	-1.2842574	-1.1664122
H	3.3464898	-2.3858465	-0.0292698
H	-2.1821259	3.6988934	-0.3082656
H	-1.2748781	3.1016255	1.1008754
H	-3.5806118	1.6975252	-0.3585968
H	-3.4667134	1.9853575	1.3851489
H	-1.8860150	0.2013116	1.6799356

H	3.8066554	-1.7875937	2.2095794
H	4.9720060	-0.8401674	1.2759890
H	4.3890451	-0.2371759	2.8535946
H	-5.1372716	-2.0388799	-0.3572680
H	-3.8590812	-3.2076713	0.0715059
H	-5.0688297	-2.7176746	1.3027217

ProM2 exo/exo

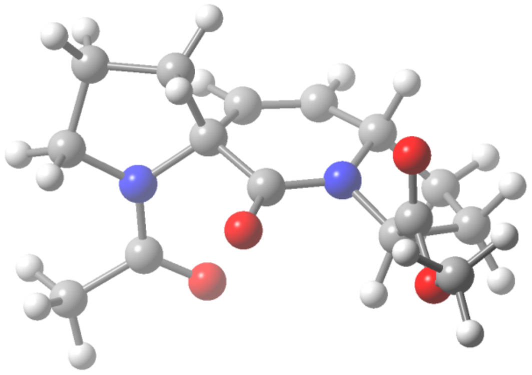

C	0.1377350	-0.3006224	-0.1213039
N	-0.8563601	0.6134815	-0.1546512
C	-0.7757038	2.0094652	-0.6061293
C	0.6351091	2.4714733	-0.7801827
C	1.6631142	1.6259609	-0.8034727
C	1.5117789	0.1265156	-0.6756929
C	1.7693027	-0.6038896	-2.0284203
C	3.2696402	-0.9200887	-2.0030969
C	3.5067089	-1.3049350	-0.5404800
N	2.5761643	-0.4292672	0.1871277
C	-1.6283259	2.7287309	0.4521622
C	-2.7842470	1.7357699	0.6623560
C	-2.0950953	0.3461013	0.5718742
H	-1.2964290	2.0903615	-1.5750854
C	-2.9613575	-0.6705329	-0.1575946
C	2.5669267	-0.2131576	1.5307150
C	3.6763430	-0.8751992	2.3291486
O	1.7047456	0.4895717	2.0691260
O	-0.0468615	-1.4426029	0.3076267
O	-3.7334599	-1.3423876	0.7106979
O	-2.9931840	-0.8188933	-1.3621945
C	-4.6601734	-2.2841702	0.1292490
H	0.7791966	3.5388580	-0.9267932
H	2.6793186	1.9844957	-0.9493793
H	1.1929787	-1.5352004	-2.0372844
H	1.4577935	0.0068612	-2.8787828
H	3.8558342	-0.0299885	-2.2572854
H	3.5473931	-1.7204193	-2.6940004
H	4.5374760	-1.1357945	-0.2197337
H	3.2574752	-2.3598848	-0.3620277
H	-1.9658559	3.7139725	0.1198554
H	-1.0423579	2.8458835	1.3709561
H	-3.5171093	1.8444472	-0.1447608
H	-3.3046316	1.8615563	1.6137245
H	-1.8531761	-0.0488152	1.5623730
H	4.6576272	-0.4806146	2.0417948
H	3.6954740	-1.9581280	2.1639333
H	3.5069654	-0.6730392	3.3876436
H	-5.3459419	-1.7721446	-0.5505680
H	-4.1177701	-3.0595286	-0.4176968
H	-5.2033481	-2.7164100	0.9689425

H_2-ProM2 endo/endo

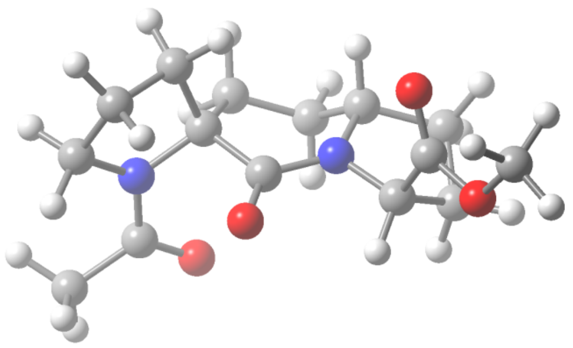

C	-0.3145117	-0.3097295	-0.1838354
N	0.8152630	0.3863558	0.0883698
C	0.9012516	1.5154346	1.0398950
C	-0.4150811	2.2823197	0.9838193
C	-1.5504761	1.3539029	1.4209902
C	-1.5291078	-0.0557808	0.7490721
C	-1.5451776	-1.1992945	1.8035513
C	-2.2616836	-2.3563973	1.1020452
C	-3.3586110	-1.6405816	0.3043438
N	-2.7558759	-0.3357010	-0.0217561
C	2.1675749	2.2840087	0.5881569
C	2.4906767	1.7460931	-0.8180174
C	1.9888778	0.2870550	-0.7624905
H	1.0465367	1.1202967	2.0534057
C	3.0296560	-0.6475735	-0.1483710
C	-3.0764068	0.4297616	-1.0971344
C	-4.3092753	0.0223744	-1.8851078
O	-2.4002835	1.4155762	-1.4184512
O	-0.3674924	-1.1588844	-1.0787705
O	4.0183745	-0.8842368	-1.0254257
O	2.9936877	-1.0935407	0.9807239
C	5.0991920	-1.7148427	-0.5491013
H	-0.3677709	3.1533487	1.6470536
H	-0.5843222	2.6330256	-0.0391344
H	-2.5189791	1.8178794	1.2222021
H	-1.4791171	1.2159366	2.5063771
H	-0.5362475	-1.4449800	2.1490216
H	-2.1251236	-0.8676033	2.6724680
H	-2.6697661	-3.0914355	1.8011594
H	-1.5787769	-2.8624579	0.4152457
H	-4.2655893	-1.4991926	0.9068838
H	-3.6335555	-2.1846501	-0.6022766
H	2.9945043	2.0713575	1.2728955
H	2.0025236	3.3650222	0.5922327
H	3.5497019	1.8161841	-1.0768809
H	1.9146329	2.2781639	-1.5815222
H	1.7096174	-0.1024997	-1.7429502
H	-4.0947259	-0.8638052	-2.4947637
H	-5.1550263	-0.2200599	-1.2339160
H	-4.5811984	0.8441539	-2.5493748
H	5.7978515	-1.7913532	-1.3815094
H	5.5802465	-1.2512787	0.3159092
H	4.7231961	-2.7024499	-0.2703296

H_2-ProM2 endo/exo

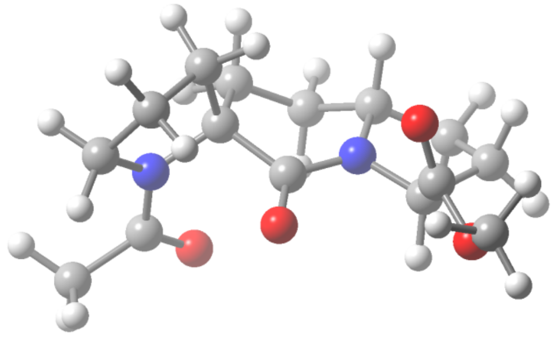

C	-0.1512774	-0.1407097	-0.0835565
N	0.8575170	0.7450997	0.0685676
C	0.7454517	2.0891162	0.6681582
C	-0.6816891	2.5863246	0.4934274
C	-1.6481879	1.6020850	1.1575888
C	-1.4351591	0.1126817	0.7442012
C	-1.3554853	-0.8185696	1.9892370
C	-1.9098482	-2.1602144	1.5039807
C	-3.0514279	-1.7354856	0.5733637
N	-2.5806967	-0.4580279	0.0081742
C	1.8508558	2.8584825	-0.0653089
C	2.9433903	1.7950180	-0.2659603
C	2.1359510	0.5157875	-0.5948660
H	0.9900710	2.0265093	1.7394901
C	2.8096963	-0.7397078	-0.0559165
C	-2.9423033	0.0441984	-1.2005443
C	-4.0845412	-0.6528130	-1.9195468
O	-2.3728147	1.0258145	-1.6945431
O	-0.0159269	-1.1620988	-0.7668419
O	3.5877094	-1.3008371	-0.9943881
O	2.7058588	-1.1486739	1.0829025
C	4.3387939	-2.4600716	-0.5756097
H	-0.7865639	3.5796000	0.9441741
H	-0.9041660	2.6615470	-0.5746467
H	-2.6831064	1.8765034	0.9382217
H	-1.5224788	1.6781251	2.2442321
H	-0.3356477	-0.8753510	2.3817378
H	-1.9971441	-0.4068157	2.7767269
H	-2.2564176	-2.7990141	2.3209948
H	-1.1491897	-2.6966821	0.9316783
H	-3.9861412	-1.5826434	1.1290926
H	-3.2395078	-2.4683609	-0.2144746
H	2.1994761	3.7263220	0.5009045
H	1.4747112	3.2060058	-1.0352709
H	3.5000386	1.6502321	0.6668722
H	3.6560880	2.0375628	-1.0568996
H	1.9895789	0.3988506	-1.6731306
H	-3.7463915	-1.6099691	-2.3346632
H	-4.9273616	-0.8599555	-1.2525470
H	-4.4147790	-0.0145302	-2.7405156
H	4.9135148	-2.7675083	-1.4486299
H	5.0040409	-2.2032426	0.2526934
H	3.6597027	-3.2570582	-0.2621577

H_2-ProM2 exo/endo

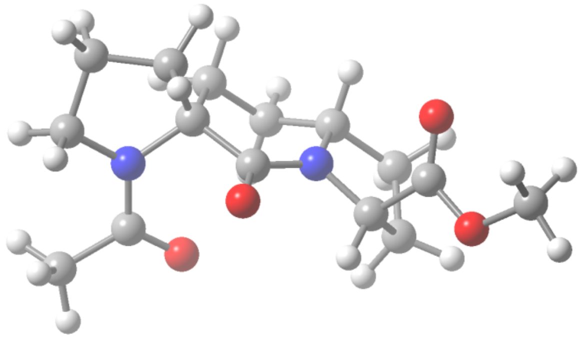

C	-0.2528774	-0.4937525	-0.2731001
N	0.8269165	0.1479291	0.2343050
C	0.8727004	0.9332094	1.4892794
C	-0.5020687	1.5217743	1.7766222
C	-1.5161630	0.3779796	1.8486967
C	-1.5520854	-0.5106960	0.5717521
C	-1.9011206	-1.9838870	0.9097509
C	-3.4328783	-1.9944033	0.9403044
C	-3.7881960	-1.1177490	-0.2635818
N	-2.6879948	-0.1376763	-0.3115942
C	2.0160605	1.9541204	1.2516513
C	2.3513274	1.8520565	-0.2480270
C	1.9981811	0.3887594	-0.5926465
H	1.1487622	0.2554627	2.3066887
C	3.1380385	-0.5598263	-0.2316457
C	-2.6461062	0.9377824	-1.1391246
C	-3.8570837	1.1492916	-2.0325533
O	-1.6808956	1.7122441	-1.1701934
O	-0.2202126	-1.0834411	-1.3571015
O	4.1103026	-0.4936639	-1.1557224
O	3.1881331	-1.2506490	0.7662240
C	5.2817110	-1.2971873	-0.8970845
H	-0.7711581	2.2274386	0.9867014
H	-0.4723714	2.0584204	2.7318028
H	-2.5246795	0.7600193	2.0317750
H	-1.2565530	-0.2542240	2.7073224
H	-1.4353451	-2.3118952	1.8419422
H	-1.5435765	-2.6239182	0.0968812
H	-3.8047377	-1.5406772	1.8656860
H	-3.8566594	-2.9988740	0.8591409
H	-4.7549784	-0.6190296	-0.1545882
H	-3.8092459	-1.7099811	-1.1881461
H	2.8875290	1.6849119	1.8560751
H	1.7134726	2.9654142	1.5378349
H	3.3895197	2.1012273	-0.4804401
H	1.6994627	2.5040508	-0.8375932
H	1.7471685	0.2502404	-1.6449603
H	-3.6546860	1.9952941	-2.6906810
H	-4.0711335	0.2614101	-2.6375211
H	-4.7518937	1.3653913	-1.4377011
H	5.9530251	-1.1138683	-1.7353070
H	5.7487597	-0.9944365	0.0435563
H	5.0116092	-2.3549003	-0.8448585

H_2-ProM2 exo/exo

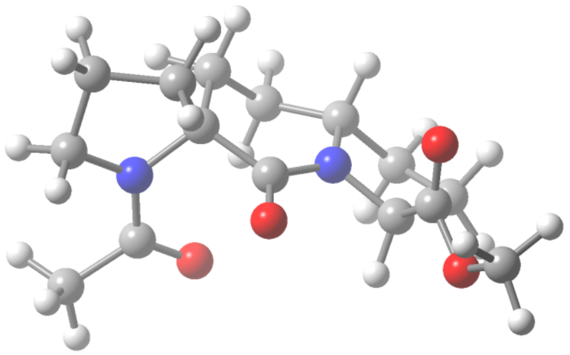

C	0.0771327	-0.3756616	-0.1025936
N	-0.8477678	0.5986355	-0.2483251
C	-0.6450010	1.9526067	-0.8086222
C	0.8308212	2.3230610	-0.8025747
C	1.6192872	1.1965780	-1.4760566
C	1.4629384	-0.1691665	-0.7571290
C	1.7499384	-1.3498651	-1.7179368
C	3.2749937	-1.4894900	-1.6712787
C	3.5811628	-1.2865820	-0.1840803
N	2.5180956	-0.3695899	0.2730564
C	-1.5881272	2.8000532	0.0515293
C	-2.7814988	1.8587008	0.2800817
C	-2.1096007	0.4784361	0.4786325
H	-1.0103872	1.9492423	-1.8471069
C	-2.9645948	-0.6539690	-0.0719735
C	2.4269570	0.1239826	1.5361578
C	3.5548516	-0.2435807	2.4861560
O	1.4851472	0.8367496	1.9057727
O	-0.1838834	-1.4247778	0.4965387
O	-3.7048329	-1.2080416	0.9017890
O	-3.0203427	-0.9762907	-1.2413934
C	-4.6165209	-2.2449671	0.4825715
H	1.1699672	2.4636654	0.2261301
H	0.9677851	3.2631072	-1.3485513
H	2.6840871	1.4414485	-1.5324540
H	1.2611306	1.0903331	-2.5082985
H	1.2792521	-2.2506694	-1.3121759
H	1.3520388	-1.1696459	-2.7194482
H	3.7543677	-0.7088933	-2.2717676
H	3.6270718	-2.4588756	-2.0338101
H	4.5720420	-0.8575990	-0.0108110
H	3.5195432	-2.2368533	0.3618357
H	-1.8702318	3.7362245	-0.4380250
H	-1.1006238	3.0379789	1.0046521
H	-3.4169631	1.8368502	-0.6124672
H	-3.4021140	2.1306718	1.1364690
H	-1.9053056	0.2798200	1.5352580
H	3.6551852	-1.3299103	2.5879289
H	4.5159357	0.1430424	2.1290545
H	3.3349725	0.1909243	3.4621923
H	-5.3253358	-1.8544869	-0.2521818
H	-4.0638278	-3.0803114	0.0449663
H	-5.1360277	-2.5613177	1.3865346

(HO)2-ProM2 endo/endo

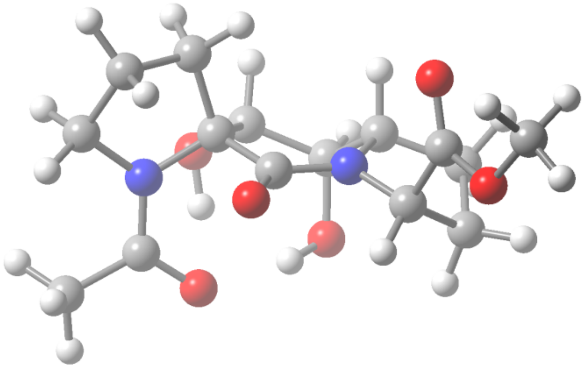

N	-0.9540390	0.2456875	-0.0508386
C	-0.9516485	1.4077902	-0.9557896
C	0.3911183	2.1272922	-0.8268494
C	1.5101980	1.1679712	-1.2735968
C	1.3689621	-0.3109733	-0.6924599
C	0.0969318	-0.5769633	0.1590835
C	1.3776022	-1.3505300	-1.8533873
C	2.0717265	-2.5800638	-1.2632226
C	3.1698020	-1.9514484	-0.4028717
N	2.5299038	-0.7264019	0.1115642
C	-2.1863813	2.2276106	-0.5123489
C	-2.5774440	1.6532446	0.8610771
C	-2.1571455	0.1713583	0.7605044
O	0.0517998	-1.5235963	0.9479924
C	2.8106489	-0.1414219	1.2914526
C	3.9813068	-0.6790664	2.0863301
O	2.1387339	0.8134583	1.7329062
C	-3.2227105	-0.6786607	0.0710138
O	-3.1788845	-1.0523764	-1.0838012
O	-4.2385605	-0.9256140	0.9116791
C	-5.3447641	-1.6748152	0.3628793
O	2.7881628	1.7286819	-1.0533854
O	0.6033202	2.6514826	0.4735528
H	-1.0708426	1.0686434	-1.9924773
H	0.3955767	2.9852549	-1.5082646
H	1.4173199	1.0767290	-2.3600480
H	0.3687305	-1.5470064	-2.2279984
H	1.9705503	-0.9503284	-2.6834358
H	1.3803429	-3.1382780	-0.6266218
H	2.4733845	-3.2497714	-2.0281821
H	4.0511639	-1.6888498	-1.0019748
H	3.4865538	-2.5991019	0.4159518
H	-2.9990081	2.0882128	-1.2313750
H	-1.9576623	3.2947925	-0.4615438
H	-3.6369105	1.7727193	1.0988563
H	-1.9828564	2.1221110	1.6497624
H	-1.9296497	-0.2730578	1.7310267
H	4.1965467	0.0193041	2.8958382
H	3.7307933	-1.6542672	2.5200027
H	4.8723084	-0.8079348	1.4645121
H	-6.0616230	-1.7757854	1.1769103
H	-5.7886909	-1.1335988	-0.4764443
H	-5.0055093	-2.6569732	0.0244111
H	2.7748800	2.0865960	-0.1483176
H	0.9377584	1.9213569	1.0418933

(HO)2-ProM2 endo/exo

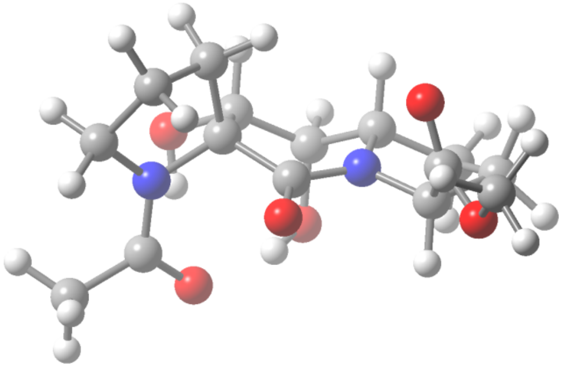

N	-0.9777763	0.6456964	-0.0462915
C	-0.7354552	1.9356755	-0.7094185
C	0.7210163	2.3560999	-0.4993860
C	1.6452730	1.2773354	-1.0968726
C	1.2385580	-0.2126814	-0.7015210
C	-0.0779868	-0.3475136	0.1024520
C	1.0604900	-1.0815658	-1.9853298
C	1.5285831	-2.4784834	-1.5720737
C	2.7177600	-2.1667415	-0.6618266
N	2.2940705	-0.9332220	0.0267739
C	-1.7962652	2.8342882	-0.0661920
C	-2.9789570	1.8777864	0.1580320
C	-2.2983317	0.5477181	0.5666010
O	-0.3251659	-1.3599570	0.7638094
C	2.6590240	-0.5766154	1.2728993
C	3.7065528	-1.4172391	1.9714079
O	2.1607949	0.4105401	1.8515762
C	-3.0522037	-0.6609243	0.0245128
O	-2.9526543	-1.0834187	-1.1098253
O	-3.8901029	-1.1535486	0.9478232
C	-4.7159244	-2.2579639	0.5199209
O	3.0030913	1.5641116	-0.8326273
O	1.0283159	2.6347502	0.8550082
H	-0.9225417	1.8391111	-1.7891234
H	0.8937020	3.2863460	-1.0518359
H	1.5377499	1.3458292	-2.1835939
H	0.0296182	-1.0500525	-2.3499237
H	1.7101388	-0.6875165	-2.7746987
H	0.7488081	-2.9854802	-0.9976573
H	1.8080003	-3.1029858	-2.4246748
H	3.6303625	-1.9758930	-1.2410082
H	2.9212578	-2.9632360	0.0551043
H	-2.0535381	3.6882543	-0.6980404
H	-1.4102284	3.2037625	0.8889869
H	-3.5285486	1.7351159	-0.7790102
H	-3.6821755	2.2207289	0.9196954
H	-2.2071455	0.4533669	1.6526603
H	4.5725330	-1.6052743	1.3295708
H	4.0249137	-0.8906212	2.8717399
H	3.2865400	-2.3875647	2.2608964
H	-4.0903939	-3.1058393	0.2295904
H	-5.3322158	-2.5129941	1.3812776
H	-5.3400626	-1.9598116	-0.3262226
H	3.0489875	1.8116312	0.1077045
H	1.2131470	1.7809303	1.3098362

$(HO)_2$-ProM2 exo/endo

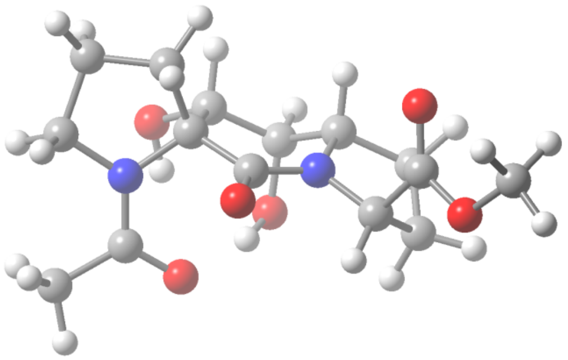

N	-0.9872483	0.1801804	-0.0696137
C	-0.9402495	1.3638651	-0.9450094
C	0.4286734	2.0254592	-0.8225824
C	1.5170492	1.0275529	-1.2544724
C	1.3488811	-0.4298501	-0.6275579
C	0.0115853	-0.7137919	0.1032906
C	1.5516203	-1.5104645	-1.7187006
C	3.0772226	-1.6271634	-1.8069025
C	3.5027905	-1.5647041	-0.3357972
N	2.4562601	-0.7356555	0.2917776
C	-2.1379727	2.2240724	-0.4750892
C	-2.5760561	1.6064381	0.8656930
C	-2.2103409	0.1141104	0.7110431
O	-0.1177174	-1.7272465	0.7911703
C	2.5208379	-0.2292246	1.5378122
C	3.7061356	-0.6326233	2.3881949
O	1.6480307	0.5397013	1.9895152
C	-3.2962698	-0.6597437	-0.0332835
O	-3.2656220	-0.9443856	-1.2136527
O	-4.3149572	-0.9455532	0.7913997
C	-5.4420415	-1.6215510	0.1922026
O	2.7993705	1.5724428	-1.0055082
O	0.6840384	2.5464096	0.4728977
H	-1.0810951	1.0514682	-1.9870542
H	0.4682473	2.8820878	-1.5038018
H	1.4400052	0.9104889	-2.3400600
H	1.1194024	-2.4526077	-1.3663142
H	1.0693690	-1.2390666	-2.6612852
H	3.4950935	-0.7754452	-2.3532434
H	3.4086963	-2.5470945	-2.2948577
H	4.4884413	-1.1118713	-0.2014960
H	3.5105891	-2.5616678	0.1211086
H	-2.9465786	2.1618459	-1.2088586
H	-1.8549420	3.2738431	-0.3687477
H	-3.6343567	1.7576216	1.0906427
H	-1.9810452	2.0181610	1.6858463
H	-2.0202830	-0.3781546	1.6661908
H	3.5394588	-0.2773881	3.4056820
H	3.8492371	-1.7178571	2.3962247
H	4.6258631	-0.1798647	1.9999549
H	-5.1301730	-2.5848149	-0.2190578
H	-6.1603473	-1.7630742	0.9988725
H	-5.8718970	-1.0077528	-0.6033392
H	2.7092495	2.0782948	-0.1765574
H	0.8376465	1.7859366	1.0853795

$(HO)_2$-ProM2 exo/exo

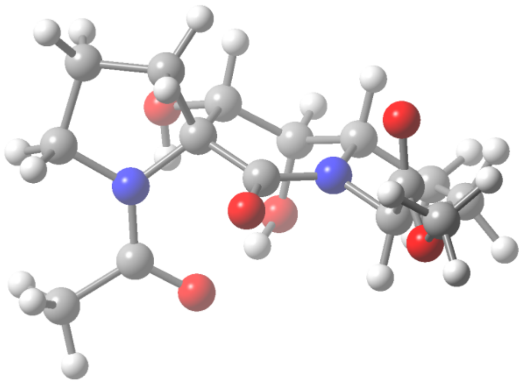

N	-1.0124597	0.5647141	-0.1014265
C	-0.7252403	1.9132949	-0.6193847
C	0.7539785	2.2537240	-0.4530431
C	1.6080099	1.1654867	-1.1232853
C	1.2348263	-0.3130032	-0.6822995
C	-0.1723799	-0.4906243	-0.0652711
C	1.3667749	-1.2788008	-1.8867745
C	2.8704768	-1.5685600	-1.9374537
C	3.2353812	-1.7157272	-0.4558452
N	2.2425180	-0.8715335	0.2406936
C	-1.7150158	2.7802474	0.1634085
C	-2.9439855	1.8674253	0.3009397
C	-2.3340249	0.4601824	0.5148192
O	-0.5190198	-1.5791588	0.4006191
C	2.2346900	-0.6515856	1.5706267
C	3.2808448	-1.3698330	2.3960382
O	1.4060009	0.1046525	2.1171831
C	-3.1685905	-0.6216120	-0.1598680
O	-3.1689207	-0.8439471	-1.3535793
O	-3.9544924	-1.2456131	0.7297449
C	-4.8448954	-2.2437173	0.1869708
O	2.9826535	1.4423166	-0.9259404
O	1.1569637	2.4665763	0.8895709
H	-0.9641474	1.9442693	-1.6924583
H	0.9493218	3.1964153	-0.9751212
H	1.4323696	1.2279914	-2.2019054
H	0.8100242	-2.1938711	-1.6615968
H	0.9650433	-0.8453267	-2.8061223
H	3.1113122	-2.4672878	-2.5108639
H	3.4090935	-0.7223586	-2.3730925
H	4.2505734	-1.3745447	-0.2370934
H	3.1354206	-2.7548182	-0.1214453
H	-1.9373839	3.7191243	-0.3502734
H	-1.2856577	3.0092511	1.1437709
H	-3.5230221	1.8771010	-0.6289564
H	-3.6076079	2.1465958	1.1219448
H	-2.2278606	0.2172403	1.5761211
H	3.1021956	-1.1468836	3.4483634
H	3.2395330	-2.4529631	2.2397275
H	4.2884015	-1.0354813	2.1246032
H	-4.2704387	-3.0428527	-0.2884259
H	-5.4060125	-2.6288546	1.0376911
H	-5.5182048	-1.7939473	-0.5471520
H	3.0470236	1.7780557	-0.0125201
H	1.0790312	1.6108856	1.3816814

(MeO)₂-ProM2 endo/endo

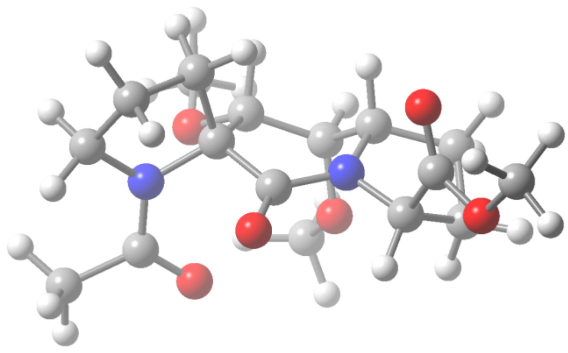

N	-1.1612224	0.1450663	-0.0231884
C	-0.9544301	1.3271708	-0.8793250
C	0.4483192	1.8754946	-0.6155064
C	1.4365628	0.7784962	-1.0804880
C	1.0960481	-0.6773636	-0.5692576
C	-0.2104107	-0.7772124	0.2572479
C	-2.1194632	2.2717039	-0.5024625
C	-2.6612600	1.7265407	0.8302979
C	-2.4051875	0.2076951	0.7228748
C	0.9202563	-1.6304527	-1.7956988
C	1.4124609	-2.9884625	-1.2958960
C	2.6168993	-2.5894361	-0.4417862
N	2.1842188	-1.3236636	0.1729661
C	2.5821514	-0.8766198	1.3953812
O	-0.4008676	-1.7067980	1.0437704
C	3.7256836	-1.6321615	2.0558102
O	2.0494941	0.0880449	1.9471529
O	2.8033875	1.0450860	-0.8333534
O	0.4848795	2.2532298	0.7426977
C	3.3758202	2.0127934	-1.7021684
C	1.6038485	3.0386876	1.1336088
H	-1.0264599	1.0385324	-1.9346462
C	-3.5251429	-0.5023624	-0.0356550
O	-3.4690577	-0.8580235	-1.1958535
O	-4.6039747	-0.6463996	0.7498868
C	-5.7593994	-1.2520833	0.1307848
H	0.6032150	2.7610038	-1.2542008
H	1.2879063	0.7565168	-2.1727798
H	-2.8916782	2.2282817	-1.2765482
H	-1.7802226	3.3066473	-0.4181342
H	-3.7140948	1.9623161	1.0022319
H	-2.0692011	2.1131445	1.6632089
H	-2.2851405	-0.2714606	1.6961557
H	-0.1111596	-1.6321542	-2.1609985
H	1.5643324	-1.2817322	-2.6107475
H	1.6781963	-3.6712216	-2.1076003
H	0.6541121	-3.4611539	-0.6652782
H	3.5106598	-2.4305437	-1.0612813
H	2.8555916	-3.3338700	0.3196263
H	3.3826483	-2.6034854	2.4318378
H	4.5538826	-1.8166428	1.3640688
H	4.0804853	-1.0396720	2.9005999
H	4.4463550	2.0323122	-1.4835074
H	3.2272488	1.7399463	-2.7577340
H	2.9653222	3.0178702	-1.5377281
H	1.3884180	3.4003834	2.1430711
H	2.5197148	2.4453698	1.1468208
H	1.7294476	3.9070826	0.4681191
H	-6.5223012	-1.2880200	0.9076659
H	-6.0979417	-0.6458687	-0.7133038
H	-5.5177387	-2.2590849	-0.2183394

(MeO)₂-ProM2 endo/exo

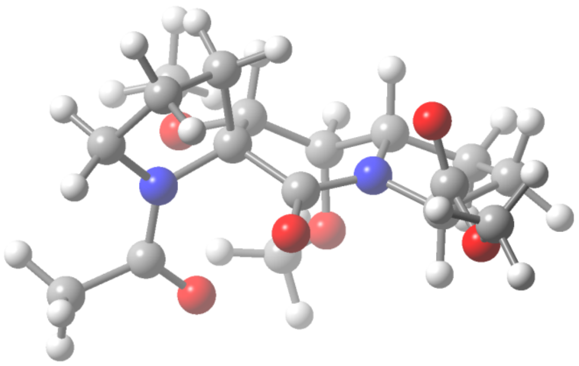

N	-1.1335430	0.6355488	-0.0930891
C	-0.6143677	1.8470265	-0.7490353
C	0.8696786	2.0026063	-0.4084265
C	1.5841610	0.7545872	-0.9838844
C	0.9102646	-0.6251855	-0.6131546
C	-0.4275488	-0.4913883	0.1467505
C	-1.5545825	2.9392513	-0.2254713
C	-2.8922712	2.2007343	-0.0570850
C	-2.4710398	0.8047337	0.4607431
C	0.5788105	-1.4075620	-1.9278676
C	0.7587400	-2.8780951	-1.5532388
C	1.9869193	-2.8197698	-0.6444965
N	1.7908621	-1.5616755	0.0941185
C	2.2081513	-1.3370970	1.3703988
O	-0.8913048	-1.4187087	0.8156775
C	3.1387360	-2.3784014	1.9748275
O	1.8548028	-0.3467748	2.0123842
O	2.9682255	0.6700402	-0.7065857
O	0.9590380	2.1987977	0.9835181
C	3.7735230	1.5598356	-1.4679923
C	2.2147915	2.6632362	1.4608470
H	-0.7183938	1.7531988	-1.8393190
C	-3.4039495	-0.2892243	-0.0448392
O	-3.3721937	-0.7583203	-1.1649531
O	-4.3094883	-0.6170293	0.8886437
C	-5.2916824	-1.5973406	0.4917041
H	1.2555282	2.8884013	-0.9399719
H	1.4591650	0.8705324	-2.0731656
H	-1.6211173	3.7878710	-0.9112172
H	-1.1845613	3.2933648	0.7407083
H	-3.3885850	2.0934651	-1.0280882
H	-3.5823790	2.6954593	0.6296460
H	-2.4407486	0.7697869	1.5542806
H	-0.4191248	-1.1587769	-2.3008130
H	1.3066688	-1.1306969	-2.6986388
H	0.9059180	-3.5250623	-2.4224430
H	-0.1044432	-3.2344400	-0.9836595
H	2.9158692	-2.7817185	-1.2306723
H	2.0459365	-3.6695809	0.0372765
H	3.5581498	-1.9646791	2.8933122
H	2.5885502	-3.2933112	2.2249890
H	3.9498193	-2.6532600	1.2928837
H	3.5927888	1.4383521	-2.5464374
H	3.6045562	2.6109839	-1.1991650
H	4.8128736	1.3049678	-1.2468116
H	2.0603523	2.9509562	2.5046315
H	2.9747957	1.8816351	1.4078846
H	2.5493364	3.5483186	0.8972870
H	-5.8632848	-1.2369824	-0.3673619
H	-4.8010726	-2.5390013	0.2324756
H	-5.9404713	-1.7286176	1.3570296

(MeO)$_2$-ProM2 exo/endo

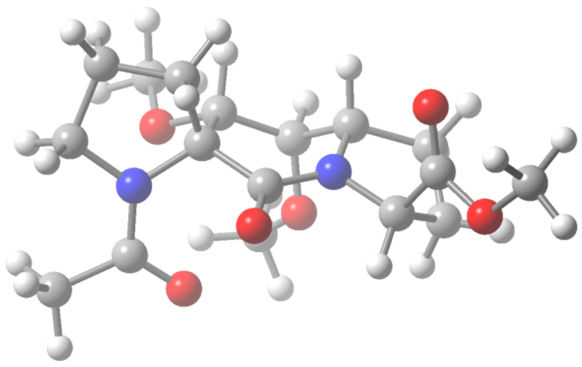

N	-1.2208101	0.0773096	-0.0434442
C	-0.9896559	1.2595375	-0.8938231
C	0.4124618	1.7977151	-0.6126962
C	1.4013258	0.6894667	-1.0399838
C	1.0532181	-0.7532894	-0.4908054
C	-0.2978045	-0.8708504	0.2461572
C	-2.1520156	2.2142057	-0.5332889
C	-2.7242501	1.6662586	0.7853087
C	-2.4826576	0.1449860	0.6724636
C	1.0242505	-1.7695004	-1.6651571
C	2.5093351	-2.0689805	-1.8887256
C	3.0563412	-2.1514499	-0.4573791
N	2.1401983	-1.3010726	0.3244632
C	2.3750589	-0.8882332	1.5994492
O	-0.5454648	-1.8345599	0.9721196
C	3.6091698	-1.4664247	2.2729972
O	1.6348996	-0.0942816	2.1843515
O	2.7602905	0.9661949	-0.7428582
O	0.4286966	2.1810663	0.7445116
C	3.3751971	1.8922593	-1.6290511
C	1.5553457	2.9338152	1.1746841
H	-1.0500711	0.9719820	-1.9499874
C	-3.5967287	-0.5383267	-0.1170996
O	-3.5460184	-0.8194189	-1.2978332
O	-4.6675545	-0.7444112	0.6658798
C	-5.8218870	-1.3206875	0.0174529
H	0.5873384	2.6781645	-1.2530921
H	1.2947136	0.6390118	-2.1357278
H	-2.9096399	2.1831179	-1.3219280
H	-1.8036382	3.2448520	-0.4352855
H	-3.7774621	1.9131038	0.9398295
H	-2.1435091	2.0400659	1.6320081
H	-2.3904913	-0.3431470	1.6439178
H	0.5008238	-2.6719810	-1.3318228
H	0.5084690	-1.3760036	-2.5449236
H	2.9877253	-1.2443180	-2.4271413
H	2.6800264	-2.9895745	-2.4528438
H	4.0850343	-1.7865862	-0.3888682
H	3.0288339	-3.1806777	-0.0785016
H	3.5871704	-1.1867886	3.3273599
H	4.5203280	-1.0605693	1.8175723
H	3.6548592	-2.5569700	2.1852680
H	4.4274469	1.9464049	-1.3401518
H	3.3037487	1.5507639	-2.6724615
H	2.9372237	2.8962046	-1.5558235
H	2.4418793	2.3046086	1.2756297
H	1.7680762	3.7628540	0.4823691
H	1.2910295	3.3521015	2.1501681
H	-5.5738149	-2.3014156	-0.3960593
H	-6.5788628	-1.4127898	0.7955156
H	-6.1719741	-0.6657992	-0.7845383

(MeO)$_2$-ProM2 exo/exo

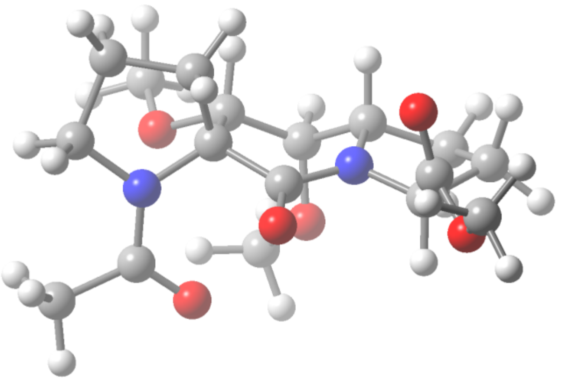

N	-1.2236060	0.5429484	-0.1408514
C	-0.7160592	1.7860759	-0.7482624
C	0.7496741	1.9743061	-0.3537260
C	1.5213439	0.7628660	-0.9245429
C	0.8856608	-0.6394746	-0.5612059
C	-0.4968323	-0.5692783	0.1126151
C	-1.7004249	2.8418431	-0.2307981
C	-3.0210974	2.0648387	-0.1158412
C	-2.5766137	0.6726403	0.3896372
C	0.7016183	-1.4803385	-1.8560376
C	2.1069186	-2.0381559	-2.0959255
C	2.5721858	-2.4013273	-0.6797712
N	1.8105339	-1.4880640	0.1929025
C	2.0630801	-1.3090600	1.5186448
O	-0.9728485	-1.5305861	0.7205237
C	3.1272555	-2.2118339	2.1227887
O	1.4692732	-0.4716531	2.2006262
O	2.8966915	0.7292000	-0.5822709
O	0.7809960	2.1193855	1.0479676
C	3.7050115	1.6406467	-1.3154439
C	2.0057916	2.5778715	1.6055041
H	-0.7815888	1.7164104	-1.8430217
C	-3.4797971	-0.4327460	-0.1431791
O	-3.4465736	-0.8614728	-1.2788969
O	-4.3664103	-0.8180213	0.7872778
C	-5.3242147	-1.8115483	0.3654800
H	1.1288434	2.8884275	-0.8394360
H	1.4418298	0.8721485	-2.0183193
H	-1.7698335	3.7019444	-0.9018017
H	-1.3684662	3.1862256	0.7525514
H	-3.4851039	1.9604173	-1.1029653
H	-3.7447290	2.5280214	0.5583444
H	-2.5621053	0.6242984	1.4830870
H	-0.0013283	-2.2938051	-1.6469835
H	0.3061494	-0.8875766	-2.6846732
H	2.7564126	-1.2647090	-2.5185350
H	2.1164084	-2.8985462	-2.7700173
H	3.6489872	-2.2595403	-0.5518479
H	2.3296280	-3.4430205	-0.4358227
H	2.9524866	-3.2677816	1.8912504
H	4.1194503	-1.9497144	1.7367883
H	3.1209113	-2.0713595	3.2046712
H	3.5968903	1.4844974	-2.3990331
H	3.4697531	2.6877474	-1.0844382
H	4.7390573	1.4409106	-1.0248968
H	1.7845975	2.8674500	2.6367068
H	2.7656956	1.7940553	1.6025723
H	2.3826487	3.4596444	1.0647443
H	-5.9111578	-1.4404241	-0.4785765
H	-4.8103956	-2.7311469	0.0739295
H	-5.9635675	-1.9875354	1.2299285

(EtO)$_2$-ProM2 endo/endo

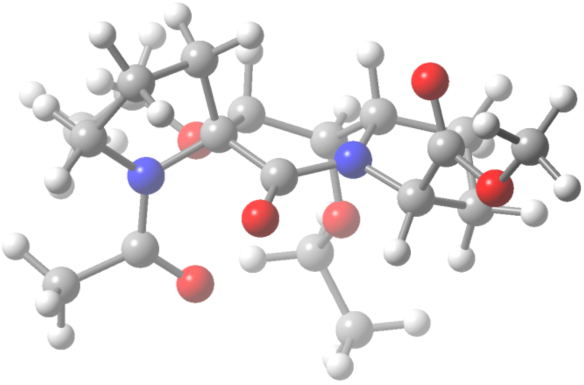

N	-1.4147047	0.0403873	-0.1401642
C	-1.1396766	0.9493340	-1.2679783
C	0.2724141	1.5107307	-1.0934126
C	1.2442342	0.3223860	-1.2271786
C	0.8113980	-0.9674830	-0.4056380
C	-0.4990457	-0.7965222	0.4033233
C	-2.2809868	1.9892921	-1.1974834
C	-2.8707106	1.8408741	0.2157945
C	-2.6657130	0.3431701	0.5311017
C	0.5682807	-2.1716247	-1.3689177
C	0.9405573	-3.3983962	-0.5341936
C	2.1611371	-2.9055414	0.2476093
N	1.8672593	-1.4775531	0.4752811
C	2.2868855	-0.7716283	1.5659980
O	-0.7281889	-1.4685487	1.4106270
C	3.4005135	-1.4025443	2.3879442
O	1.8021108	0.3170989	1.8738139
O	2.5623628	0.7478642	-0.9688590
O	0.2712793	2.2046498	0.1331379
C	3.5730077	0.0424499	-1.6906099
C	1.3781051	3.0701947	0.3940584
C	0.9687766	3.9893766	1.5327969
C	4.9198836	0.5596293	-1.2182926
C	-3.7984481	-0.5148799	-0.0299250
O	-3.7367747	-1.1809520	-1.0439726
O	-4.8943740	-0.4028609	0.7371924
C	-6.0596579	-1.1241190	0.2823165
H	-1.1855368	0.3953730	-2.2133811
H	0.4965113	2.2047297	-1.9196617
H	1.1719495	0.0425362	-2.2901333
H	-3.0361483	1.7568004	-1.9546026
H	-1.9099995	2.9994167	-1.3863605
H	-3.9182903	2.1432712	0.2852209
H	-2.2830341	2.4225467	0.9295308
H	-2.5768464	0.1449469	1.6009081
H	-0.4587255	-2.1871804	-1.7461514
H	1.2380604	-2.0851613	-2.2318273
H	1.1607080	-4.2787915	-1.1440019
H	0.1326629	-3.6417145	0.1614473
H	3.0849016	-3.0235831	-0.3351367
H	2.2885172	-3.4339365	1.1943372
H	3.0068125	-2.2098938	3.0171074
H	4.1914590	-1.8245715	1.7597598
H	3.8200636	-0.6327011	3.0377565
H	3.4395596	0.2100970	-2.7715155
H	3.4940532	-1.0367536	-1.5089526
H	1.6171135	3.6507607	-0.5114635
H	2.2540265	2.4768187	0.6629825
H	1.7864374	4.6739401	1.7846042
H	0.0886279	4.5816783	1.2587316
H	0.7246627	3.4002759	2.4235965
H	5.7310029	0.0682221	-1.7657444
H	4.9934748	1.6402797	-1.3802337
H	5.0494125	0.3601078	-0.1492454
H	-5.8480112	-2.1953525	0.2367755
H	-6.8368083	-0.9175412	1.0173270
H	-6.3615490	-0.7706607	-0.7068868

(EtO)$_2$-ProM2 endo/exo

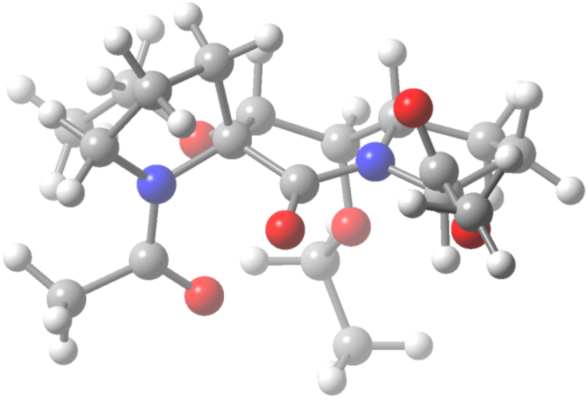

N	-1.4090012	0.4860293	-0.3763926
C	-0.8744699	1.4874111	-1.3156239
C	0.5936098	1.7468129	-0.9629437
C	1.3506800	0.4228386	-1.1910945
C	0.6458492	-0.8403579	-0.5336255
C	-0.6847324	-0.5109719	0.1805187
C	-1.8347167	2.6697795	-1.1333878
C	-3.1673779	1.9940639	-0.7722198
C	-2.7355292	0.8135452	0.1279967
C	0.2795716	-1.8901651	-1.6311147
C	0.3583672	-3.2327551	-0.9033401
C	1.5756396	-3.0420459	0.0046082
N	1.5184392	-1.6101395	0.3591361
C	1.9710869	-1.0985336	1.5419703
O	-1.1277123	-1.2059307	1.0982580
C	2.8884232	-1.9994475	2.3556807
O	1.6662506	0.0262082	1.9368383
O	2.7023946	0.5736282	-0.8229461
O	0.6051486	2.2507839	0.3522762
C	3.6185530	-0.2427114	-1.5544568
C	1.8050386	2.8914721	0.7898791
C	1.4878126	3.5833178	2.1041420
C	4.9994830	-0.0282083	-0.9614552
C	-3.6737820	-0.3788281	-0.0103258
O	-3.6568371	-1.1674759	-0.9339608
O	-4.5666163	-0.4036609	0.9906078
C	-5.5537446	-1.4544216	0.9233917
H	-0.9340723	1.1049286	-2.3441830
H	1.0136759	2.4877269	-1.6620709
H	1.3010300	0.2685175	-2.2809083
H	-1.9015281	3.2874059	-2.0327182
H	-1.4810306	3.2885567	-0.3039911
H	-3.6507134	1.6036678	-1.6749426
H	-3.8706830	2.6564031	-0.2629437
H	-2.6856599	1.1074838	1.1815902
H	-0.6992151	-1.6842486	-2.0740081
H	1.0256013	-1.8498628	-2.4325720
H	0.4716933	-4.0813280	-1.5833930
H	-0.5365379	-3.3841897	-0.2932274
H	1.5286743	-3.6674947	0.8980061
H	2.5101717	-3.2737115	-0.5244507
H	3.3896108	-1.3841670	3.1047146
H	3.6356006	-2.5023338	1.7335054

H 2.3099009 -2.7732598 2.8744435
H 3.5944919 0.0393922 -2.6193467
H 3.3341531 -1.2999861 -1.4836647
H 2.1322454 3.6200637 0.0297639
H 2.5947866 2.1501237 0.9205014
H 2.3742039 4.1022489 2.4857821
H 0.6829962 4.3158437 1.9762583
H 1.1712956 2.8438867 2.8471020
H 5.7451386 -0.6137343 -1.5093947
H 5.2788262 1.0294775 -1.0129719
H 5.0138975 -0.3395820 0.0884527
H -6.1371502 -1.3700536 0.0030289
H -5.0664817 -2.4322333 0.9561277
H -6.1905852 -1.3139605 1.7961212

$(EtO)_2$-ProM2 exo/endo

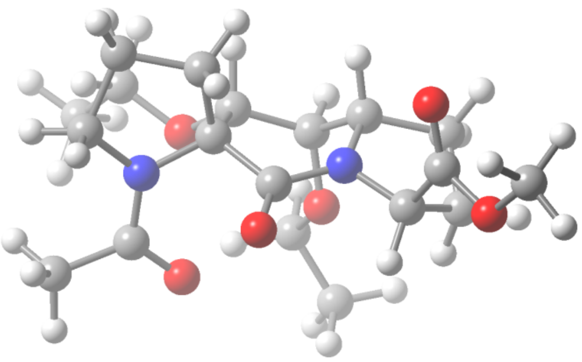

N -1.4490399 -0.0236148 -0.1385682
C -1.1558371 0.8317410 -1.3038775
C 0.2453341 1.4139414 -1.1198262
C 1.2502906 0.2453922 -1.1688085
C 0.7806078 -1.0187982 -0.3211563
C -0.5326600 -0.8163385 0.4686378
C -2.3058162 1.8643809 -1.3082353
C -2.9146599 1.7909251 0.1026542
C -2.7078736 0.3132883 0.5027420
C 0.5095890 -2.2514315 -1.2390417
C 1.8465477 -2.9970190 -1.2732862
C 2.3628524 -2.8257083 0.1581871
N 1.8466693 -1.5045336 0.5541570
C 2.2210083 -0.8195667 1.6733367
O -0.7750929 -1.4474129 1.4974552
C 3.3533906 -1.4323348 2.4818727
O 1.6871187 0.2383243 2.0091403
O 2.5216164 0.7412782 -0.8149387
O 0.2050453 2.1365716 0.0917026
C 3.6401754 0.1692770 -1.4876173
C 1.2725306 3.0468546 0.3662769
C 0.7486604 4.0630325 1.3682103
C 4.8718612 0.9564378 -1.0745638
C -3.8369031 -0.5704841 -0.0228848
O -3.7851250 -1.2487416 -1.0292128
O -4.9212775 -0.4605574 0.7614290
C -6.0866554 -1.1973367 0.3328388
H -1.1752311 0.2335111 -2.2225424
H 0.4814413 2.0892333 -1.9573036
H 1.2763218 -0.0763279 -2.2215086
H -3.0481920 1.5826717 -2.0611571
H -1.9402293 2.8655265 -1.5489789
H -3.9646321 2.0913340 0.1403864
H -2.3400521 2.4137946 0.7915306
H -2.6292490 0.1751868 1.5824518
H -0.2512280 -2.8852484 -0.7716294
H 0.1413777 -1.9572482 -2.2252281
H 2.5320210 -2.5287776 -1.9870023
H 1.7375430 -4.0485057 -1.5513458
H 3.4538793 -2.8581192 0.2185835
H 1.9602301 -3.6054888 0.8186772
H 3.1380340 -2.4680417 2.7663077
H 4.2855773 -1.4353952 1.9045304
H 3.4954507 -0.8325445 3.3819170
H 3.4870661 0.2187690 -2.5774211
H 3.7586273 -0.8864920 -1.2149993
H 1.5914847 3.5417520 -0.5637977
H 2.1261573 2.4980479 0.7697976
H 1.5356812 4.7768258 1.6363067
H -0.1007686 4.6171590 0.9537822
H 0.4167537 3.5572930 2.2818289
H 5.7624931 0.5588464 -1.5724105
H 4.7610419 2.0120859 -1.3436635
H 5.0195470 0.8886269 0.0085742
H -5.8659556 -2.2670658 0.2956016
H -6.8542473 -0.9898406 1.0775909
H -6.4072860 -0.8577446 -0.6553566

$(EtO)_2$-ProM2 exo/exo

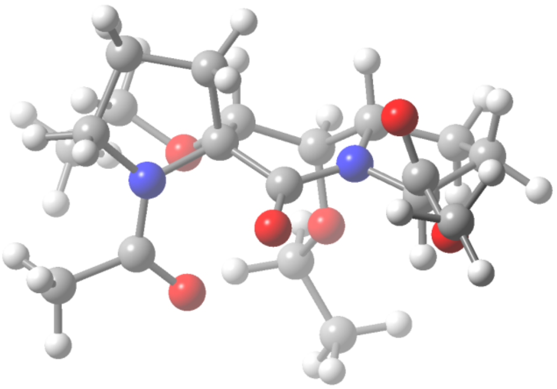

N -1.4508453 0.3960337 -0.4151580
C -0.9109208 1.3691029 -1.3814993
C 0.5342041 1.6820824 -0.9860472
C 1.3563872 0.3842465 -1.1270471
C 0.6252530 -0.8796866 -0.4906087
C -0.7148325 -0.5575944 0.2011223
C -1.9038525 2.5354270 -1.2847611
C -3.2270282 1.8502525 -0.9068393
C -2.7840074 0.7339834 0.0654508
C 0.2481188 -1.9274444 -1.5855769
C 1.4463048 -2.8790418 -1.6236547
C 1.8487026 -2.9725178 -0.1493174
N 1.5127599 -1.6415931 0.3864548
C 1.8791008 -1.1831010 1.6192189
O -1.1638175 -1.2342458 1.1276142
C 2.8128901 -2.0839123 2.4126290
O 1.4926070 -0.1032542 2.0669504
O 2.6463999 0.6230625 -0.6122171
O 0.4755000 2.2069718 0.3221576
C 3.7254631 -0.0378530 -1.2683260
C 1.6233465 2.9010994 0.8172361
C 1.1494755 3.8074801 1.9415923
C 5.0147183 0.4572074 -0.6361708
C -3.7060539 -0.4770948 -0.0003545
O -3.7024496 -1.2989462 -0.8946689
O -4.5700016 -0.4786146 1.0262801
C -5.5382510 -1.5486704 1.0277888

H	-0.9231769	0.9397207	-2.3928245
H	0.9583502	2.4248621	-1.6796366
H	1.4344594	0.1935671	-2.2086345
H	-1.9688827	3.0982180	-2.2194727
H	-1.5814823	3.2102048	-0.4867968
H	-3.6859290	1.3961223	-1.7922779
H	-3.9535276	2.5243340	-0.4478275
H	-2.7406954	1.0939190	1.0988356
H	-0.6473733	-2.4715433	-1.2675322
H	0.0269320	-1.4541197	-2.5456538
H	2.2606762	-2.4514108	-2.2174180
H	1.1978460	-3.8555265	-2.0474463
H	2.9106651	-3.1950435	-0.0167662
H	1.2693263	-3.7485014	0.3687553
H	2.4038474	-3.0932457	2.5307651
H	3.7832501	-2.1776327	1.9108108
H	2.9637434	-1.6380831	3.3967252
H	3.7016468	0.1873889	-2.3463989
H	3.6422475	-1.1253549	-1.1527949
H	2.0828822	3.4885375	0.0075377
H	2.3600445	2.1795870	1.1759534
H	1.9965296	4.3484006	2.3782535
H	0.4197466	4.5375866	1.5743843
H	0.6755658	3.2144745	2.7316120
H	5.8791410	-0.0165997	-1.1130292
H	5.1042371	1.5429545	-0.7466303
H	5.0301448	0.2151057	0.4319033
H	-5.0317411	-2.5156336	1.0838773
H	-6.1528954	-1.3861191	1.9125475
H	-6.1489980	-1.5108327	0.1221603

ProPro endo/endo

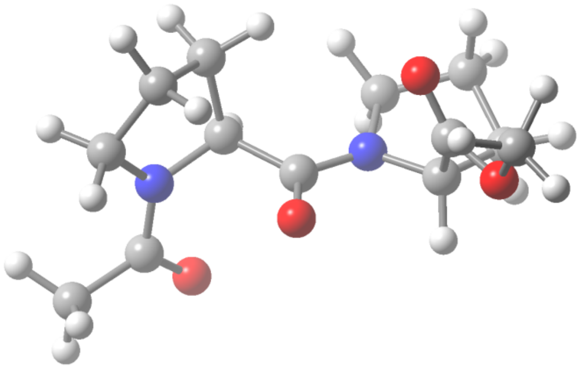

N	-2.7277153	-0.1196825	0.1447454
C	-1.5041047	0.4040984	0.7402085
C	-1.3358154	-0.4565486	2.0146988
C	-1.9443218	-1.8054285	1.5971070
C	-3.1458995	-1.4040243	0.7289713
C	-0.3058258	0.1823062	-0.1946037
O	-0.2869059	-0.7394658	-1.0162650
N	0.7638266	0.9950307	-0.0142888
C	2.0020164	0.7005125	-0.7278619
C	2.9198494	1.8843753	-0.3515458
C	2.4505112	2.2468753	1.0657843
C	0.9278510	2.0577752	0.9970120
C	2.5872052	-0.6249819	-0.2373157
O	3.3925948	-1.1589384	-1.1675018
O	2.3977368	-1.1055415	0.8626446
C	4.0688257	-2.3778543	-0.7905539
C	-3.2826877	0.5109254	-0.9213523
O	-2.7875393	1.5473604	-1.3815627
C	-4.5309274	-0.1222429	-1.5086468

H	-1.6259504	1.4672703	0.9577023
H	-1.9051288	-0.0072391	2.8353440
H	-0.2900940	-0.5396424	2.3237629
H	-2.2352670	-2.4266031	2.4480404
H	-1.2229620	-2.3603484	0.9897487
H	-4.0528404	-1.2699330	1.3329219
H	-3.3610281	-2.1401066	-0.0507178
H	1.8287749	0.6262437	-1.8031690
H	3.9797091	1.6237116	-0.4094599
H	2.7341738	2.7106174	-1.0457801
H	2.7285836	3.2607354	1.3638626
H	2.8751479	1.5441655	1.7899361
H	0.4206925	2.9724649	0.6661838
H	0.5072438	1.7590384	1.9599676
H	4.7030292	-2.2052772	0.0826780
H	3.3394570	-3.1588917	-0.5615236
H	4.6716568	-2.6569568	-1.6540155
H	-5.2721433	-0.3544725	-0.7366781
H	-4.9635107	0.5668598	-2.2354499
H	-4.2794757	-1.0611182	-2.0164818

ProPro endo/exo

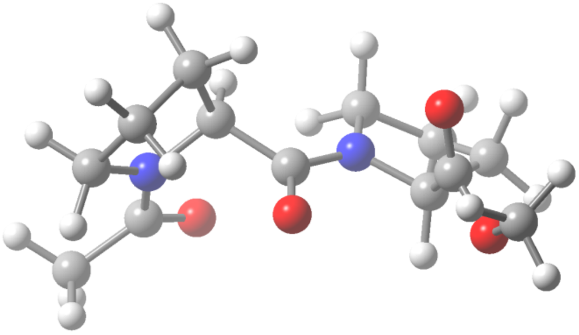

N	-2.6512668	-0.2176816	0.0244216
C	-1.4738097	-0.1609283	0.8964819
C	-1.5711543	-1.4728739	1.7081759
C	-2.3248496	-2.4298161	0.7700174
C	-3.3415367	-1.5163026	0.0720103
C	-0.1878859	-0.1410487	0.0590981
O	0.0307285	-1.0281952	-0.7765429
N	0.7291222	0.8181771	0.3206836
C	2.0152556	0.7728954	-0.3773083
C	2.7845474	1.9731689	0.2147578
C	1.6632595	2.9658110	0.5550667
C	0.5423335	2.0640042	1.0894658
C	2.7345278	-0.5437612	-0.1107679
O	3.4375824	-0.9303168	-1.1865090
O	2.7304076	-1.1313040	0.9521066
C	4.2205897	-2.1312245	-1.0228716
C	-2.9883709	0.8623135	-0.7223222
O	-2.3045056	1.8957737	-0.7004666
C	-4.2305760	0.7344094	-1.5841540
H	-1.5383016	0.7163224	1.5408337
H	-2.1526286	-1.2854974	2.6173398
H	-0.5877422	-1.8471555	2.0051790
H	-2.8074896	-3.2557898	1.2993901
H	-1.6310926	-2.8375904	0.0309325
H	-4.2710489	-1.4313323	0.6506445
H	-3.5961339	-1.8619521	-0.9335924
H	1.8695754	0.8815224	-1.4569835
H	3.3005909	1.6592073	1.1293263
H	3.5262488	2.3659201	-0.4842364

H	1.3261044	3.4784185	-0.3529424
H	1.9695814	3.7225949	1.2820124
H	-0.4523454	2.4701370	0.8994625
H	0.6609258	1.8734122	2.1631639
H	3.5687200	-2.9784313	-0.7946891
H	4.7267546	-2.2829769	-1.9756704
H	4.9473535	-2.0052506	-0.2162637
H	-5.0923608	0.3933325	-1.0004864
H	-4.4506649	1.7073344	-2.0256802
H	-4.0687191	0.0067641	-2.3880812

ProPro exo/endo

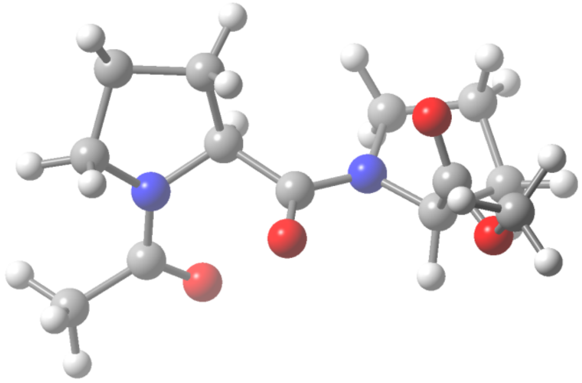

N	-2.7133046	-0.1538981	-0.0197162
C	-1.5070636	0.2225024	0.7174253
C	-1.4722054	-0.7720655	1.9052392
C	-2.9315176	-1.2338308	2.0456308
C	-3.4175940	-1.2865082	0.5927447
C	-0.2556272	0.0476640	-0.1501283
O	-0.1716674	-0.8711980	-0.9706031
N	0.7780616	0.8913346	0.0799881
C	2.0332602	0.6822195	-0.6344741
C	2.8859350	1.9017602	-0.2185872
C	2.3853393	2.2051323	1.2018988
C	0.8748340	1.9433438	1.1100889
C	2.6894069	-0.6241622	-0.1872664
O	3.4997303	-1.0978524	-1.1462682
O	2.5475576	-1.1370656	0.9048435
C	4.2426663	-2.2890977	-0.8099118
C	-3.0523103	0.5228279	-1.1457094
O	-2.3722020	1.4770538	-1.5443334
C	-4.3020639	0.0605205	-1.8729497
H	-1.6049229	1.2583835	1.0545775
H	-1.0718029	-0.3119108	2.8119338
H	-0.8332212	-1.6229014	1.6448279
H	-3.5140982	-0.4915976	2.6027970
H	-3.0259642	-2.1973017	2.5537607
H	-4.5001391	-1.1733714	0.5020524
H	-3.1249879	-2.2279989	0.1078725
H	1.8657728	0.6339510	-1.7122684
H	3.9583947	1.6974551	-0.2713760
H	2.6649304	2.7346779	-0.8942555
H	2.6096894	3.2237889	1.5277003
H	2.8366710	1.5059078	1.9130852
H	0.3279564	2.8379961	0.7877473
H	0.4572862	1.6048661	2.0614835
H	3.5578085	-3.1108928	-0.5860662
H	4.8422336	-2.5185119	-1.6901620
H	4.8840094	-2.1046342	0.0556676
H	-4.2661458	-1.0126271	-2.0892598
H	-5.1972559	0.2458020	-1.2678740
H	-4.3852187	0.6175242	-2.8072634

ProPro exo/exo

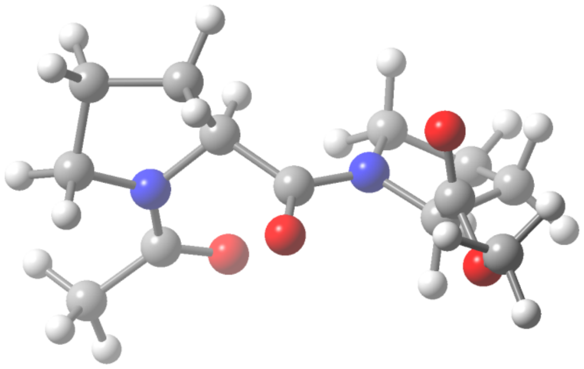

N	2.6278532	-0.1913646	0.1143066
C	1.4836178	-0.2685549	-0.8059134
C	1.6531187	-1.6503187	-1.4751793
C	3.1606148	-1.9268958	-1.3706121
C	3.5187843	-1.3556765	0.0061360
C	0.1592733	-0.1979529	-0.0331083
O	-0.0810192	-1.0179593	0.8615465
N	-0.7572737	0.7214816	-0.4126730
C	-2.0546249	0.7407831	0.2657002
C	-2.8078547	1.8868645	-0.4409360
C	-1.6769117	2.8522430	-0.8230796
C	-0.5393188	1.9152095	-1.2525324
C	-2.7788740	-0.5899985	0.1078403
O	-3.4841607	-0.8832580	1.2116533
O	-2.7782872	-1.2625427	-0.9031849
C	-4.2743566	-2.0886308	1.1468954
C	2.7855891	0.8922060	0.9129157
O	1.9813072	1.8346914	0.8800703
C	3.9890544	0.8947045	1.8374991
H	1.5605739	0.5346179	-1.5427622
H	1.2774785	-1.6512606	-2.5013449
H	1.0942876	-2.3937999	-0.8983194
H	3.7053631	-1.3825822	-2.1503705
H	3.4065604	-2.9886706	-1.4579762
H	4.5667142	-1.0567331	0.0831868
H	3.3045261	-2.0769617	0.8061433
H	-1.9242608	0.9456158	1.3334925
H	-3.2956998	1.4992974	-1.3426988
H	-3.5704094	2.3308211	0.2028609
H	-1.3672703	3.4329866	0.0531476
H	-1.9606923	3.5510092	-1.6144126
H	0.4465882	2.3353792	-1.0493895
H	-0.6143277	1.6542980	-2.3151147
H	-3.6271902	-2.9565403	0.9961154
H	-4.7864627	-2.1550553	2.1062894
H	-4.9960969	-2.0278366	0.3283023
H	4.0455395	-0.0261567	2.4275553
H	4.9191457	0.9767184	1.2629007
H	3.9101860	1.7526884	2.5065237

ProHyp endo/endo

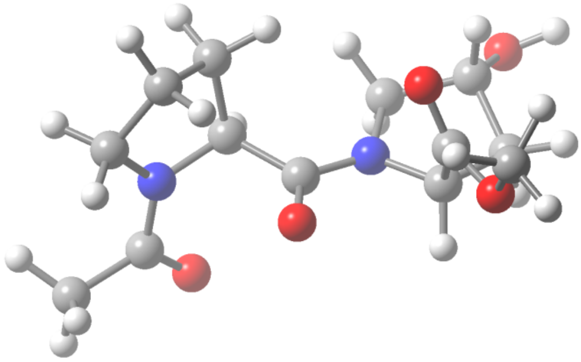

C	-1.5828195	0.5565683	0.6253206
C	-1.4530859	-0.0260895	2.0527830
C	-2.2221127	-1.3533835	1.9495535
C	-3.4096301	-1.0108549	1.0379456
N	-2.8798375	0.0614948	0.1804897
C	-0.4572878	0.0046485	-0.2610258
N	0.7054808	0.7034114	-0.2762116
O	-0.5727273	-1.0624639	-0.8702118
C	1.8719974	0.1111490	-0.9302927
C	2.9416132	1.2130941	-0.8082643
C	2.5682650	1.9194130	0.5064183
C	1.0332430	1.9161469	0.4838598
C	-3.4098400	0.5116794	-0.9853507
C	-4.7438909	-0.0832719	-1.3972978
O	-2.8256453	1.3626631	-1.6676413
C	2.2980865	-1.1568598	-0.1869415
O	2.1176139	-1.3582734	0.9979877
O	2.9548449	-1.9891615	-1.0059173
C	3.4787942	-3.1893909	-0.3963838
O	3.0219079	3.2587510	0.6080532
H	-1.5790846	1.6485779	0.6090375
H	-1.9334856	0.6510254	2.7669427
H	-0.4102095	-0.1620636	2.3525741
H	-2.5413481	-1.7403574	2.9205641
H	-1.5923343	-2.1050311	1.4641054
H	-4.2694155	-0.6465946	1.6152328
H	-3.7373103	-1.8667624	0.4412681
H	1.6512605	-0.1499740	-1.9667359
H	2.8335361	1.9248666	-1.6333779
H	3.9613309	0.8192011	-0.8184290
H	2.9166338	1.3187463	1.3574027
H	0.6740847	2.8159276	-0.0313660
H	0.6103734	1.8933878	1.4901327
H	-5.1297959	0.4799318	-2.2482034
H	-5.4715088	-0.0570563	-0.5792470
H	-4.6190166	-1.1316820	-1.6935651
H	4.1861617	-2.9336445	0.3964387
H	2.6642395	-3.7865472	0.0209334
H	3.9793638	-3.7299644	-1.1988186
H	3.9790758	3.2344559	0.7650542

ProHyp endo/exo

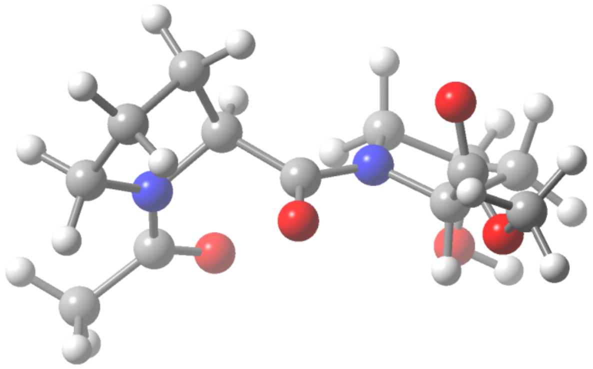

C	-1.5717346	-0.2947829	0.9066620
C	-1.7702277	-1.6336956	1.6534005
C	-2.5712033	-2.4917115	0.6604073
C	-3.5135500	-1.4793653	-0.0048343
N	-2.7371164	-0.2295980	0.0195602
C	-0.2752462	-0.3243358	0.0859961
N	0.7041414	0.5505275	0.4129598
O	-0.1058404	-1.1796372	-0.7921579
C	1.9909157	0.4663826	-0.2801059
C	2.8250765	1.5641139	0.4094289
C	1.7694700	2.6213483	0.7635917
C	0.5802604	1.7761777	1.2225338
C	-2.9893290	0.9053572	-0.6776673
C	-4.2242859	0.9041121	-1.5589146
O	-2.2369589	1.8870123	-0.5981018
C	2.6198667	-0.9092141	-0.1034345
O	2.5848169	-1.5569577	0.9232429
O	3.2832280	-1.2775695	-1.2097831
C	3.9822136	-2.5374672	-1.1288036
O	1.3437308	3.3437128	-0.3924172
H	-1.5856591	0.5539758	1.5912169
H	-2.3525705	-1.4497776	2.5626894
H	-0.8193101	-2.0876256	1.9451175
H	-3.1160203	-3.3076736	1.1428433
H	-1.8945661	-2.9095515	-0.0889190
H	-4.4442301	-1.3594182	0.5656255
H	-3.7746859	-1.7583347	-1.0293296
H	1.8668199	0.6713426	-1.3471872
H	3.6212323	1.9506130	-0.2317386
H	3.2746221	1.1692738	1.3274813
H	2.1103244	3.3041250	1.5523341
H	-0.3641750	2.2786516	1.0083532
H	0.6523237	1.5516753	2.2925028
H	-4.3725256	1.9103716	-1.9530940
H	-4.0986143	0.2081961	-2.3966834
H	-5.1155174	0.5927804	-1.0035921
H	4.4665910	-2.6662304	-2.0961635
H	4.7242776	-2.5103398	-0.3267595
H	3.2751190	-3.3496809	-0.9416329
H	2.1122202	3.8271185	-0.7365273

ProHyp exo/endo

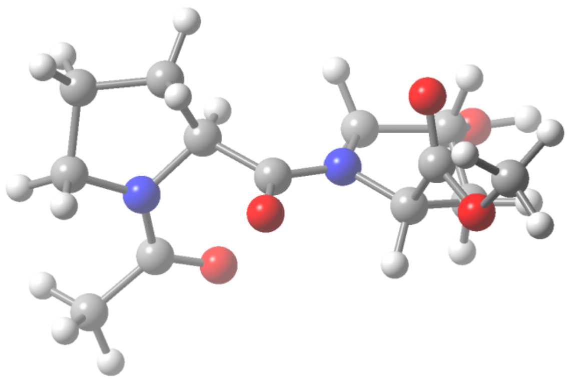

C	-1.6791517	-0.1982340	0.7920670
C	-1.9905627	-1.4043089	1.7042450
C	-3.5246692	-1.4895414	1.6916930
C	-3.8766998	-1.1206492	0.2458661
N	-2.8522747	-0.1222370	-0.0904770
C	-0.4094639	-0.4428819	-0.0359206
N	0.6400645	0.3945575	0.1328363
O	-0.3468583	-1.4061042	-0.8074126
C	1.8350533	0.2014321	-0.6880993
C	2.5921110	1.5367249	-0.5387188
C	2.1683506	2.0296318	0.8548617
C	0.6933569	1.6181782	0.9385215
C	-2.9107413	0.8293167	-1.0548758
C	-4.1549843	0.8508998	-1.9238071
O	-1.9870600	1.6406058	-1.2077729
C	2.6624338	-0.9729921	-0.1676506
O	2.6650186	-1.3721910	0.9791450
O	3.4351001	-1.4700447	-1.1453654
C	4.3306808	-2.5351842	-0.7600779
O	2.2641558	3.4322327	1.0399252
H	-1.6113961	0.7292212	1.3652554
H	-1.5670829	-1.2770100	2.7035779
H	-1.5617257	-2.3065666	1.2566385
H	-3.9549944	-0.7506790	2.3771678
H	-3.8993447	-2.4774724	1.9728874
H	-4.8809796	-0.7024174	0.1465547
H	-3.7960825	-1.9906922	-0.4195521
H	1.5595100	-0.0068423	-1.7237929
H	2.2292618	2.2497477	-1.2863022
H	3.6738180	1.4296552	-0.6553654
H	2.7387476	1.4906480	1.6242517
H	0.0577332	2.3957629	0.4983792
H	0.3797492	1.4379906	1.9690879
H	-5.0314302	1.1559931	-1.3401201
H	-4.0011258	1.5684073	-2.7309746
H	-4.3674577	-0.1365055	-2.3471549
H	4.8776070	-2.7989290	-1.6645983
H	5.0163309	-2.1909220	0.0183229
H	3.7618058	-3.3921606	-0.3907915
H	3.2053956	3.6561875	1.1132330

ProHyp exo/exo

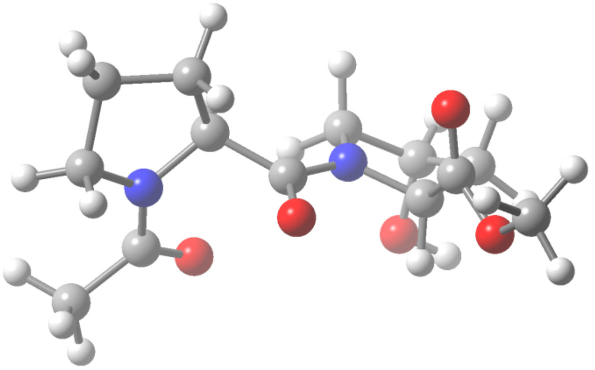

C	1.5833346	-0.3390975	-0.8092082
C	1.8301736	-1.7073968	-1.4834963
C	3.3502261	-1.9021149	-1.3732767
C	3.6720271	-1.3149396	0.0058259
N	2.7197553	-0.2004610	0.1113802
C	0.2567644	-0.3486232	-0.0380555
N	-0.7269245	0.4848248	-0.4498748
O	0.0737356	-1.1619460	0.8748211
C	-2.0313983	0.4225678	0.2112185
C	-2.8615144	1.4541216	-0.5764648
C	-1.8175672	2.5174018	-0.9455615
C	-0.5851387	1.6814193	-1.2986824
C	2.8123609	0.8892847	0.9116211
C	4.0101710	0.9603921	1.8404864
O	1.9543171	1.7828440	0.8755971
C	-2.6342500	-0.9723524	0.1144965
O	-2.5750891	-1.6836458	-0.8678259
O	-3.3076635	-1.2798407	1.2336132
C	-3.9852201	-2.5537106	1.2249535
O	-1.4666502	3.3194776	0.1829251
H	1.6142629	0.4706305	-1.5424362
H	1.4594152	-1.7243755	-2.5112467
H	1.3102941	-2.4830935	-0.9125106
H	3.8674445	-1.3275000	-2.1499952
H	3.6535079	-2.9488547	-1.4617153
H	4.7021062	-0.9604975	0.0876574
H	3.4931475	-2.0478441	0.8039780
H	-1.9428517	0.7044176	1.2644266
H	-3.6951340	1.8551689	0.0054433
H	-3.2587110	0.9928474	-1.4876801
H	-2.1381001	3.1426763	-1.7885870
H	0.3321351	2.2172981	-1.0495375
H	-0.5858320	1.4140409	-2.3610159
H	4.9366133	1.0929591	1.2694706
H	3.8810732	1.8134943	2.5079754
H	4.1150522	0.0448216	2.4322124
H	-4.4864751	-2.6253131	2.1896537
H	-4.7116719	-2.5934955	0.4092689
H	-3.2619684	-3.3646728	1.1066031
H	-2.2631371	3.7999171	0.4613126

HypPro endo/endo

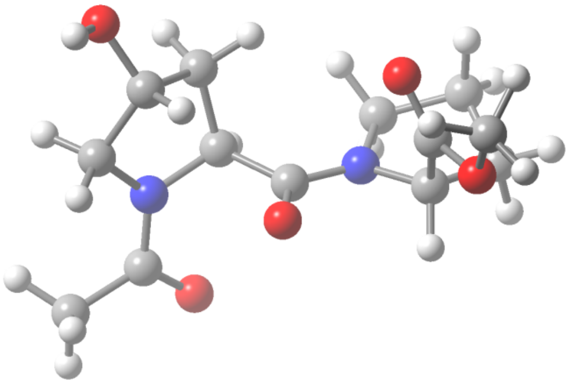

C	1.3730899	-0.3815200	0.7275262
C	1.3453835	0.9252259	1.5463625
C	2.0281138	1.9330336	0.6163950
C	3.1473613	1.1007405	-0.0447132
N	2.5950366	-0.2554818	-0.0633708
C	0.1400787	-0.4384962	-0.1873105
N	-0.9680126	-1.0297488	0.3185629
O	0.1381199	0.1029601	-1.2974030
C	-2.2234961	-0.9183978	-0.4173009
C	-3.1976993	-1.7867919	0.4083446
C	-2.6722908	-1.6246773	1.8430003
C	-1.1465656	-1.6068732	1.6659703
C	3.0152274	-1.3038899	-0.8181971
C	4.2633705	-1.0829776	-1.6515642
O	2.4007225	-2.3769761	-0.8134857
O	2.5046193	3.0375847	1.3680105
C	-2.6807967	0.5410905	-0.4514300
O	-2.3840798	1.3831051	0.3731421
O	-3.5077202	0.7529672	-1.4855754
C	-4.0712614	2.0791540	-1.5802791
H	1.4292065	-1.2739998	1.3534304
H	1.9461464	0.8154499	2.4557457
H	0.3331828	1.2293928	1.8253875
H	1.3167639	2.2553247	-0.1546012
H	4.0518438	1.1575230	0.5749775
H	3.3891272	1.4504663	-1.0523351
H	-2.1106621	-1.2709995	-1.4442043
H	-3.1109798	-2.8270621	0.0776520
H	-4.2383162	-1.4764790	0.2845800
H	-3.0011534	-2.4226348	2.5131250
H	-3.0050672	-0.6669274	2.2557400
H	-0.7226966	-2.6178512	1.6994852
H	-0.6524382	-0.9991758	2.4276884
H	5.0765272	-0.6503251	-1.0593358
H	4.5809031	-2.0411207	-2.0654388
H	4.0531273	-0.3924760	-2.4771104
H	2.7807105	3.7261474	0.7426291
H	-4.7073474	2.0641179	-2.4645823
H	-4.6586546	2.3060135	-0.6869657
H	-3.2757759	2.8203783	-1.6905431

HypPro endo/exo

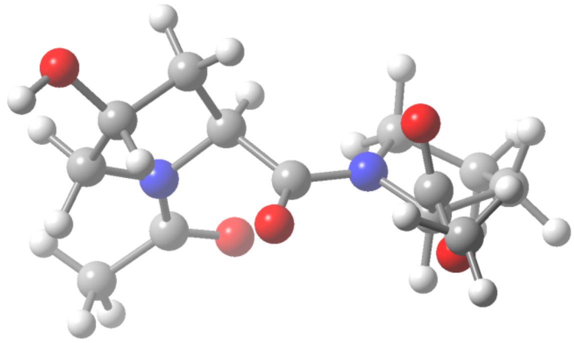

C	1.3378980	0.1199085	-0.7889242
C	1.6404935	-1.2495308	-1.4274961
C	2.4687984	-1.9816584	-0.3673591
C	3.3293810	-0.8550900	0.2418790
N	2.4763906	0.3282060	0.1161575
C	0.0281614	0.0571729	0.0097835
N	-0.9991779	0.8412868	-0.3845968
O	-0.0960986	-0.7516972	0.9391743
C	-2.2953831	0.6951967	0.2806475
C	-3.1973330	1.6957773	-0.4733352
C	-2.2107697	2.7912234	-0.9034845
C	-0.9542612	2.0020597	-1.2951944
C	2.6219770	1.5202039	0.7479678
C	3.8254605	1.6667093	1.6591862
O	1.8011374	2.4329631	0.5836778
O	3.2328603	-3.0102478	-0.9789654
C	-2.8146028	-0.7323524	0.1584289
O	-2.6809760	-1.4357619	-0.8224490
O	-3.5075841	-1.0823754	1.2528936
C	-4.1138297	-2.3914090	1.2174251
H	1.3115424	0.9155942	-1.5336084
H	2.2590280	-1.1122984	-2.3213866
H	0.7355223	-1.7924212	-1.7108652
H	1.8005615	-2.3836387	0.4022283
H	4.2511492	-0.7496068	-0.3455571
H	3.5971300	-1.0465979	1.2845783
H	-2.2120012	0.9497152	1.3421792
H	-4.0153387	2.0580676	0.1531377
H	-3.6253208	1.2062096	-1.3556350
H	-1.9866688	3.4495827	-0.0566085
H	-2.5875940	3.4059085	-1.7251048
H	-0.0341928	2.5650739	-1.1315363
H	-0.9999157	1.6709306	-2.3398323
H	4.7537421	1.3913985	1.1474012
H	3.8854964	2.7033994	1.9931557
H	3.7264551	1.0153176	2.5354565
H	3.6049590	-3.5628617	-0.2735969
H	-4.6350745	-2.4985011	2.1681400
H	-4.8160349	-2.4633530	0.3829172
H	-3.3445891	-3.1606808	1.1118160

HypPro exo/endo

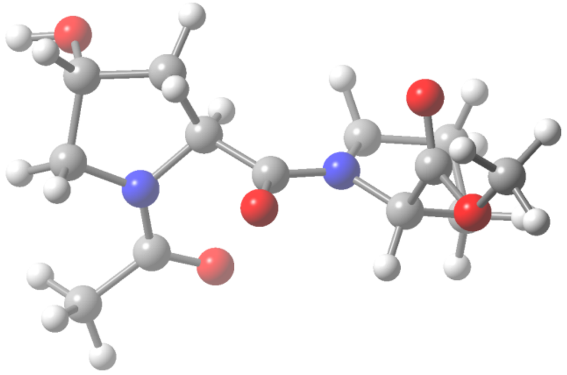

C	-1.3993410	-0.3500025	0.4445819
C	-1.7293038	-1.8472687	0.6110266
C	-3.2455542	-1.9142381	0.4309646
C	-3.4911038	-0.8925389	-0.6892290
N	-2.4837446	0.1382661	-0.4195773
C	-0.0413878	-0.1604927	-0.2464966
N	0.9287448	0.5051429	0.4187806
O	0.1527243	-0.6438480	-1.3679862
C	2.2100165	0.7348756	-0.2410347
C	2.8538610	1.8603886	0.6010266
C	2.2692386	1.6244068	2.0015018
C	0.8228281	1.1987246	1.7154942
C	-2.5184877	1.4413138	-0.7944291
C	-3.6893135	1.8751742	-1.6573048
O	-1.6321646	2.2307199	-0.4415743
O	-3.8308396	-1.4970720	1.6640639
C	3.0639575	-0.5315571	-0.2223866
O	2.9485092	-1.4459787	0.5680812
O	4.0074859	-0.4705234	-1.1762188
C	4.9369674	-1.5742500	-1.2136870
H	-1.4471870	0.1754249	1.3998796
H	-1.4127095	-2.2408627	1.5792524
H	-1.2404824	-2.4153443	-0.1857744
H	-3.5930965	-2.9157544	0.1463908
H	-4.5042275	-0.4838725	-0.6519583
H	-3.3352364	-1.3495419	-1.6754097
H	2.0593887	1.0283299	-1.2821319
H	2.5273958	2.8273032	0.2042148
H	3.9459375	1.8317301	0.5693676
H	2.3207039	2.5080885	2.6424184
H	2.8043937	0.8072609	2.4970249
H	0.1487698	2.0567650	1.6131739
H	0.4315130	0.5324104	2.4883012
H	-4.6213269	1.8609492	-1.0799473
H	-3.5053793	2.8928311	-2.0045126
H	-3.8239673	1.2127801	-2.5188357
H	-4.7951097	-1.4959259	1.5521310
H	4.4059486	-2.5098508	-1.4066723
H	5.6255402	-1.3496635	-2.0275925
H	5.4741443	-1.6505159	-0.2648355

HypPro exo/exo

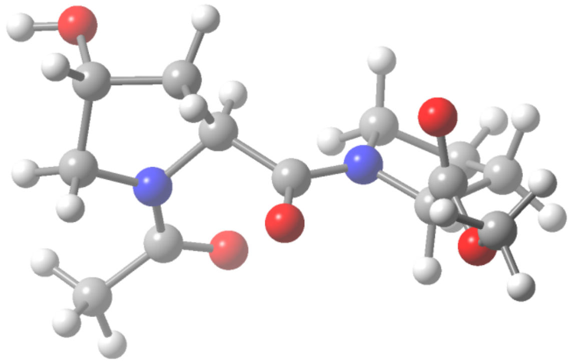

C	1.3334498	-0.2351104	-0.5372749
C	1.6209093	-1.6850601	-0.9801445
C	3.1253694	-1.8446931	-0.7644470
C	3.3551287	-1.0733189	0.5435238
N	2.3934319	0.0301434	0.4464483
C	-0.0518792	-0.1349334	0.1151252
N	-0.9702316	0.6809435	-0.4494500
O	-0.3302015	-0.8457037	1.0886845
C	-2.3174474	0.7226946	0.1227713
C	-3.0564224	1.7316388	-0.7808834
C	-1.9366037	2.6920450	-1.2061677
C	-0.7352524	1.7622355	-1.4259528
C	2.4503527	1.2270383	1.0808910
C	3.5884125	1.4290412	2.0639871
O	1.6083726	2.1084598	0.8600784
O	3.7742986	-1.2167144	-1.8696513
C	-2.9745692	-0.6512101	0.0868730
O	-2.8668942	-1.4461733	-0.8246099
O	-3.7538348	-0.8359547	1.1640898
C	-4.4897730	-2.0765348	1.1969593
H	1.4383561	0.4562531	-1.3756169
H	1.3320969	-1.8698539	-2.0168696
H	1.0812152	-2.3766321	-0.3269685
H	3.4290634	-2.8952944	-0.6712264
H	4.3814699	-0.7061930	0.6251634
H	3.1416276	-1.7082461	1.4134788
H	-2.2798521	1.0671651	1.1615012
H	-3.8821228	2.2183603	-0.2570818
H	-3.4588588	1.2086507	-1.6560438
H	-1.7167693	3.3952090	-0.3949014
H	-2.1851190	3.2683188	-2.1012296
H	0.2161034	2.2516357	-1.2138555
H	-0.7214260	1.3595168	-2.4459601
H	3.4187425	2.3617731	2.6035665
H	3.6588739	0.6007056	2.7766510
H	4.5472088	1.4945859	1.5362251
H	4.7326904	-1.2733524	-1.7260047
H	-5.1492885	-2.1514209	0.3285374
H	-3.8001838	-2.9246073	1.2027663
H	-5.0702066	-2.0484874	2.1185868

HypHyp endo/endo

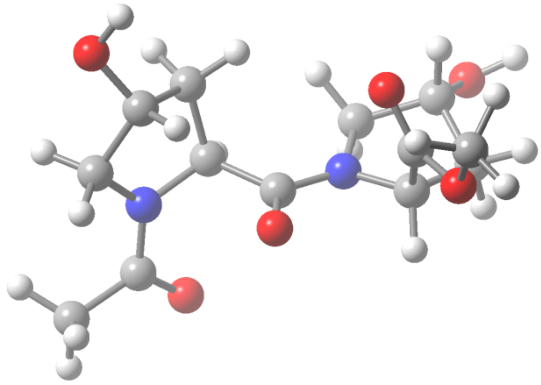

C	1.4426866	-0.5938364	0.5531700
C	1.4781259	0.3849377	1.7453194
C	2.3295104	1.5502627	1.2094637
C	3.4031220	0.8466566	0.3679902
N	2.7280049	-0.3646777	-0.1023831
C	3.1111732	-1.1696493	-1.1277339
C	4.4371262	-0.8426769	-1.7880921
O	2.4030833	-2.1158438	-1.4913073
C	0.2838684	-0.2234211	-0.3840288
N	-0.9117875	-0.8129627	-0.1403025
O	0.4140763	0.6296085	-1.2666744
C	-2.0896162	-0.3390398	-0.8670538
C	-3.2037797	-1.2890603	-0.3887425
C	-2.7759205	-1.6357274	1.0476970
C	-1.2480204	-1.7451448	0.9436923
C	-2.3934361	1.1094372	-0.4778052
O	-2.1111676	1.6121882	0.5920737
O	-3.0699931	1.7303356	-1.4529573
C	-3.4859528	3.0841244	-1.1680893
O	2.9659313	2.3270535	2.2119899
O	-3.2900936	-2.8625333	1.5378160
H	1.3677436	-1.6384141	0.8615751
H	1.9965693	-0.0715916	2.5949348
H	0.4803334	0.6993614	2.0648714
H	1.7126918	2.1786606	0.5541694
H	4.2634145	0.6041413	1.0059967
H	3.7488480	1.4649394	-0.4642470
H	5.2352716	-0.7020704	-1.0517262
H	4.7012414	-1.6576976	-2.4633914
H	4.3554119	0.0848713	-2.3673987
H	-1.9301160	-0.3844324	-1.9457117
H	-3.1916644	-2.2039414	-0.9902148
H	-4.1995500	-0.8425392	-0.4521957
H	-3.0324339	-0.8042452	1.7179374
H	-0.9731915	-2.7741150	0.6802144
H	-0.7548033	-1.4757746	1.8798834
H	-4.0202963	3.4184555	-2.0565427
H	-4.1413180	3.1026003	-0.2937677
H	-2.6132967	3.7152512	-0.9828178
H	2.2859470	2.8740685	2.6359372
H	-4.2323377	-2.7316898	1.7290460

HypHyp endo/exo

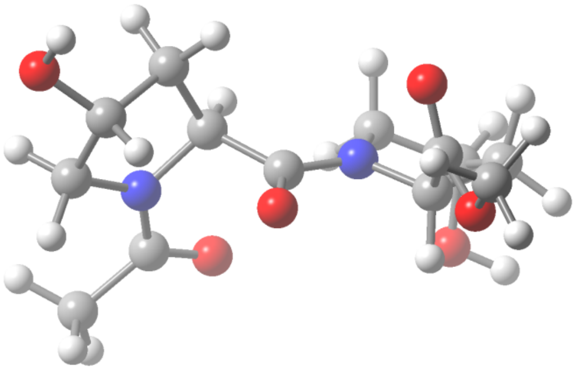

C	1.4372373	0.0619550	-0.7977616
C	1.8906698	-1.2768487	-1.4121583
C	2.7842741	-1.9011507	-0.3253010
C	3.5069646	-0.6878818	0.2741816
N	2.5386826	0.3987192	0.1148525
C	2.5640557	1.6141367	0.7176533
C	3.7386428	1.8968246	1.6343926
O	1.6614366	2.4396094	0.5245872
C	0.1325407	-0.1258758	-0.0112737
N	-0.9648632	0.5461486	-0.4254926
O	0.0788294	-0.9352589	0.9237668
C	-2.2456094	0.2912512	0.2370360
C	-3.2256250	1.1788598	-0.5556945
C	-2.3499860	2.3717449	-0.9646351
C	-1.0203150	1.7098005	-1.3288687
C	-2.6260457	-1.1809179	0.1480337
O	-2.4414681	-1.8834247	-0.8251816
O	-3.2608972	-1.5782728	1.2608238
C	-3.7344172	-2.9415734	1.2574221
O	3.7549126	-2.8145211	-0.8166981
O	-2.0938852	3.2360388	0.1428157
H	1.3374684	0.8419343	-1.5530505
H	2.5039083	-1.0879713	-2.2997793
H	1.0501636	-1.9155307	-1.6969733
H	2.1535993	-2.3638399	0.4422977
H	4.4245915	-0.4933556	-0.2974058
H	3.7742298	-0.8371978	1.3232039
H	3.6910733	1.2617441	2.5267789
H	4.6943663	1.6969598	1.1383383
H	3.6977013	2.9427519	1.9412552
H	-2.1991726	0.5884148	1.2883580
H	-4.0979410	1.4698807	0.0346462
H	-3.5687100	0.6484093	-1.4510358
H	-2.7710396	2.9285977	-1.8114090
H	-0.1812836	2.3798695	-1.1364890
H	-1.0174235	1.3946843	-2.3782175
H	-4.2251225	-3.0832625	2.2197608
H	-4.4409174	-3.0971359	0.4380955
H	-2.8943062	-3.6322334	1.1486238
H	3.2916673	-3.6328542	-1.0554432
H	-2.9453326	3.6007483	0.4338565

HypHyp exo/endo

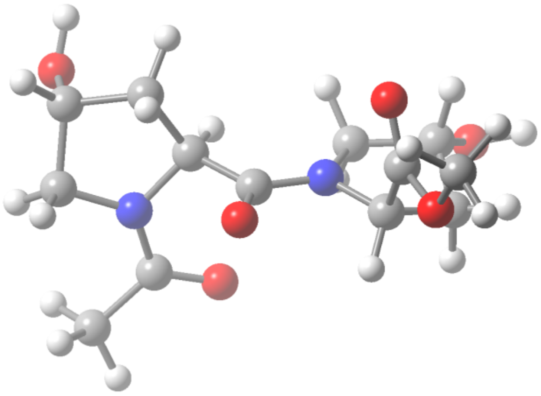

C	-1.5208866	-0.1964715	0.5115318
C	-1.9213457	-1.4460902	1.3194010
C	-3.4532073	-1.4589132	1.2315877
C	-3.7074024	-1.0112441	-0.2074140
N	-2.6191029	-0.0575599	-0.4548944
C	-2.6004813	0.9529142	-1.3595275
C	-3.7917657	1.0581479	-2.2937421
O	-1.6505173	1.7455464	-1.4096871
C	-0.1903467	-0.4163644	-0.2204610
N	0.8470330	0.3999331	0.0750547
O	-0.0722604	-1.3422768	-1.0300395
C	2.1059706	0.2305585	-0.6495288
C	2.8632675	1.5429541	-0.3627287
C	2.3284455	1.9653487	1.0156911
C	0.8466396	1.5748596	0.9520605
C	2.8700237	-0.9841554	-0.1244396
O	2.7627857	-1.4478405	0.9926634
O	3.7242636	-1.4364097	-1.0548202
C	4.5700602	-2.5353417	-0.6522482
O	-4.0407301	-0.4709066	2.0799577
O	2.4257472	3.3535195	1.2875023
H	-1.4951721	0.6925300	1.1443464
H	-1.5552759	-1.4122382	2.3491304
H	-1.5165365	-2.3388614	0.8319850
H	-3.8769903	-2.4518744	1.4297397
H	-4.6873954	-0.5393863	-0.3049293
H	-3.6511305	-1.8627721	-0.8966122
H	-4.6865020	1.3741798	-1.7444250
H	-3.5671285	1.8022361	-3.0591821
H	-4.0180584	0.0985686	-2.7702998
H	1.9187767	0.0825113	-1.7149353
H	2.5737185	2.3015029	-1.0973294
H	3.9495287	1.4242352	-0.3936192
H	2.8245774	1.3747563	1.7985340
H	0.2596953	2.3869264	0.5066837
H	0.4454838	1.3412181	1.9407452
H	5.1852729	-2.2462664	0.2035894
H	3.9611600	-3.4029862	-0.3861735
H	5.1945378	-2.7557708	-1.5172831
H	-3.8015231	-0.6906518	2.9948014
H	3.3593148	3.5552552	1.4584037

References

[1] J. Zaminer, C. Brockmann, P. Huy, R. Opitz, C. Reuter, M. Beyermann, C. Freund, M. Müller, H. Oschkinat, R. Kühne, H.-G. Schmalz, *Angew. Chem. Int. Ed.* **2010**, *49*, 7111-7115.

[2] C. Reuter, R. Opitz, A. Soicke, S. Dohmen, M. Barone, S. Chiha, M. T. Klein, J.-M. Neudörfl, R. Kühne, H.-G. Schmalz, *Chem. Eur. J.* **2015**, *21*, 8464-8470.

[3] S. Chiha, A. Soicke, M. Barone, M. Müller, J. Bruns, R. Opitz, J.-M. Neudörfl, R. Kühne, H.-G. Schmalz, *Eur. J. Org. Chem.* **2018**, *4*, 455-460.

[4] I. Coin, M. Beyermann, M. Bienert, *Nat. Protoc.* **2007**, *2*, 3247-3256.

[5] J. L. S. Lopes, A. J. Miles, L. Whitmore, B. A. Wallace, *Protein Sci.* **2014**, *23*, 1765-1772.

[6] A. V. Persikov, Y. Xu, B. Brodsky, *Protein Sci.* **2004**, *13*, 893-902.

[7] J. Engel, H. T. Chen, D. J. Prockop, H. Klump, *Biopolymers* **1977**, *16*, 601-622.

[8] S. Frank, R. A. Kammerer, D. Mechling, T. Schulthess, R. Landwehr, J. Bann, Y. Guo, A. Lustig, H. P. Bächinger, J. Engel, *J. Mol. Biol.* **2001**, *308*, 1081-1089.

[9] L. Lasdon, A. Waren, *Generalized Reduced Gradient Nonlinear Solving Method for MS Excel Solver tool*, Frontline Systems, Inc., Incline Village, NV, **2009**.

[10] *MacroModel. v11.5*, Release 2017-1, Schrödinger, LLC, New York, **2017**.

[11] M. Cutini, M. Corno, P. Ugliengo, *J. Chem. Theory Comput.* **2017**, *13*, 370-379.

[12] S. Grimme, J. Antony, S. Ehrlich, H. Krieg, *J. Chem. Phys.* **2010**, *132*, 154104.

[13] A. d. Bochevarov, E. Harder, T. F. Hughes, J. R. Greenwood, D. A. Braden, D. M. Philipp, D. Rinaldo, M. d. Halls, J. Zhang, R. A. Friesner, *Int. J. Quantum Chem.* **2013**, *113*, 2110-2142.

[14] *Gaussian 16, Revision A.03*, M. J. Frisch, G. W. Trucks, H. B. Schlegel, G. E. Scuseria, M. A. Robb, J. R. Cheeseman, G. Scalmani, V. Barone, G. A. Petersson, H. Nakatsuji, X. Li, M. Caricato, A. V. Marenich, J. Bloino, B. G. Janesko, R. Gomperts, B. Mennucci, H. P. Hratchian, J. V. Ortiz, A. F. Izmaylov, J. L. Sonnenberg, D. Williams-Young, F. Ding, F. Lipparini, F. Egidi, J. Goings, B. Peng, A. Petrone, T. Henderson, D. Ranasinghe, V. G. Zakrzewski, J. Gao, N. Rega, G. Zheng, W. Liang, M. Hada, M. Ehara, K. Toyota, R. Fukuda, J. Hasegawa, M. Ishida, T. Nakajima, Y. Honda, O. Kitao, H. Nakai, T. Vreven, K. Throssell, J. A. Montgomery, Jr., J. E. Peralta, F. Ogliaro, M. J. Bearpark, J. J. Heyd, E. N. Brothers, K. N. Kudin, V. N. Staroverov, T. A. Keith, R. Kobayashi, J. Normand, K. Raghavachari, A. P. Rendell, J. C. Burant, S. S. Iyengar, J. Tomasi, M. Cossi, J. M. Millam, M. Klene, C. Adamo, R. Cammi, J. W. Ochterski, R. L. Martin, K. Morokuma, O. Farkas, J. B. Foresman, D. J. Fox, Gaussian, Inc., Wallingford CT, **2016**.

[15] M. L. DeRider, S. J. Wilkens, M. J. Waddell, L. E. Bretscher, F. Weinhold, R. T. Raines, J. L. Markley, *J. Am. Chem. Soc.* **2002**, *124*, 2497-2505.

[16] M. D. Shoulders, R. T. Raines, *Annu. Rev. Biochem.* **2009**, *78*, 929-958.

[17] R. Improta, C. Benzi, V. Barone, *J. Am. Chem. Soc.* **2001**, *123*, 12568-12577.

[18] R. Berisio, L. Vitagliano, L. Mazzarella, A. Zagari, *Protein Sci.* **2002**, *11*, 262-270.

[19] *Origin(Pro)*, OriginLab Corporation, Northhampton, MA, USA, **2010**.

Author Contributions

Andreas Maaßen conducted the chemical syntheses and characterization of ProMs and CMPs, determined the T_m by CD denaturation studies, performed the computer-assisted conformational analysis, developed the structure-activity model, performed the HSP47 assays, wrote major parts of the manuscript (incl. preparation of all graphics).

Jan Gebauer performed the X-ray crystallographic analysis of CMPs and designed the HSP47 binding assay.

Elena Theres Abraham expressed and purified HSP47.

Isabelle Grimm contributed to the synthesis of Boc-ep-ProM-1-O*t*Bu.

Jörg-Martin Neudörfl performed the X-ray crystallographic analyses of small molecules (ProMs).

Ines Neundorf contributed to the research design and provided the methods for peptide synthesis.

Ronald Kühne designed ProM1 and ProM2.

Ulrich Baumann and Hans-Günther Schmalz designed and supervised the research project and contributed to the refinement of the manuscript.
